# High-throughput discovery of organic cages and catenanes using computational screening fused with robotic synthesis

**DOI:** 10.1038/s41467-018-05271-9

**Published:** 2018-07-20

**Authors:** R. L. Greenaway, V. Santolini, M. J. Bennison, B. M. Alston, C. J. Pugh, M. A. Little, M. Miklitz, E. G. B. Eden-Rump, R. Clowes, A. Shakil, H. J. Cuthbertson, H. Armstrong, M. E. Briggs, K. E. Jelfs, A. I. Cooper

**Affiliations:** 1 Department of Chemistry and Materials Innovation Factory, University of Liverpool, 51 Oxford Street, Liverpool, L7 3NY UK; 2 Department of Chemistry, Imperial College London, South Kensington, London, SW7 2AZ UK

## Abstract

Supramolecular synthesis is a powerful strategy for assembling complex molecules, but to do this by targeted design is challenging. This is because multicomponent assembly reactions have the potential to form a wide variety of products. High-throughput screening can explore a broad synthetic space, but this is inefficient and inelegant when applied blindly. Here we fuse computation with robotic synthesis to create a hybrid discovery workflow for discovering new organic cage molecules, and by extension, other supramolecular systems. A total of 78 precursor combinations were investigated by computation and experiment, leading to 33 cages that were formed cleanly in one-pot syntheses. Comparison of calculations with experimental outcomes across this broad library shows that computation has the power to focus experiments, for example by identifying linkers that are less likely to be reliable for cage formation. Screening also led to the unplanned discovery of a new cage topology—doubly bridged, triply interlocked cage catenanes.

## Abstract (web summary)

Supramolecular assemblies remain of great importance to a variety of fields, yet their targeted design and synthesis remains highly challenging. Here, Cooper and colleagues combine computational screening with high-throughput robotic synthesis and discover 33 new organic cage molecules that form cleanly in one-pot syntheses.

## Introduction

Supramolecular self-assembly is a powerful approach for generating complex organic and organometallic molecules, such as macrocycles^1,2^, cages^3–6^, catenanes^7^, rotaxanes^8^, molecular knots^9^, and molecular machines^10,11^. The targeted design of such molecules, however, can be very challenging, particularly as they become more elaborate^12,13^. Intuitive design strategies often fail as the system complexity increases and the number of possible self-assembled structures proliferates. Likewise, de novo computational design strategies are hampered by the size of such molecules and their conformational complexity^14–16^. As a result, the discovery of new self-assembled molecules can be relatively slow and labour intensive.

**Fig. 1:**
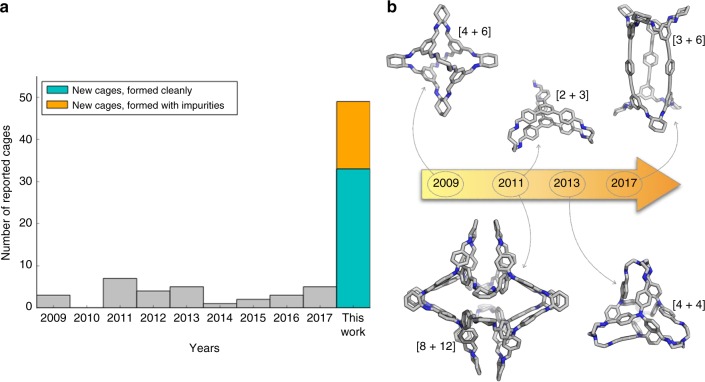
High-throughput methods fused with computation provides a step-change in the discovery rate for new cage molecules. **a** Timeline shows the number of organic cage molecules reported by our research group using traditional, iterative experiments in period 2009–2017 (grey bars; total of 30 cages). The number of cage molecules synthesised in this new study is also shown: we achieved clean conversion to 33 cages (teal bar) plus partial formation of 16 others (orange bar). **b** Timeline showing variation in cage topologies studied by our group during the same period

Porous organic cages (POCs) are a class of self-assembled molecules that show potential for molecular separation^17^, sensing^18,19^, and as building blocks for new materials, such as porous liquids^20^. However, despite significant interest in this area, the discovery rate for new cages has been relatively modest, reflecting the challenge in designing wholly new systems from scratch. New structures often involve small, iterative changes to known molecules. To relate this to our own activity, since 2009 we have published an average of three new cage molecules per year (Fig. 1a), with a variation on the cage topology every couple of years or so (Fig. 1b)^4,21–24^. Again, most of these molecules were discovered by making small, iterative structural changes to the precursors^25–27^. A bottleneck to advancing this area, as in many other areas of supramolecular assembly, is our ability to reliably design new self-assembled organic molecules. Pure in silico design strategies cannot yet cope with the complexity of supramolecular materials. Blind experimental screening approaches, while potentially rapid, are inefficient. Our strategy here is the close fusion of computational screening with automated synthesis and measurement protocols to create an integrated workflow for supramolecular materials discovery.

There have been significant advances in the use of computational design strategies for POCs and for porous materials in general^16,28–31^, along with a less widespread adoption in the use of high-throughput automation for materials synthesis^32–35^. Computation allows the prediction of the most likely cage topology by studying the relative energies of different molecular assemblies or their formation energies^36,37^. Calculations can determine a priori whether a candidate cage structure will exhibit shape-persistence, and hence perhaps be porous^22^, and predict the effect of solvent scaffolding on molecular conformation^38^. Implicit solvation models, such as a polarisable continuum model (PCM) can be used to account for the electrostatic interactions between cages and the surrounding solvent, and have previously been found to stabilise the cage molecules relative to consideration in the gas phase^37^. However, there are also significant limitations to de novo computational design: for example, it is currently challenging to fully include the effect of solvent choice, particularly for large studies across a broad range of starting materials. Our strategy, therefore, was to identify the affordable elements of computational prediction that are of most value for fusion with high-throughput automated synthesis methods.

We report here a new family of organic cages discovered using this hybrid approach. A total of 78 possible imine cage combinations were investigated based on an array of three candidate amines and 26 candidate aldehydes. First, simulations were used to determine the most likely cage topology for each of the three types of amine–aldehyde combinations. Computation suggested clear topological preferences for some precursor combinations, as manifested by large energy differences, while the topological preferences for other combinations were less clear cut, suggesting the potential for mixed products. However, in this study none of the candidate reactions were discounted: rather, all combinations were trialled experimentally to test our predictions, like a training set. Overall, 33 cages were synthesised in pure form, and a further 16 cages could be identified along with side-products; this significantly exceeds the total number of cage molecules reported by our research group over the last 9 years (Fig. 1a). Successful experimental ‘hits’ were analysed with further simulations to identify candidate shape-persistent cages prior to scale-up and characterisation. We present single crystal X-ray diffraction for 18 of these structures, and there is excellent agreement with our a priori computational structure predictions. We also use post-experiment computation to explain why some systems formed unanticipated topologies or mixtures, such as a unique doubly bridged, triply interlocked cage catenane. This surprising result illustrates the power of high-throughput experimental screening to reveal unexpected molecular structures. Finally, we propose a more generalised workflow for the streamlined discovery of supramolecular materials based on what we learnt from this study.

## Results

### Computational prediction of model cage topologies

**Fig. 2:**
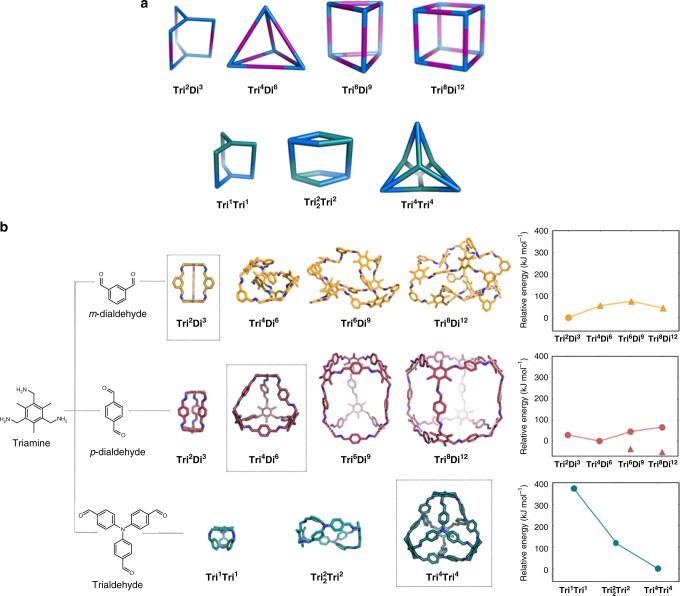
Reaction schemes and computational modelling for three model covalent organic cages. **a** Scheme showing possible cage topologies; (top) candidate topologies for tritopic + ditopic reactions (**Tri**^**x**^**Di**^**y**^) and (bottom) for tritopic + tritopic reactions (**Tri**^**x**^**Tri**^**y**^). Tritopic precursors are shown in blue or cyan, ditopic precursors in purple. **b** Computationally derived structures for the possible topological combinations of the tritopic triamine linker ((2,4,6-trimethylbenzene-1,3,5-triyl)trimethanamine), with *m*-dialdehyde (isophthalaldehyde), *p*-dialdehyde (terephthalaldehyde), and trialdehyde (tris(4-formylphenyl)amine), respectively. The energetically preferred topologies have a grey box around them. The plots (right) show the relative internal energies calculated for the different topologies, plotted on a common energy scale. For topologies where both collapsed and open conformations are computed, the energy of the collapsed conformation is indicated by a triangle symbol; open conformations are indicated by circle symbols. For the *p*-dialdehyde, only open conformations are shown in the images, collapsed **Tri**^**6**^**Di**^**9**^and **Tri**^**8**^**Di**^**12**^ topologies are shown in Supplementary Fig. 1. **Tri**^**2**^**Di**^**3**^ cages are shown with orange carbons, **Tri**^**4**^**Di**^**6**^ with maroon, and **Tri**^**4**^**Tri**^**4**^ with teal. All nitrogens are blue and hydrogens are omitted

**Fig. 3:**
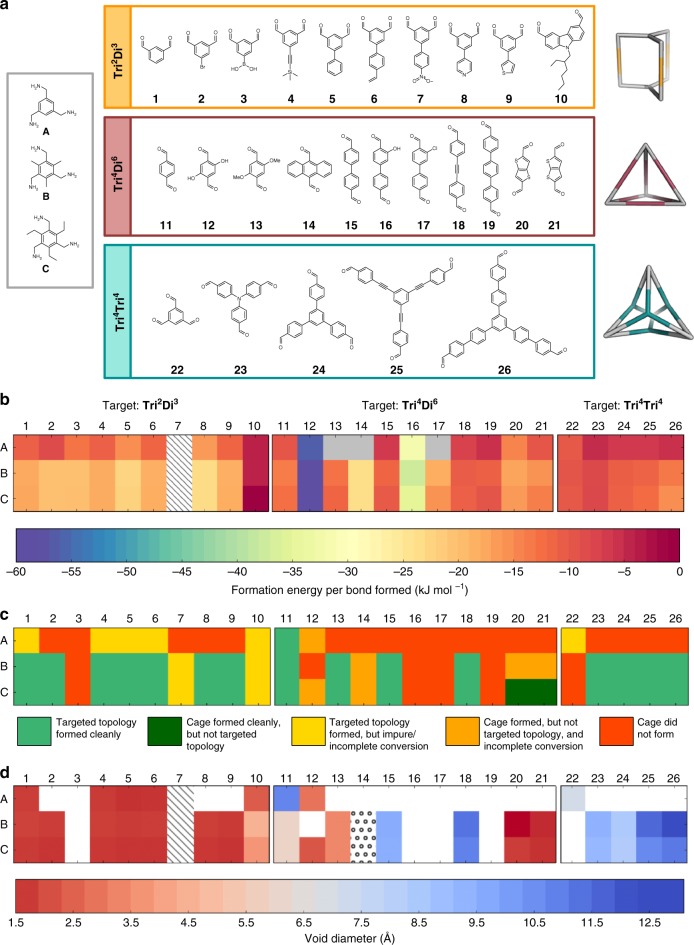
Cage precursors, target cage topologies, and results of high-throughput computation and synthesis screening. **a** Three triamines (**A**–**C**) were combined with three types of aldehydes to target **Tri**^**2**^**Di**^**3**^ capsules (**1**–**10**), **Tri**^**4**^**Di**^**6**^ tetrahedrons (**11**–**21**), and **Tri**^**4**^**Tri**^**4**^ tetrapods (**22**–**26**). **b** DFT formation energies per imine bond formed for the target cage topologies. These energies were not calculated for molecules that lacked shape persistence and collapsed (grey boxes). Cross-hatched squares denote three molecules excluded from the calculations because the force field was unreliable. **c** Results of the high-throughput synthesis screen. (i) light green squares—targeted cage topology was formed cleanly (31 examples); (ii) dark green squares—cage product was formed cleanly, but with topology that was not targeted (two examples); (iii) yellow squares—target topology formed but product impure or there was incomplete conversion (10 examples); (iv) orange squares—alternative topology formed and either impure or incomplete conversion (six examples); (v) red squares—no cage was formed (29 examples; 18 of these for triamine **A**). Cages that went against our targeted topology assumptions and formed an alternative topology are: **A12**, **C12**, **C14**, **B20**, **C20**, **B21**, and **C21** (**Tri**^**2**^**Di**^**3**^ observed instead of targeted **Tri**^**4**^**Di**^**6**^) and **B14** (formed a mixture of **Tri**^**2**^**Di**^**3**^ and **Tri**^**4**^**Di**^**6**^). **d** Void diameters as calculated from a priori computational models for cages that were formed in the subsequent reaction screen. White squares correspond to combinations where no cage was formed experimentally; squares filled with circles correspond to cages calculated to have no void

Prior to any experiments, we used computation to assess the possible model cage topologies (see Supplementary Methods) that might be formed by the reaction of a triamine precursor, (2,4,6-trimethylbenzene-1,3,5-triyl)trimethanamine, with a representative example of the three different aldehyde types; *meta* and *para*dialdehydes, and trigonal trialdehydes (Fig. 2), investigated in the high-throughput screen (Fig. 3a). These precursors were selected for the representative examples as the triamine is neither the most flexible, nor the most pre-configured, the *meta* and *para* dialdehydes the simplest linkers in the subsets, and the trigonal trialdehyde previously used to form tetrapods with a more flexible triamine linker^23^. We used our previous nomenclature for the cage topologies (Supplementary Note 1);^36^ that is, **Tri**^**x**^**Di**^**y**^ and **Tri**^**x**^**Tri**^**y**^, where the superscripts signify the number of tri-topic and di-topic precursors incorporated into the cage.

The relative internal energies were computed for each possible topology (Fig. 2b, right; Supplementary Note 2). Since the cages are formed by reversible imine chemistry, one might expect thermodynamic products to be formed—that is, the topologies with the lowest relative internal energy. These calculations do not take into account the potential effect of reaction solvent. We chose not to include an implicit solvation model so as to explore only the effect of the internal energy of the cages’ structures, especially since many of the reactions here were attempted in multiple solvents, each of which would have a differing dielectric constant. Ideally, we would want to geometry optimise each structure with the implicit solvation model, to explore how the conformation adapts to the electrostatic influence of each solvent, however this would be prohibitively computationally expensive for the large number of systems here.

For cages involving the *m*-dialdehyde, an energetic preference of 43 kJ mol^−1^ per [2 + 3] unit was found for a **Tri**^**2**^**Di**^**3**^ capsule topology (Fig. 2b, top row). The resulting cage was shape persistent; that is, it did not collapse. Larger candidate cage topologies (e.g., **Tri**^**4**^**Di**^**6**^) were all found to be higher in energy and non-shape-persistent, with the lowest energy conformations being collapsed with no internal void. This predicted preference for a **Tri**^**2**^**Di**^**3**^ topology with (2,4,6-trimethylbenzene-1,3,5-triyl)trimethanamine agrees with previous studies on reactions of this amine with isophthalaldehyde^39,40^, and alternative *meta* dialdehydes^41^, which form **Tri**^**2**^**Di**^**3**^ capsules.

For cages involving the *p*-dialdehyde (Fig. 2b, middle row), the **Tri**^**4**^**Di**^**6**^ tetrahedron topology was predicted to be energetically favoured by 29 kJ mol^−1^ per [2 + 3] unit with respect to other ‘open’ topologies, while the larger cage topologies collapsed to give lower energy forms (triangle points in Fig. 2b); their open conformations (circle points in Fig. 2b), which might be expected to dominate in solution^38^, were higher in energy than the uncollapsed **Tri**^**4**^**Di**^**6**^ topology.

For cages that combined the trialdehyde with the tritopic amine (Fig. 2b, bottom row), three potential cage topologies were considered, **Tri**^**1**^**Tri**^**1**^, **Tri**^**2**^_**2**_**Tri**^**2**^, and **Tri**^**4**^**Tri**^**4**^. The **Tri**^**4**^**Tri**^**4**^ ‘tetrapod’ topology was predicted to be much more stable by 118 kJ mol^−1^ per [1 + 1] unit.

The magnitude of the topological preference based on the relative energy differences for these three systems was ranked as **Tri**^**4**^**Tri**^**4**^ (trialdehyde) > **Tri**^**2**^**Di**^**3**^ (*m*-dialdehyde) > **Tri**^**4**^**Di**^**6**^ (*p*-dialdehyde). That is, the energetic preference for the **Tri**^**4**^**Tri**^**4**^ topology was predicted to be the most clear cut, suggesting a more reliable design basis for cages based on this topology, at least for this model reaction.

We next developed a robust synthesis method for these three model cages (Supplementary Note 3). The reaction conditions, which use only moderately high dilution and modest temperatures, were selected to be easily translated onto a robotic synthesis platform. Analysis of the cages by high-resolution mass spectroscopy (HRMS) confirmed that the experimental cage topologies agreed with the computational predictions; that is, for these three model systems at least, relative energies are a good predictor of cage topology preference.

### High-throughput cage synthesis

Using our optimised reaction conditions, we screened a much broader range of aldehydes (**1**–**26**) and amines (**A**–**C**) for cage formation using a synthesis robot (Fig. 3, Supplementary Fig. 2). For all combinations, the target cage topology was the one that was observed for the representative *m*-dialdehyde, *p*-dialdehyde, and trialdehyde in the model study, above. These three model combinations will be referred to henceforth as **B1, B11**, and **B23**, respectively (Fig. 3a).

We explored two additional analogues of triamine **B**, both with less (**A**) and more (**C**) alkyl substitution on the arene ring; this was designed to introduce different degrees of flexibility and structural pre-configuration. For the targeted **Tri**^**2**^**Di**^**3**^ analogues of **B1**, we explored ten different *m*-dialdehydes with various substituents at the five position (**1**–**9**), plus a carbazole dialdehyde, **10** (Fig. 3a, top panel). For the targeted **Tri**^**4**^**Di**^**6**^ analogues of **B11**, we studied 11 linear dialdehydes (**11**–**21**) containing different functionalities and degrees of rotational flexibility (Fig. 3a, middle panel). For the **Tri**^**4**^**Tri**^**4**^ analogues of **B23**, we studied five tritopic aldehydes (**22**–**26**) with various lengths and flexibility (Fig. 3a, bottom panel). In total, these three amines permutated with 26 aldehydes gave 78 reaction combinations.

Prior to the high-throughput experimental screen, models for all but three of the 78 target cages were constructed in silico and analysed computationally (Supplementary Note 4). Cages containing the di-topic linker **7** were omitted because the nitro-group was poorly described by the forcefield^42^. We also simplified **10** by removing the external alkyl substituents. We assessed the formation energies of the hypothetical cages and screened them for conformation, size, and shape persistence, the latter to identify cages with voids that are stable to desolvation (see Supplementary Tables 1–2). For each possible outcome, we searched for the low energy conformations and then minimised those structures using DFT calculations at the PBE+D3/TZVP level, before calculating the formation energy per imine bond formed, as shown in Fig. 3b. This energy normalisation using formation energies, rather than relative energies per sub-unit, allows us to directly compare the energies of the cages regardless of their size or topology. We chose the PBE+D3/TZVP level of theory as a compromise between accuracy and the ability for us to run the large number of calculations required in a high-throughput screen. Taken in isolation, we did not expect these formation energies to be generally predictive of experimental ‘hits’, due to the exclusion of factors, such as solvation effects and phase behaviour, coupled with the potential inaccuracies of DFT formation energies. Rather, we calculated these formation energies to establish whether they might play a more statistical role in guiding high-throughput screening studies in combination with other computational and experimental information.

The calculated formation energy per imine bond was found to be negative in all cases, although some hypothetical cages were much more energetically favoured than others. The calculated energy per imine bond ranged from ∼−1 to −58 kJ mol^−1^, and there was good correlation between cages with a less energetically favoured formation energy (dark red in Fig. 3b) and cages that did not form experimentally (red in Fig. 3c). Outliers for this trend include **A16**, **B16**, and **C16**, which had favourable formation energies (average −34.7 kJ mol^−1^), but yet were not observed. This could be due to factors not considered in determining the formation energy of the complete cage alone, for instance, a “kinetic bottleneck” in the reaction pathway to that molecule^37,43^. On the other hand, the formation energy for **B** and **C** combined with precursors from **23**–**26** was generally only weakly favoured (average formation energy −11.0 kJ mol^−1^), but yet these molecules were all formed experimentally. In this instance, these large, open molecules may be particularly stabilised, relative to their precursors, by the influence of solvent^37^.

With the exception of three of the **Tri**^**4**^**Di**^**6**^ cages (**A13**, **A14**, and **A17**, grey squares in Fig. 3b, structures in Supplementary Fig. 3), all of the hypothetical cages were predicted to be shape persistent in their target topologies. The average formation energy per imine bond for the three series of cages built from amines **A**, **B**, and **C** was predicted to be −12.5, −17.9, and −17.3 kJ mol^−1^, respectively. A priori computation suggests, therefore, that triamines **B** and **C** might be better choices for imine cage formation than triamine **A**, possibly due to the increased flexibility and lack of pre-organisation in triamine **A**^44^. This was reflected by experiment: the failure rate for triamine **A** (red, yellow, and orange squares in top row, Fig. 3c; only one system, **A11**, gave clean cage conversion) is much higher than for triamines **B** and **C**.

The **Tri**^**2**^**Di**^**3**^ cages (**1**–**10**) were found to be generally similar in energy, with the exception of cages containing aldehyde **10**, which were less favoured; we did not observe clean cage formation for **10** with any of the three triamines (Fig. 3c). **Tri**^**4**^**Tri**^**4**^ cages (**22**–**26**) were in general somewhat less energetically favoured than **Tri**^**2**^**Di**^**3**^cages (average formation energies per imine bond of −10 and −16 kJ mol^−1^, respectively), although the experimental ‘hit rate’ for these two topologies across the array was rather similar (Fig. 3c). For the **Tri**^**4**^**Di**^**6**^ (**11**–**21**) cages, the picture was more complicated with a wide variety of formation energies; for example, cages formed from aldehyde **12** were predicted to be particularly favoured, most likely due to intramolecular H-bonding^41,45^, although this did not translate into experiment (Fig. 3c).

**Fig. 4:**
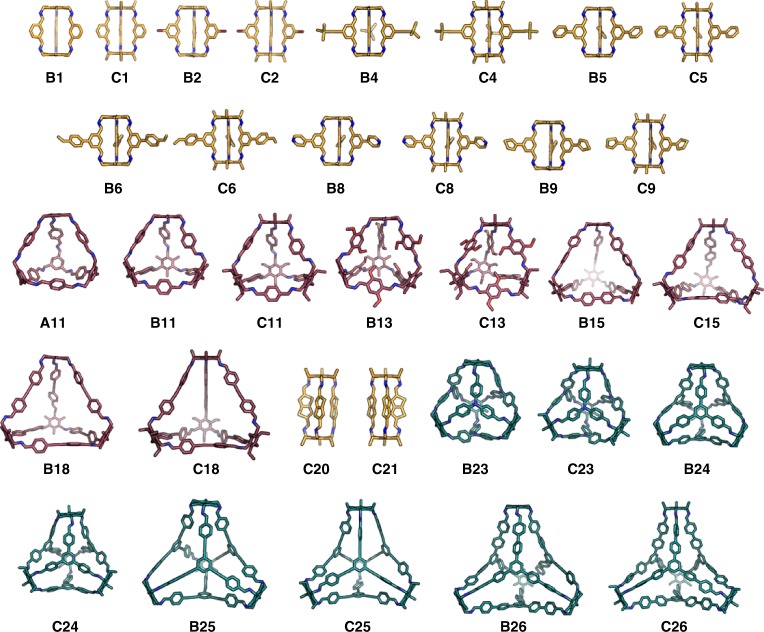
The 33 cages that formed cleanly in this high-throughput study. **Tri**^**2**^**Di**^**3**^ cages are shown with orange carbons, **Tri**^**4**^**Di**^**6**^ with maroon, and **Tri**^**4**^**Tri**^**4**^ with teal. Remaining atom colouring is as follows; oxygen (red), bromine (brown), boron (pink), silicon and sulphur (yellow), and nitrogen (blue). Hydrogens are omitted. All structures are derived from computational predictions. For cages **C20** and **C21**, **Tri**^**2**^**Di**^**3**^ cages were formed as shown, rather than the predicted **Tri**^**4**^**Di**^**6**^ cages. All other cages formed the predicted, targeted topology. Supplementary Fig. 4 shows structures for cages that were identified by solution analysis, but that were either impure or showed incomplete conversion of the starting materials. Supplementary Fig. 3 shows the computed structures for cages that did not form by experiment (red squares in Fig. 3c)

Overall, the high-throughput robotic synthesis screen (Supplementary Fig. 5, Supplementary Table 3) yielded a total of 33 cages (Fig. 4), where both ^1^H NMR spectroscopy and HRMS indicated clean cage formation without additional work up (green squares in Fig. 3c). There were a further 16 reactions (yellow/orange squares in Fig. 3c) where partial cage formation was observed (Supplementary Fig. 4). In some cases—for example, cage **C20**—the ^1^H NMR spectrum indicated clean cage formation, but there was no clear mass ion in the HRMS. For such systems, diffusion NMR was particularly useful because it provides an alternative and rapid method to determine the size (Supplementary Tables 4–9), and hence the topology, of the cages directly from the reaction mixtures^46^. We calculated the average size and maximum diameters of the cages from the energy-minimised computed conformations (Supplementary Note 5) and compared these values to the experimentally determined solvodynamic diameters (Supplementary Tables 10–11), allowing us to determine the topology of the cages. There was strong agreement between the measured size and the computed average size (Supplementary Fig. 6), which provides a rapid and direct measure of the success of our predictions without the need to obtain single crystal structures.

We next computed the cavity sizes for cages that were formed by experiment (Fig. 3d) by calculating the largest sphere that could be placed in the central molecular cavity (Supplementary Tables 1, 2, and 12). If we assume that a diameter of more than 2.89 Å (the kinetic diameter of H_2_)^47^ is required for the cages to host any guest at all, then 21 cages in this screen (27% of the 78 combinations) met this criteria. Most **Tri**^**2**^**Di**^**3**^ cages had cavities predicted to be smaller than 1.8 Å in diameter, making them too small to host guests. The **Tri**^**4**^**Di**^**6**^cages had larger cavities with diameters ranging from 2.3 to 11.2 Å, with the smaller cavities typically resulting from functionality present on the aldehyde penetrating into the internal cavity, as for **B13** and **C13** (Fig. 4). The largest predicted cavities across the library were calculated for the **Tri**^**4**^**Tri**^**4**^ cages, with **B26** having the largest cavity diameter (12.3 Å).

### Comparison of computed structures and crystal structures

For cages where we could obtain single crystal X-ray structures (Supplementary Figs. 7–27), we found an excellent geometric match with the predicted low-energy conformation obtained by a priori computation (Fig. 5a, Supplementary Table 13, and Supplementary Fig. 28), even though these calculations do not take into account crystal packing forces or solvent effects. The average root-mean-square-deviation (RMSD) between the crystal structure and the simulations was 0.53, 1.07, and 1.26 Å for the **Tri**^**2**^**Di**^**3**^**, Tri**^**4**^**Di**^**6**^, and **Tri**^**4**^**Tri**^**4**^ cages respectively. In all cases, the RMSD decreases upon geometry optimisation of a molecule extracted from the experimental crystal structure (to 0.24, 0.96, and 1.15 Å respectively), which could be indicative of crystal packing only having a minor influence on the structures.

**Fig. 5:**
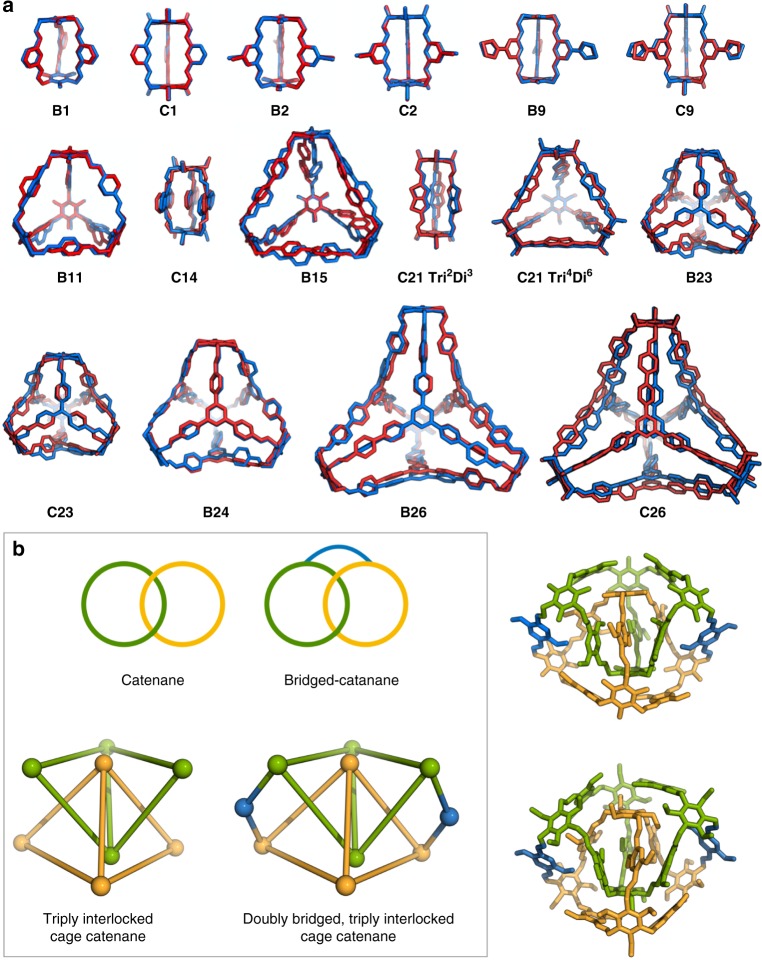
Crystal structures of cages and bridged cage catenanes. **a** Comparison of modelled and experimental cage structures—overlays of available single crystal X-ray structures for each cage (red) with the computed lowest energy conformation (blue). **b** Crystal structures of the two unexpected [8 + 12] covalently bridged, triply interlocked cage catenanes formed by re-equilibration of targeted cages, **B13** and **C13**, and comparison of the unique topology with previous catenanes, bridged catenanes, and triply interlocked cage catenanes. The [8 + 12] bridged cage catenane is triply interlocked (symmetry equivalents coloured in yellow and green), but uniquely, it has two different shaped windows and is linked by two covalent bridges (blue). Hydrogens and external functional groups have been removed for clarity. For the full crystal structures see Supplementary Figs. 12 and 22

**C21**, which formed a **Tri**^**2**^**Di**^**3**^ cage in the high-throughput synthesis screen, gave a product mixture upon scale-up and direct recrystallisation of the reaction solution: in this case, a crystal structure of both a **Tri**^**2**^**Di**^**3**^ (Supplementary Fig. 24) and a **Tri**^**4**^**Di**^**6**^ (Supplementary Fig. 25) cage was obtained. The formation of different cage topologies from the same precursors by simply changing reaction conditions has been observed before^48,49^. When the reaction was carried out in chloroform at reflux in the high-throughput screen, the **Tri**^**2**^**Di**^**3**^ cage dominated. By contrast, the scaled-up reaction in dichloromethane at reflux gave a mixture of the **Tri**^**2**^**Di**^**3**^ and **Tri**^**4**^**Di**^**6**^ cages (Supplementary Figs. 29 and 30). **C21** illustrates the potential sensitivity of the cage topology to the precise experimental conditions.

### Discovery of a unique bridged cage catenane

A unique and unpredicted cage topology was also discovered during recrystallisation studies: a covalently bridged, triply interlocked cage catenane (Fig. 5b). This topology can be formed upon crystallisation by dynamic re-equilibration of two of the **Tri**^**4**^**Di**^**6**^ cages, **B13** and **C13**. They comprise an [8 + 12] stoichiometry and are both triply interlocked (catenated) and di-covalently linked (bridged); this topology seems to be stabilised by offset π–π stacking interactions between the two sets of dimethoxy substituted aromatic groups, which are stacked <4 Å apart in the crystal structure (Supplementary Figs. 12 and 22). The formation of simpler interlocked cage catenanes has been observed before, either upon recrystallisation or by direct synthesis using an acid catalyst^50,51^. Here, we found that the targeted, non-catenated **Tri**^**4**^**Di**^**6**^ cages **B13** and **C13** re-equilibrated to form covalently bridged cage catenanes during recrystallisation. Additional simulations rationalised the formation of these unique structures (Supplementary Note 6); the bridged catenated [8 + 12] species was calculated to be more stable (>17 kJ mol^−1^ per imine bond or 215 kJ mol^−1^ per [4 + 6] unit) in comparison to the parent **Tri**^**4**^**Di**^**6**^ cage, **B13**, which was initially formed in solution, as targeted.

### Comparison of experiment with computational predictions

In the 33 cases where a single cage product was formed cleanly with good conversion, 31 cages (94%) were formed with the predicted, targeted topology. Of these, two cages (**B13** and **C13**) were found to re-equilibrate to form catenanes. Computed formation energies were found to be useful at a coarse grain level—for example, in suggesting correctly that reactions with triamine **A** were less promising than for triamines **B** and **C**—but they are not an unambiguous predictor of whether or not a given cage can be synthesised. For example, cages with precursor **10** were found to have the *least* favourable formation energies, with relatively high strain around the imine bonds, and yet **A10**, **B10**, and **C10** were all formed by experiment, albeit with residual aldehyde starting material and potential oligomeric side products. Cages comprising aldehyde **12** were found to have particularly favourable formation energies, yet **B12** was not formed under these reaction conditions and both **A12** and **C12** were formed (in impure form) in a **Tri**^**2**^**Di**^**3**^ topology rather than **Tri**^**4**^**Di**^**6**^.

Some precursor combinations went against our targeted topology assumptions and formed an alternative topology. All of these examples were found in the **Tri**^**4**^**Di**^**6**^ series; where **Tri**^**2**^**Di**^**3**^ and **Tri**^**4**^**Tri**^**4**^ topologies were targeted, all cages gave the predicted topology. For **A12**, **C12**, **C14**, **B20**, **C20**, **B21**, and **C21**, a **Tri**^**2**^**Di**^**3**^ cage was observed instead of the targeted **Tri**^**4**^**Di**^**6**^; **B14** formed a mixture of both of these topologies (Supplementary Fig. 31). Thus, of the 33 reactions predicted to form **Tri**^**4**^**Di**^**6**^ cages, only 9 formed the predicted **Tri**^**4**^**Di**^**6**^; 7 formed **Tri**^**2**^**Di**^**3**^, 16 did not form a cage, and 1 formed a **Tri**^**2**^**Di**^**3**^/**Tri**^**4**^**Di**^**6**^ mixture. The energetic preference for the **Tri**^**4**^**Di**^**6**^ topology in the example system, **B11**, was the smallest of the three systems investigated; 29 kJ mol^−1^ per [2 + 3] unit, compared to 43 kJ mol^−1^ per [2 + 3] unit, and 118 kJ mol^−1^ per [1 + 1] unit for **Tri**^**2**^**Di**^**3**^ and **Tri**^**4**^**Tri**^4^ topologies, respectively. This may rationalise the poor predictability for the **Tri**^**4**^**Di**^**6**^ systems; the lack of a strong driving force for a particular topology might also help to explain why so many of the **Tri**^**4**^**Di**^6^ targeted reactions failed to produce any cage product at all (red squares, Fig. 3c, middle panel).

To try to explain the unexpected **Tri**^**2**^**Di**^**3**^ topologies (**A12**, **C12**, **B14**, **C14**, **B20**, **C20**, **B21**, and **C21**), the energies for these outcomes were calculated and compared to the targeted **Tri**^**4**^**Di**^**6**^ topology (Supplementary Tables 12 and 14, and Supplementary Fig. 31). For **B21** and **C21**, a preference was found for the observed **Tri**^**2**^**Di**^3^ topology (by 24 and 35 kJ mol^−1^ per [2 + 3] unit, respectively); in all the remaining cases, an energetic preference was calculated for the **Tri**^**4**^**Di**^**6**^ topology rather than the observed **Tri**^**2**^**Di**^**3**^ topology. This preference was often small: around 5 kJ mol^−1^ per [2 + 3] unit for **A12** and **C20**. We cannot therefore rationalise the observed topologies for **A12**, **C12**, **B14**, **C14**, **B20** and **C20** based upon the relative internal energies of the isolated cage molecules alone. It is possible that the entropy of the systems, solvent interactions, the stability and solubility of reaction intermediates or competing reaction pathways play a decisive role. Taken together, our results suggest that an energetic preference of at least 30 kJ mol^−1^ per repeat unit is required to be confident of a reliable computational prediction of a particular cage topology.

### Integrated experimental–computational workflow

**Fig. 6:**
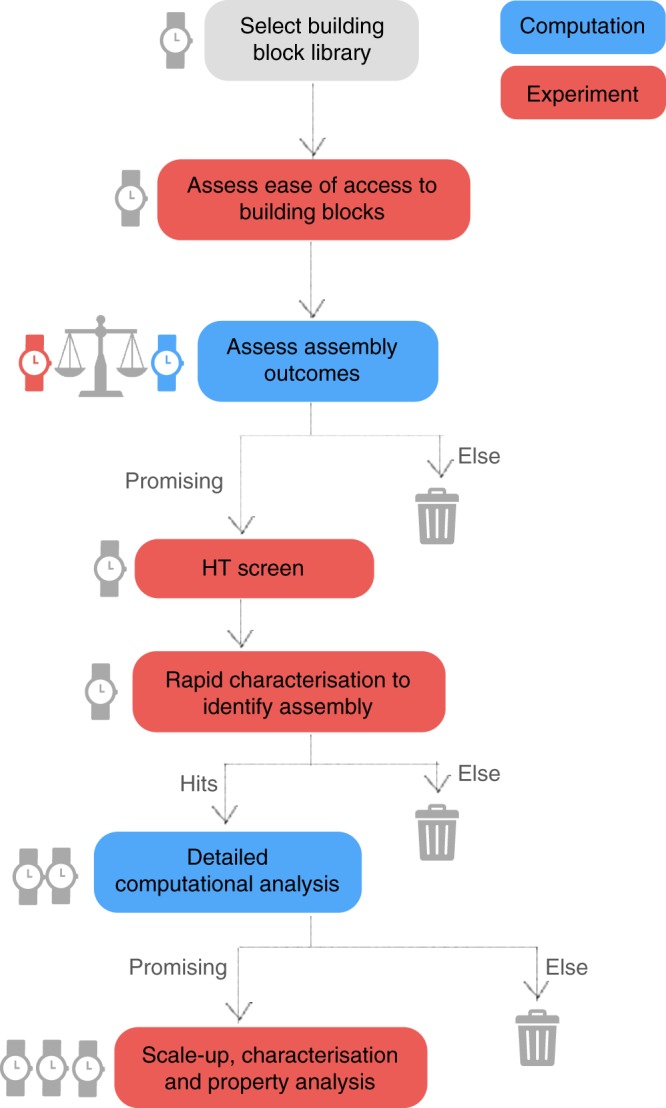
Proposed workflow for the fusion of high-throughput automated synthesis with computation for the accelerated discovery of supramolecular materials. Experimental stages are shown in red and computational stages in blue. Approximate time cost for each step is indicated (1 clock = quick; 3 clocks = time-intensive). In this first study, all building block combinations were investigated to validate the approach, but the future goal is to remove low-value targets as early as possible, thus saving time and cost. First, an initial building block library is selected, and their ease of synthesis is assessed. Then, a judgement is made on a sliding scale as to whether a very rapid computational assessment of properties is conducted (e.g., test for shape-persistence in assumed topology), up to a full analysis of multiple assembly outcomes, detailed energetic assessment of the likely outcome and full property assessment. The basis for this judgement revolves around the synthetic effort in obtaining a given building block. For building blocks that are commercially available at low cost, there is less benefit for full computational assessment of the outcomes, but for building blocks that require custom, multi-step synthesis, there is great value in only embarking on synthesis if computation suggests there is a strong likelihood of forming supramolecular products of interest. After the synthetic screen, rapid assessment of the reaction outcomes (e.g., via mass spectrometry and diffusion NMR) are used to identify where ‘hits’ have occurred and what the reaction outcome is. For ‘hits’, more detailed computational analysis is performed and only those that are promising (e.g., possessing shape persistent voids for porous solids) are selected for scale-up and property measurements

While previous studies have compared computation and experiment for a few closely related cage molecules^36,37,52,53^, this new study is unique because we make this comparison across a broad array of 78 supramolecular systems. Based on the lessons learned, we propose a more generalised workflow for supramolecular materials discovery that couples automated synthesis closely with computational studies (Fig. 6). One goal of this hybrid approach is to remove as many inferior targets as early as possible, to save time and cost. By applying computation at the appropriate stages, we can greatly streamline the materials discovery process and target more effective reaction combinations: for example, removal of the ‘unreliable’ triamine **A** from the array based on formation energy calculations (Fig. 3b) would have reduced the number of reactions by one third while missing only one of the 33 reactions (**A11**) that were found by experiment to give clean cage conversion (Fig. 3c). Likewise, the high success rate for the target **Tri**^**4**^**Tri**^**4**^ topologies (80% if one excludes triamine **A**; Fig. 3c) was anticipated by our relative energy calculations that showed a more pronounced energy penalty for alternative cage topologies (Fig. 2b).

### Discussion

In conclusion, the combination of computational modelling with robotic automation and high-throughput characterisation techniques has led to the discovery of a range of new organic cages. By investigating 78 building block combinations, 33 cages, 32 of which are new, were synthesised cleanly on a robot synthesiser in one-pot reactions without work up, with 16 other combinations showing evidence of cage formation along with side products or unreacted starting material. The scale-up of these experimental ‘hits’ was investigated, and crystallisation studies were conducted; this yielded 18 single crystal structures and led to the serendipitous discovery of two covalently bridged cage catenanes.

From a computational perspective, it is beneficial to have a strong energetic preference towards a topological outcome, as this increases the chance of the target cage being formed. This study shows how smaller energetic preferences can be overridden by other experimental factors, such as solvent stabilisation. Our rule of thumb is that a computed energetic preference of at least 30 kJ mol^−1^ per repeat unit is required for confident predictive design.

False negatives are also a possibility in high-throughput screening: for example, in the case of **C22** there was no indication of the targeted **Tri**^**4**^**Tri**^4^ cage in the ^1^H NMR spectrum arising from the robotic screen. Coupled with the formation of an insoluble precipitate, this suggested the formation of oligomers or polymer. However, Mastalerz et al. recently reported the successful synthesis of this cage under different reaction conditions, albeit in only 27% yield and with a considerable amount of polymer formation too (68%)^44^. In the future, this might be addressed by using higher capacity synthesis robots and by investigating a broader range of reaction solvents.

Such high-throughput studies covering a broad range of conditions stand to benefit particularly from computational guidance; for example, by focussing the range of precursors that is investigated. Coupled over many cycles, this hybrid discovery approach promises to yield a large database of successful and failed reactions, along with associated computational properties, that should fuel machine learning approaches in the future^54^. Moreover, this methodology could be combined with fast crystal structure prediction methods to predict the likely packing motifs for new cages^27,55^, thus allowing us to weight the selection of precursors toward systems that are likely to deliver materials with specific solid-state properties, such as adsorption selectivity for a particular gas. Furthermore, this integrated experimental–computational workflow could be applicable to other supramolecular assemblies, such as metal-organic cages, and is not limited to reversible imine chemistries.

Applications of these new cages are currently under investigation: for example, after purification some of the hits from this study demonstrate gas uptakes that are comparable to other porous cages in the literature; **B11** and **B23** demonstrate H_2_ uptakes of 2.31 and 3.15 mmol g^−1^ (77 K, 1 bar), respectively (Supplementary Figs. 32–34). We are also considering some of these molecules as tectons in other supramolecular assemblies, such as hydrogen-bonded organic frameworks or other extended crystalline frameworks^56^.

## Methods

### General synthetic and analytical methods

See Supplementary Methods for further details.

### Procedures for the synthesis of precursors

The synthesis and characterisation of triamines **A**, **B**, and **C**, is described in the Supplementary Methods. Aldehydes **1**, **3**, **10**, **11**, **13**–**15**, and **20**–**23** were commercially available, and the synthesis and characterisation of aldehydes **2**, **4**–**9**, **12**, **16**–**19**, and **24**–**26** is included in the Supplementary Methods. For spectra of novel precursors see Supplementary Figs. 35–42.

### Optimisation of model cage reactions

Terephthalaldehyde **11** (9.7 mg, 0.072 mmol, 3.0 eq.) and (2,4,6-trimethylbenzene-1,3,5-triyl)trimethanamine **B** (2.0–3.0 eq.) were dissolved in CDCl_3_ (1–13 mL) and stirred at room temperature or 60 °C for 1–6 days (see Supplementary Table 15). The formation of insoluble precipitate was monitored, alongside reaction progress by ^1^H NMR spectroscopy, to determine the optimal cage formation conditions (see Supplementary Fig. 43). The optimised conditions were then investigated for scalability with the **Tri**^**4**^**Di**^**6**^ cage **B11**, before confirming that the conditions were translatable to both a **Tri**^**2**^**Di**^**3**^ (**B1**), and **Tri**^**4**^**Tri**^**4**^ (**B23**) topology—see Supplementary Methods for the synthesis and characterisation details, and Supplementary Figs. 44–49 for the corresponding spectra.

### High-throughput synthetic screen general method

Synthetic screening was carried out on a Chemspeed Accelerator SLT-100 synthesiser platform by liquid dispensing stock solutions of the precursors (2.5–5 mg mL^−1^, Supplementary Tables 16–19) into jacketed reactor vessels and heating for 3 days at 65 °C with vortexing (800 rpm)—for ease of analysis the reactions were carried out in deuterated chloroform. The reactions were analysed directly, prior to isolation, using high-throughput techniques including ^1^H NMR spectroscopy and high-resolution mass spectrometry (HRMS) –see Supplementary Figs. 50–72. For each targeted topology (**Tri**^**2**^**Di**^**3**^, **Tri**^**4**^**Di**^**6**^, and **Tri**^**4**^**Tri**^**4**^), a number of precursor combinations, which afforded clean cage formation, were selected, and diffusion NMR conducted, allowing the stoichiometry and sizes of the formed cages to be determined. For combinations that indicated cage formation, the reaction solutions were then filtered, where necessary, to remove any insoluble precipitate (see Supplementary Table 20), and the solvent removed under reduced pressure in a Combidancer evaporator. The resulting isolated solids were analysed by powder X-ray diffraction (PXRD); all samples were amorphous on direct isolation from the reaction solvent (Supplementary Figs. 73–76). The stability to isolation was also investigated by re-dissolving and re-analysing the samples by ^1^H NMR spectroscopy—in some instances, the solid either did not fully re-dissolve suggesting some instability to isolation, or decomposition was visible after comparing the pre-isolation and post-isolation spectra—for a representative worked example of the high-throughput characterisation see Supplementary Note 7, and for full details and a summary of the results see Supplementary Methods  and Supplementary Tables 21–26).

### Representative procedure for the scale-up of the new cage series, as exemplified by B11

For ease of isolation, the solvent used in the scale-up reactions was switched from chloroform to DCM where possible, as this allowed a simpler solvent exchange to hexane with the resulting precipitated cages collected by filtration. A solution of terephthalaldehyde **11** (194 mg, 1.45 mmol, 6.0 eq.) and (2,4,6-trimethylbenzene-1,3,5-triyl)trimethanamine **B** (250 mg, 1.21 mmol, 5.0 eq.) dissolved in DCM (260 mL) was heated at 40 °C for 2 days. The reaction was allowed to cool to room temperature and the mixture filtered to remove any insoluble precipitate before the addition of hexane (200 mL). The DCM was carefully removed in vacuo to give a colourless precipitate which was collected by filtration and dried in vacuo to yield pure cage product **B11** (224 mg, 0.16 mmol, 65%). **IR** (*ν*_max_ (cm^−1^)) 3391 (br), 2868, 1636, 1566, 1449, 1374, 1315, 1218; ^**1**^**H NMR** (500 MHz, CDCl_3_) δ_H_ 8.30 (t, *J* = 1.6 Hz, 12H), 7.75 (s, 24H), 4.93 (s, 24H), 2.37 (s, 36H); ^**13**^**C NMR** (101 MHz, CDCl_3_) δ_C_ 160.4, 138.3, 137.7, 133.2, 128.5, 59.2, 16.4; **HRMS** (ES+) calc. for **Tri**^**4**^**Di**^**6**^ cage C_96_H_96_N_12_ 1417.7912; found [M + H]^+^ 1418.8056, [M + 2 H]^2+^ 709.9087 and [M + H + Na]^2+^ 720.8998.

The synthesis and characterisation data of the other cage hits are described in the Supplementary Methods—for the corresponding spectra, see Supplementary Figs. 77–114. See Supplementary Methods for crystal structure refinement details and notes for those cages and catenanes where single crystal X-ray structures could be obtained.

### Computational modelling

To determine the intrinsic structural preference for a topology in the representative set of three systems (**B1**, **B11**, **B23**), models of the most likely topologies were assembled: for **B1** and **B11**—**Tri**^**2**^**Di**^**3**^, **Tri**^**4**^**Di**^**6**^, **Tri**^**6**^**Di**^**9**^ and **Tri**^**8**^**Di**^**12**^, and for **B23**—**Tri**^**1**^**Tri**^**1**^, **Tri**$$\begin{array}{*{20}{c}} 2 \\ 2 \end{array}$$**Tri**^**2**^ and **Tri**^**4**^**Tri**^**4**^. Conformer searches were carried out for each structure using high-temperature MD simulations, followed by geometry optimisation, all with OPLS3^42^. Low-energy structures were then geometry optimised with DFT calculations at the PBE+D3/TZVP level^57,58^ and relative energies compared. The same computational approach was used to find the low-energy conformations for the complete set of high-throughput cages, assuming their targeted topology was realised. Precursor 7 was not included and precursor **10** was simplified by removing alkyl groups. The void diameters of cages were calculated by consideration of the distance between the centre of mass of a cage and the edge of the closest atom; the maximum cage diameter as the largest distance between the edges of any two atoms. The calculation of the average molecular diameter is described in the supporting information. For the set of molecules that unexpectedly formed **Tri**^**2**^**Di**^**3**^ rather than the targeted **Tri**^**4**^**Di**^**6**^ topology, the **Tri**^**2**^**Di**^**3**^ topology was modelled to compare the relative energies of the two outcomes. To compare the relative energy of the bridged catenated structure to the related **Tri**^**4**^**Di**^**6**^ topology, the structure of a single [8 + 12] molecule was taken from the SC-XRD structure and geometry optimised at the DFT level.

### Data availability

Supplementary crystallographic files, which include structure factors, have been deposited with the Cambridge Crystallographic Data Centre (CCDC) as deposition numbers 1827867–1827887. These data files can be obtained free of charge from http://www.ccdc.cam.ac.uk/data_request/cif.

All relevant data including full synthetic, characterisation, crystallographic, and computational details are available in the Supplementary Information. Structures for all the low-energy computed cages are also supplied as Supplementary Data 1.

## Electronic supplementary material

Supplementary Information

Description of Additional Supplementary Files

Supplementary Data 1
