# Supplementary material for: High-throughput discovery of organic cages and catenanes using computational screening fused with robotic synthesis
Source: Nat Commun. 2018 Jul 20;9:2849. doi: 10.1038/s41467-018-05271-9 (PMC6054661; doi:10.1038/s41467-018-05271-9)
Supplement: Supplementary file 1 — Supplementary Information [file 41467_2018_5271_MOESM1_ESM.pdf]

Supplementary Information

High-throughput discovery of organic cages and catenanes using computational screening fused with robotic synthesis

R. L. Greenaway *et al.*

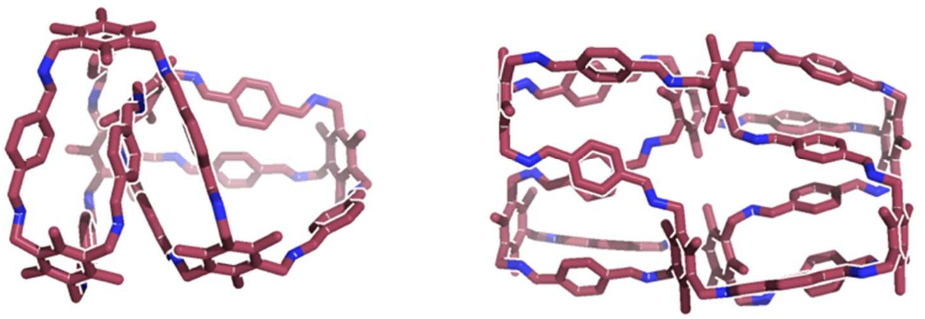

Supplementary Figure 1: The lowest energy conformations of the larger topologies of **B11**, which are collapsed; (left) **Tri^{6}Di^{9}** and (right) **Tri^{8}Di^{12}**.

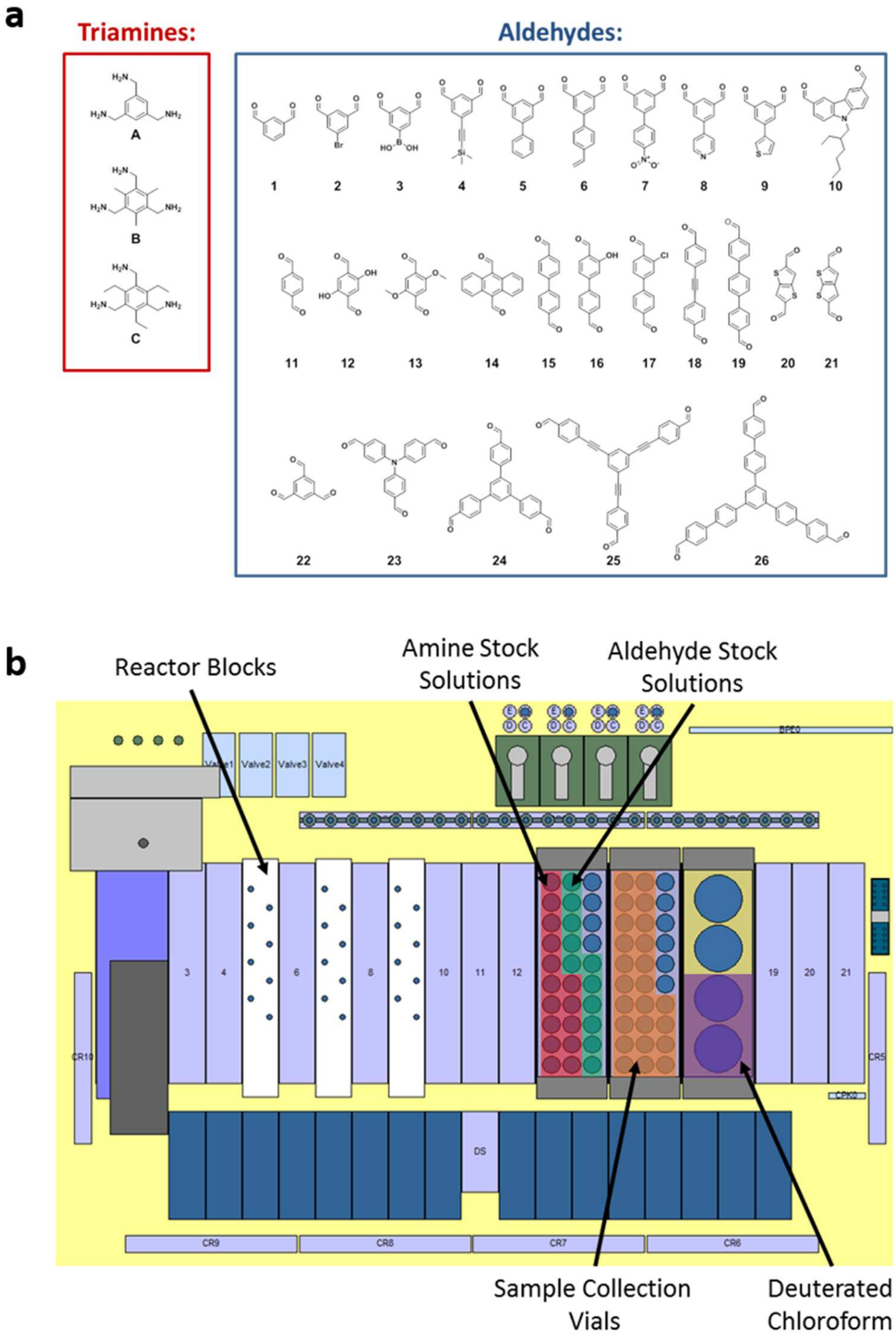

Supplementary Figure 2: High-throughput screening. (a) Three triamines (**A-C**) and 26 aldehydes (**1-26**) were selected leading to a total of 78 combinations for the high-throughput cage screen. Cage naming is based on which 2 components were used in the cage formation, for example, the reaction between triamine **B** with aldehyde **12** leads to cage **B12**. **(b)** General Chemspeed Accelerator SLT-100 deck layout used for the high-throughput synthetic screen. Reactors blocks on the left contained the cage forming reactions after addition of the stock solutions of the triamines and aldehydes, and top-up solvent (deuterated chloroform), by liquid dispensing from the right.

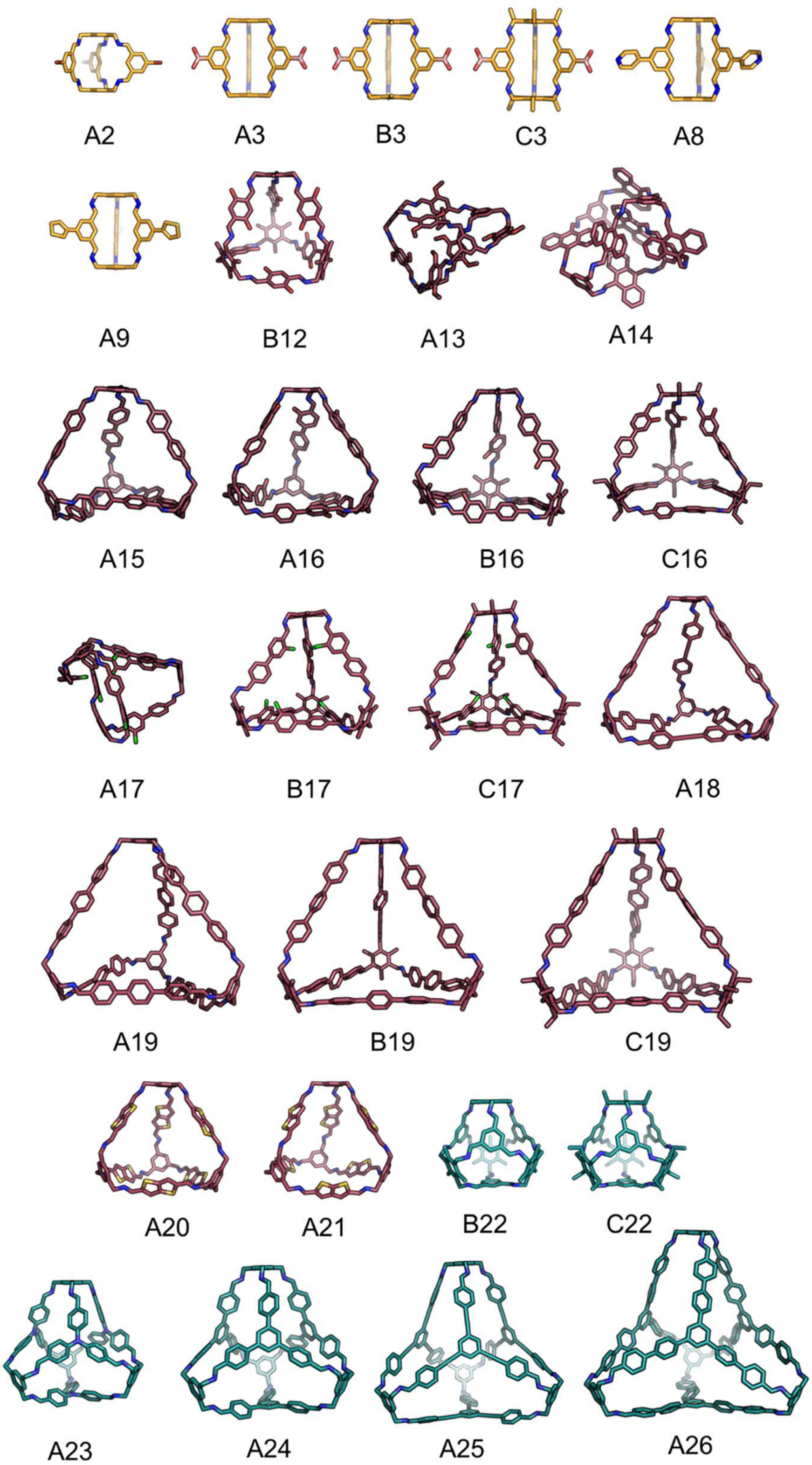

Supplementary Figure 3: The 29 cages that did not form in this study, shown in their targeted topology. The cage **A7** is not shown, as it was not modelled. Cages **A13**, **A14** and **A17** were predicted to not be shape-persistent. **Tri^{2}Di^3** cages are shown with orange carbons, **Tri^{4}Di^6** in maroon and **Tri^{4}Tri^4** in teal. Remaining atom colouring is as follows; oxygen (red), bromine (brown), boron (pink), silicon and sulfur (yellow), chlorine (green) and nitrogen (blue). Hydrogens are omitted.

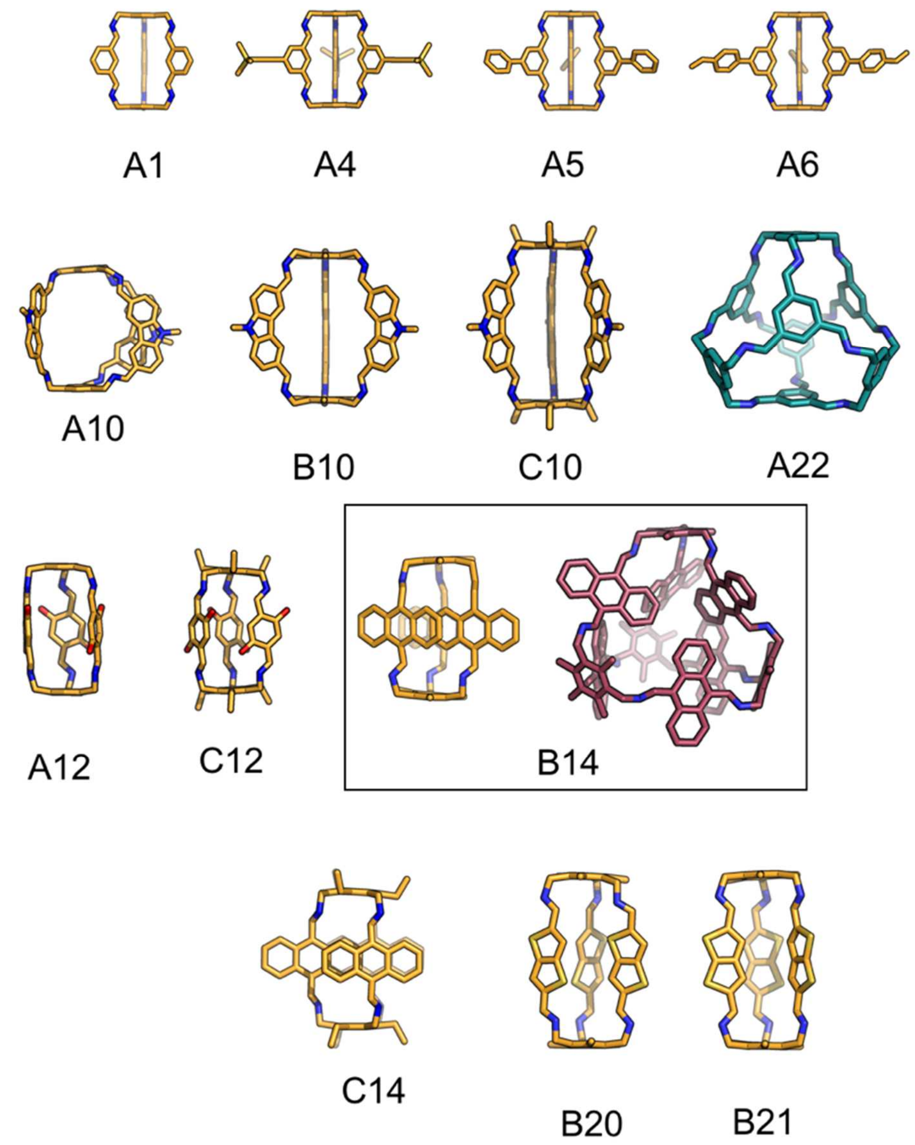

Supplementary Figure 4: The 16 cages that formed in this study, but for which the mixture was impure or there was incomplete conversion of starting material. The two cages **B7** and **C7** with precursor **7** are not shown, as these were not modelled. **Tri^{2}Di^3** cages are shown with orange carbons, **Tri^{4}Di^6** in maroon and **Tri^{4}Tri^4** in teal. Remaining atom colouring is as follows; oxygen (red), bromine (brown), boron (pink), silicon and sulfur (yellow), and nitrogen (blue). Cages synthesised with **10** are simplified with the external alkyl groups replaced with a methyl group. Hydrogens are omitted.

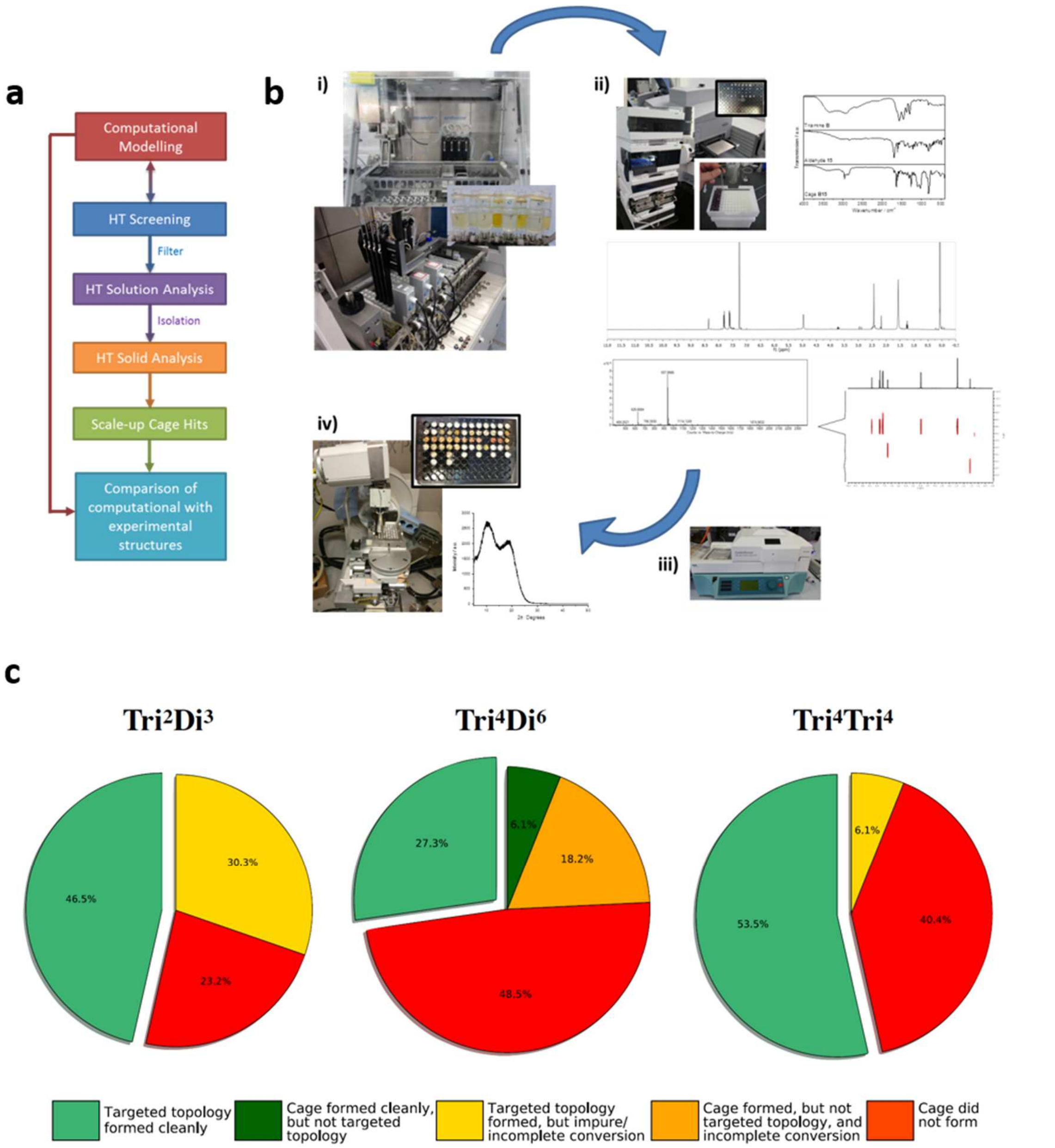

Supplementary Figure 5: Summary of the overall high-throughput (HT) workflow for the discovery, synthesis and characterisation of organic cages. (a) Overall workflow used for the design, synthesis, and characterisation of organic cages; **(b)** High-throughput synthetic workflow: i) The 78 possible combinations for cage formation were screened on a Chemspeed Accelerator SLT-100 robotic platform using liquid dispensing with the reactions heated for 3 days at 65 °C with vortexing at 800 rpm; ii) Aliquots of all 78 reactions were taken directly from the high-throughput screen (pre-isolation) and analysed by ^{1}H NMR spectroscopy, high-resolution mass-spectrometry (HRMS), and FTIR using a thin film deposited on a 96-well silica wafer. For a selection of reactions, blind diffusion NMR was carried out where clear cage formation was visible. This allowed the stoichiometry and size of the formed cages to be determined; iii) Combinations which indicated cage formation were filtered to remove any insoluble precipitate, and isolated by removal of the solvent under reduced pressure on a Combidancer evaporator; iv) For the isolated solid samples, the sample's crystallinity was investigated by powder X-ray diffraction (PXRD), and they were re-analysed by ^{1}H NMR spectroscopy to determine stability to isolation; **(c)** Visual comparison of results from the high-throughput screen.

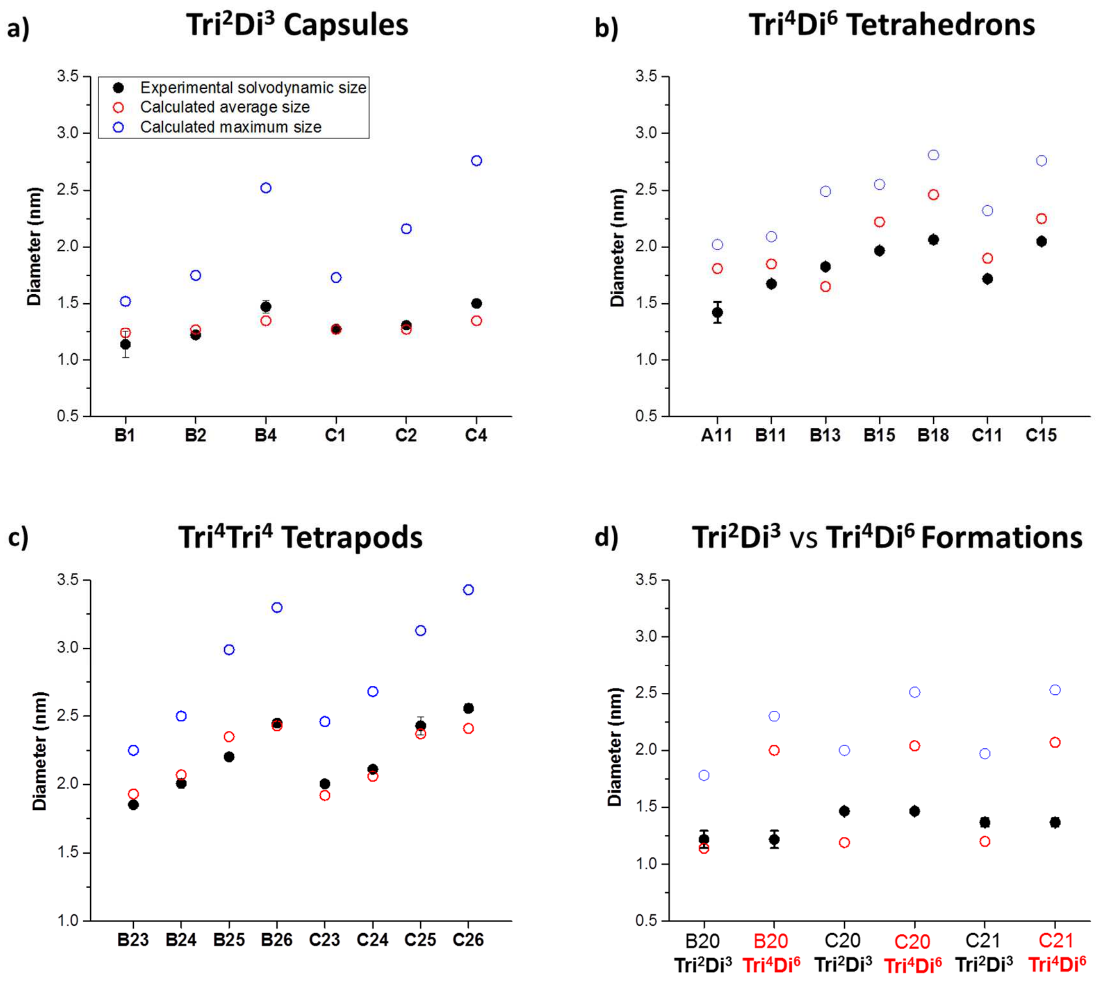

Supplementary Figure 6: Comparison of solvodynamic diameters from diffusion NMR measurements with cage sizes calculated from predicted cage conformers. Diffusion NMR measurements (black filled circles) are compared to the simulated average cage sizes (red circles) and the maximum cage diameters (blue circles) of the molecules; for structures of the cages, see **Fig. 4** and **Supplementary Fig. 4**. (a) **Tri^{2}Di^3** cages; (b) **Tri^{4}Di^6** cages; (c) **Tri^{4}Tri^4** cages; (d) Cages that deviated from the predicted/targeted topology. The solvodynamic diameters obtained by diffusion NMR were generally in good agreement with the average diameters for the computationally-predicted cages, particularly for the **Tri^{2}Di^3** (a) and **Tri^{4}Tri^4** cages (c). The good correlation between the measured solvodynamic diameters and the average predicted diameters allowed us to determine the size of cages where mass spectrometry failed, such as **A11**, **B20**, **C20**, **B24**, **C24**, **B25**, **B26** and **C26**. Diffusion NMR of **B20** and **C20** indicated the formation of a **Tri^{2}Di^3** topology rather than the targeted **Tri^{4}Di^6** cage (d). To further validate this method, the size of **C21** was also measured by diffusion NMR, with the prior knowledge from HRMS that a **Tri^{2}Di^3** cage had formed instead of the targeted **Tri^{4}Di^6** analogue – (d) shows that **C21** has a similar solvodynamic diameter to both **B20** and **C20**, confirming its **Tri^{2}Di^3** topology.

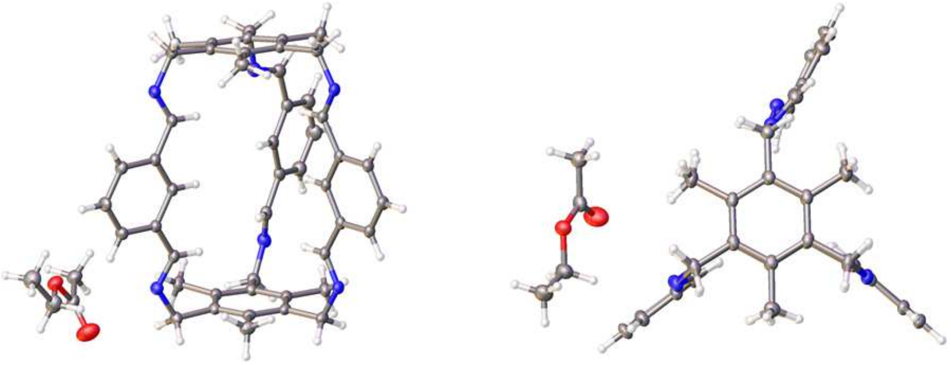

Supplementary Figure 7: Displacement ellipsoid plots from the single crystal structure, **B1**·0.82($C_4H_8O_2$)·0.18(CH_2Cl_2)·0.2(H_2O); ellipsoids are displayed at 50% probability level; two views are shown. Disordered CH_2Cl_2 and H_2O has been omitted for clarity. The general colouring scheme for the displacement ellipsoid plots are as follows; C = grey; H = white; N = blue; O = red; Br = brown; Cl = green; and S = yellow.

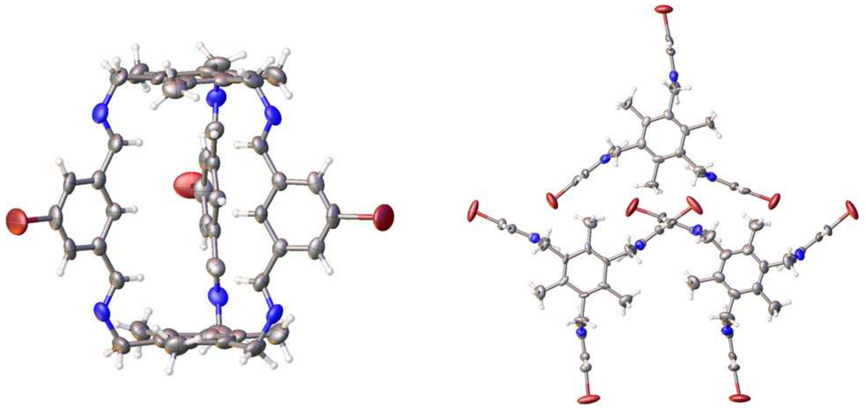

Supplementary Figure 8: Displacement ellipsoid plots from the single crystal structure, 3(**B2**)·6(CH_2Cl_2); shown for one **B2** cage in entirety (left); and for the three crystallographically distinct cages shown in entirety (right). Ellipsoids are displayed at 50% probability level and CH_2Cl_2 solvent has been omitted for clarity. The general colouring scheme for the displacement ellipsoid plots are as follows; C = grey; H = white; N = blue; O = red; Br = brown; Cl = green; and S = yellow.

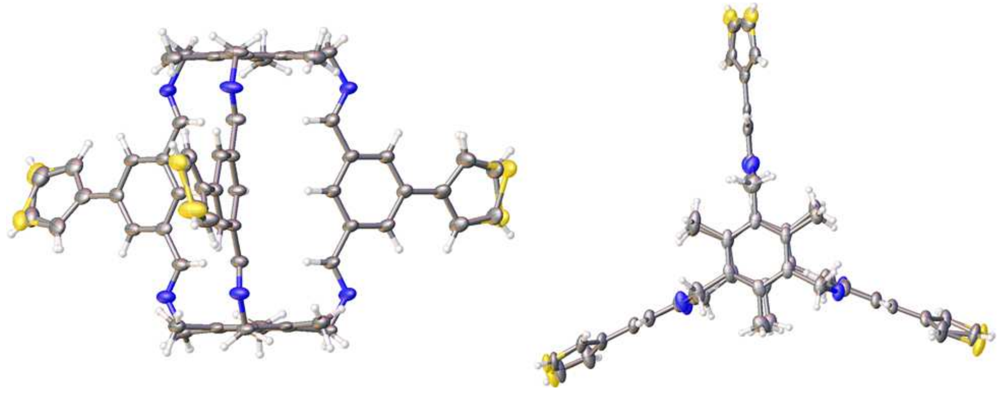

Supplementary Figure 9: Displacement ellipsoid plots from the single crystal structure, **B9**·0.75($CDCl_3$)·1.99(H_2O); two views shown; ellipsoids are displayed at 50% probability level. The disordered solvent has been omitted for clarity. The general colouring scheme for the displacement ellipsoid plots are as follows; C = grey; H = white; N = blue; O = red; Br = brown; Cl = green; and S = yellow.

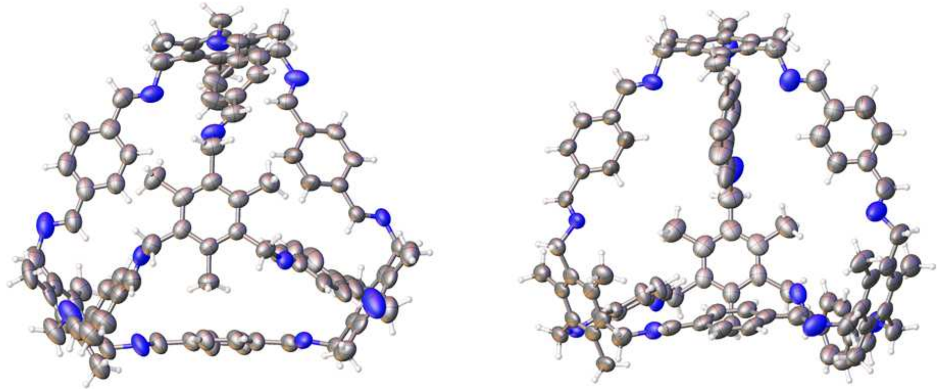

Supplementary Figure 10: Displacement ellipsoid plots from the single crystal structure, **B11**·4.5(C_6H_{14})·4.5(CH_2Cl_2); ellipsoids are displayed at 50% probability level; two views shown. The general colouring scheme for the displacement ellipsoid plots are as follows; C = grey; H = white; N = blue; O = red; Br = brown; Cl = green; and S = yellow.

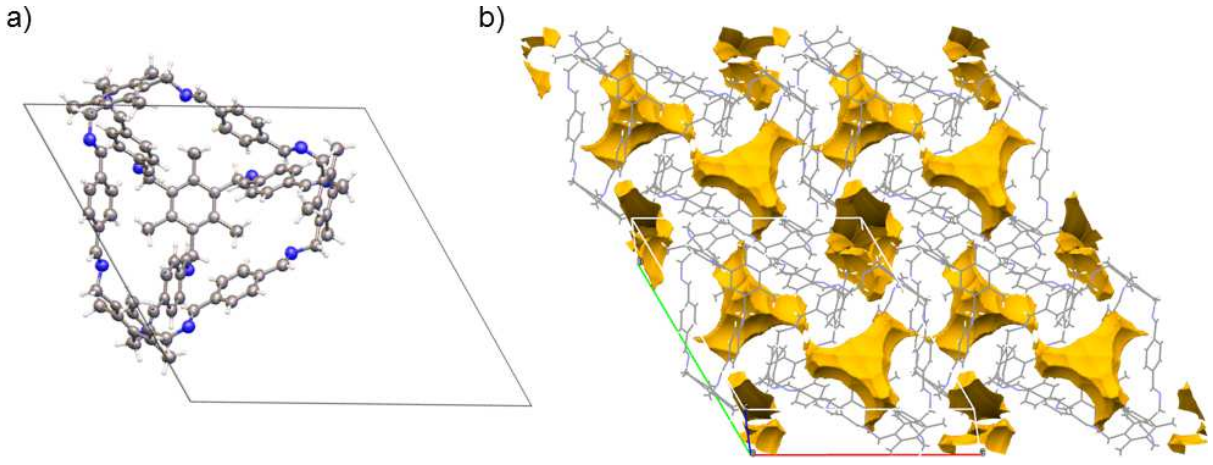

Supplementary Figure 11: (a) Displacement ellipsoid plot from the desolvated single crystal structure of **B11** (space group symmetry, $P\overline{3}$) showing **B11** cage in entirety; perspective view [001]; ellipsoids are displayed at 50% probability level. (b) Crystal packing from the desolvated crystal structure of **B11**, voids are shown in yellow (probe radius 1.2 Å, grid spacing 0.15 Å). The general colouring scheme for the displacement ellipsoid plots are as follows; C = grey; H = white; N = blue; O = red; Br = brown; Cl = green; and S = yellow.

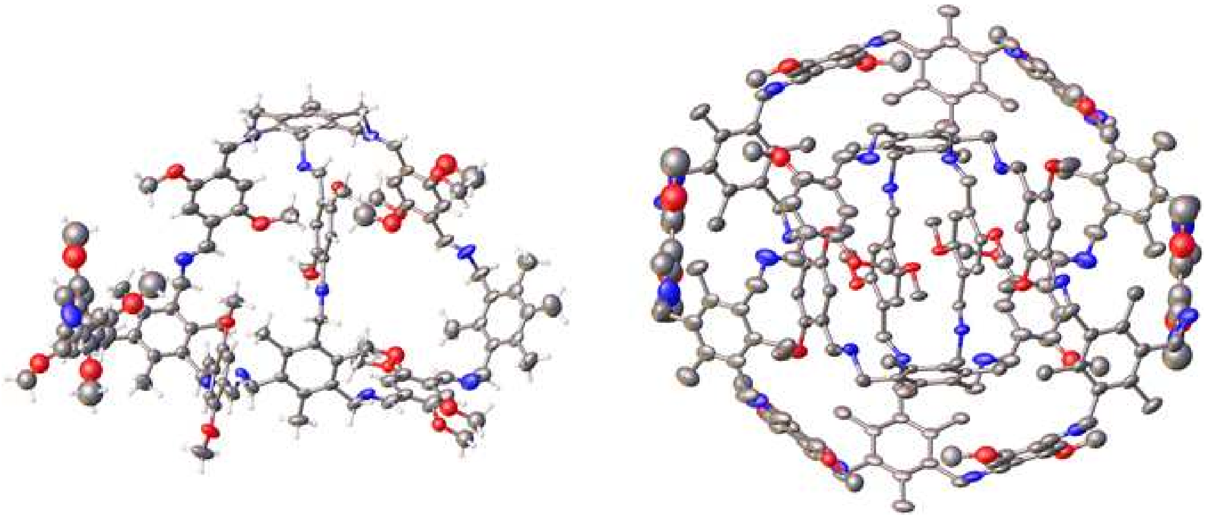

Supplementary Figure 12: Displacement ellipsoid plot of the asymmetric unit from the solvated single crystal structures, **B13**$_{[8+12]}$·2($CHCl_3$)·2(THF)·18.5(H_2O) (left); isotropically refined atoms shown as spheres; ellipsoids displayed at 20% probability. Displacement ellipsoid plot of the complete **B13**$_{[8+12]}$ structure (right); ellipsoids displayed at 20% probability level; hydrogens, solvent, and disorder are omitted for clarity. The general colouring scheme for the displacement ellipsoid plots are as follows; C = grey; H = white; N = blue; O = red; Br = brown; Cl = green; and S = yellow.

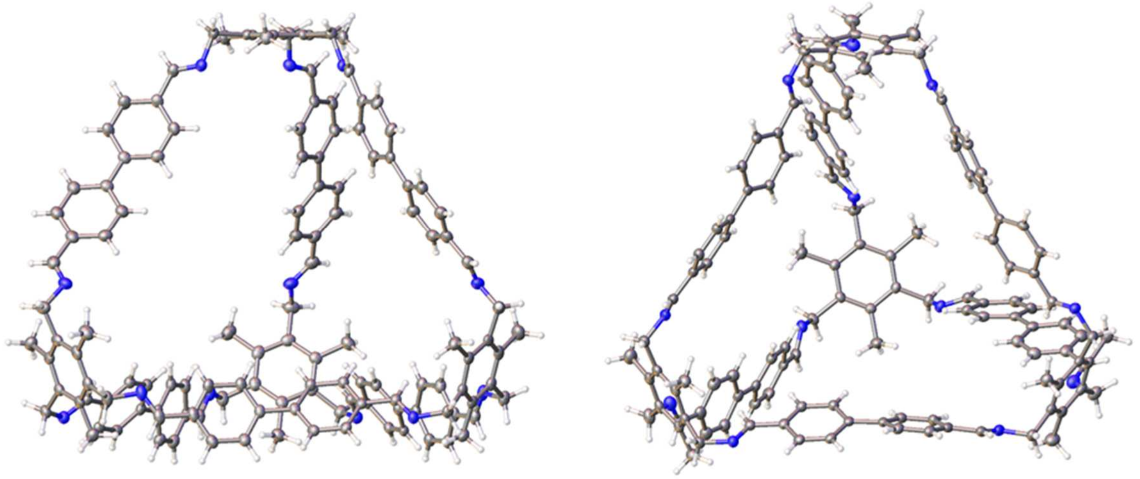

Supplementary Figure 13: Displacement ellipsoid plots from the single crystal structure, **B15**·1.5(C_6H_{14})·5.5($CHCl_3$)·3(H_2O); **B15** shown in entirety; perspective [100] (left) right [001] (right). Ellipsoids are displayed at 50% probability level and solvent molecules have been omitted for clarity. The general colouring scheme for the displacement ellipsoid plots are as follows; C = grey; H = white; N = blue; O = red; Br = brown; Cl = green; and S = yellow.

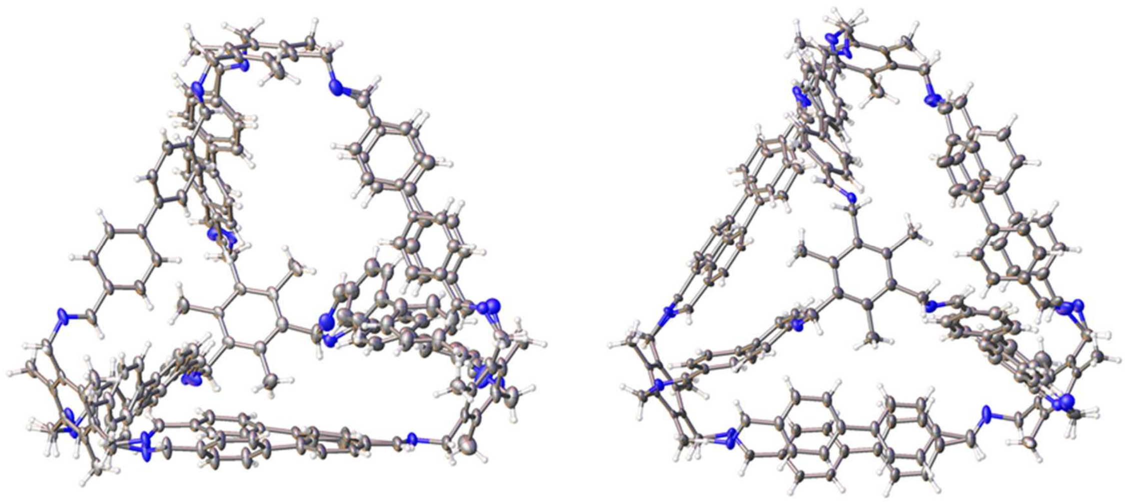

Supplementary Figure 14: Displacement ellipsoid plot of the asymmetric unit from the single crystal structure, **B15**·2.25(C_6H_{14})2.25(CH_2Cl_2); two views shown. Ellipsoids are displayed at 30% probability level and solvent molecules have been omitted for clarity. The general colouring scheme for the displacement ellipsoid plots are as follows; C = grey; H = white; N = blue; O = red; Br = brown; Cl = green; and S = yellow.

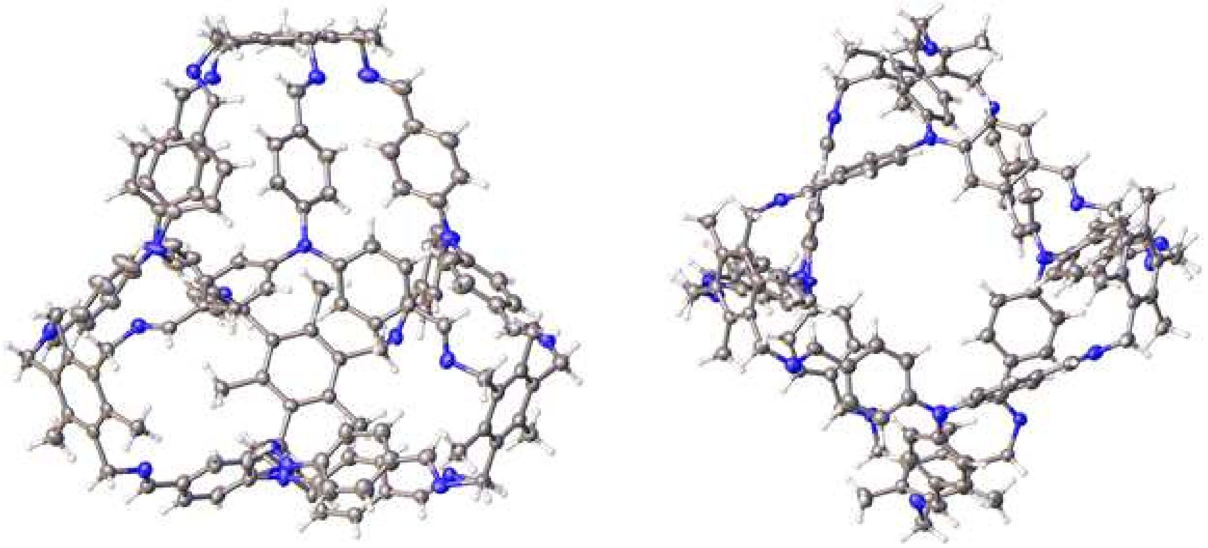

Supplementary Figure 15: Displacement ellipsoid plot of the asymmetric unit from the single crystal structure, **B23**·7.75(CH_2Cl_2)·4.5(C_6H_{14})·0.5(H_2O); two views shown. Ellipsoids are displayed at 50% probability level and solvent molecules have been omitted for clarity. The general colouring scheme for the displacement ellipsoid plots are as follows; C = grey; H = white; N = blue; O = red; Br = brown; Cl = green; and S = yellow.

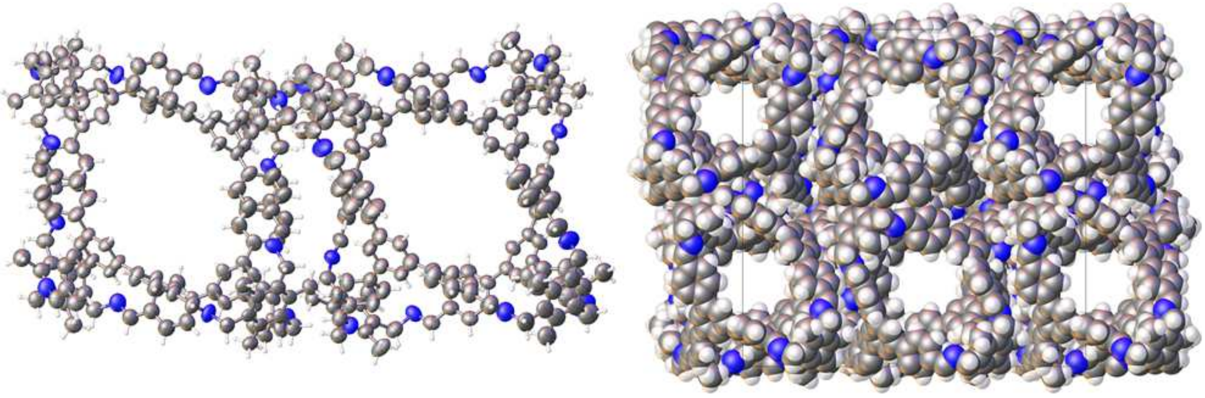

Supplementary Figure 16: Displacement ellipsoid plot from the single crystal structure, **B24**·13.35($C_4H_8O_2$)·11.75(CH_2Cl_2) (left); ellipsoids are shown at 50% probability level; solvent omitted for clarity. Space filling representation of the crystal packing from the crystal structure (right); perspective view [100]; unit cell shown. The general colouring scheme for the displacement ellipsoid plots are as follows; C = grey; H = white; N = blue; O = red; Br = brown; Cl = green; and S = yellow.

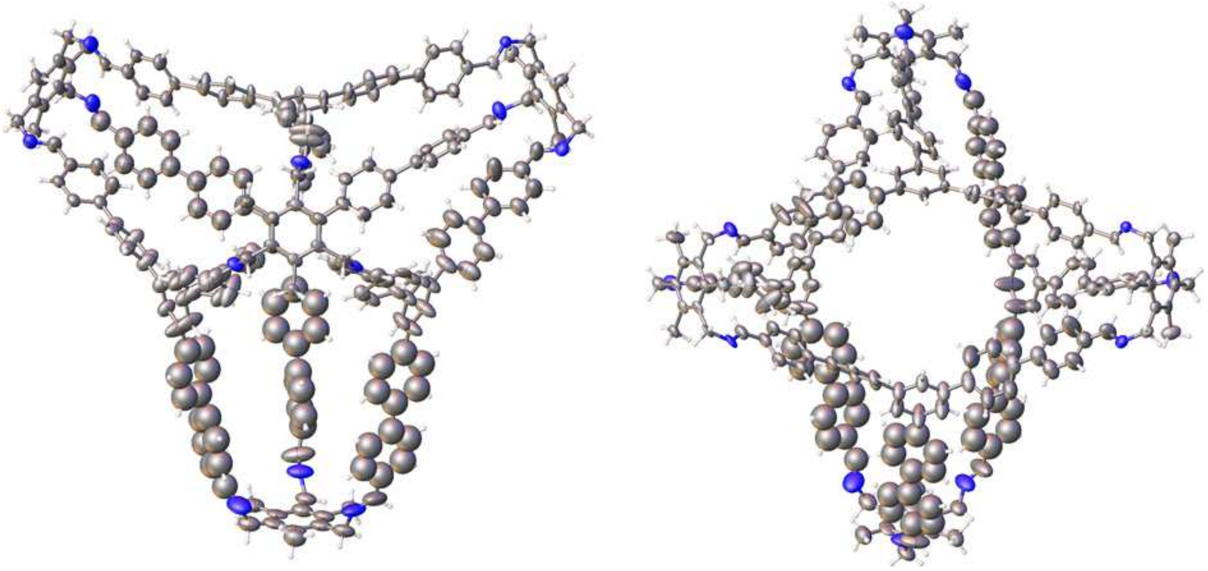

Supplementary Figure 17: Displacement ellipsoid plot of the asymmetric unit from the single crystal structure, **B26**·23.25(C_4H_8O)·23.25($CHCl_3$); ellipsoids are shown at 50% probability level; two views are shown. The general colouring scheme for the displacement ellipsoid plots are as follows; C = grey; H = white; N = blue; O = red; Br = brown; Cl = green; and S = yellow.

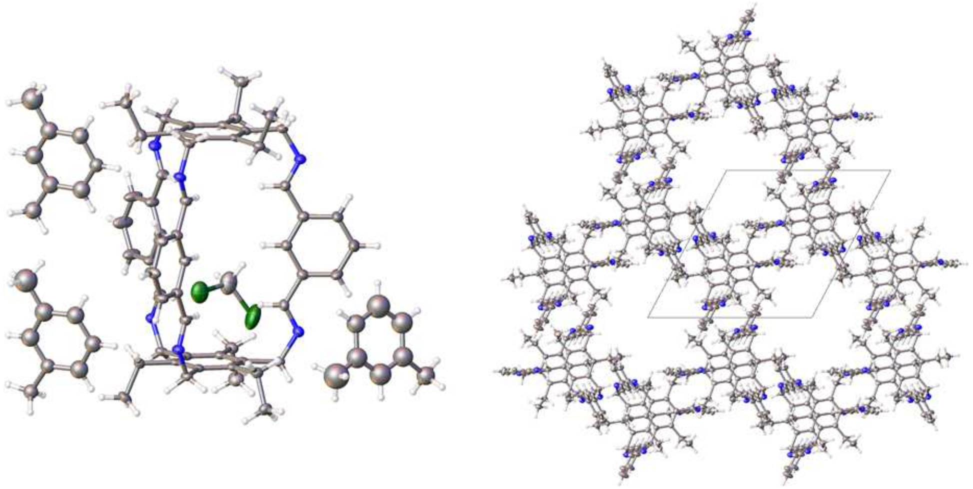

Supplementary Figure 18: Displacement ellipsoid plot of the asymmetric unit from the single crystal structure, **C1**·(C_8H_{10})·0.25(CH_2Cl_2)·0.25 (H_2O) (left); ellipsoids are shown at 50% probability level. Crystal packing diagram showing 1-D channels that are full of *m*-xylene (right); perspective view [100]; *m*-xylene omitted for clarity. The general colouring scheme for the displacement ellipsoid plots are as follows; C = grey; H = white; N = blue; O = red; Br = brown; Cl = green; and S = yellow.

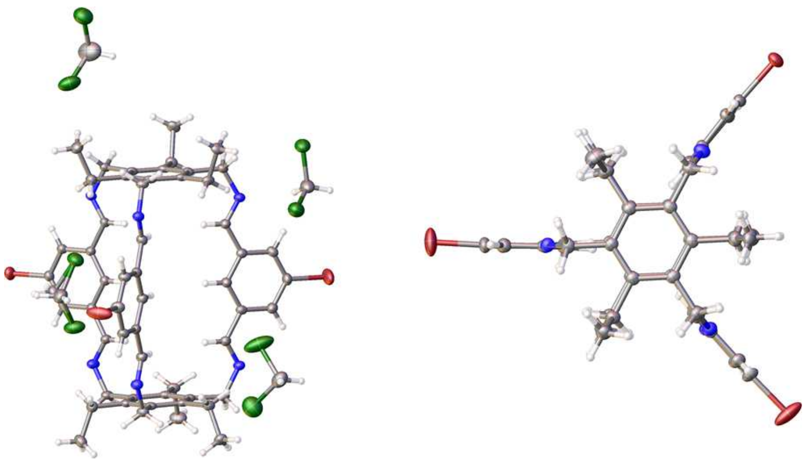

Supplementary Figure 19: Displacement ellipsoid plots from the solvated single crystal structure, **C2**·3(CH_2Cl_2) (left); also shown for **C2** only (right); ellipsoids are displayed at 50% probability level. The general colouring scheme for the displacement ellipsoid plots are as follows; C = grey; H = white; N = blue; O = red; Br = brown; Cl = green; and S = yellow.

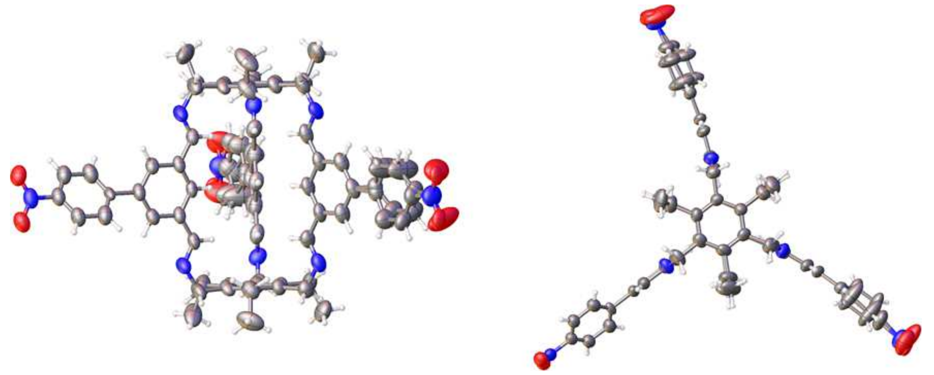

Supplementary Figure 20: Displacement ellipsoid plots from the single crystal structure, **C7**·2.66($CHCl_3$)·1.5(C_4H_8O)·1.6(H_2O); two views shown; ellipsoids displayed at 50% probability level; disordered solvent omitted for clarity. The general colouring scheme for the displacement ellipsoid plots are as follows; C = grey; H = white; N = blue; O = red; Br = brown; Cl = green; and S = yellow.

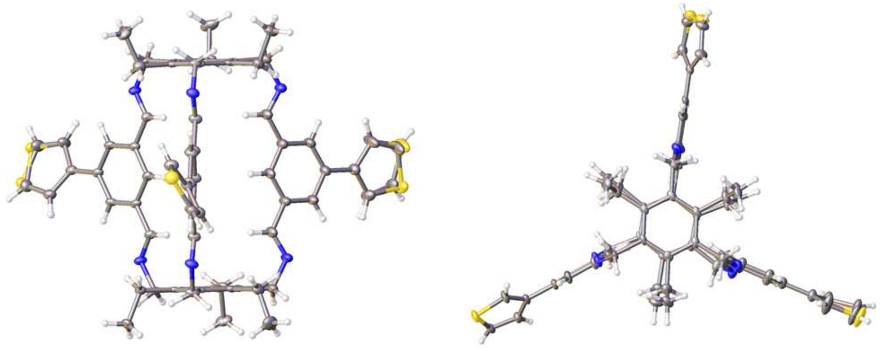

Supplementary Figure 21: Displacement ellipsoid plots the single crystal structure, **C9**·4.66($CDCl_3$)·0.48(C_2H_3N); two views shown; ellipsoids displayed at 50% probability level; disordered solvent omitted for clarity. The general colouring scheme for the displacement ellipsoid plots are as follows; C = grey; H = white; N = blue; O = red; Br = brown; Cl = green; and S = yellow.

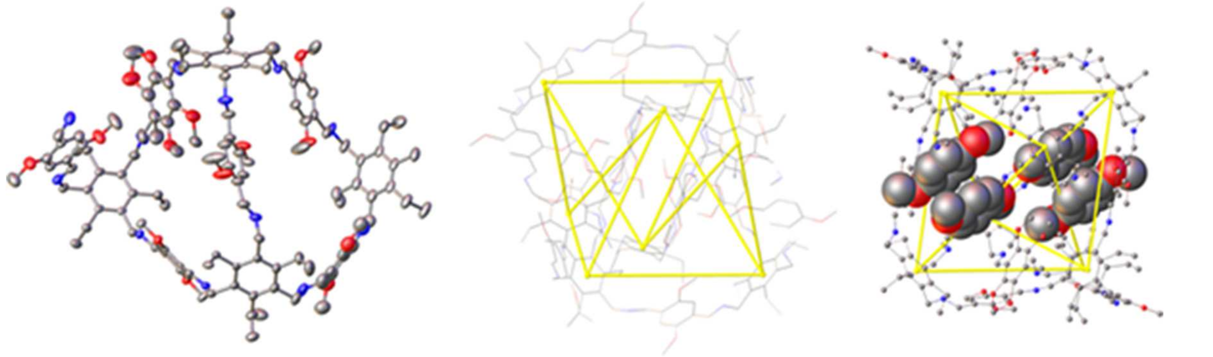

Supplementary Figure 22: Displacement ellipsoid plot of the asymmetric unit from the single crystal structure, **C13**$_{[8+12]}$·3.67(CH_2Cl_2)·6(CH_4O)·1.5(H_2O) (left); ellipsoids displayed at 30% probability level; disordered solvent omitted for clarity. Connectivity of tritopic aromatic groups, via linear ditopic dimethoxy group, shown in yellow (middle). Offset π - π stacking interactions are evident between dimethoxy benzene groups in the crystal structure (right, dimethoxy benzene groups shown in space filling format). The general colouring scheme for the displacement ellipsoid plots are as follows; C = grey; H = white; N = blue; O = red; Br = brown; Cl = green; and S = yellow.

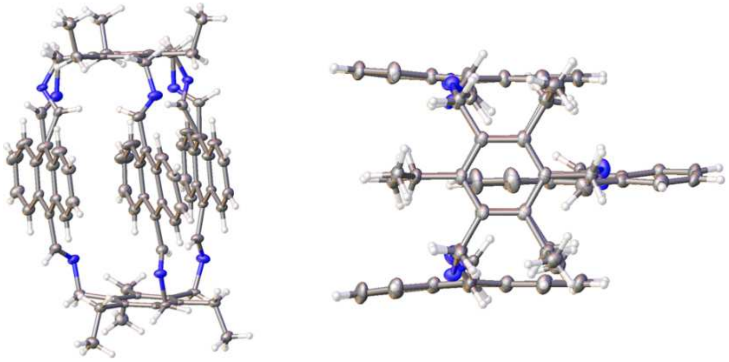

Supplementary Figure 23: Displacement ellipsoid plots from the single crystal structure, **C14**·2(C_4H_8O); two views shown; ellipsoids displayed at 50% probability level. The general colouring scheme for the displacement ellipsoid plots are as follows; C = grey; H = white; N = blue; O = red; Br = brown; Cl = green; and S = yellow.

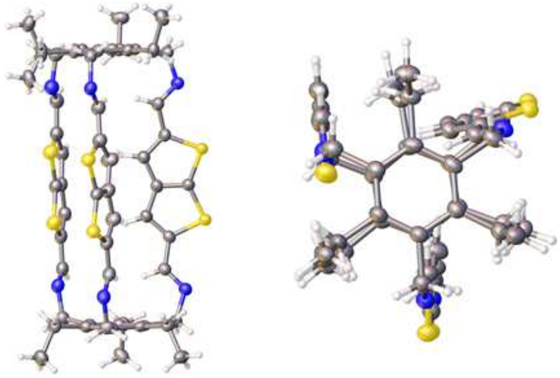

Supplementary Figure 24: Displacement ellipsoid plots from the solvated single crystal structures, **C21**$_{[2+3]}$·0.5(CH_2Cl_2)·0.25(Et2O); solvent omitted for clarity; ellipsoids displayed at 50% probability level; two views shown. The general colouring scheme for the displacement ellipsoid plots are as follows; C = grey; H = white; N = blue; O = red; Br = brown; Cl = green; and S = yellow.

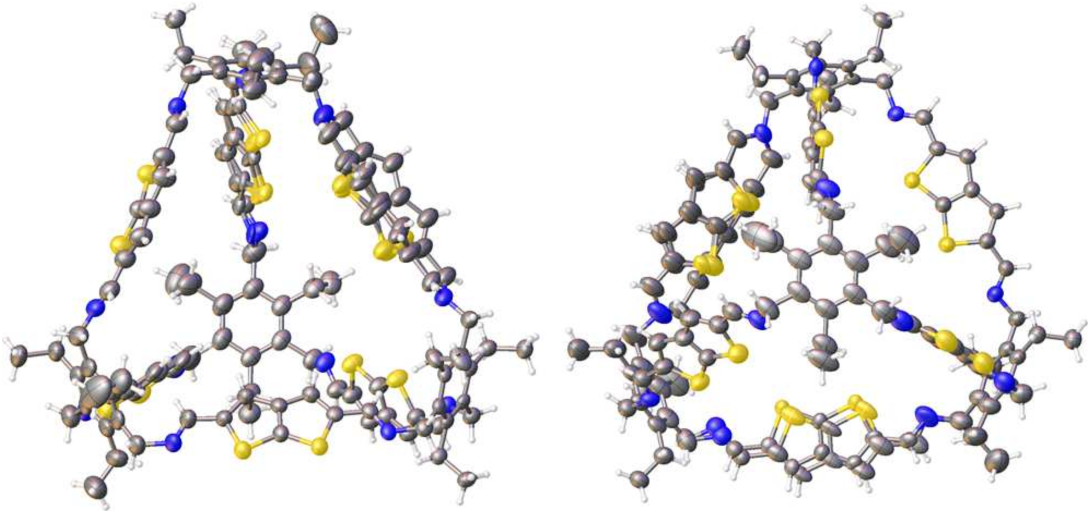

Supplementary Figure 25: Displacement ellipsoid plots from the solvated single crystal structures, **C21**$_{[4+6]}$·7.12(CH_2Cl_2)·5.12(C_2H_3N)·0.25(H_2O); solvent omitted for clarity; ellipsoids displayed at 50% probability level two views shown. The general colouring scheme for the displacement ellipsoid plots are as follows; C = grey; H = white; N = blue; O = red; Br = brown; Cl = green; and S = yellow.

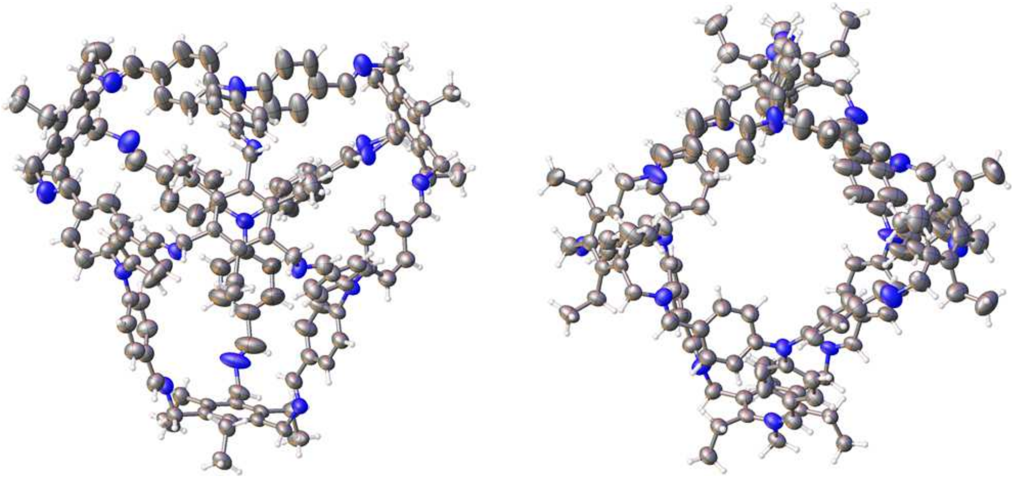

Supplementary Figure 26: Displacement ellipsoid plots from the single crystal structure, **C23**·6.5(CH_2Cl_2)·7.5(C_2H_3N); solvent omitted for clarity; ellipsoids displayed at 50% probability level; two views shown. The general colouring scheme for the displacement ellipsoid plots are as follows; C = grey; H = white; N = blue; O = red; Br = brown; Cl = green; and S = yellow.

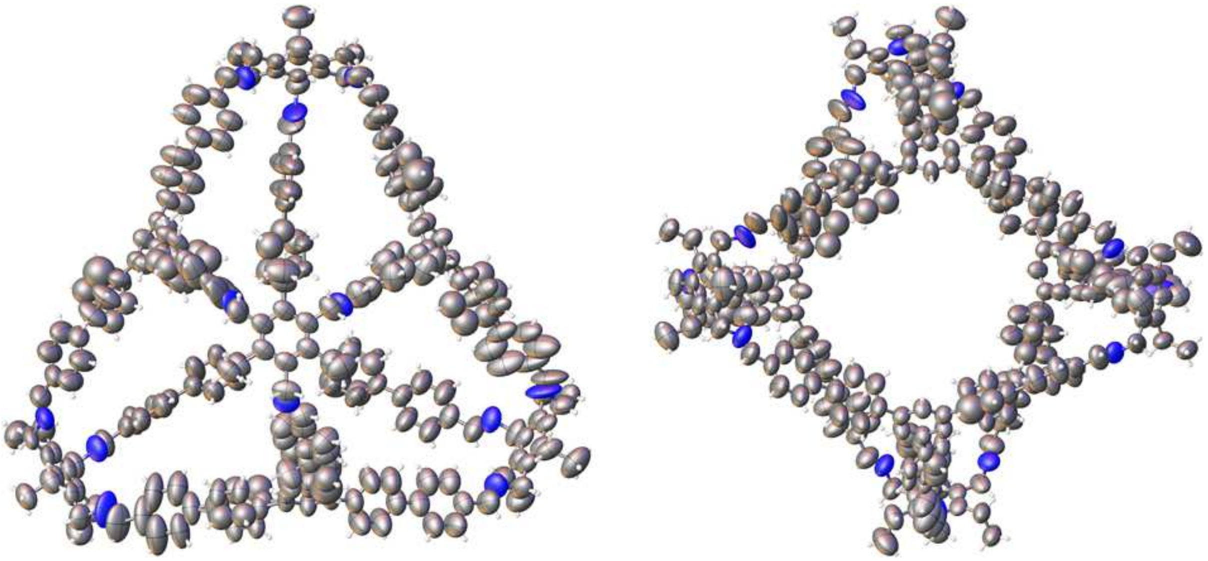

Supplementary Figure 27: Displacement ellipsoid plots from the single crystal structure, **C26**·12.25($CHCl_2$)·12.25($C_4H_{10}O$); **C26** shown in entirety; ellipsoids displayed at 50% probability level; two views shown. The general colouring scheme for the displacement ellipsoid plots are as follows; C = grey; H = white; N = blue; O = red; Br = brown; Cl = green; and S = yellow.

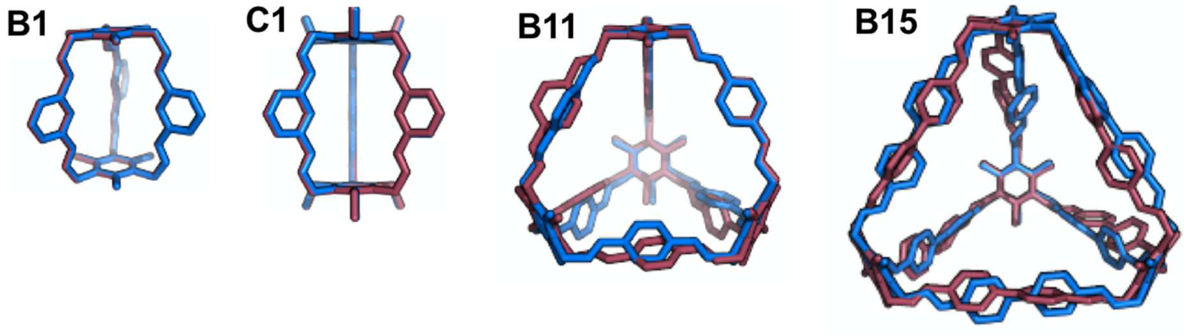

Supplementary Figure 28: Overlays between the OPLS-minimised SCXRD structures of each cage (dark red) and the computed lowest energy conformation (blue) for **B1**, **C1**, **B11** and **B15**.

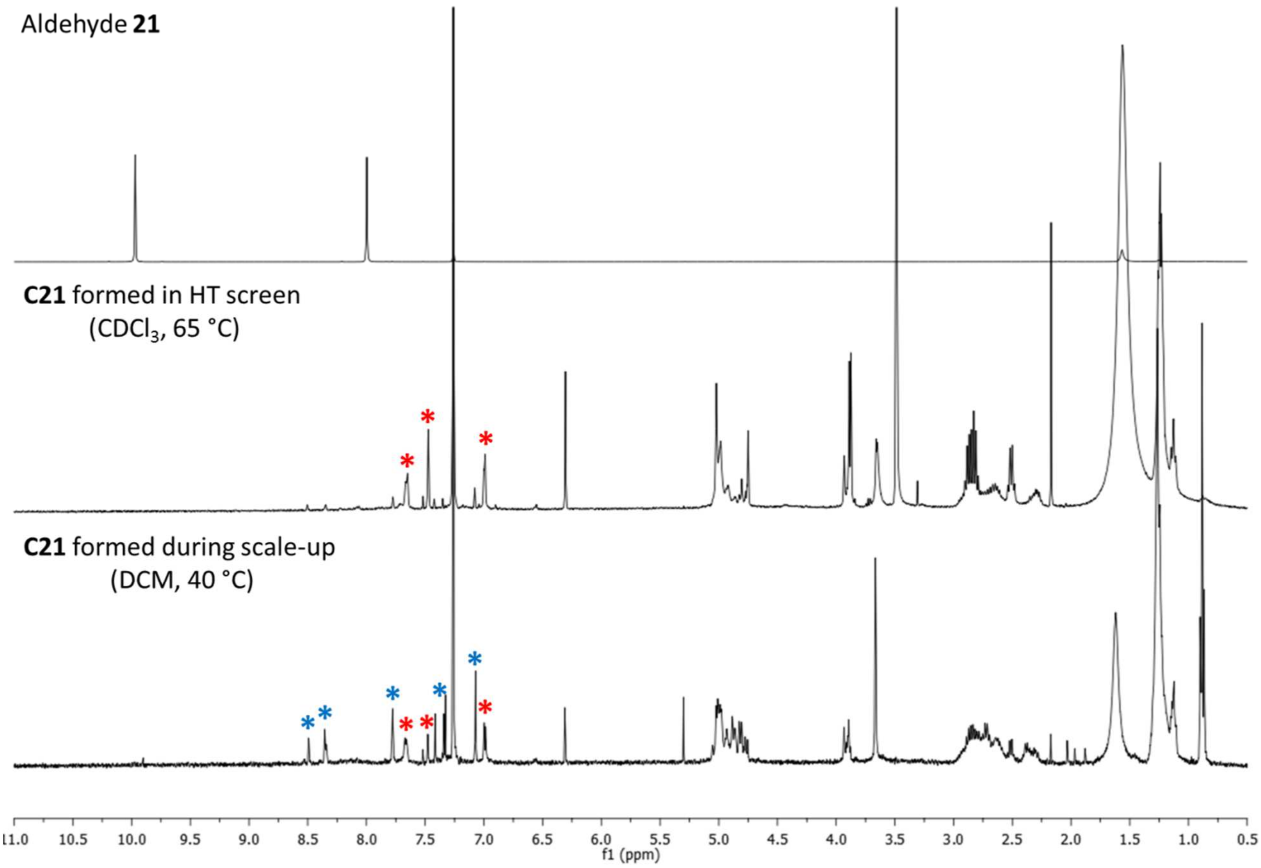

Supplementary Figure 29: Comparison of ^{1}H NMR spectra for cage **C21**. Spectra taken directly from the high-throughput screen (middle) in $CDCl_3$, compared to the mixture obtained during scale-up in DCM (bottom) — crude aliquot of reaction scale-up mixture taken and concentrated under N_2 stream, before dilution in $CDCl_3$ for ^{1}H NMR analysis.

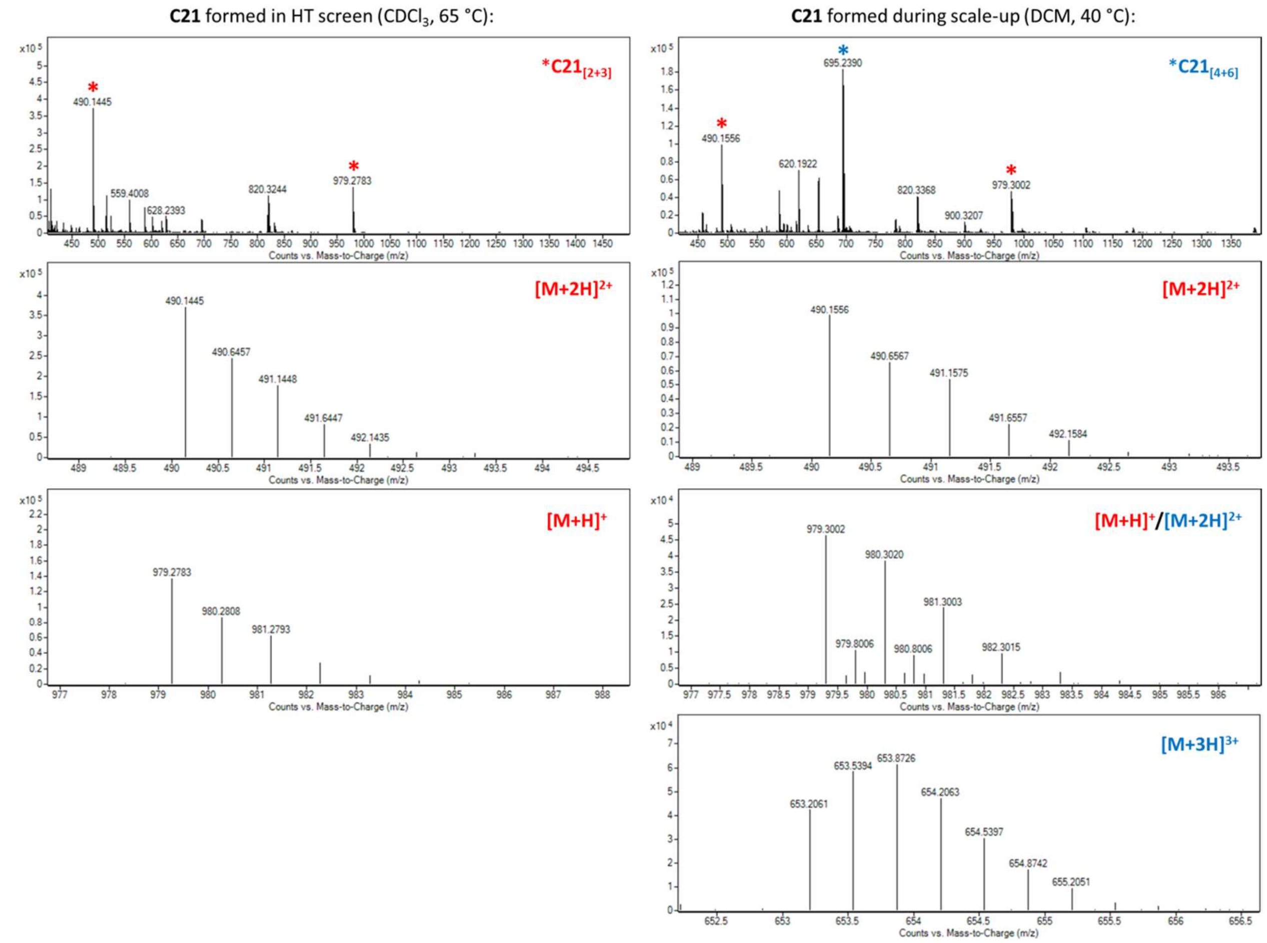

Supplementary Figure 30: Comparison of HRMS spectra for cage **C21**. Spectra taken directly from the high-throughput screen (left), indicating clear formation of a [2+3] cage (*, **C21$_{[2+3]}$**) — $[M+H]^+$ 979.2783 and $[M+2H]^{2+}$ 490.1445, compared to spectra from scale-up reaction (right) indicating clear formation of a mixture of [2+3] (*) and [4+6] (*) cage — overlapping isotope patterns for **C21$_{[2+3]}$** $[M+H]^+$ and **C21$_{[4+6]}$** $[M+2H]^{2+}$ visible at mass ion 979.3002, but $[M+3H]^{3+}$ 653.8726 consistent with **C21$_{[4+6]}$**, and $[M+2H]^{2+}$ 490.1556 consistent with **C21$_{[2+3]}$**.

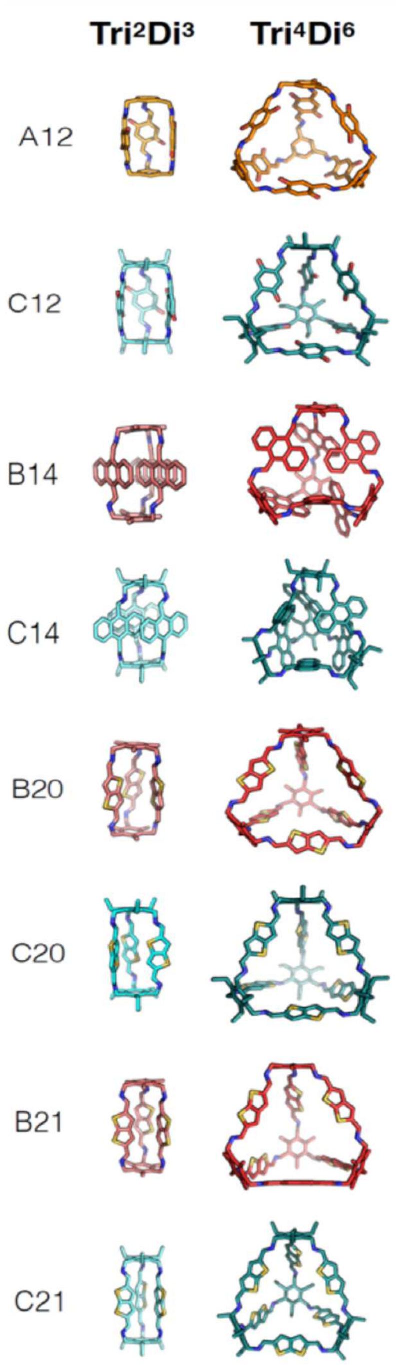

Supplementary Figure 31: The **Tri^{2}Di^3** and **Tri^{4}Di^6** cages for the systems where a **Tri^{4}Di^6** cage was targeted, but a **Tri^{2}Di^3** was found to form. **A**-cages have orange carbons, **B**-cages have maroon carbons and **C**-cages have teal carbons. Remaining atom colouring is as follows; oxygen (red), bromine (brown), boron (pink), silicon and sulfur (yellow), chlorine (green) and nitrogen (blue). Hydrogens are omitted.

a

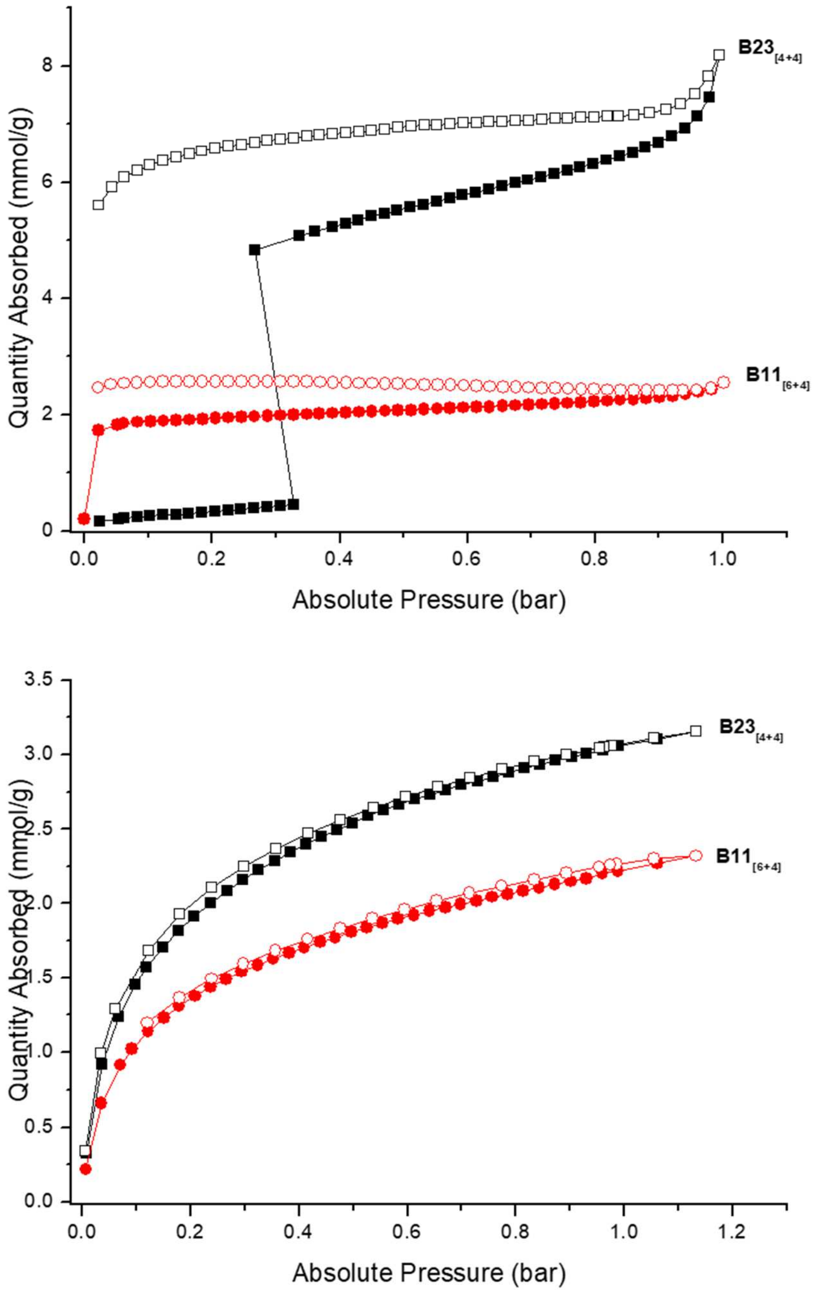

b

Supplementary Figure 32: We investigated the gas sorption properties of **B11** and **B23** after isolation by solvent exchange, which showed that cages can indeed be porous 'as-made'. On comparison to the PXRDs from the HT screen which showed amorphous material on solvent removal, isolation of the cages by solvent exchange leads to more crystalline material (**Supplementary Fig. 33 and 34**). Nitrogen **(a)** and hydrogen **(b)** adsorption (filled shapes) / desorption (empty shapes) isotherms for cages **B11**$_{[6+4]}$ (red, circles) and **B23**$_{[4+4]}$ (black, squares) at 77 K and 1 bar. The porosity was comparable to other organic cages reported previously in the literature, with both cages absorbing significant quantities of H_2 and N_2 at 77 K and 1 bar (**B11**, N_2 2.55 mmol g^{-1}, H_2 2.31 mmol g^{-1}, SA_{BET} = 131 m^2/g; **B23** N_2 8.18 mmol g^{-1}, H_2 3.15 mmol g^{-1}).

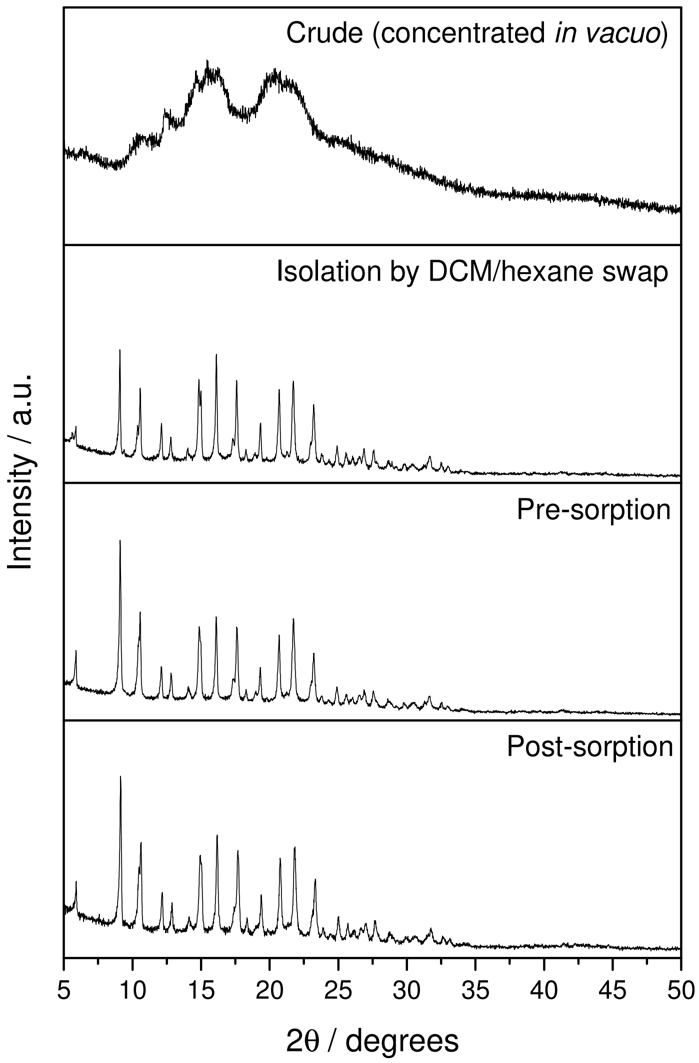

Supplementary Figure 33: Stacked PXRD analysis of **B11 Tri⁴Di⁶** with the as-made, crude product isolated by concentration *in* vacuo (top), post-purification and isolation by solvent exchange from DCM to hexane (2nd from top), pre-sorption after desolvation at 90 °C overnight (2nd to bottom) and post-sorption (bottom).

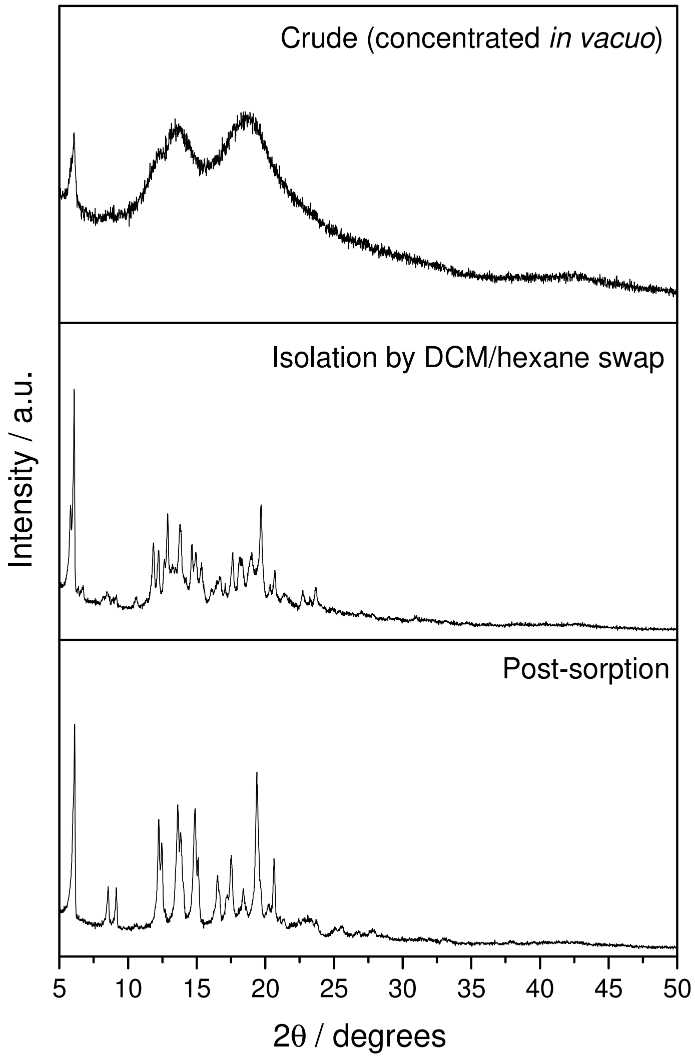

Supplementary Figure 34: Stacked PXRD analysis of **B23 Tri^{4}Tri^4** with the as-made, crude product isolated by concentration *in* vacuo (top), post-purification and isolation by solvent exchange from DCM to hexane (middle), and post-sorption (bottom).

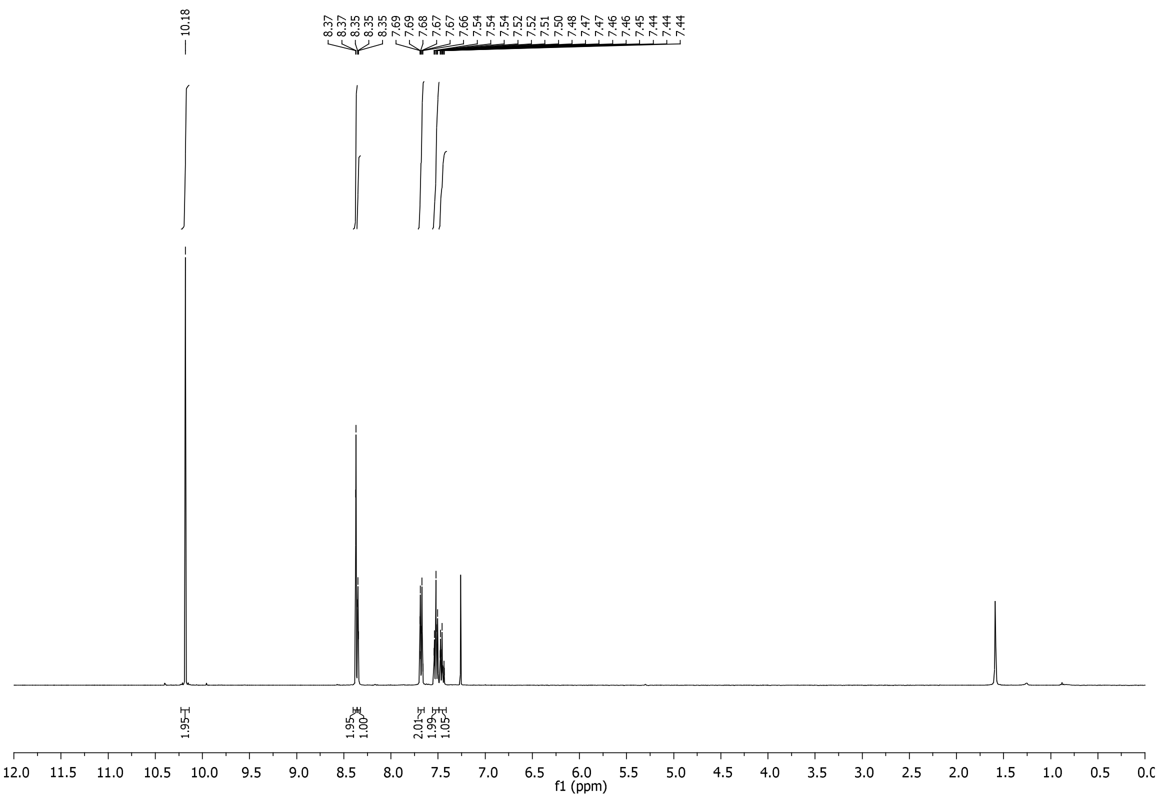

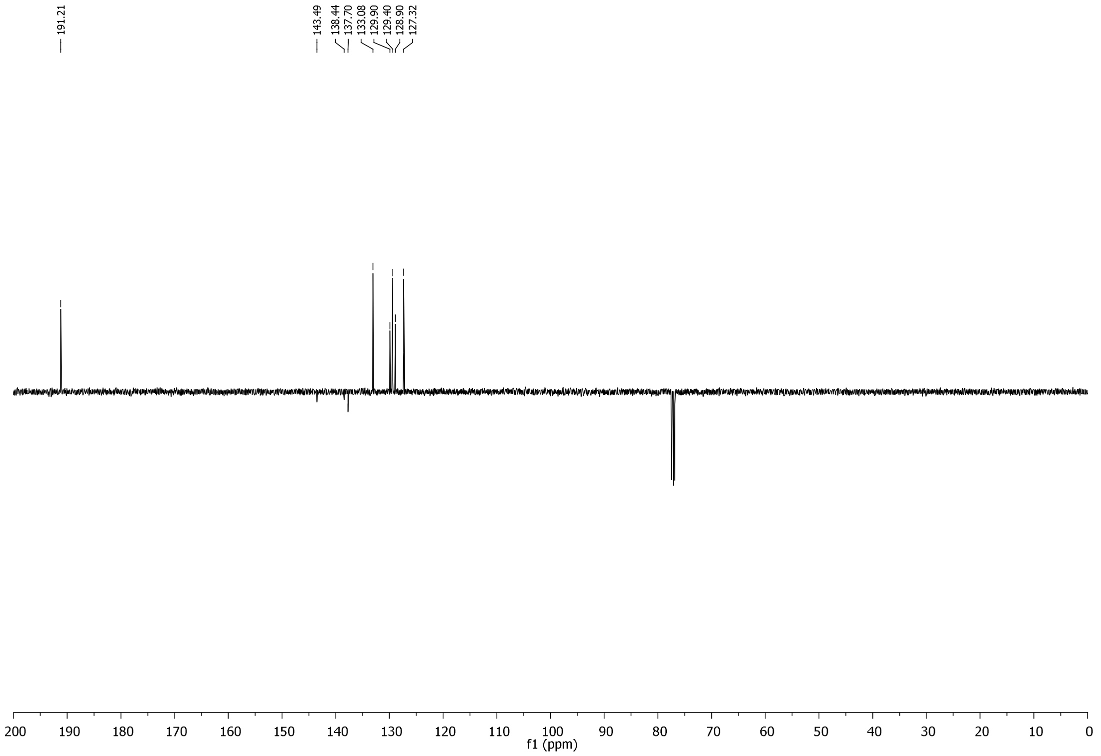

Supplementary Figure 35: ^{1}H NMR ($CDCl_3$; upper) and ^{13}C DEPT NMR ($CDCl_3$; lower) of [1,1'-biphenyl]-3,5-dicarbaldehyde, **5**

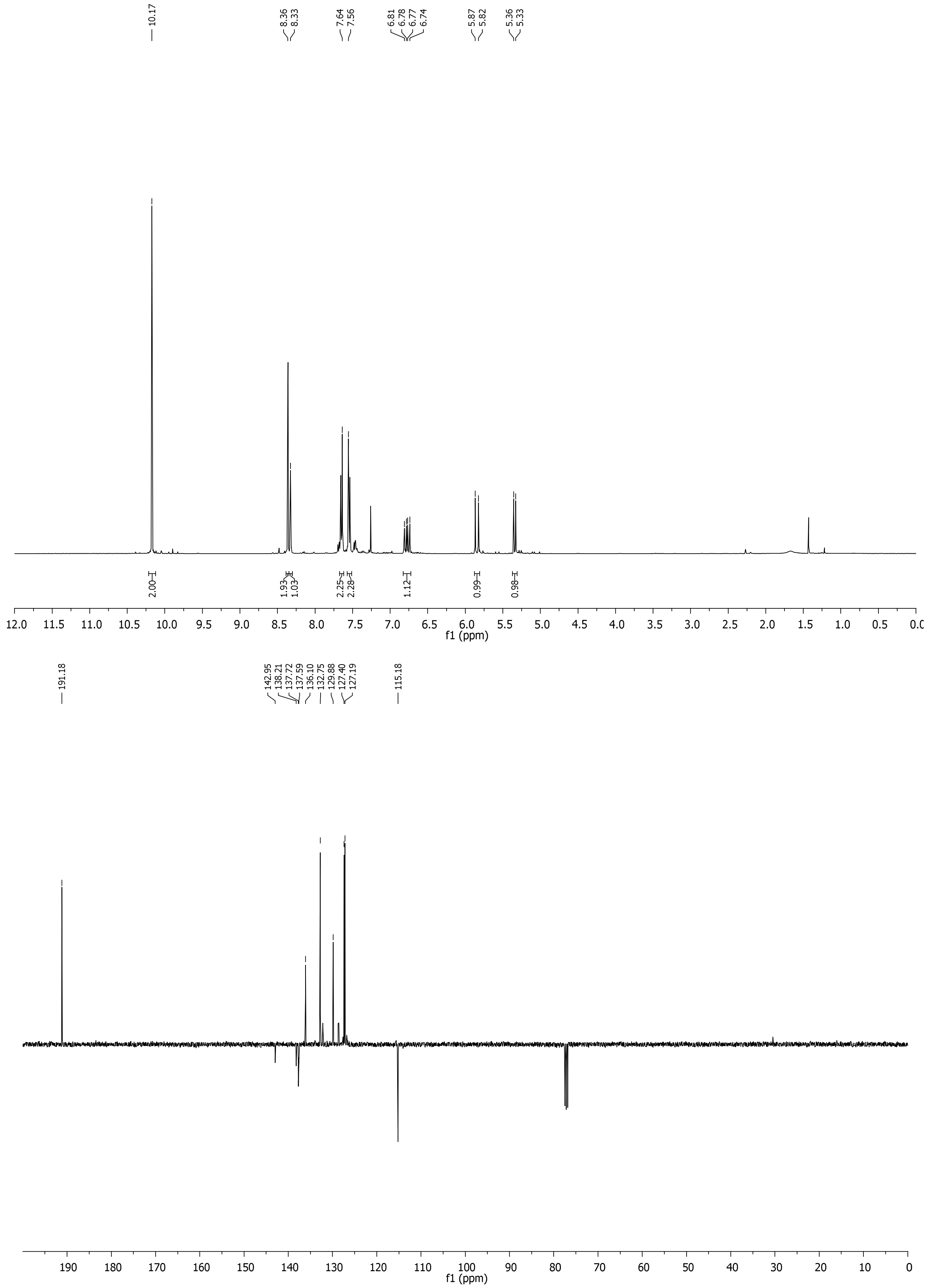

Supplementary Figure 36: ^{1}H NMR ($CDCl_3$; upper) and ^{13}C DEPT NMR ($CDCl_3$; lower) of 4'-vinyl-[1,1'-biphenyl]-3,5-dicarbaldehyde, **6**

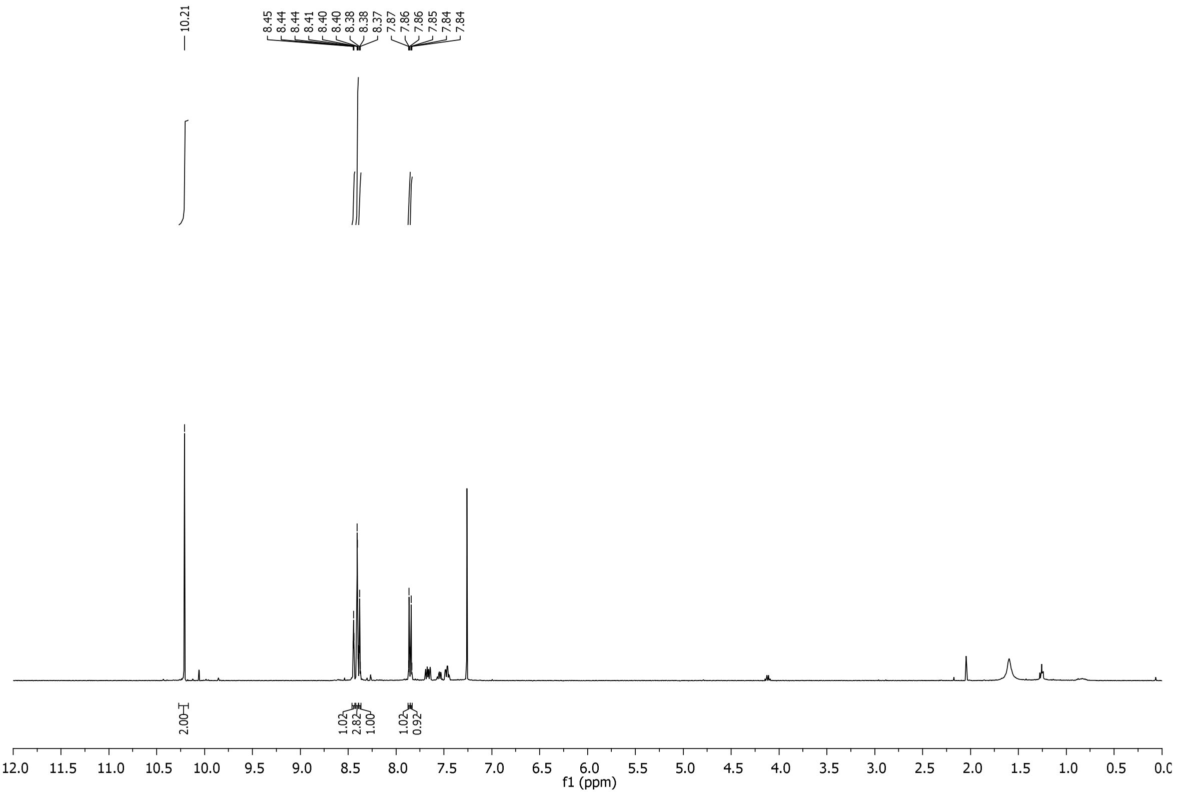

Supplementary Figure 37: ^{1}H NMR ($CDCl_3$) of 4'-nitro-[1,1'-biphenyl]-3,5-dicarbaldehyde, **7**

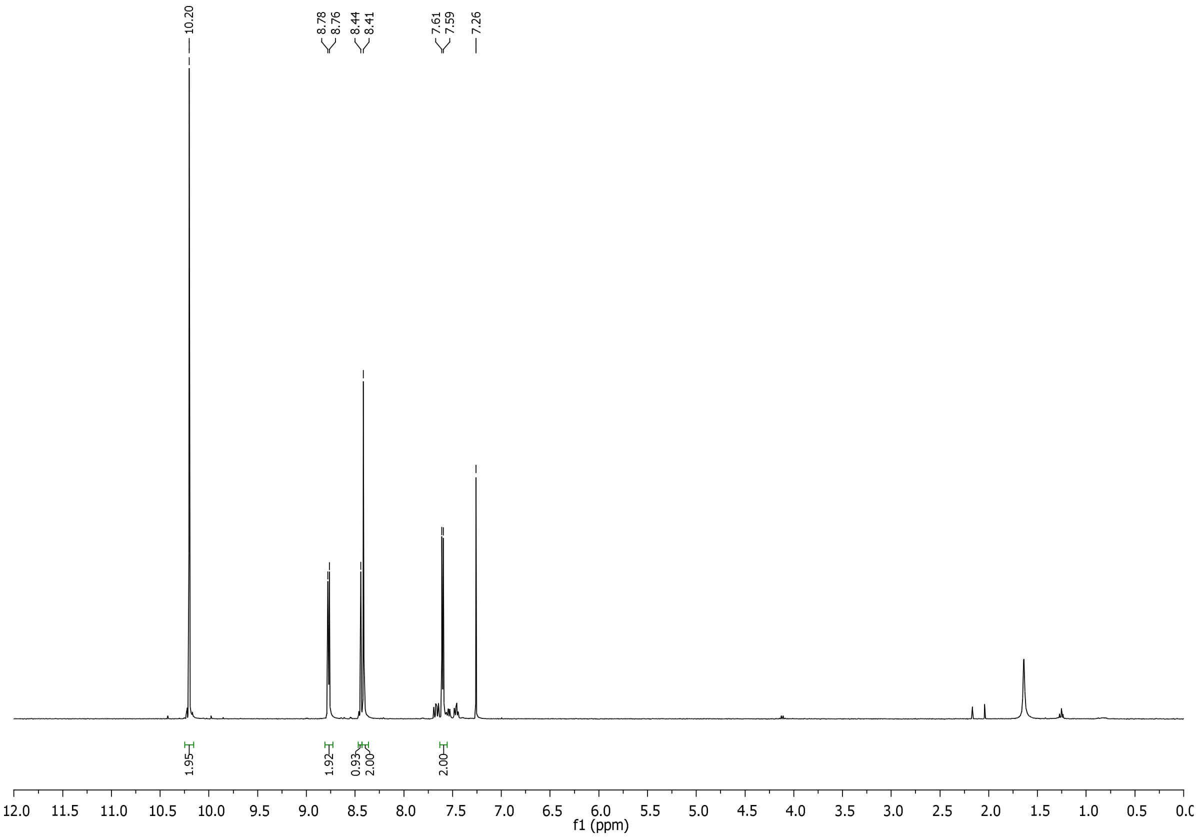

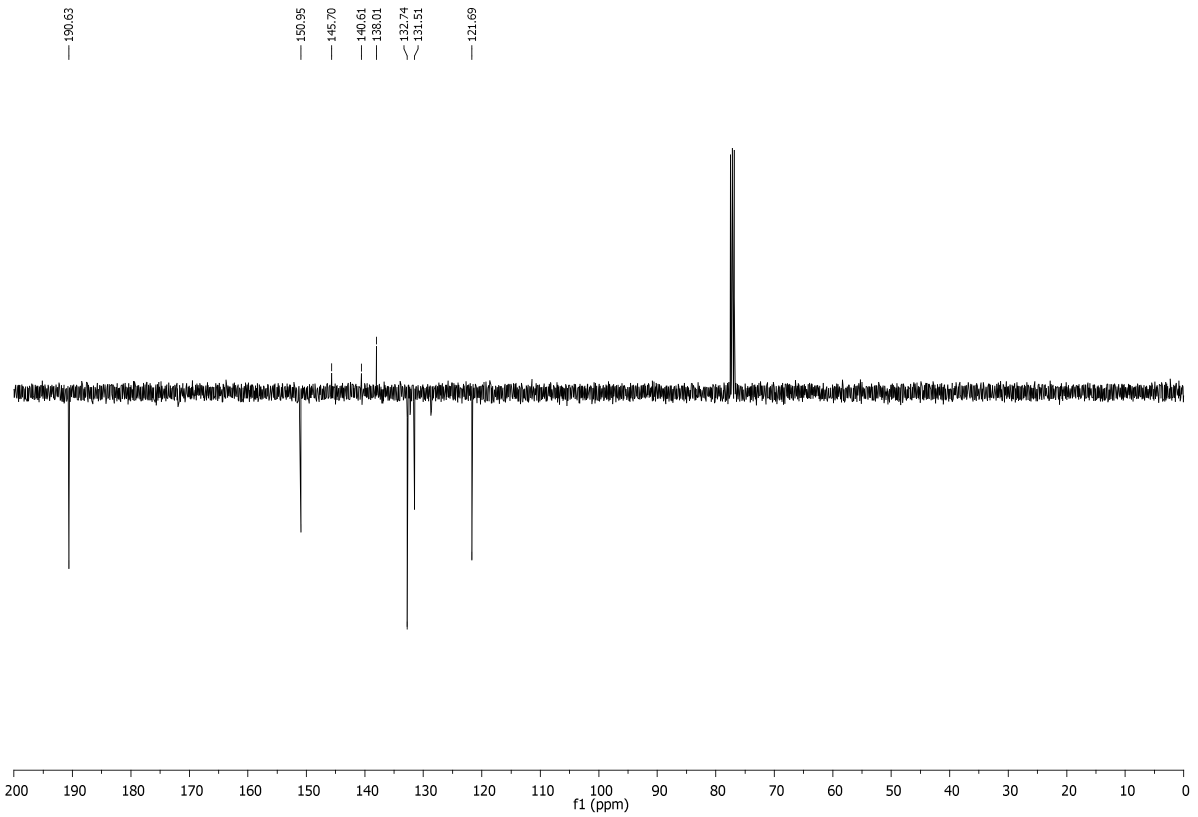

Supplementary Figure 38: ^{1}H NMR ($CDCl_3$; upper) and ^{13}C DEPT NMR ($CDCl_3$; lower) for 5-(pyridin-4-yl)isophthalaldehyde, **8**

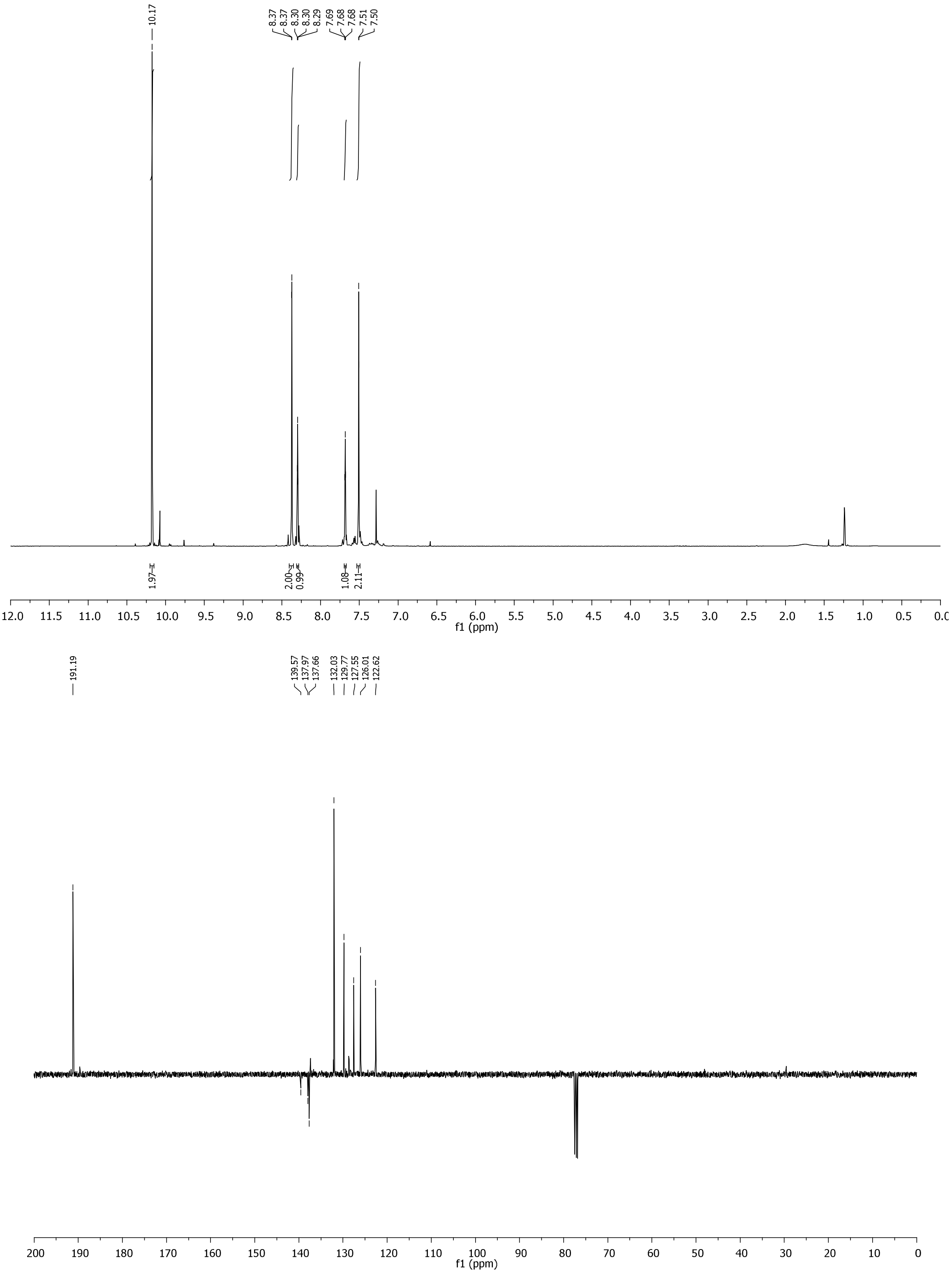

Supplementary Figure 39: ^{1}H NMR ($CDCl_3$; upper) and ^{13}C NMR ($CDCl_3$; lower) of 5-(thiophen-3-yl)isophthalaldehyde, **9**

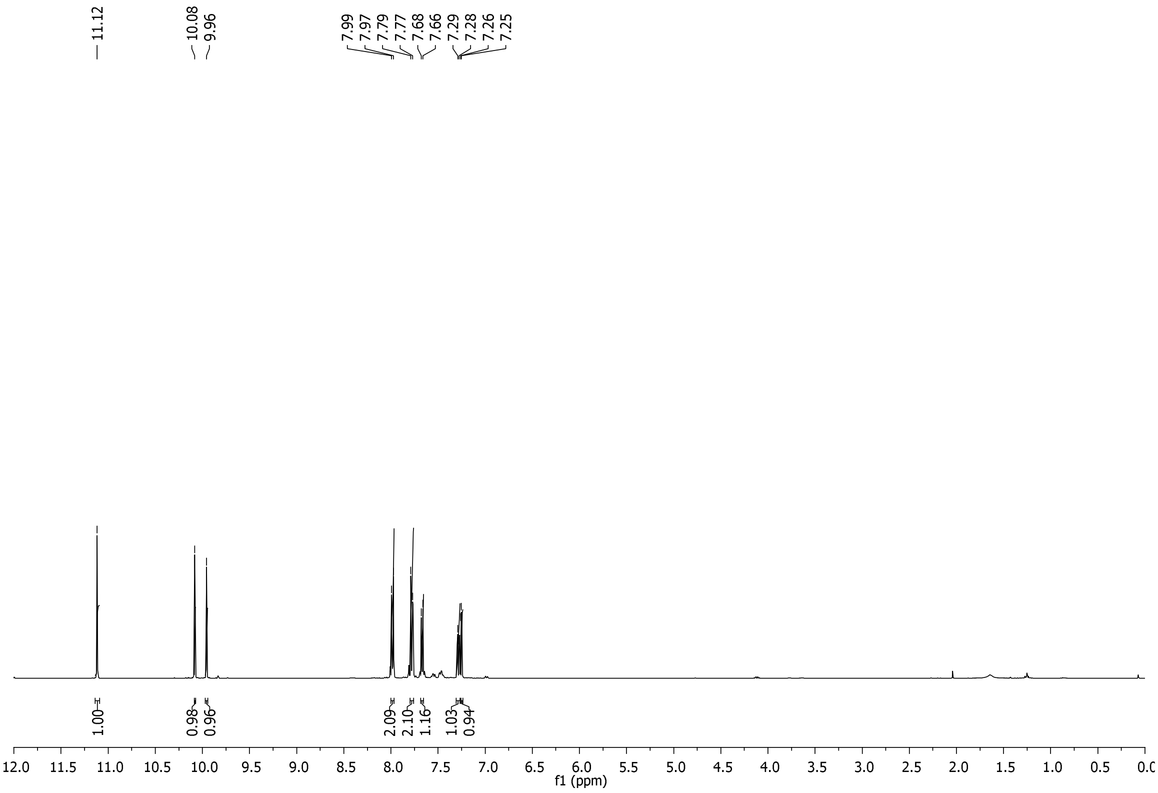

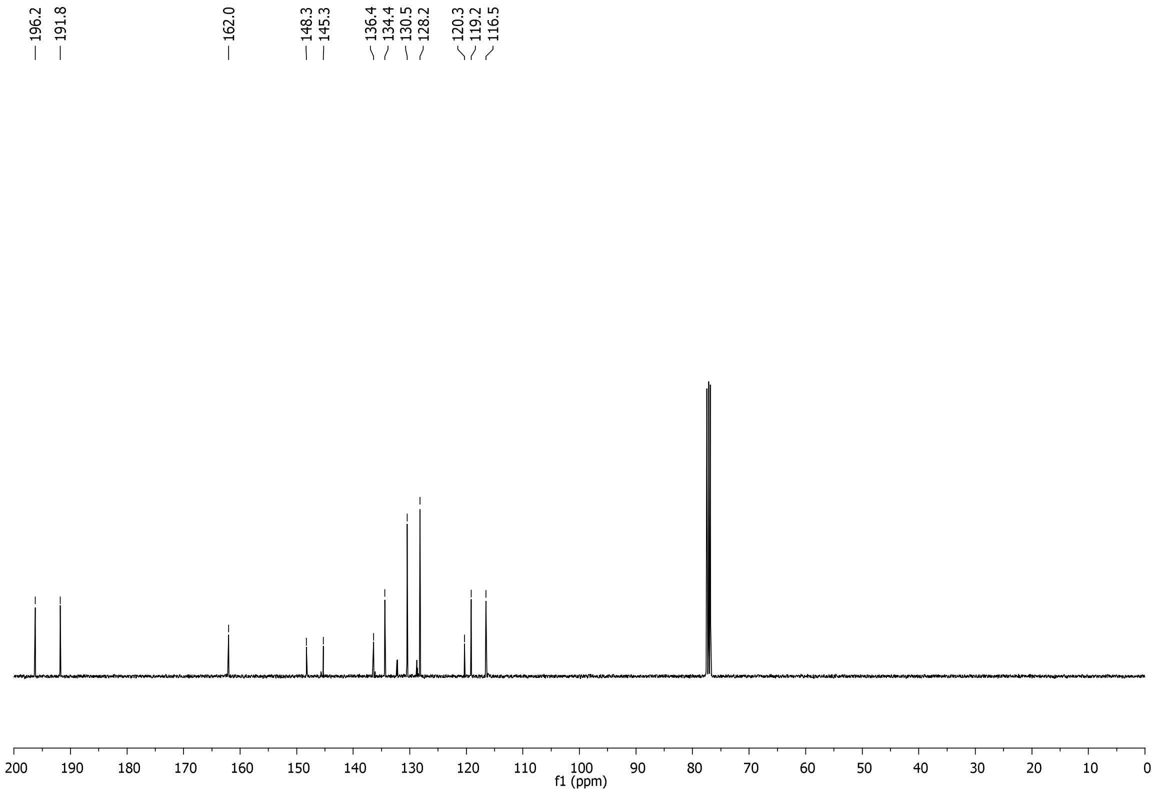

Supplementary Figure 40: ^{1}H NMR ($CDCl_3$; upper) and ^{13}C NMR ($CDCl_3$; lower) for 3-hydroxy-[1,1'-biphenyl]-4,4'-dicarbaldehyde, **16**

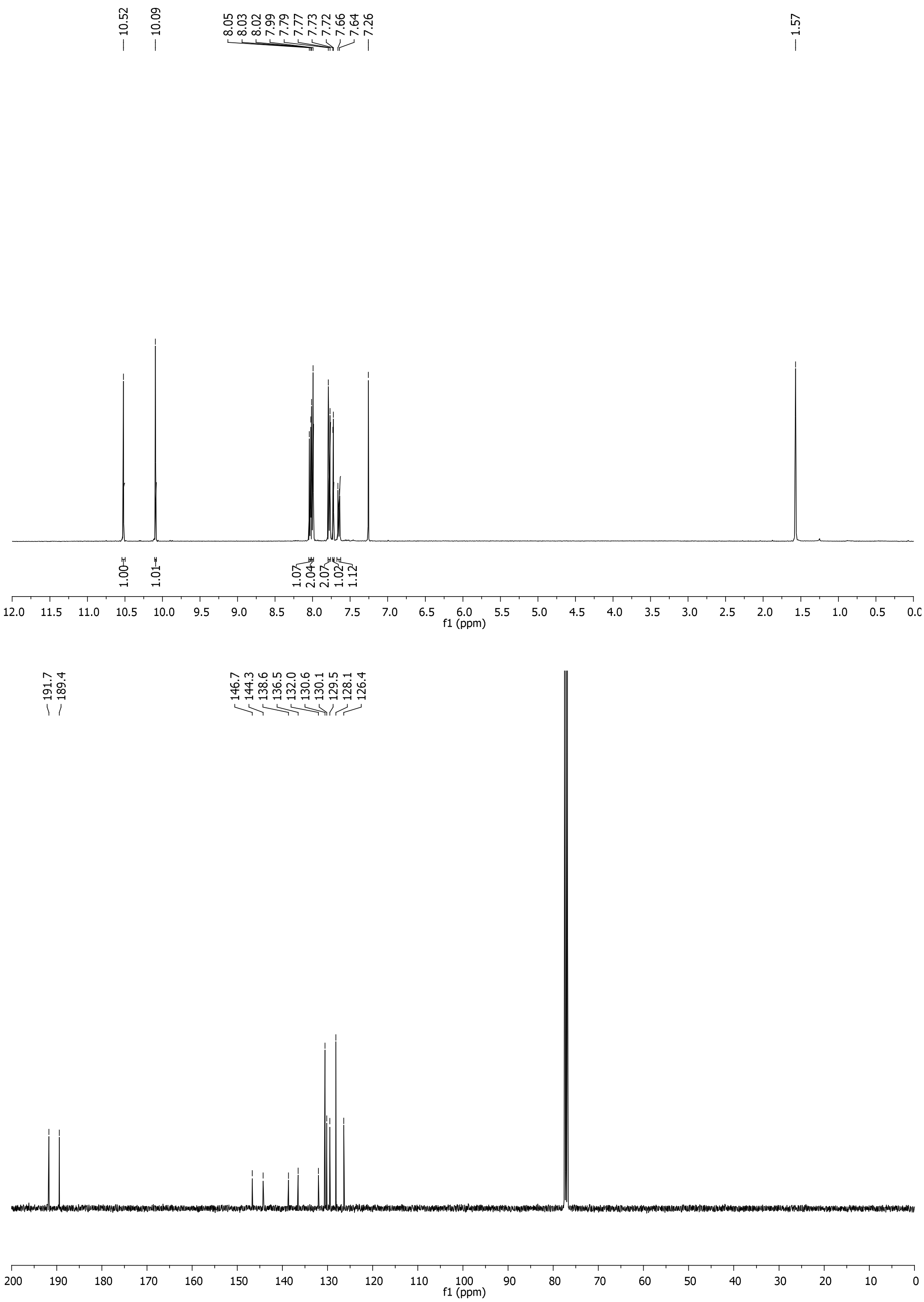

Supplementary Figure 41: ^{1}H NMR ($CDCl_3$; upper) and ^{13}C NMR ($CDCl_3$; lower) for 3-chloro-[1,1'-biphenyl]-4,4'-dicarbaldehyde, **17**

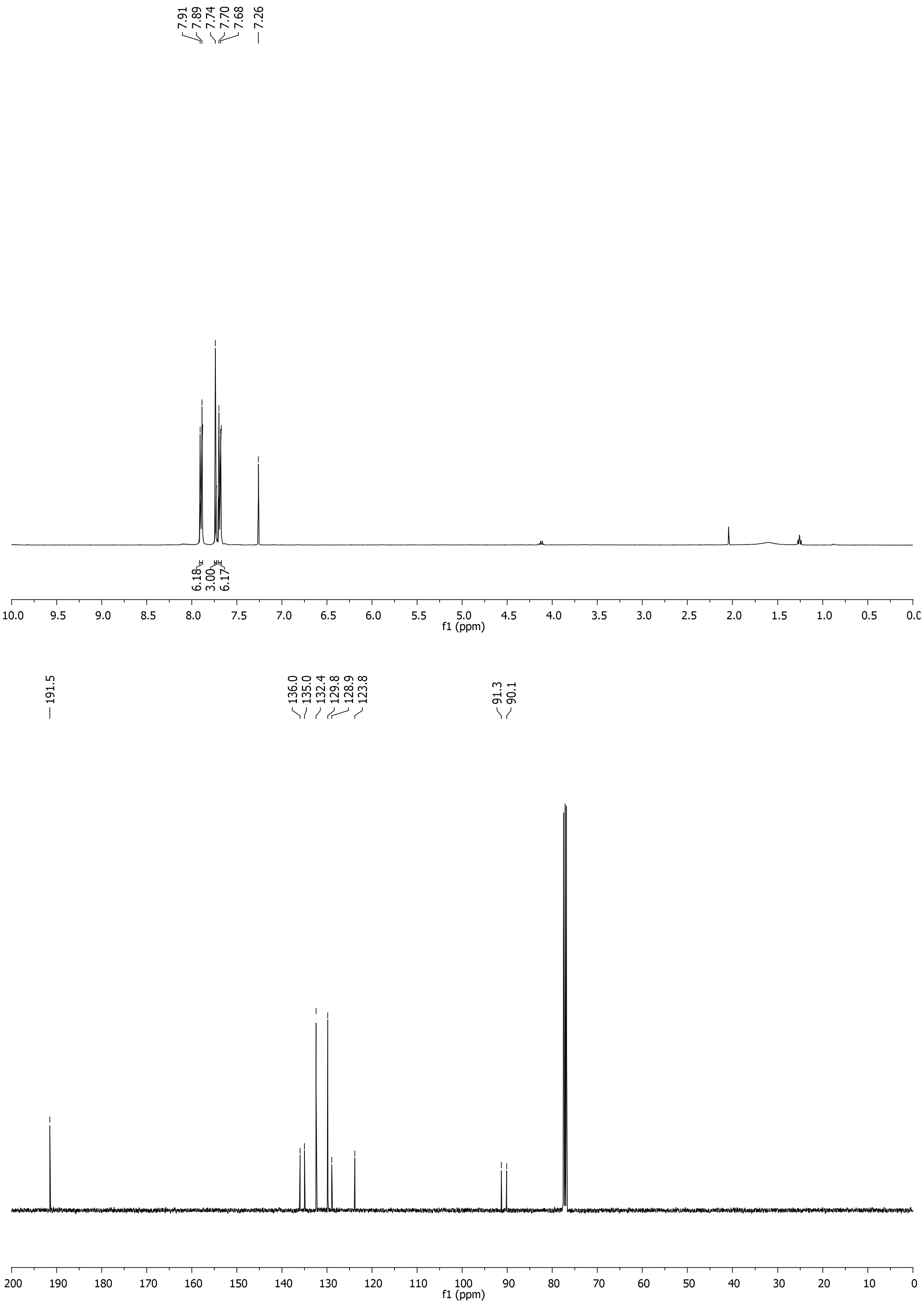

Supplementary Figure 42: ^{1}H NMR ($CDCl_3$; upper) and ^{13}C NMR ($CDCl_3$; lower) for 4,4',4''-(benzene-1,3,5-triyltris(ethyne-2,1-diyl))tribenzaldehyde, **25**

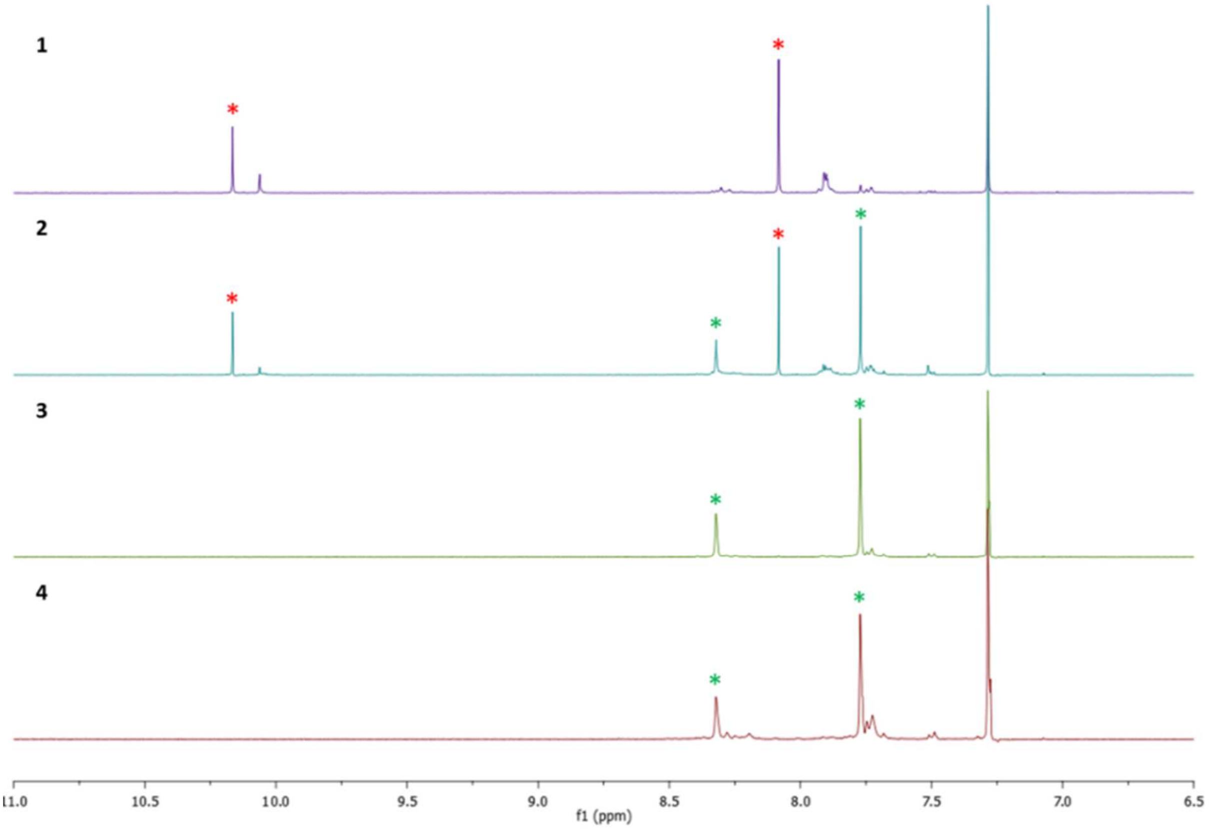

Supplementary Figure 43: Stacked ^{1}H NMR spectra from reactions **1–4** of the optimisation of the model cage reaction (**Supplementary Table 15**) showing conversion of terephthalaldehyde (**11**)* to cage (**B11**)*.

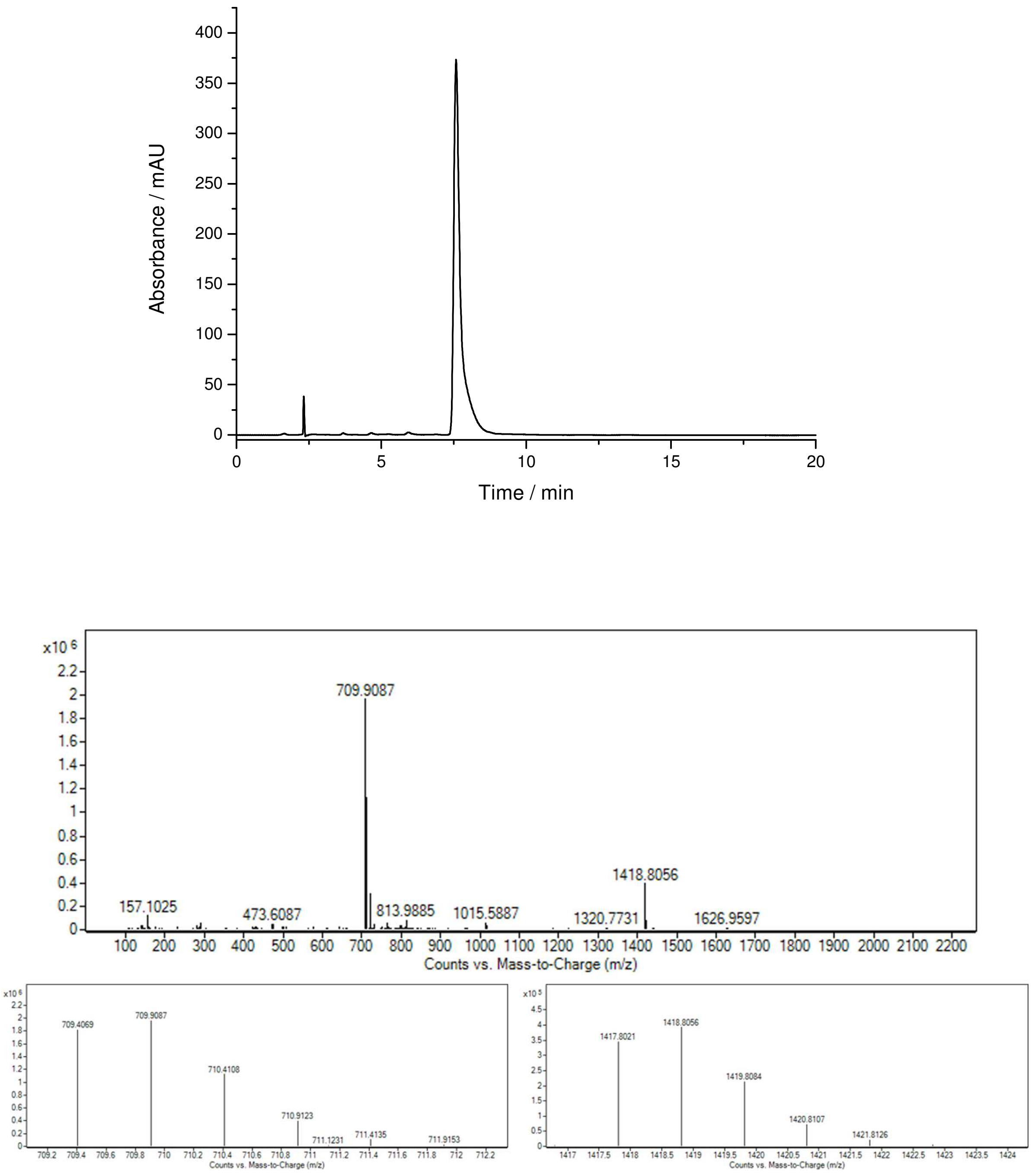

Supplementary Figure 44: HPLC trace (upper) and HRMS spectra (lower) for cage **B11$_{[4+6]}$**.

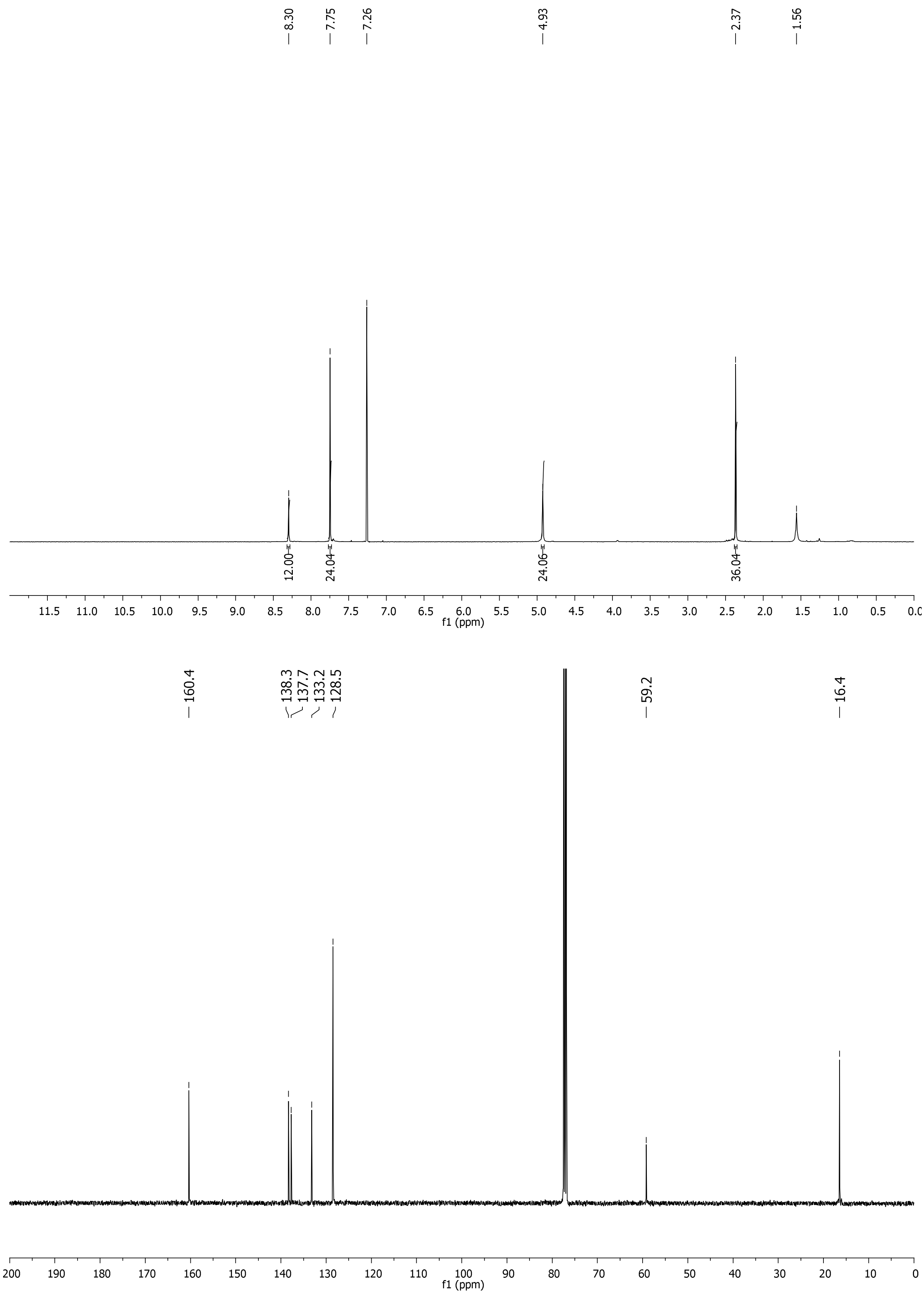

Supplementary Figure 45: ^{1}H NMR ($CDCl_3$; upper) and ^{13}C NMR ($CDCl_3$; lower) for cage **B11$_{[4+6]}$**.

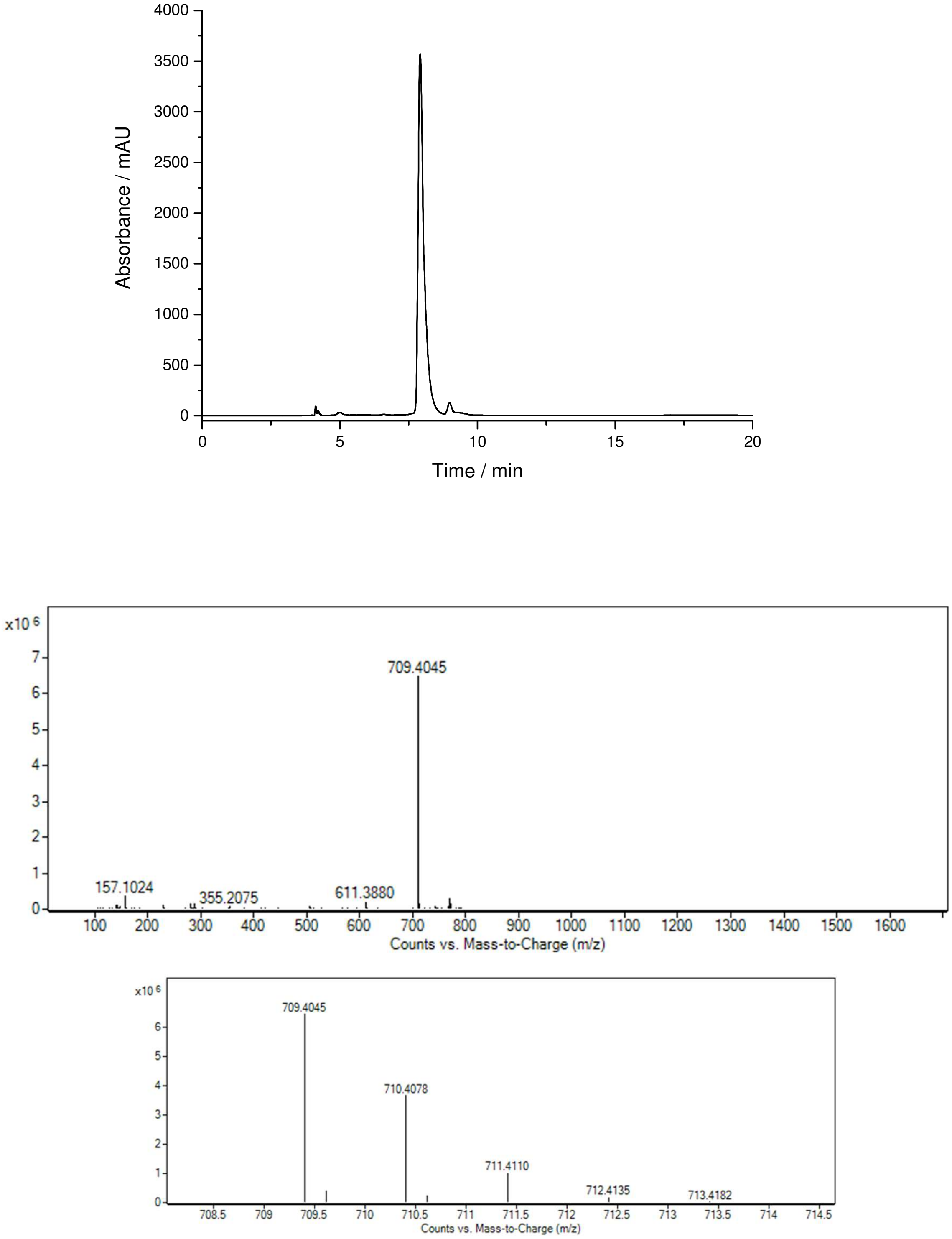

Supplementary Figure 46: HPLC trace (upper) and HRMS spectra (lower) for cage **B1** $_{[2+3]}$.

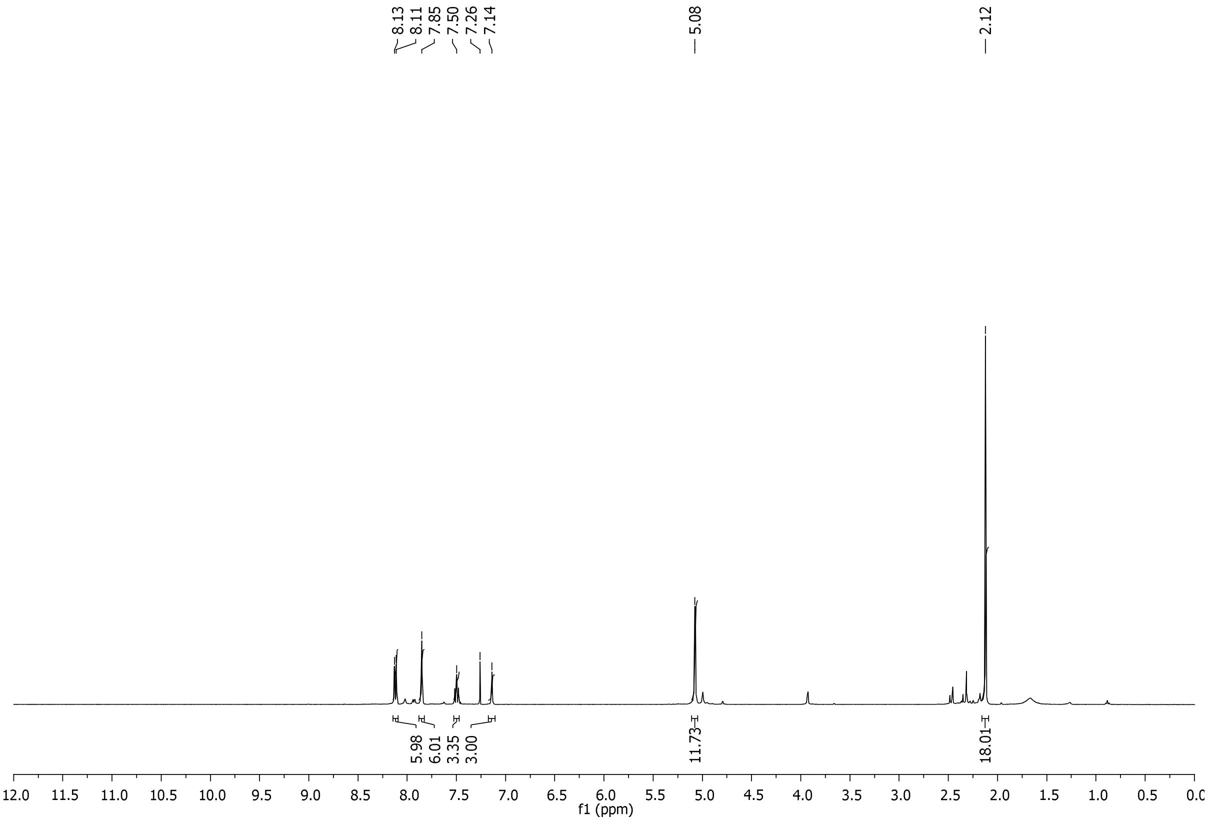

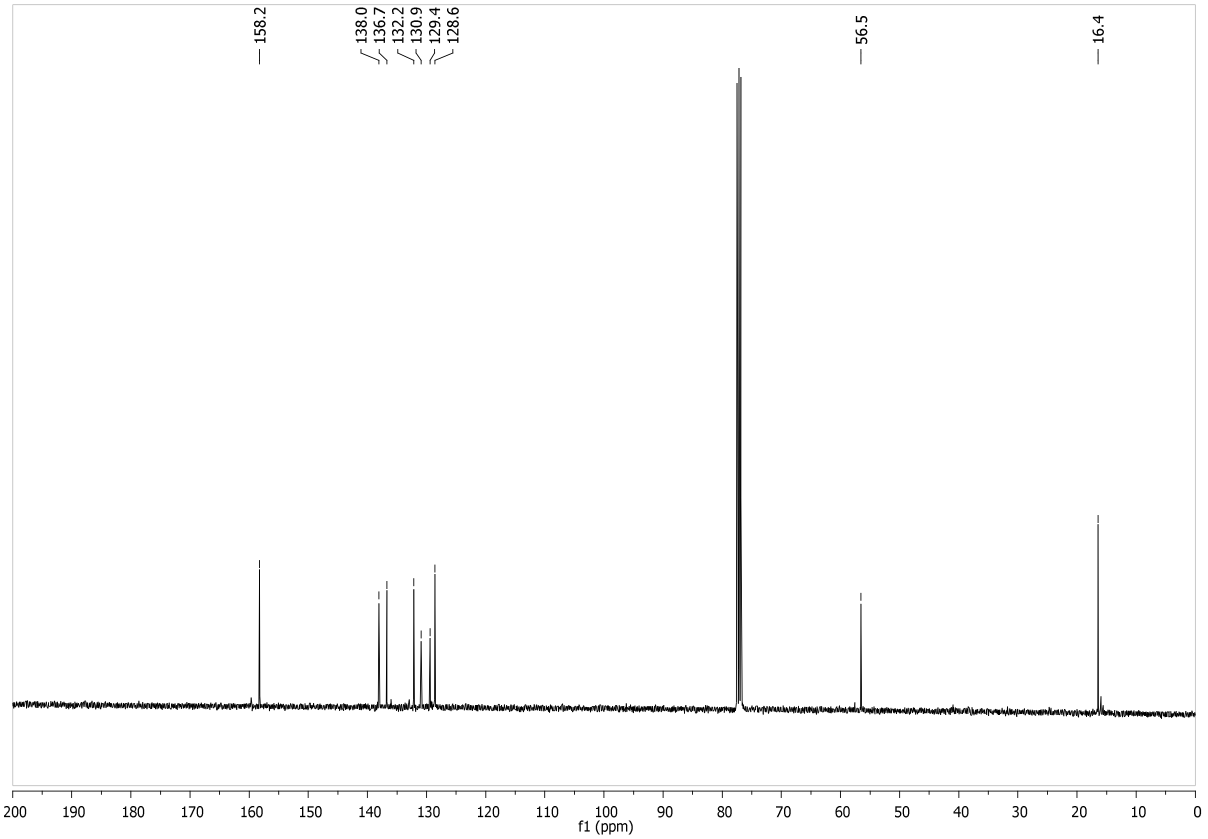

Supplementary Figure 47: ^{1}H NMR ($CDCl_3$; upper) and ^{13}C NMR ($CDCl_3$; lower) for cage **$B1_{[2+3]}$**.

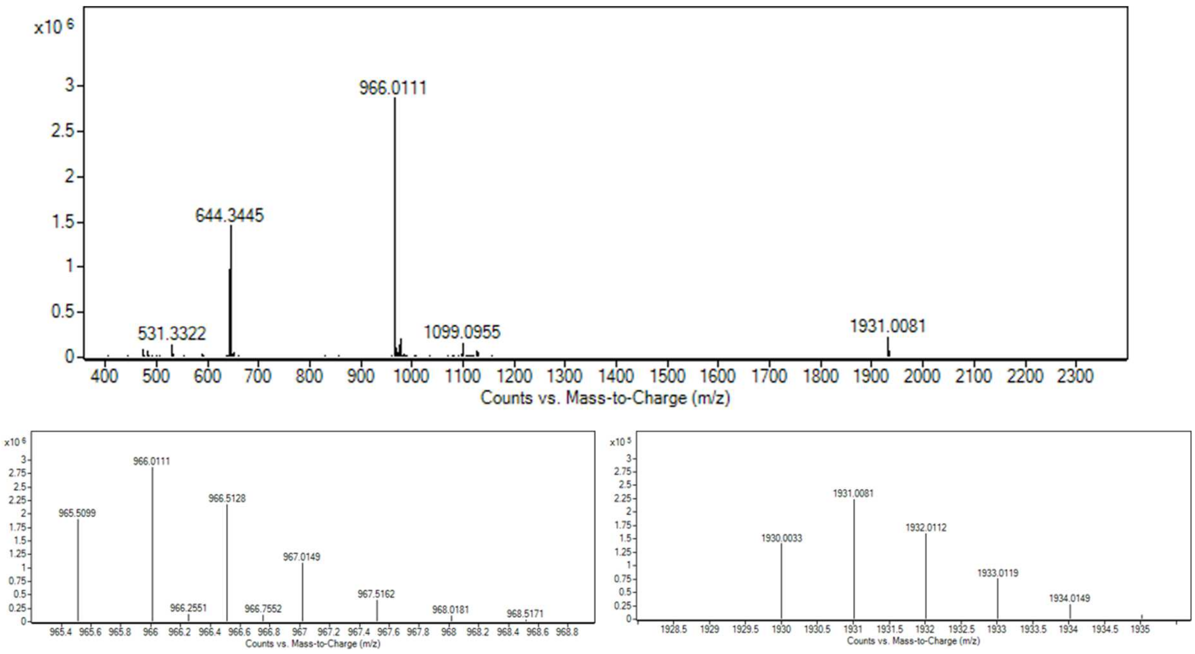

Supplementary Figure 48: HRMS spectra for cage **B23$_{[4+4]}$**.

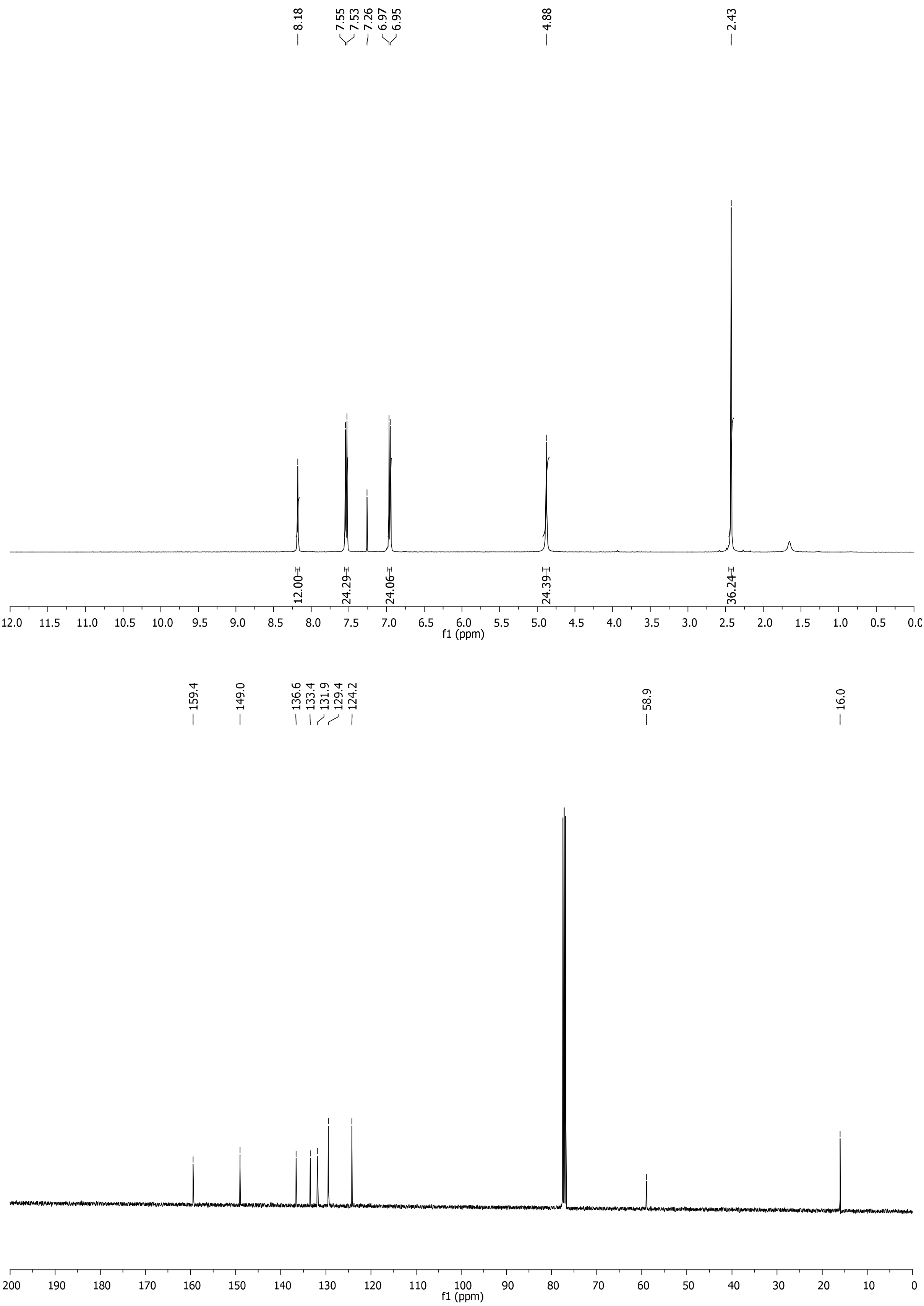

Supplementary Figure 49: ^{1}H NMR ($CDCl_3$; upper) and ^{13}C NMR ($CDCl_3$; lower) for cage **B23[4+4]**.

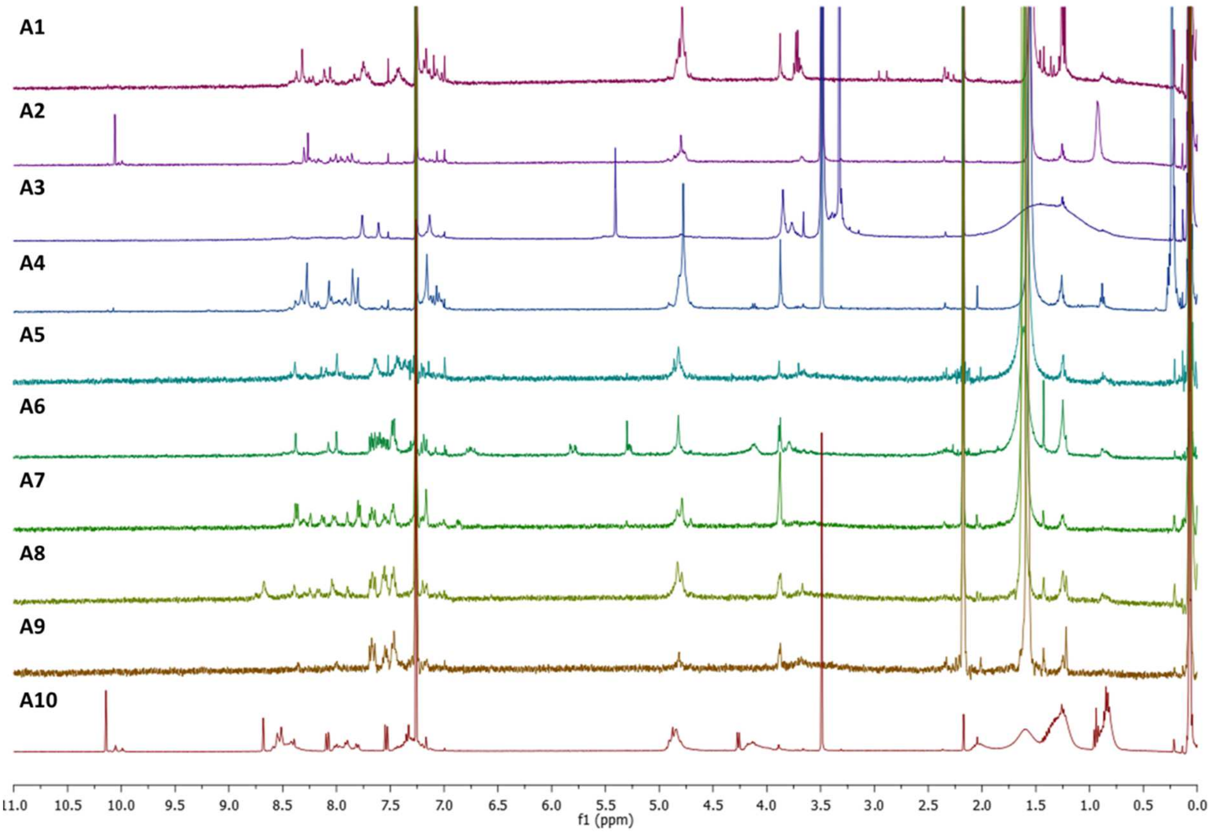

Supplementary Figure 50: Stacked ^{1}H NMR spectra ($CDCl_3$) of the crude reactions of triamine **A** with aldehydes **1–10** targeting [2+3] cages **A1–A10**, taken directly from the high-throughput screen (pre-isolation).

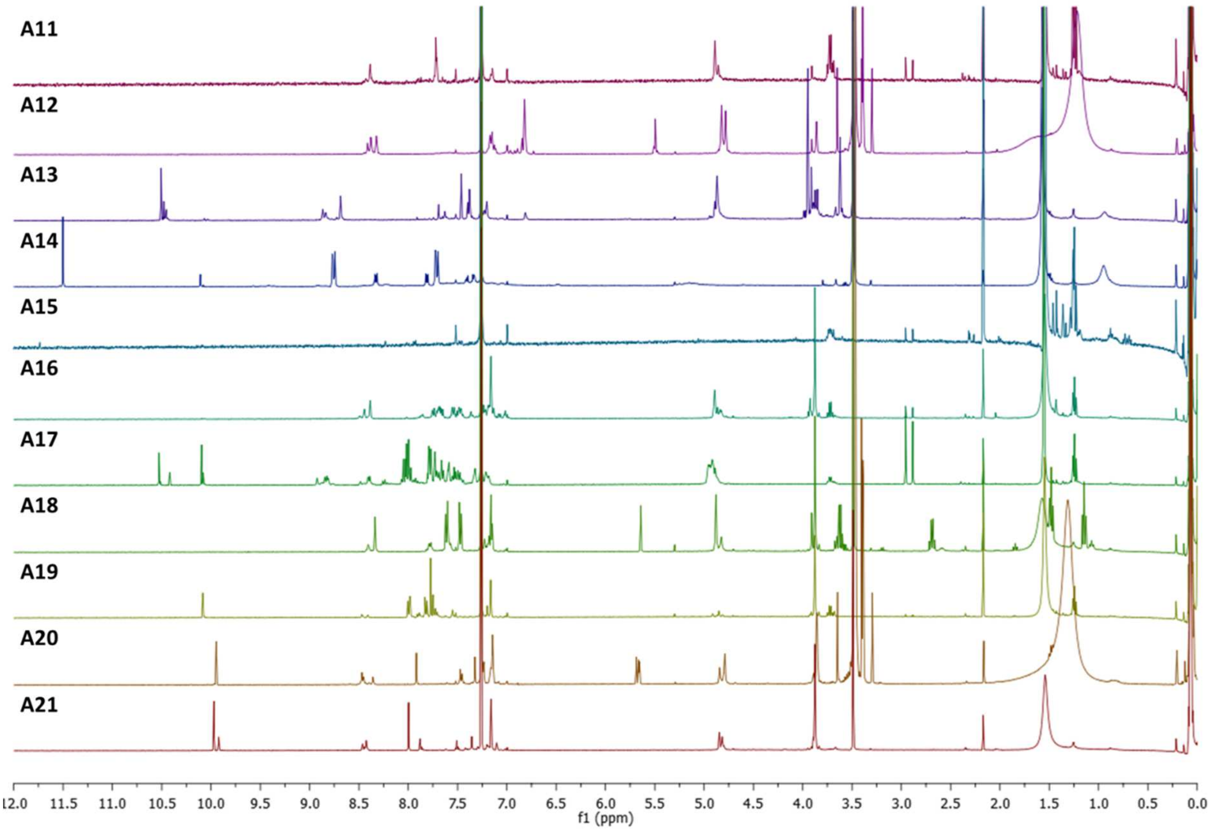

Supplementary Figure 51: Stacked ^{1}H NMR spectra ($CDCl_3$) of the crude reactions of triamine **A** with aldehydes **11–21** targeting [4+6] cages **A11–A21**, taken directly from the high-throughput screen (pre-isolation).

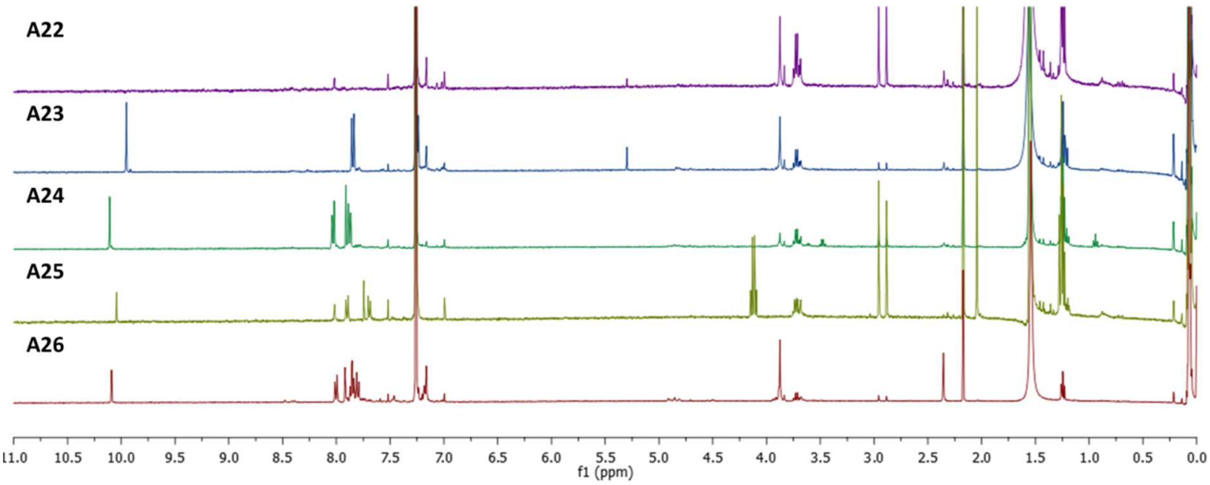

Supplementary Figure 52: Stacked ^{1}H NMR spectra ($CDCl_3$) of the crude reactions of triamine **A** with aldehydes **22–26** targeting [4+4] cages **A22–A26**, taken directly from the high-throughput screen (pre-isolation).

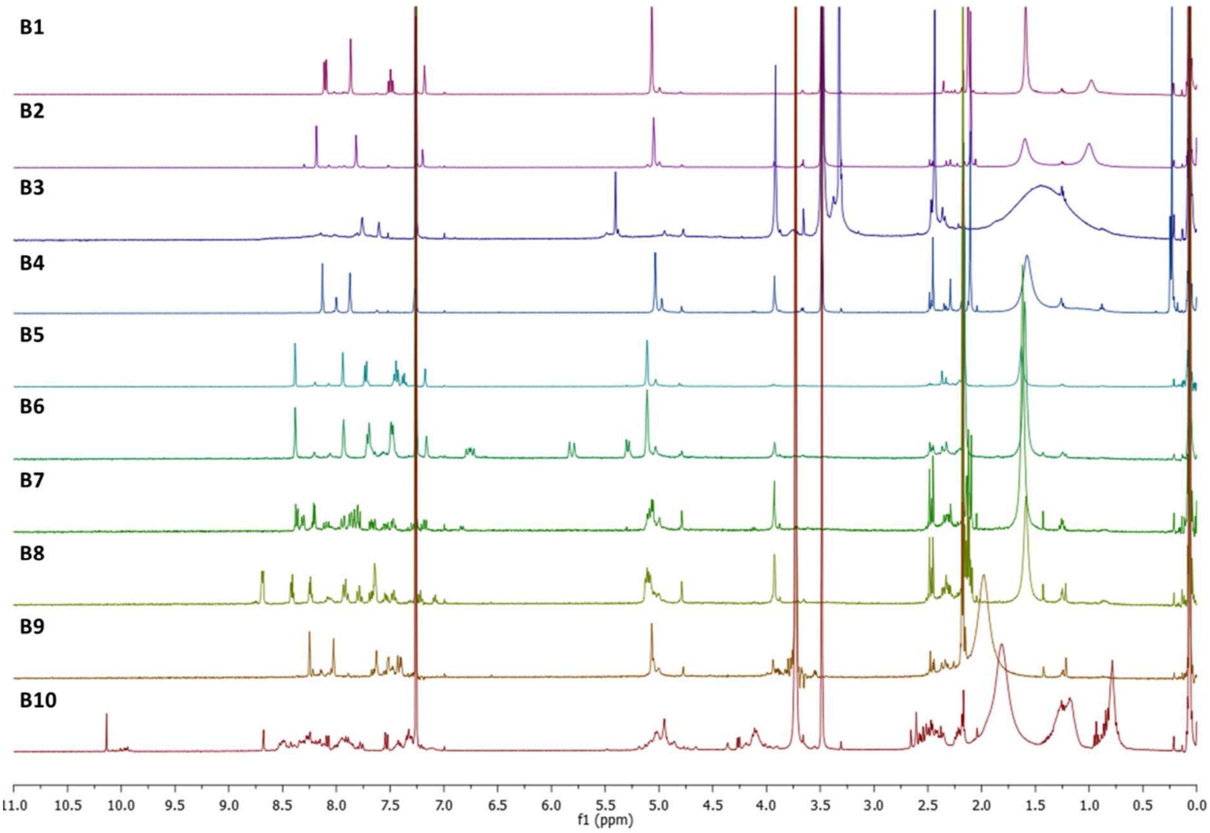

Supplementary Figure 53: Stacked ^{1}H NMR spectra ($CDCl_3$) of the crude reactions of triamine **B** with aldehydes **1–10** targeting [2+3] cages **B1–B10**, taken directly from the high-throughput screen (pre-isolation).

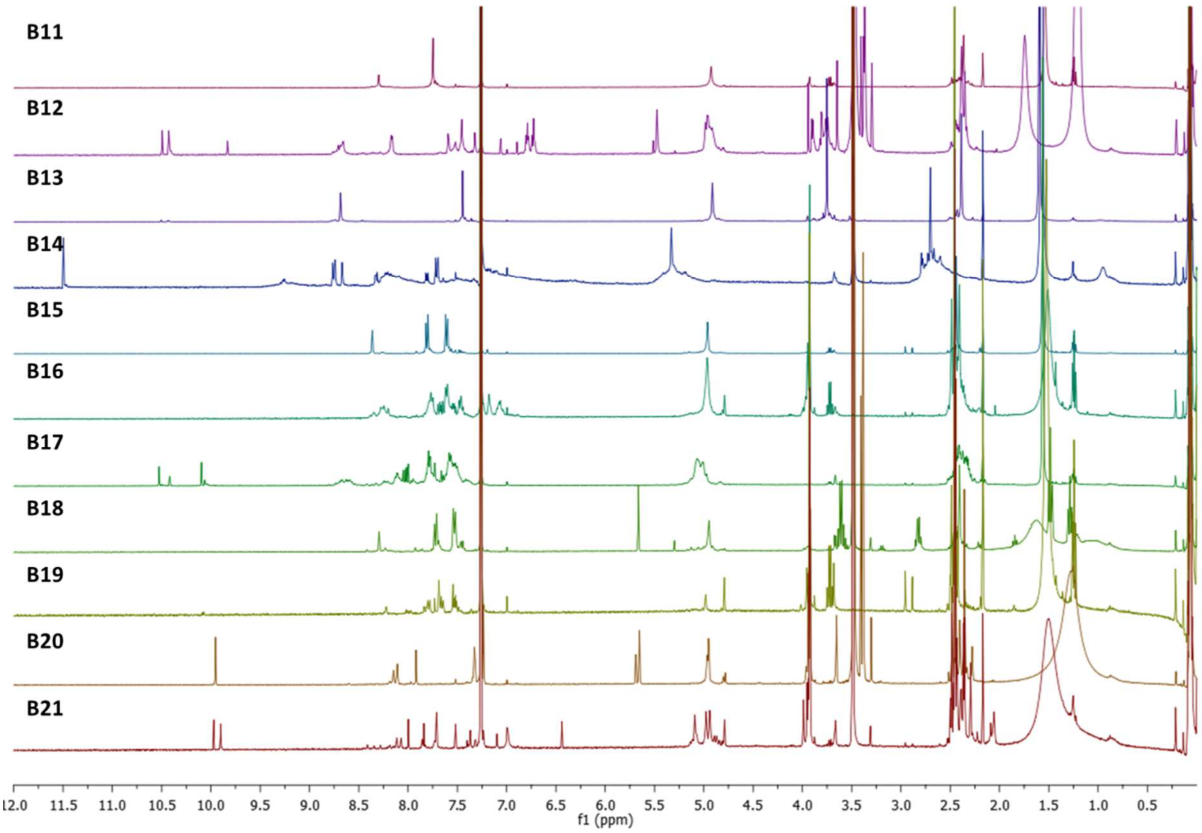

Supplementary Figure 54: Stacked ^{1}H NMR spectra ($CDCl_3$) of the crude reactions of triamine **B** with aldehydes **11–21** targeting [4+6] cages **B11–B21**, taken directly from the high-throughput screen (pre-isolation).

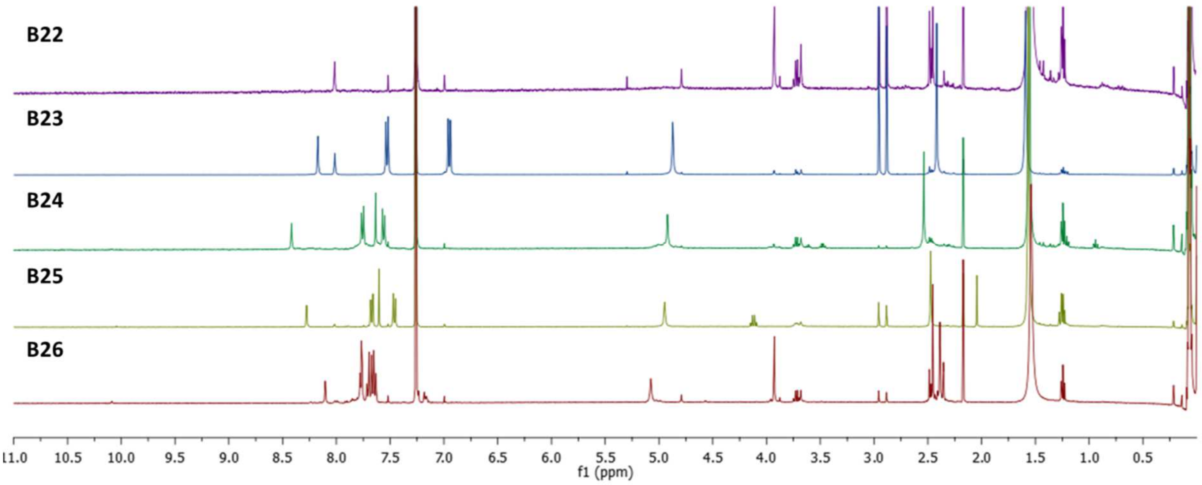

Supplementary Figure 55: Stacked ^{1}H NMR spectra ($CDCl_3$) of the crude reactions of triamine **B** with aldehydes **22–26** targeting [4+4] cages **B22–B26**, taken directly from the high-throughput screen (pre-isolation).

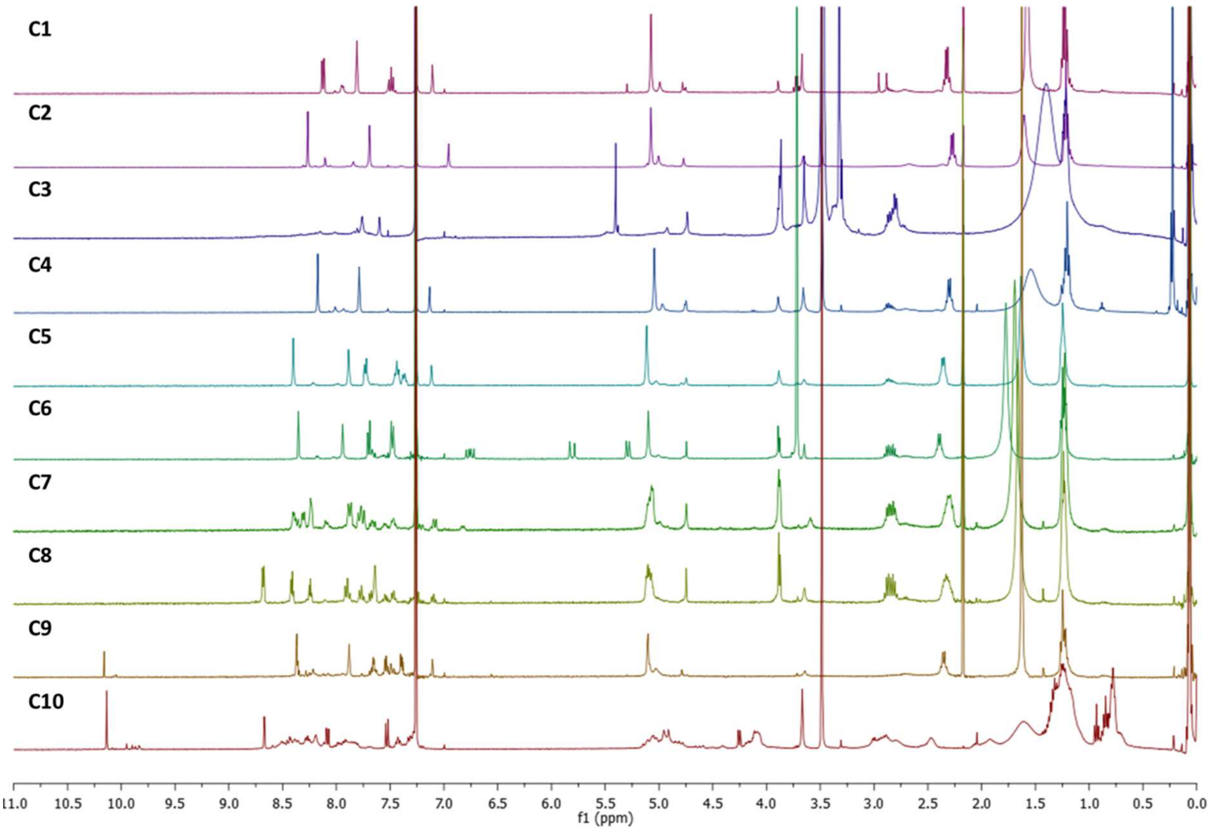

Supplementary Figure 56: Stacked ^{1}H NMR spectra ($CDCl_3$) of the crude reactions of triamine **C** with aldehydes **1–10** targeting [2+3] cages **C1–C10**, taken directly from the high-throughput screen (pre-isolation).

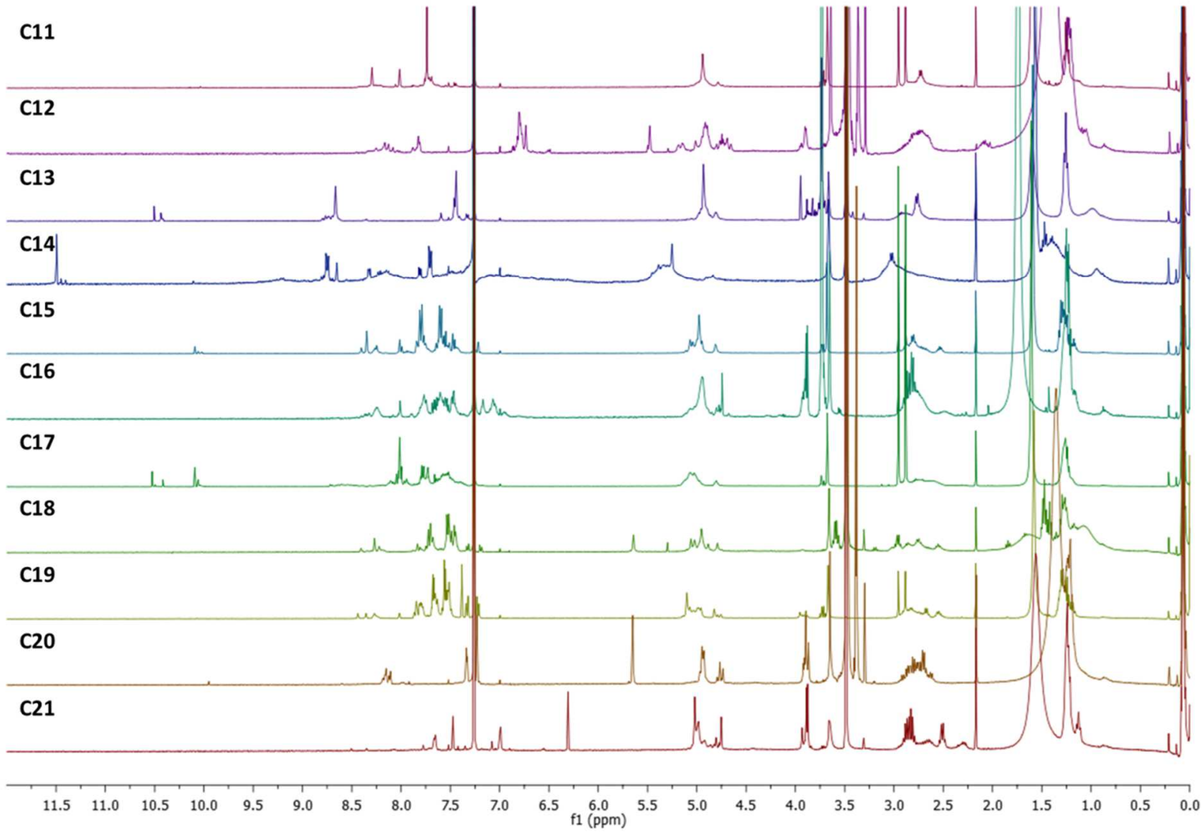

Supplementary Figure 57: Stacked ^{1}H NMR spectra ($CDCl_3$) of the crude reactions of triamine **C** with aldehydes **11–21** targeting [4+6] cages **C11–C21**, taken directly from the high-throughput screen (pre-isolation).

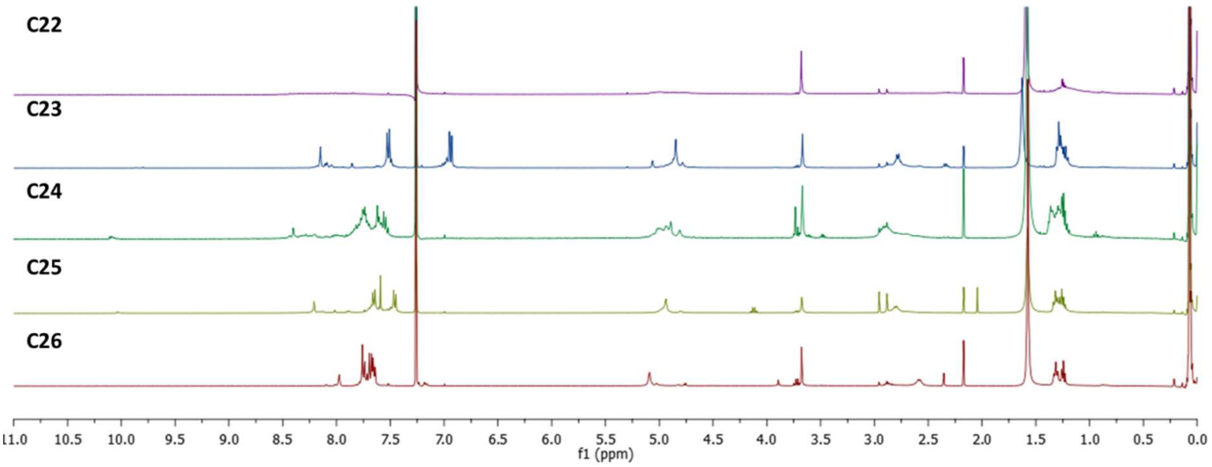

Supplementary Figure 58: Stacked ^{1}H NMR spectra ($CDCl_3$) of the crude reactions of triamine **C** with aldehydes **22–26** targeting [4+4] cages **C22–C26**, taken directly from the high-throughput screen (pre-isolation).

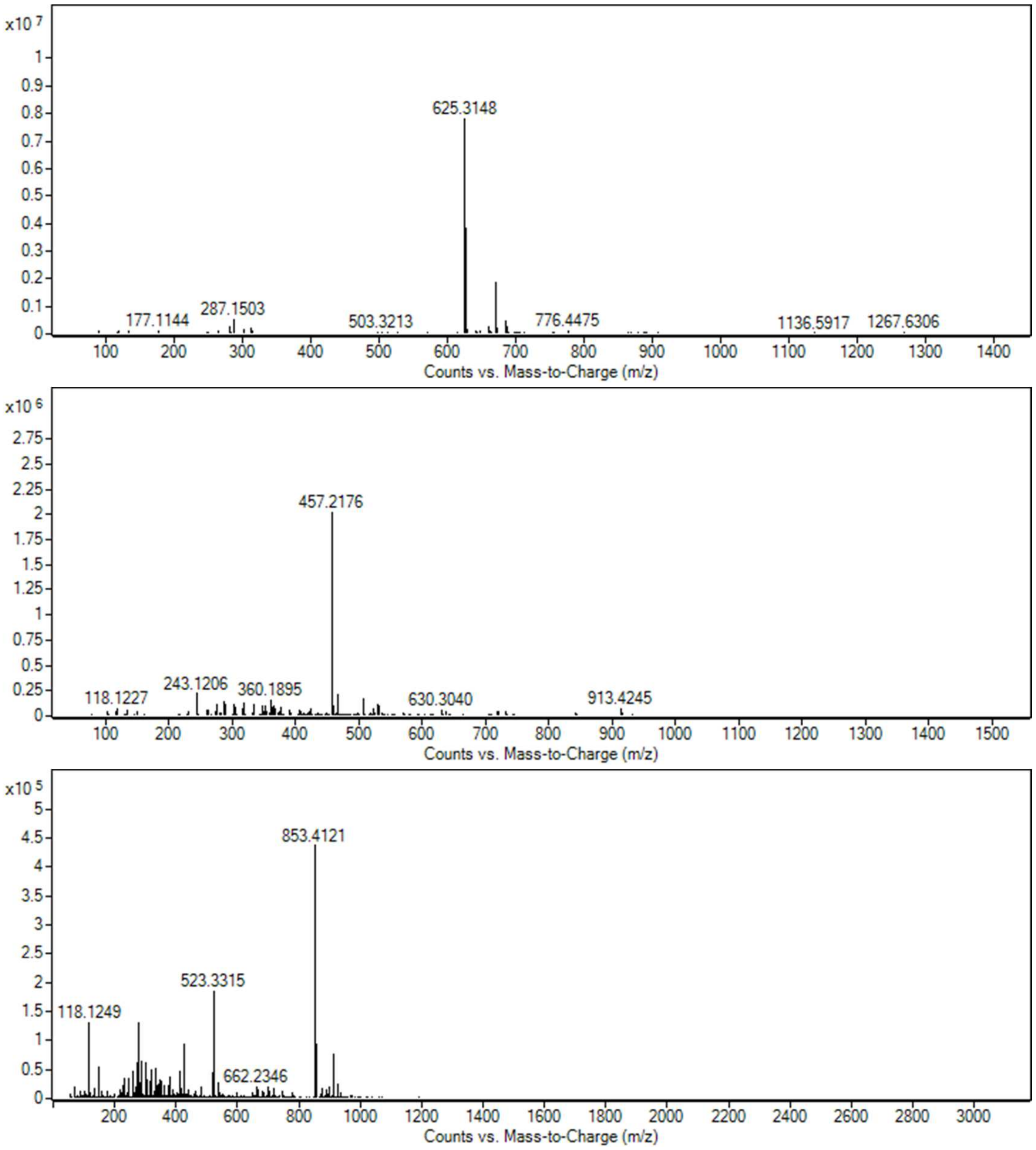

Supplementary Figure 59: HRMS spectra for crude reactions taken directly from the high-throughput screen (pre-isolation): upper = **A1**, indicating clear formation of a [2+3] cage — $[M+H]^+$ 625.3148; middle = **A4**, indicating clear formation of a [2+3] cage — $[M+2H]^{2+}$ 457.2176; lower = **A5**, indicating clear formation of a [2+3] cage — $[M+H]^+$ 853.4121.

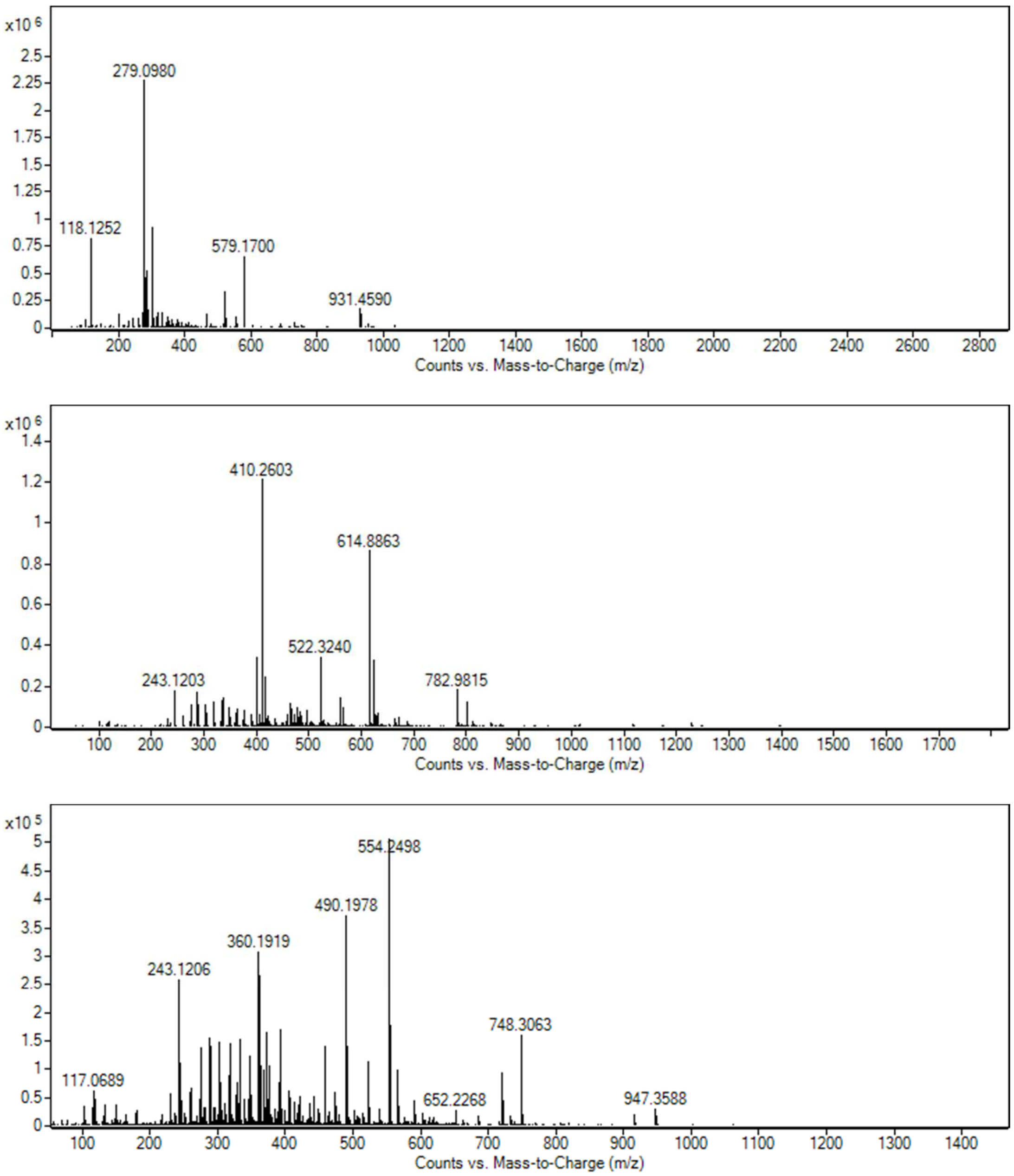

Supplementary Figure 60: HRMS spectra for crude reactions taken directly from the high-throughput screen (pre-isolation): upper = **A6**, indicating some formation of a [2+3] cage — $[M+H]^+$ 931.4590; middle = **A10**, indicating formation of a [2+3] cage — $[M+2H]^{2+}$ 614.886 and $[M+3H]^{3+}$ 410.2603; lower = **A12**, indicating some formation of a [2+3] cage — $[M+H]^+$ 721.2757.

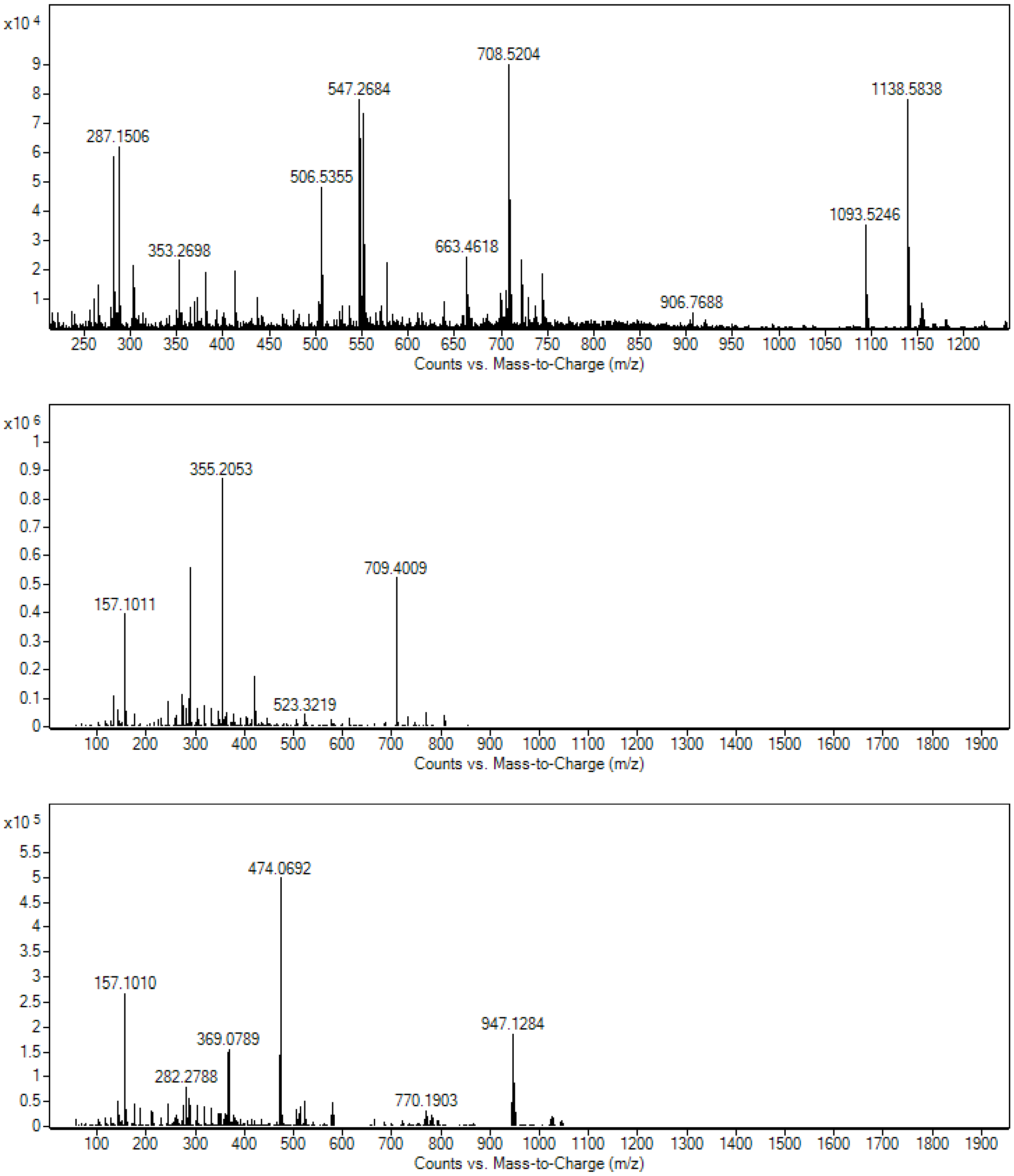

Supplementary Figure 61: HRMS spectra for crude reactions taken directly from the high-throughput screen (pre-isolation): upper = **A22**, indicating some formation of a [4+4] cage — $[M+H]^+$ 1093.5246 and $[M+2H]^{2+}$ 547.2684; middle = **B1**, indicating clear formation of a [2+3] cage — $[M+H]^+$ 709.4009 and $[M+2H]^{2+}$ 355.2053; lower = **B2**, indicating clear formation of a [2+3] cage — $[M+H]^+$ 947.1284 and $[M+2H]^{2+}$ 474.0692.

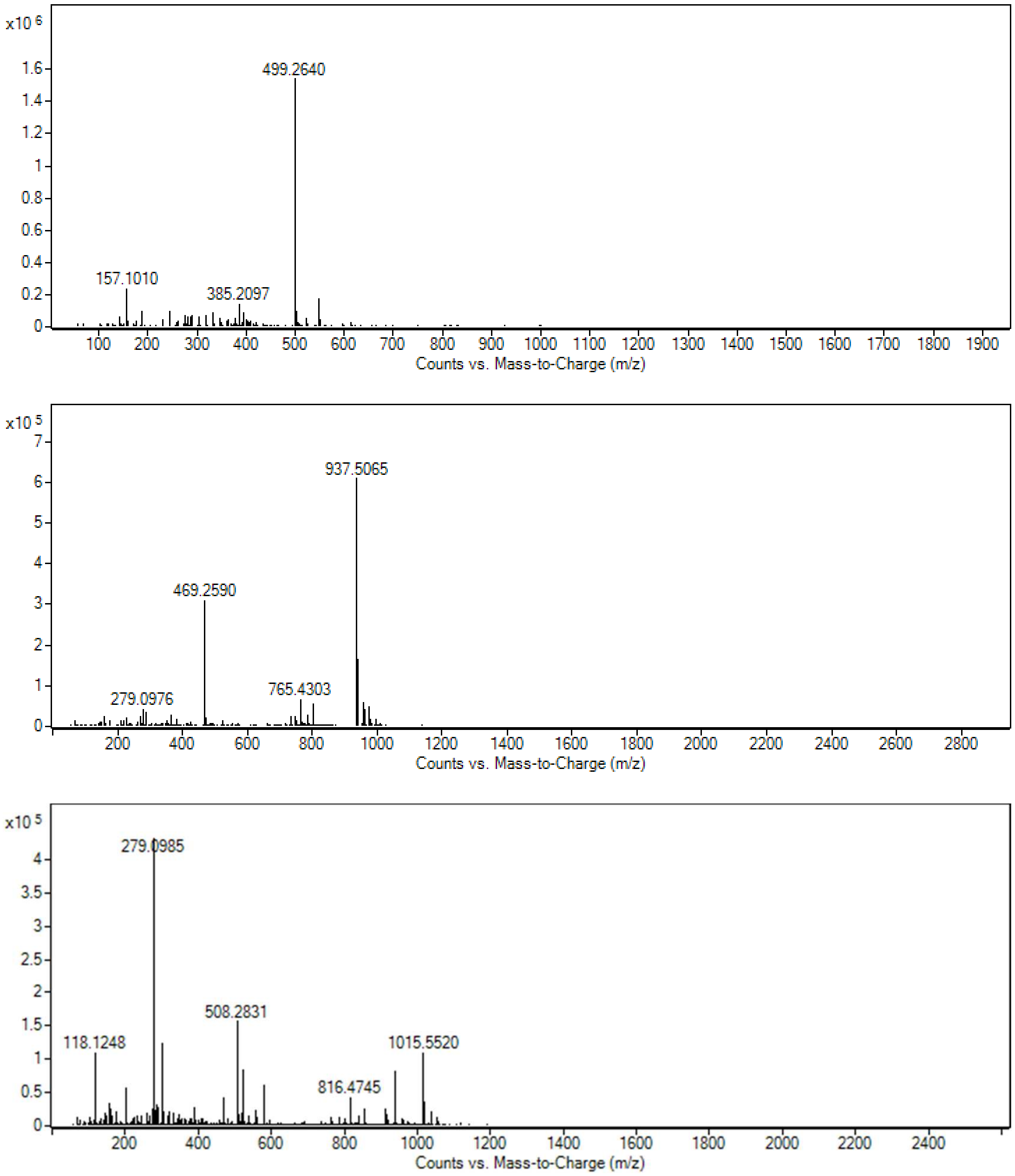

Supplementary Figure 62: HRMS spectra for crude reactions taken directly from the high-throughput screen (pre-isolation): upper = **B4**, indicating clear formation of a [2+3] cage — $[M+2H]^{2+}$ 499.2640; middle = **B5**, indicating clear formation of a [2+3] cage — $[M+H]^{+}$ 937.5065 and $[M+2H]^{2+}$ 469.2590; lower = **B6**, indicating formation of a [2+3] cage — $[M+H]^{+}$ 1015.5520 and $[M+2H]^{2+}$ 508.2831.

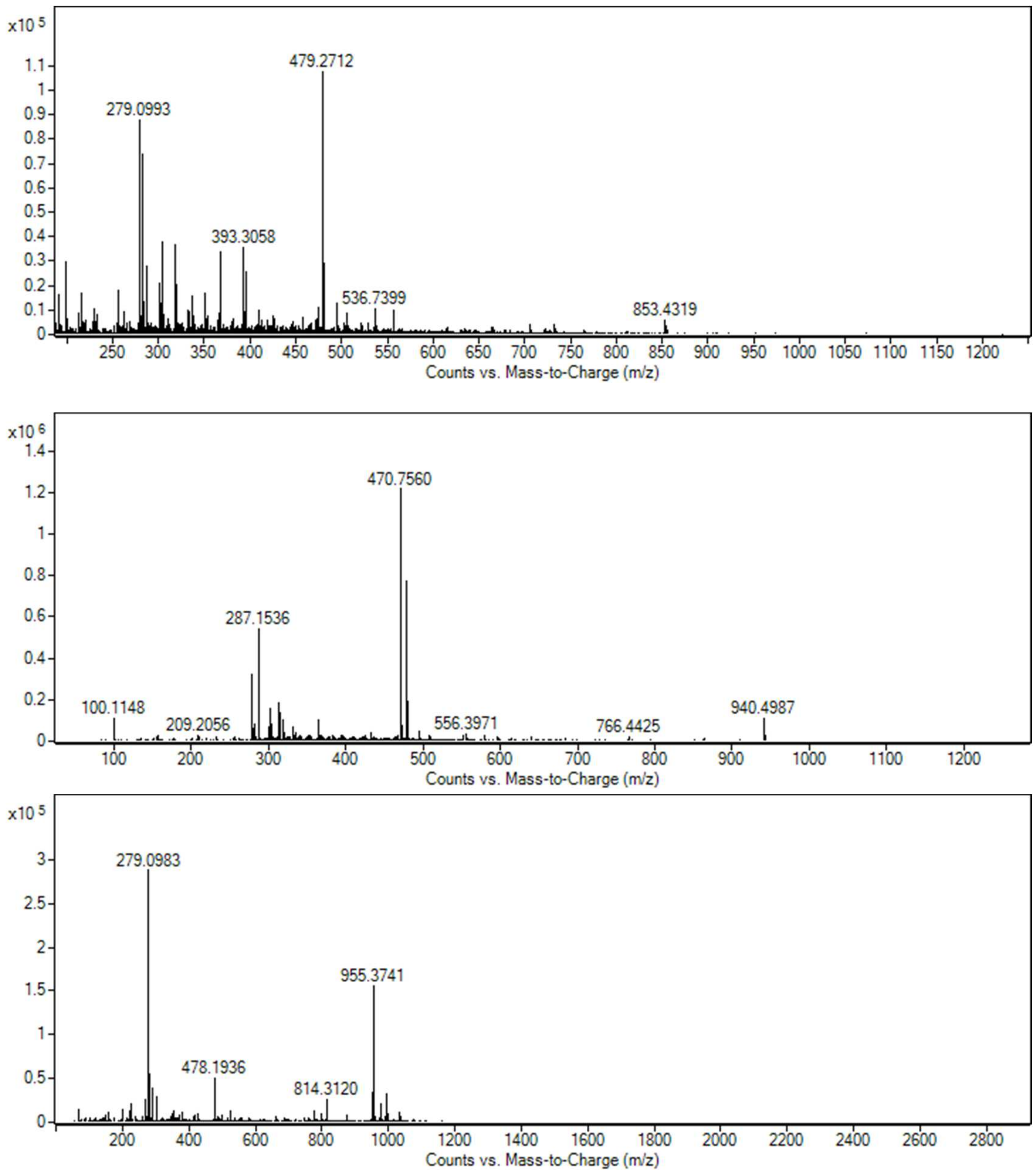

Supplementary Figure 63: HRMS spectra for crude reactions taken directly from the high-throughput screen (pre-isolation): upper = **B7**, indicating some formation of a [2+3] cage — $[M+2H]^{2+}$ 536.7399; middle = **B8**, indicating clear formation of a [2+3] cage — $[M+H]^{+}$ 940.4987 and $[M+2H]^{2+}$ 470.7560; lower = **B9**, indicating clear formation of a [2+3] cage — $[M+H]^{+}$ 955.3741 and $[M+2H]^{2+}$ 478.1936.

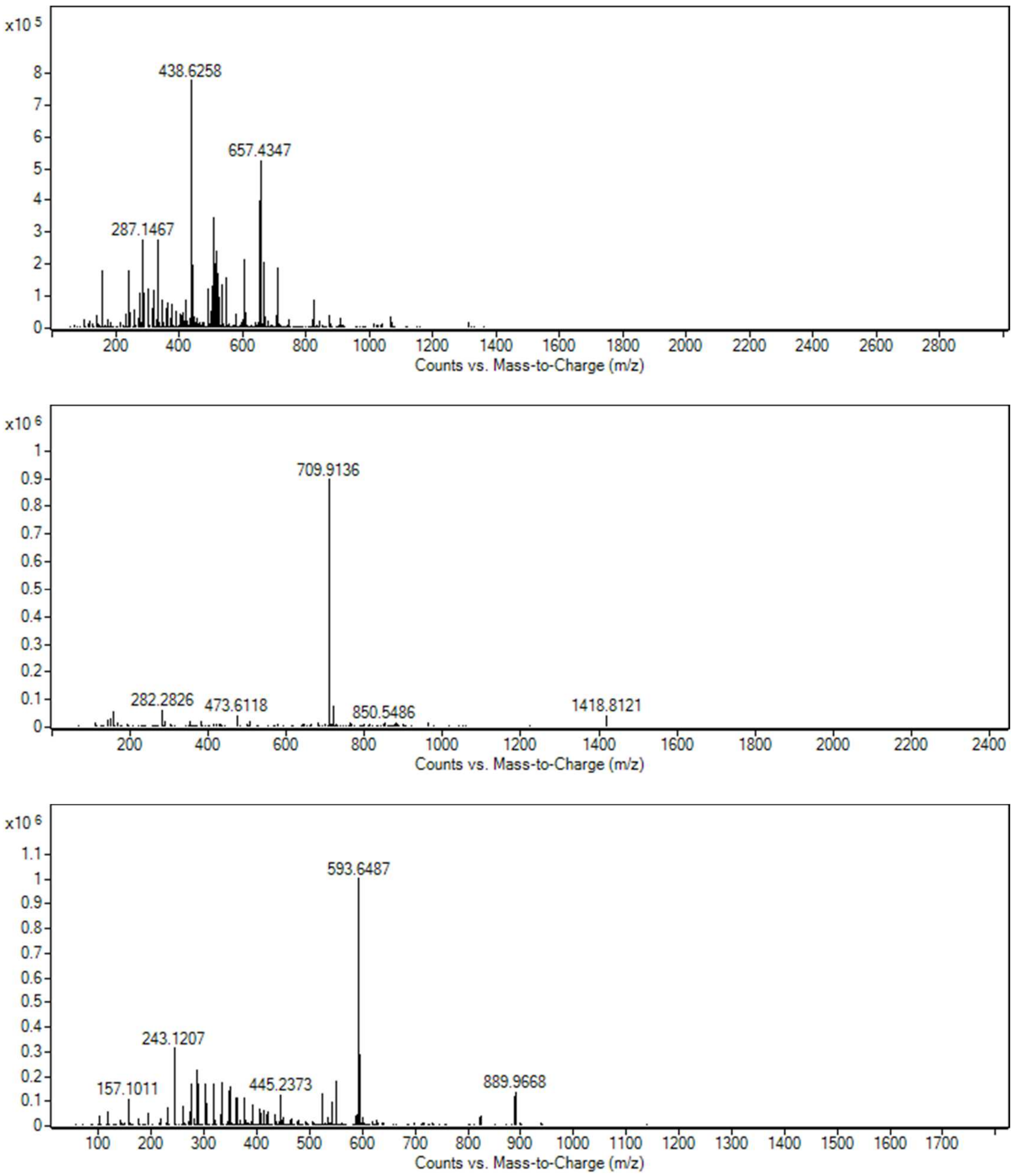

Supplementary Figure 64: HRMS spectra for crude reactions taken directly from the high-throughput screen (pre-isolation): upper = **B10**, indicating some formation of a [2+3] cage — $[M+2H]^{2+}$ 656.9329; middle = **B11**, indicating clear formation of a [4+6] cage — $[M+H]^{+}$ 1418.8121 and $[M+2H]^{2+}$ 709.9136; lower = **B13**, indicating clear formation of a [4+6] cage — $[M+2H]^{2+}$ 889.9668 and $[M+3H]^{3+}$ 593.6487.

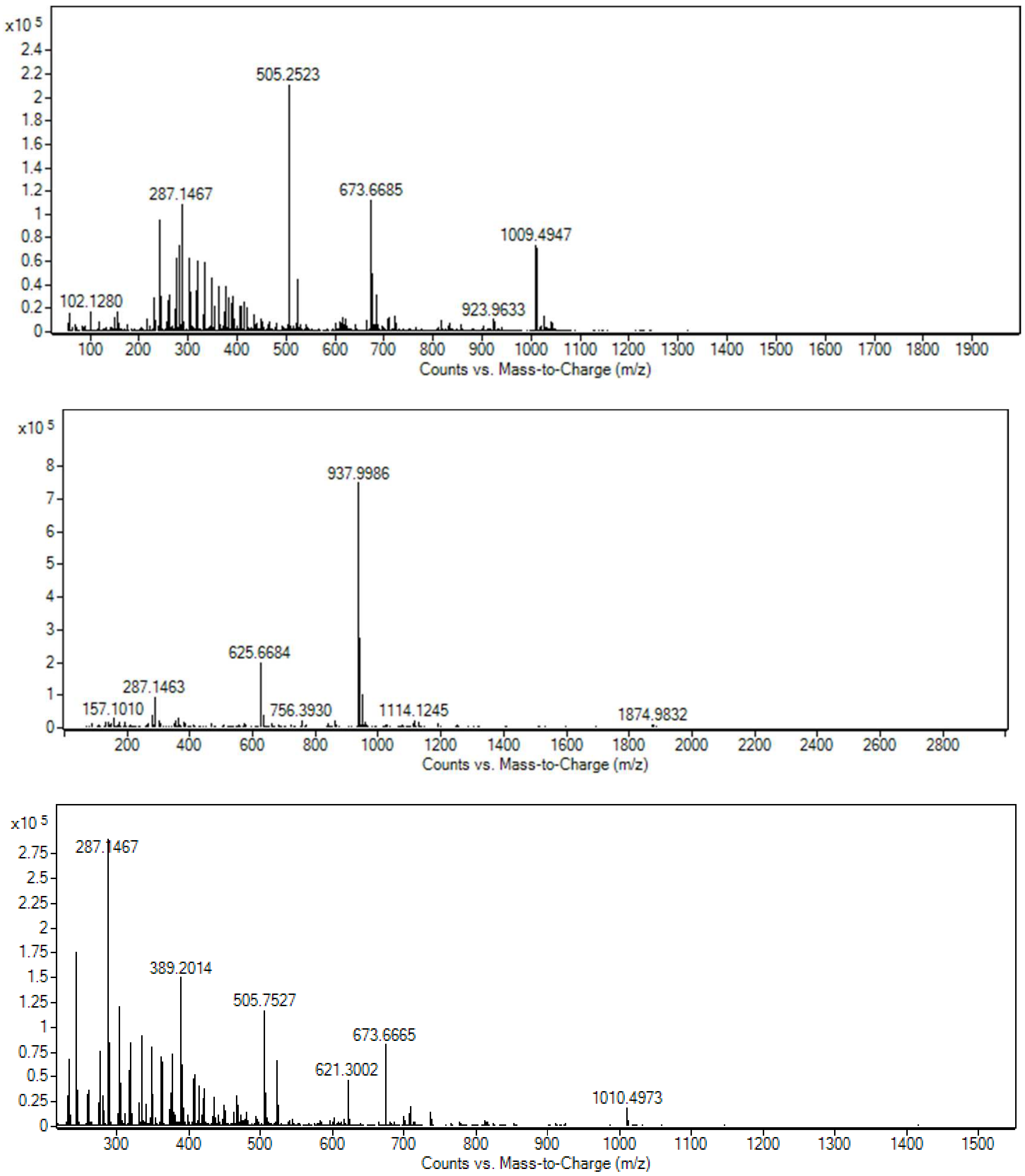

Supplementary Figure 65: HRMS spectra for crude reactions taken directly from the high-throughput screen (pre-isolation): upper = **B14**, indicating clear formation of a mixture of [2+3] and [4+6] cages — overlapping isotope patterns for **B14**$_{\mathbf{[2+3]}}$ $[M+H]^+$ and **B14**$_{\mathbf{[4+6]}}$ $[M+2H]^{2+}$ visible at mass ion 1009.4947, but $[M+3H]^{3+}$ 673.6685 consistent with **B14**$_{\mathbf{[4+6]}}$, and $[M+2H]^{2+}$ 505.2523 consistent with **B14**$_{\mathbf{[2+3]}}$; middle = **B15**, indicating clear formation of a [4+6] cage — $[M+2H]^{2+}$ 937.9986 and $[M+3H]^{3+}$ 625.6684; lower = **B18**, indicating formation of a [4+6] cage — $[M+2H]^{2+}$ 1009.4926, $[M+3H]^{3+}$ 673.6665 and $[M+4H]^{4+}$ 505.2523.

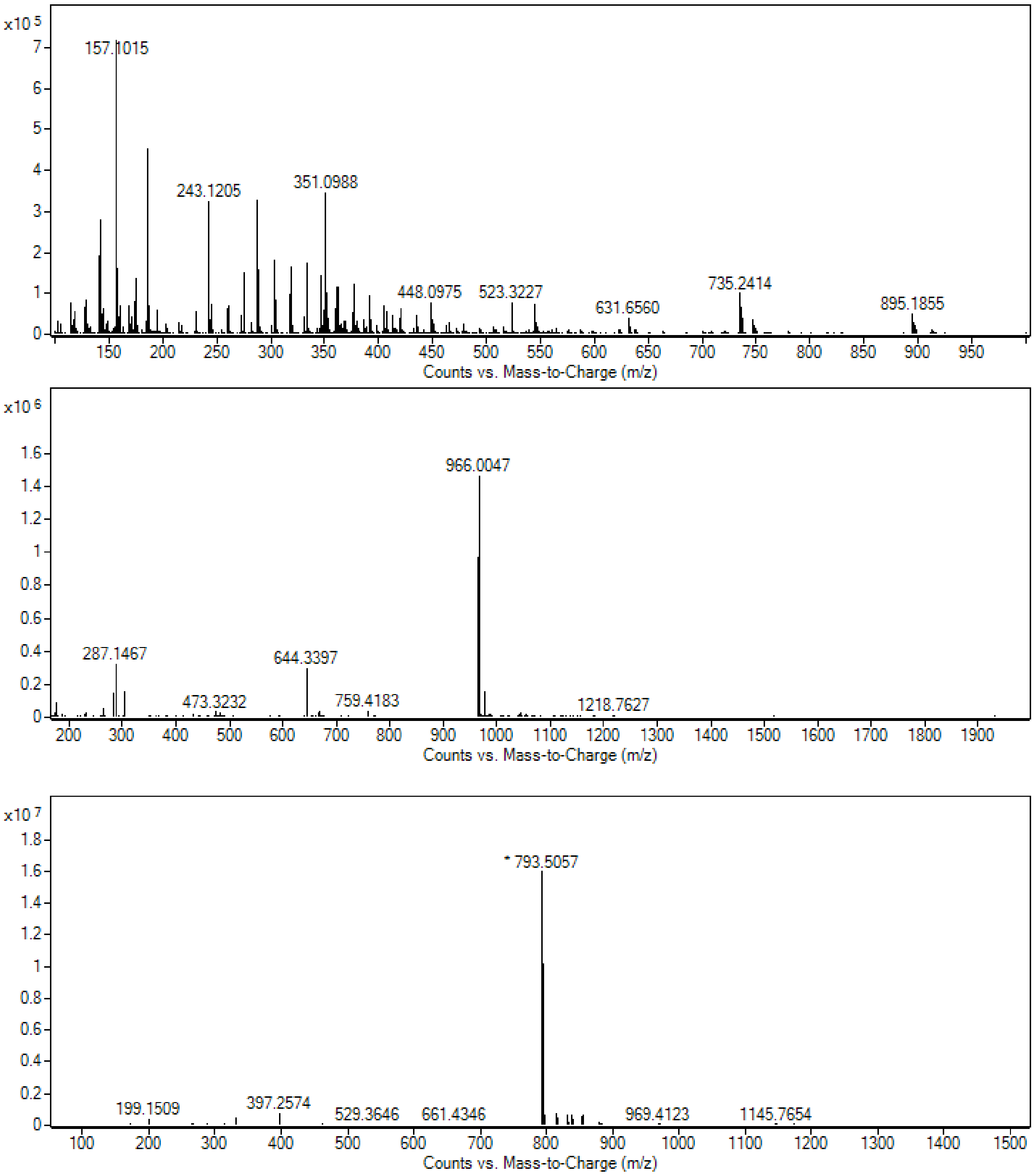

Supplementary Figure 66: HRMS spectra for crude reactions taken directly from the high-throughput screen (pre-isolation): upper = **B21**, indicating some formation of a [2+3] cage — $[M+H]^+$ 895.1855 and $[M+2H]^{2+}$ 448.0975; middle = **B23**, indicating clear formation of a [4+4] cage — $[M+2H]^{2+}$ 966.0047 and $[M+3H]^{3+}$ 644.3397; lower = **C1**, indicating clear formation of a [2+3] cage — $[M+H]^+$ 793.5057.

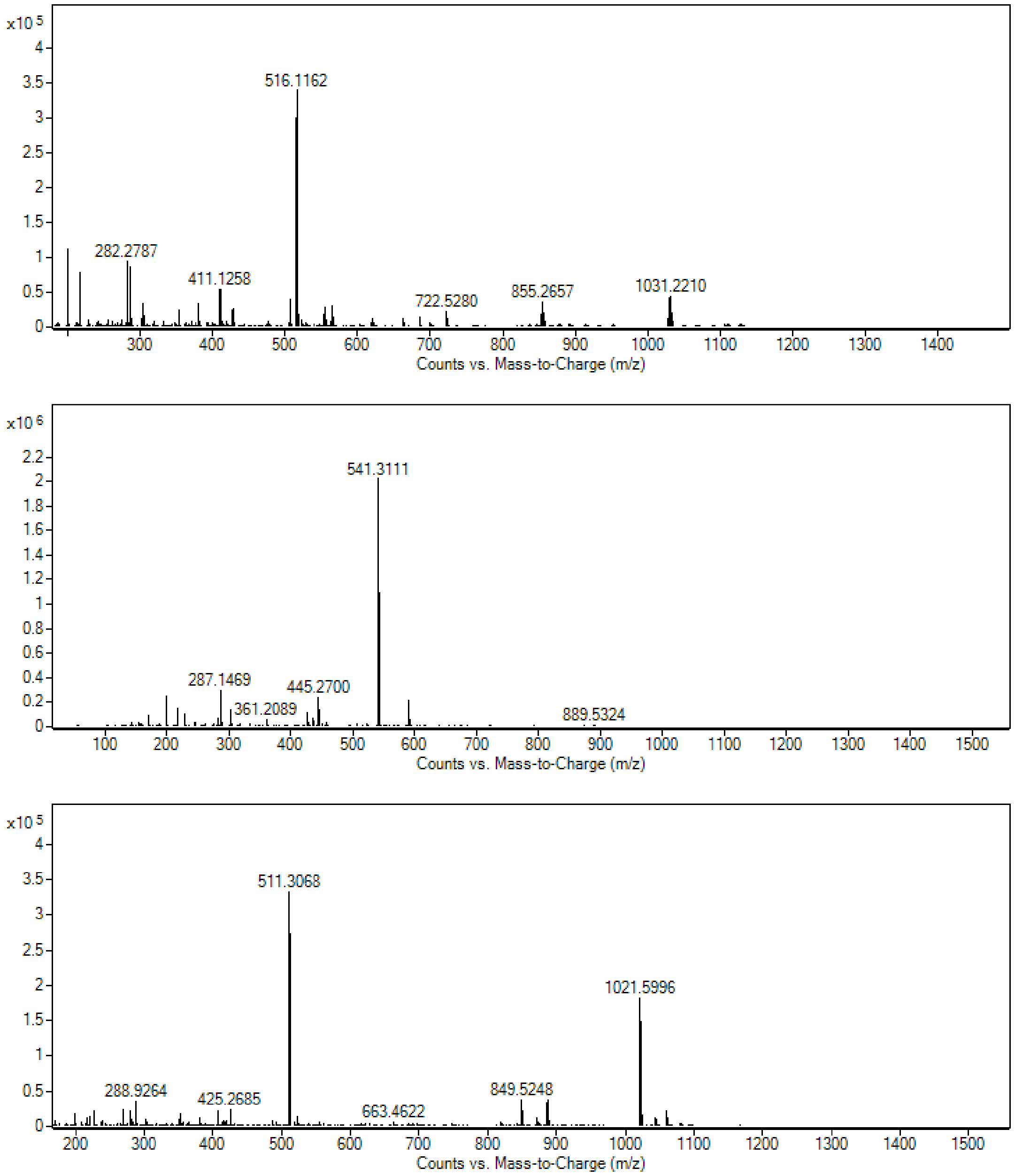

Supplementary Figure 67: HRMS spectra for crude reactions taken directly from the high-throughput screen (pre-isolation): upper = **C2**, indicating clear formation of a [2+3] cage — $[M+H]^+$ 1031.2210 and $[M+2H]^{2+}$ 516.1162; middle = **C4**, indicating clear formation of a [2+3] cage — $[M+2H]^{2+}$ 541.3111; lower = **C5**, indicating clear formation of a [2+3] cage — $[M+H]^+$ 1021.5996 and $[M+2H]^{2+}$ 511.3068.

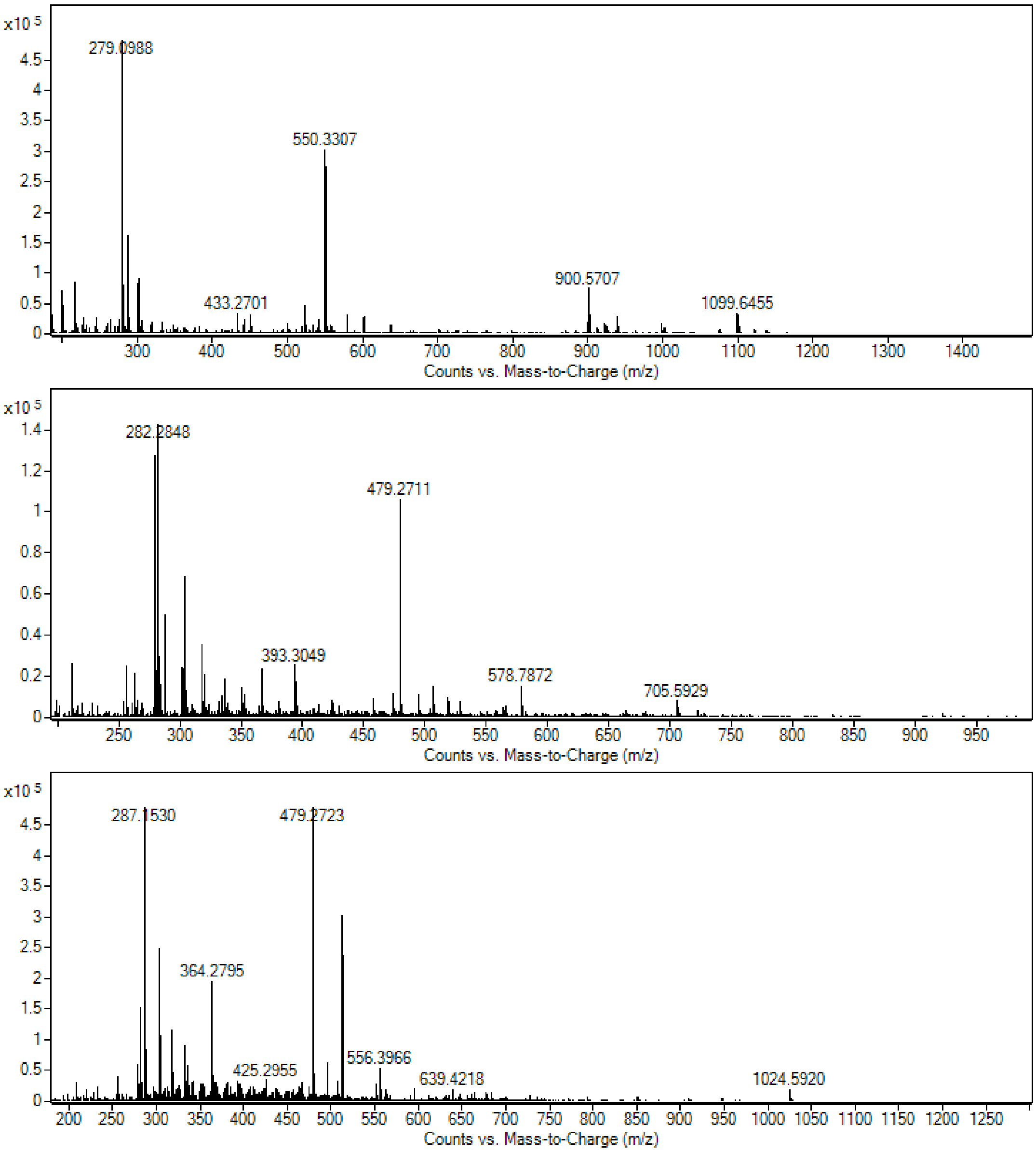

Supplementary Figure 68: HRMS spectra for crude reactions taken directly from the high-throughput screen (pre-isolation): upper = **C6**, indicating clear formation of a [2+3] cage — $[M+H]^+$ 1099.6455 and $[M+2H]^{2+}$ 550.3307; middle = **C7**, indicating some formation of a [2+3] cage — $[M+2H]^{2+}$ 578.7872; lower = **C8**, indicating formation of a [2+3] cage — $[M+H]^+$ 1024.5920 and $[M+2H]^{2+}$ 512.8024.

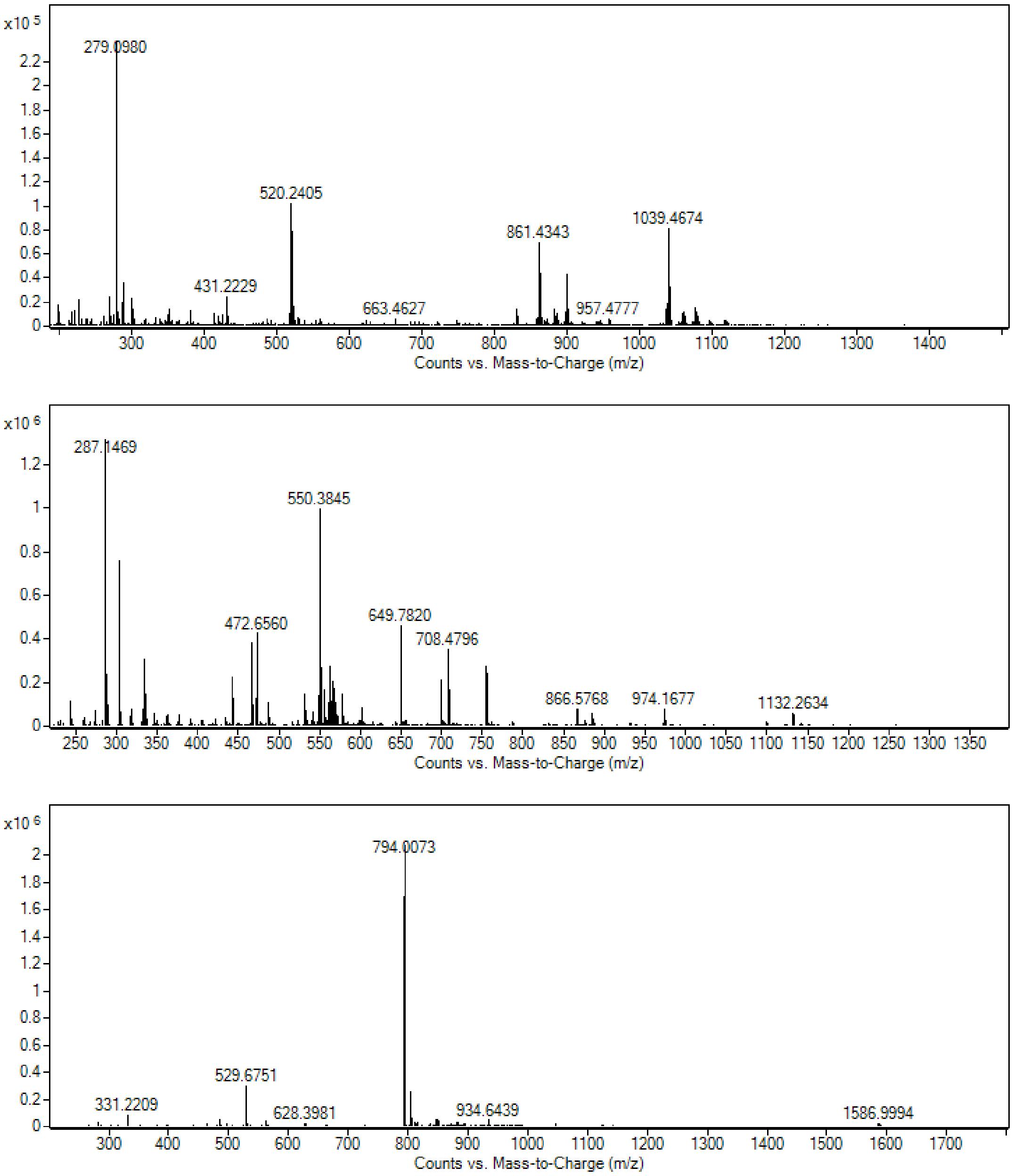

Supplementary Figure 69: HRMS spectra for crude reactions taken directly from the high-throughput screen (pre-isolation): upper = **C9**, indicating clear formation of a [2+3] cage — $[M+H]^+$ 1039.4674 and $[M+2H]^{2+}$ 520.2405; middle = **C10**, indicating some formation of a [2+3] cage — $[M+2H]^{2+}$ 699.4800; lower = **C11**, indicating clear formation of a [4+6] cage — $[M+H]^+$ 1586.9994, $[M+2H]^{2+}$ 794.0073 and $[M+3H]^{3+}$ 529.6751.

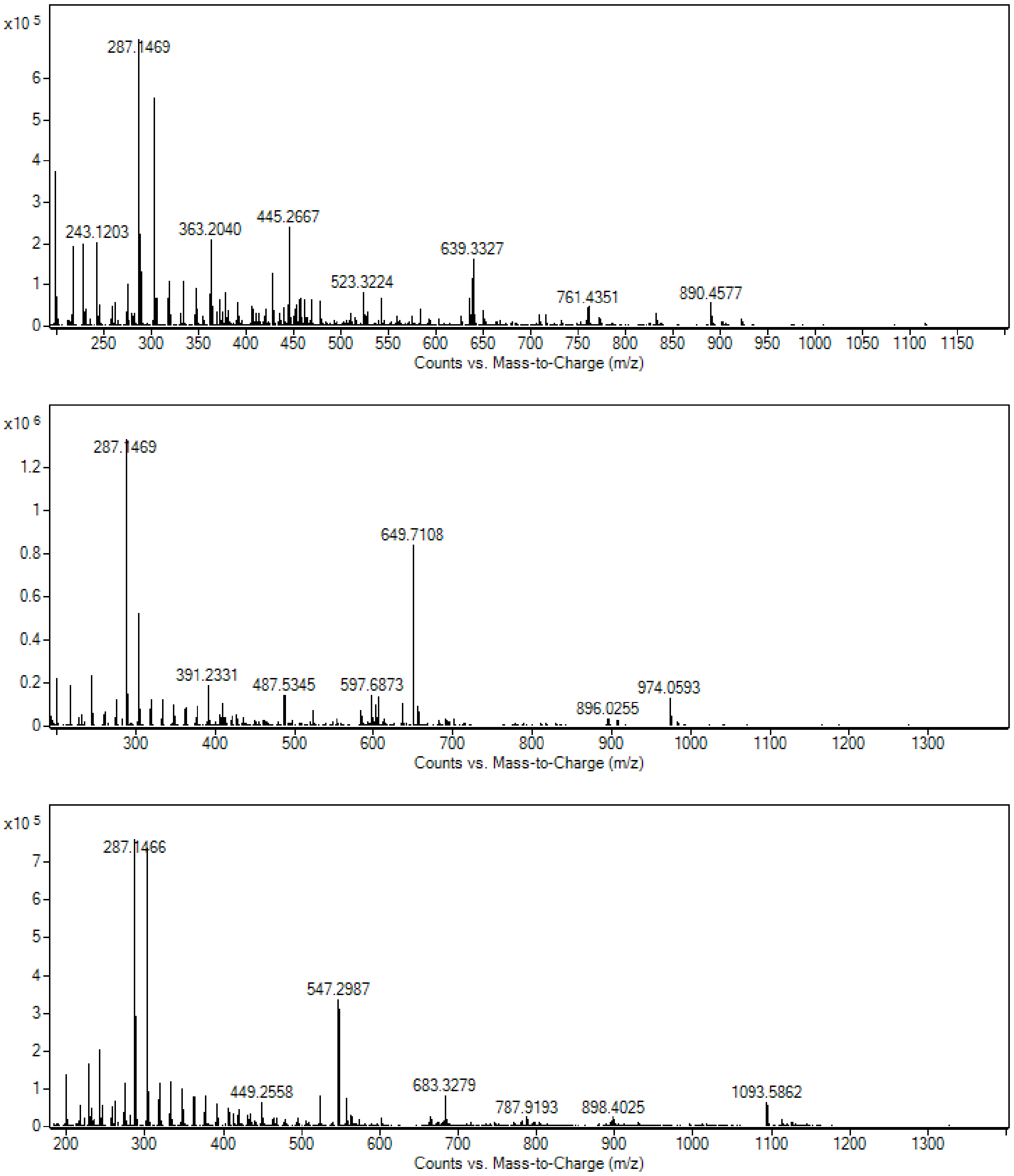

Supplementary Figure 70: HRMS spectra for crude reactions taken directly from the high-throughput screen (pre-isolation): upper = **C12**, indicating some formation of a [2+3] cage — $[M+H]^{+}$ 890.4577; middle = **C13**, indicating clear formation of a [4+6] cage — $[M+2H]^{2+}$ 974.0593 and $[M+3H]^{3+}$ 649.7108; lower = **C14**, indicating clear formation of a [2+3] cage — $[M+H]^{+}$ 1093.5862 and $[M+2H]^{2+}$ 547.2987.

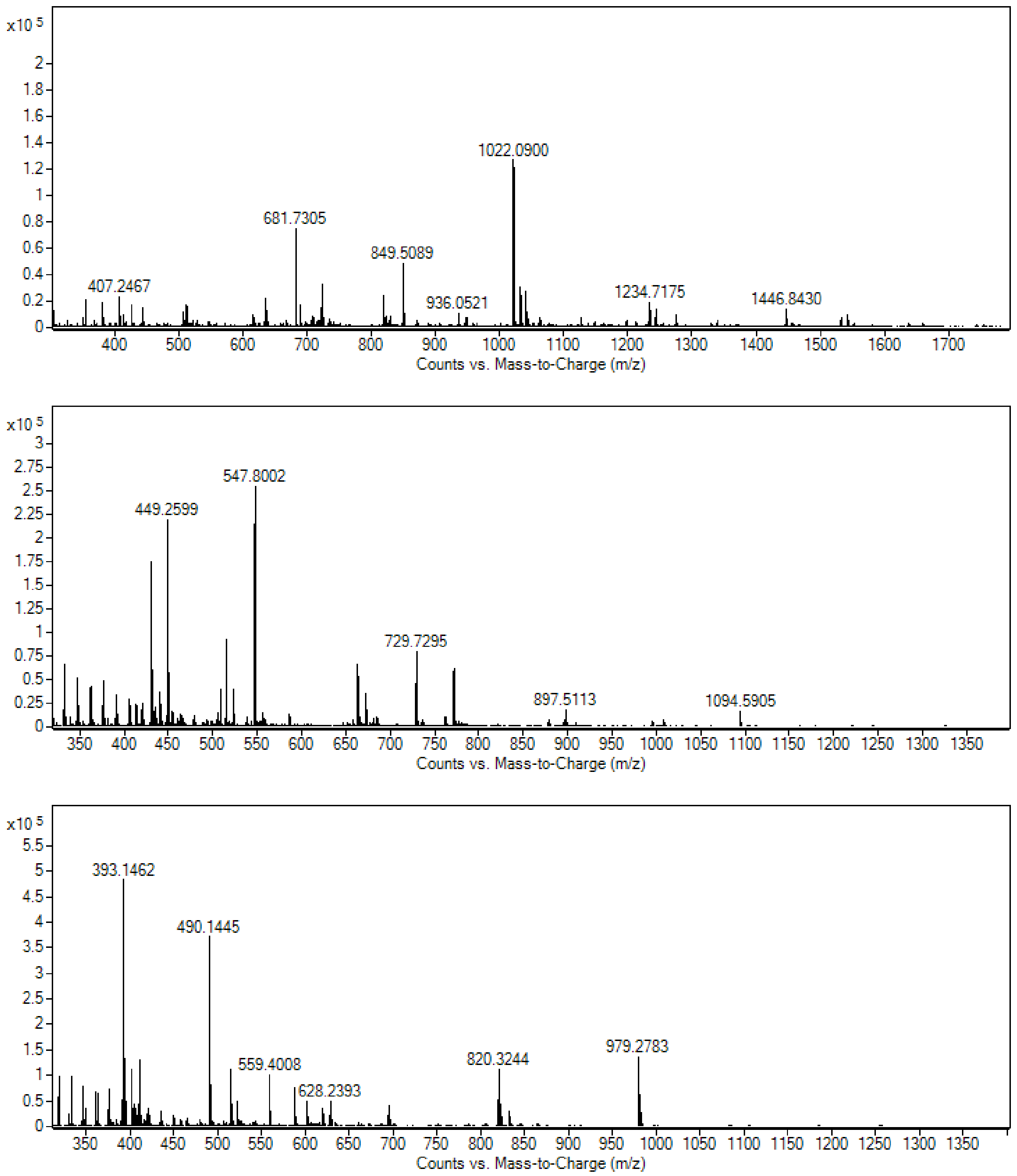

Supplementary Figure 71: HRMS spectra for crude reactions taken directly from the high-throughput screen (pre-isolation): upper = **C15**, indicating formation of a [4+6] cage — $[M+2H]^{2+}$ 1022.0900; middle = **C18**, indicating formation of a [4+6] cage — $[M+3H]^{3+}$ 729.7295 and $[M+4H]^{4+}$ 547.5497; lower = **C21**, indicating formation of a [2+3] cage — $[M+H]^{+}$ 979.2783 and $[M+2H]^{2+}$ 490.1445.

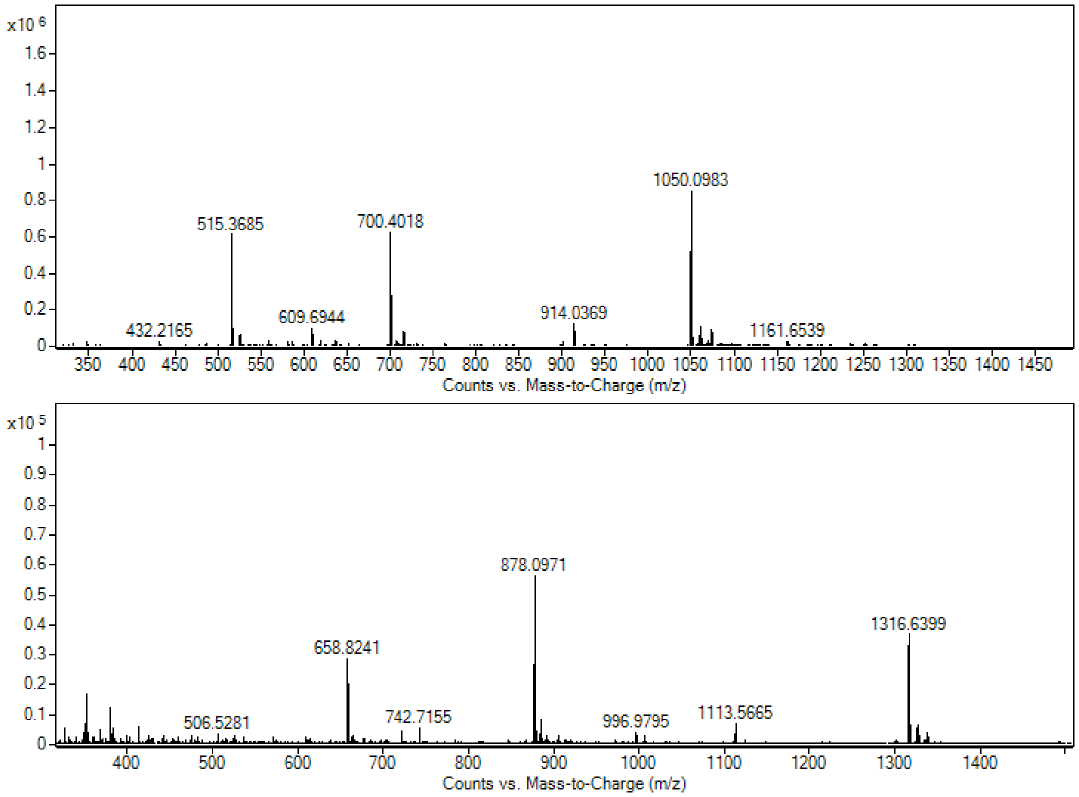

Supplementary Figure 72: HRMS spectra for crude reactions taken directly from the high-throughput screen (pre-isolation): upper = **C23**, indicating clear formation of a [4+4] cage — $[M+2H]^{2+}$ 1050.0983 and $[M+3H]^{3+}$ 700.4018; lower = **C25**, indicating clear formation of a [4+4] cage — $[M+2H]^{2+}$ 1316.6399, $[M+3H]^{3+}$ 878.0971 and $[M+4H]^{4+}$ 658.8241.

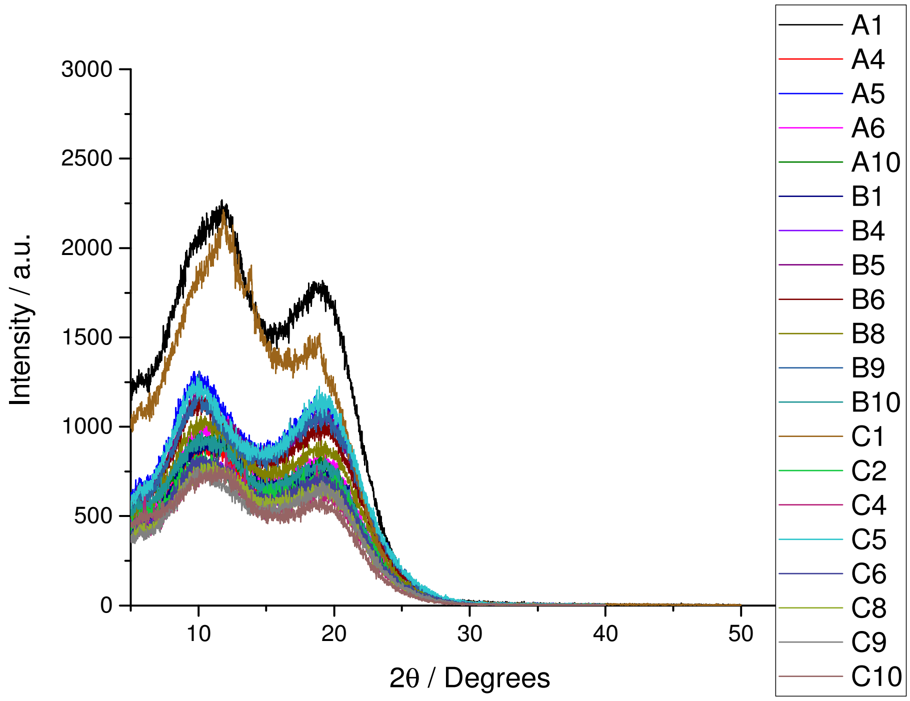

Supplementary Figure 73: Overlaid PXRDs of the **Tri^{2}Di^3** crude samples (post-isolation) indicating that the non-purified [2+3] cages are amorphous in nature.

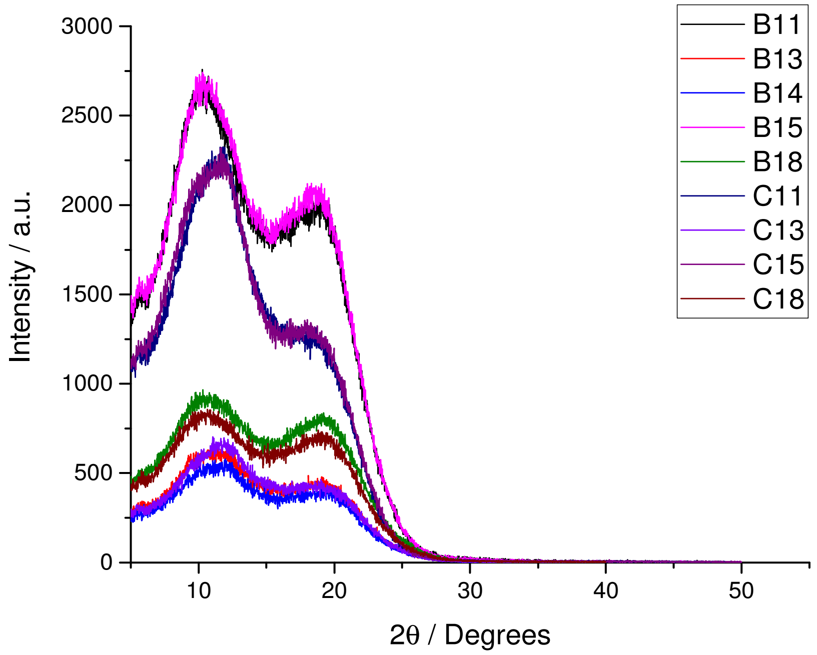

Supplementary Figure 74: Overlaid PXRDs of the **Tri^{4}Di^6** crude samples (post-isolation) indicating that the non-purified [4+6] cages are amorphous in nature.

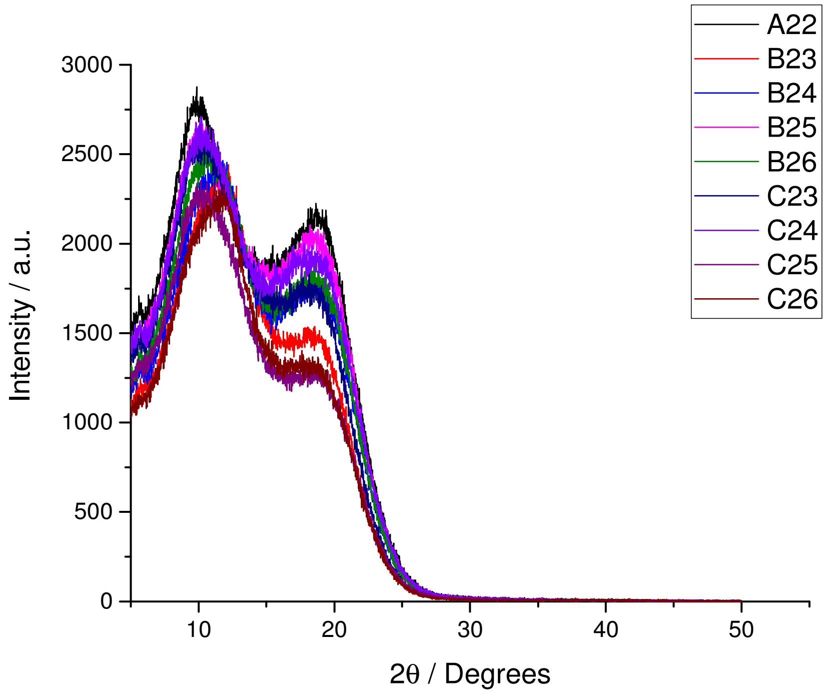

Supplementary Figure 75: Overlaid PXRDs of the **Tri^{4}Tri^4** crude samples (post-isolation) indicating that the non-purified [4+4] cages are amorphous in nature.

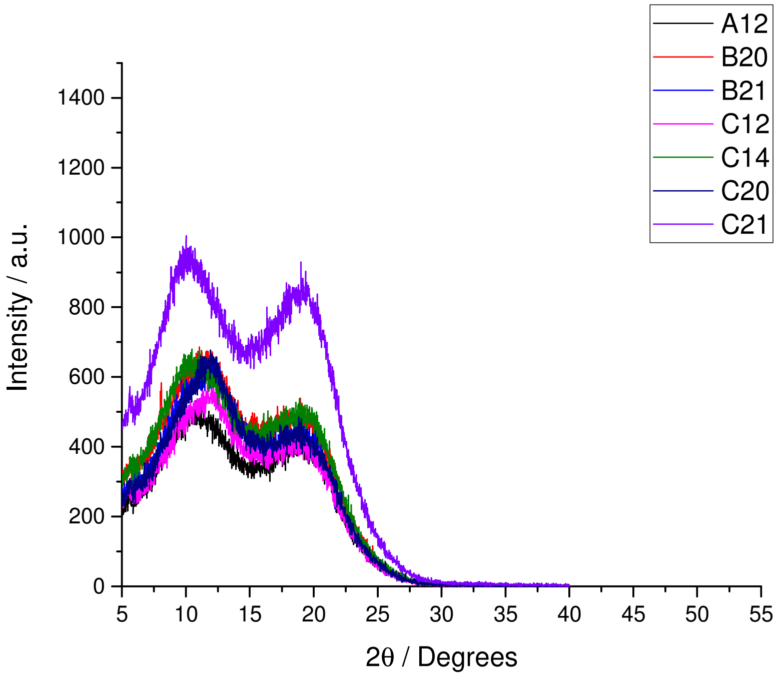

Supplementary Figure 76: Overlaid PXRDs of the crude samples (post-isolation) that went against our initial targeted cages and formed either a **Tri^{2}Di^3** cage or a **Tri^{2}Di^3/Tri^{4}Di^6** mixture instead of the targeted **Tri^{4}Di^6**, indicating that the non-purified cages are amorphous in nature.

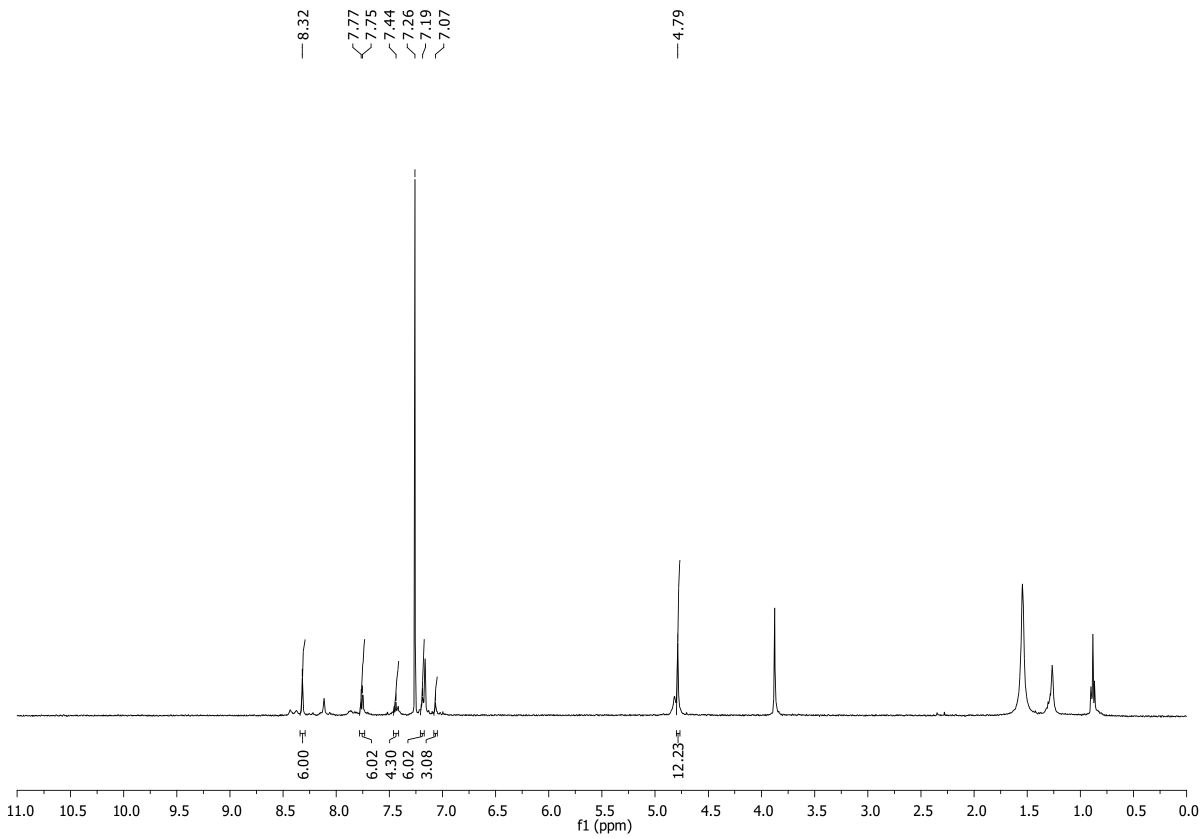

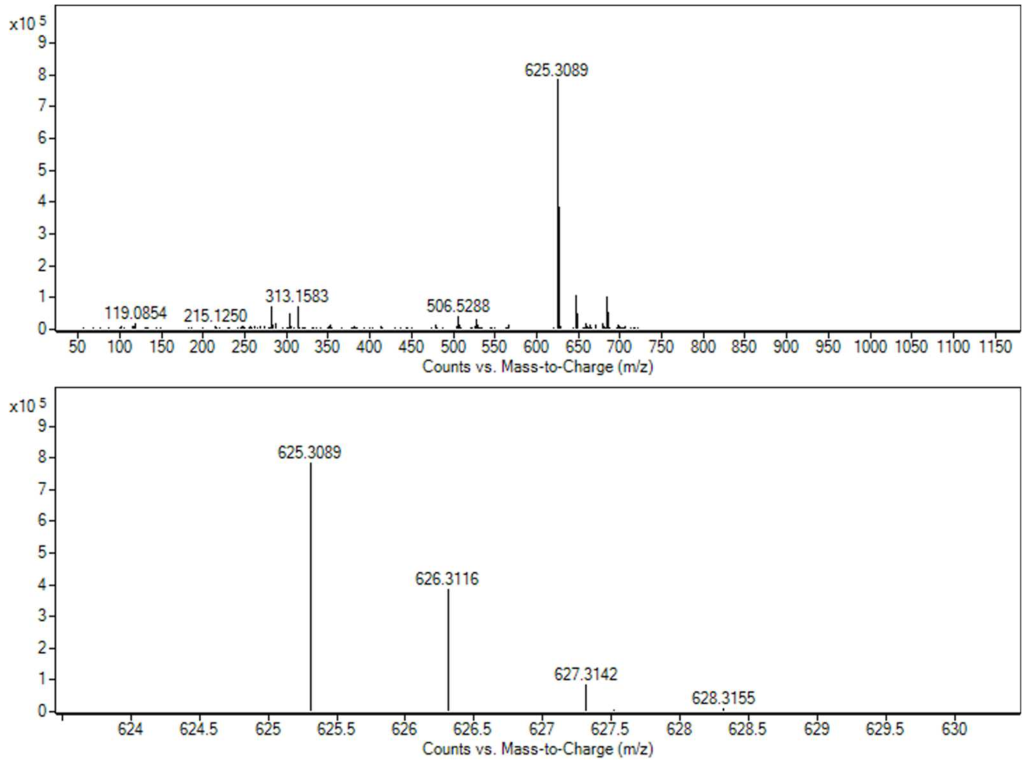

Supplementary Figure 77: ^{1}H NMR spectra ($CDCl_3$, upper) and HRMS spectra (lower) for cage **$A1_{[2+3]}$**

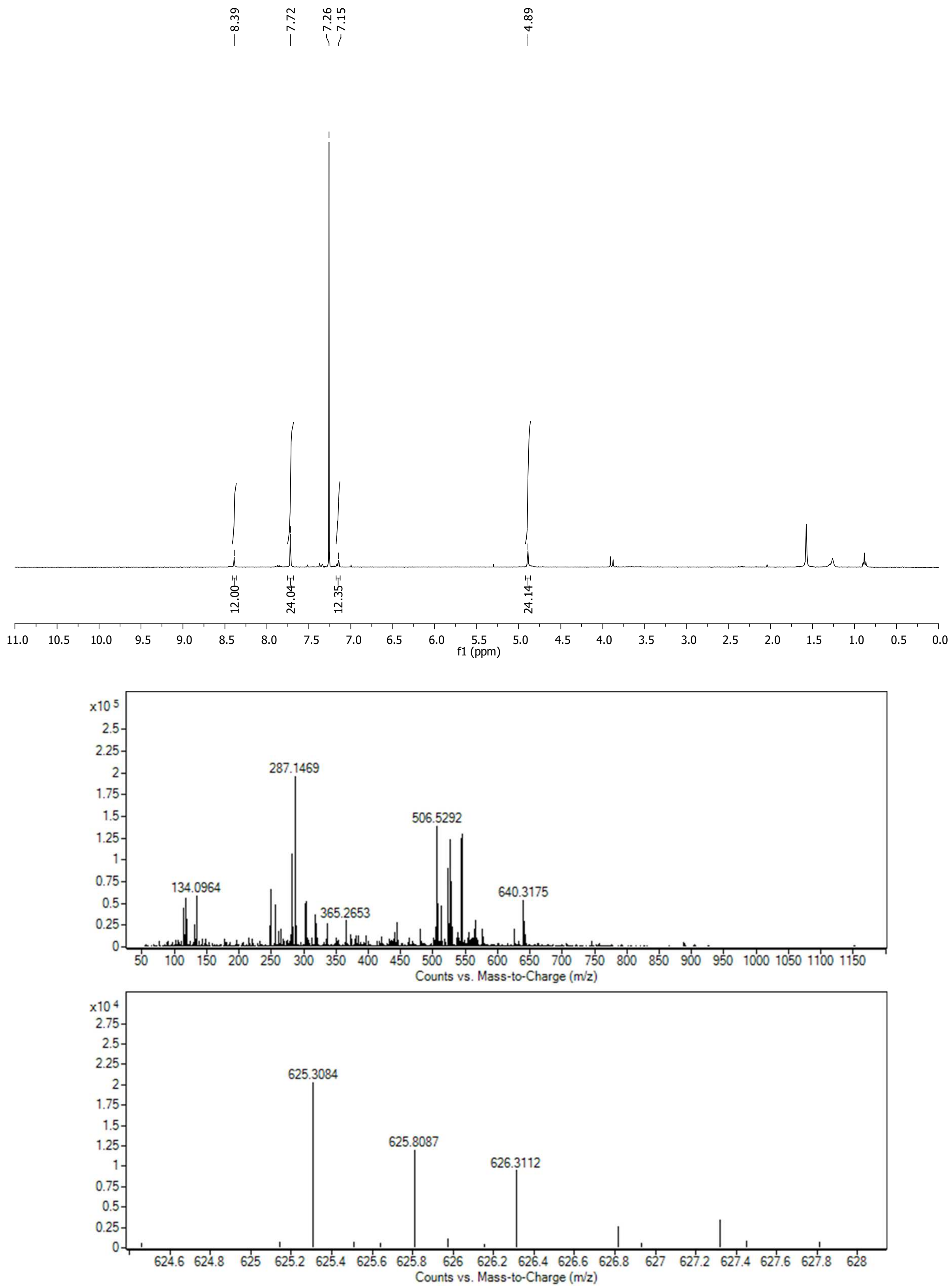

Supplementary Figure 78: ^{1}H NMR spectra ($CDCl_3$, upper) and HRMS spectra (lower) for cage **A11$_{[4+6]}$**

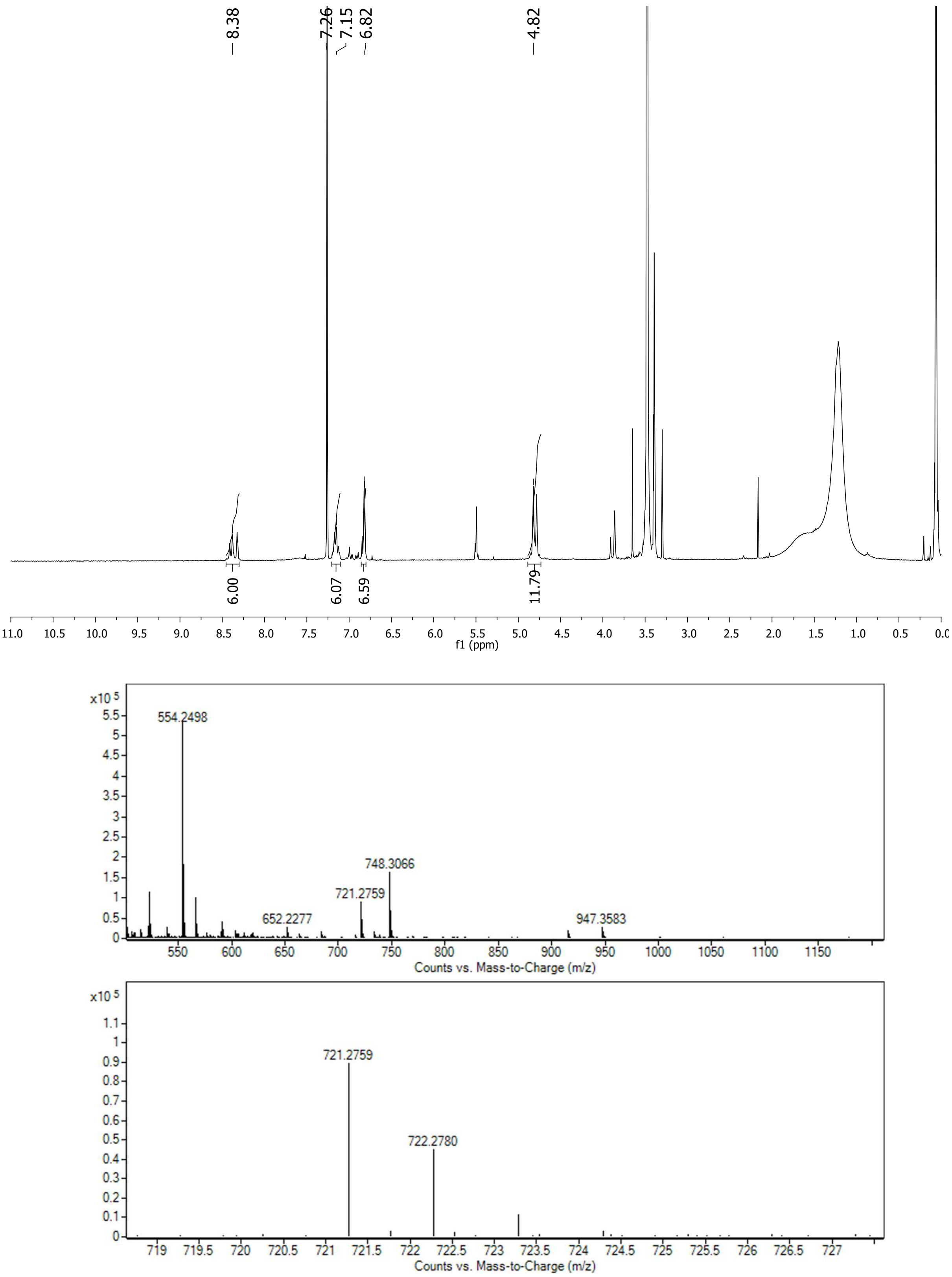

Supplementary Figure 79: ^{1}H NMR spectra ($CDCl_3$, upper) and HRMS spectra (lower) for cage **A12$_{[2+3]}$**

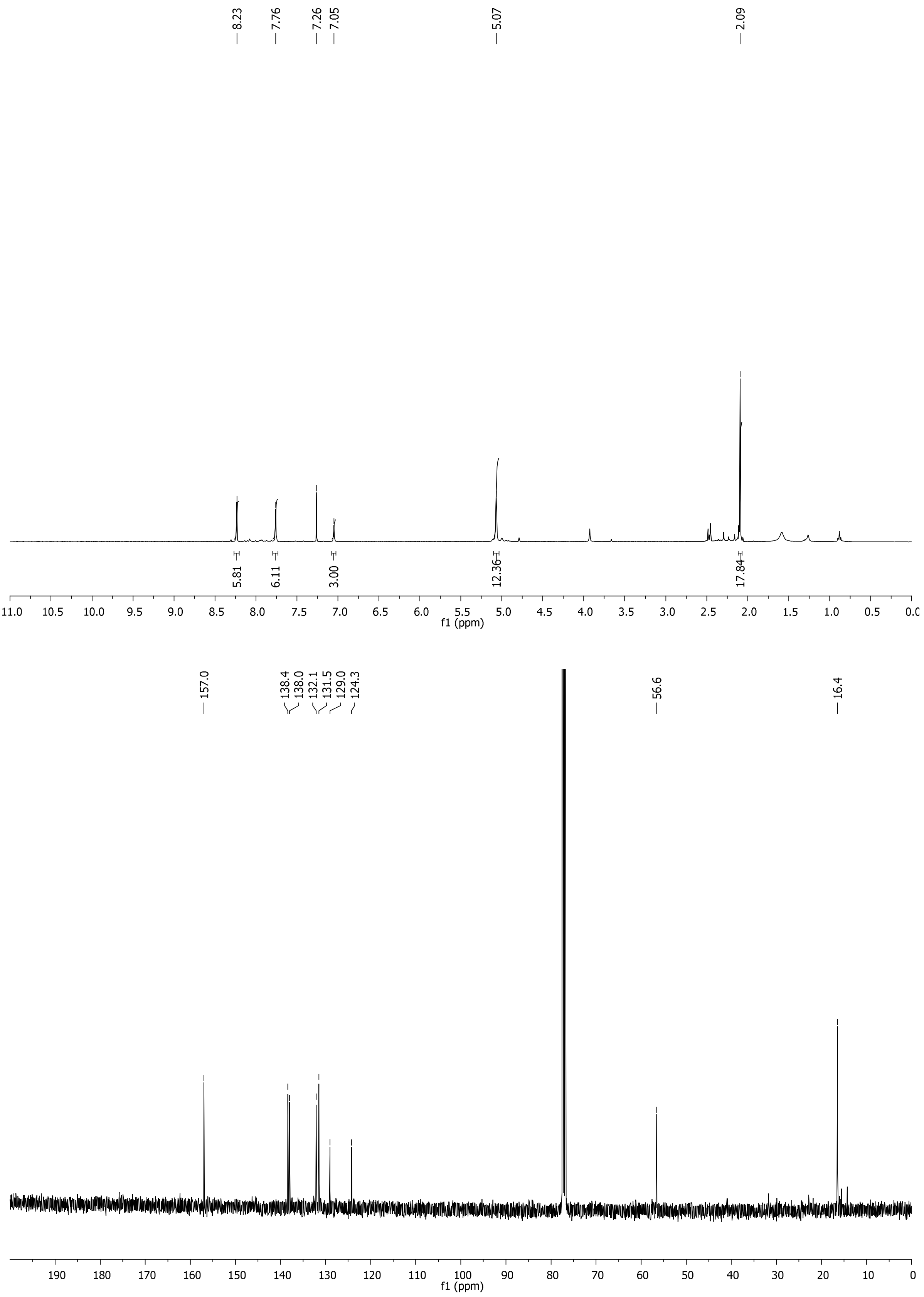

Supplementary Figure 80: ^{1}H NMR spectra ($CDCl_3$; upper) and ^{13}C NMR spectra ($CDCl_3$; lower) for cage **B2**$_{\mathbf{[2+3]}}$

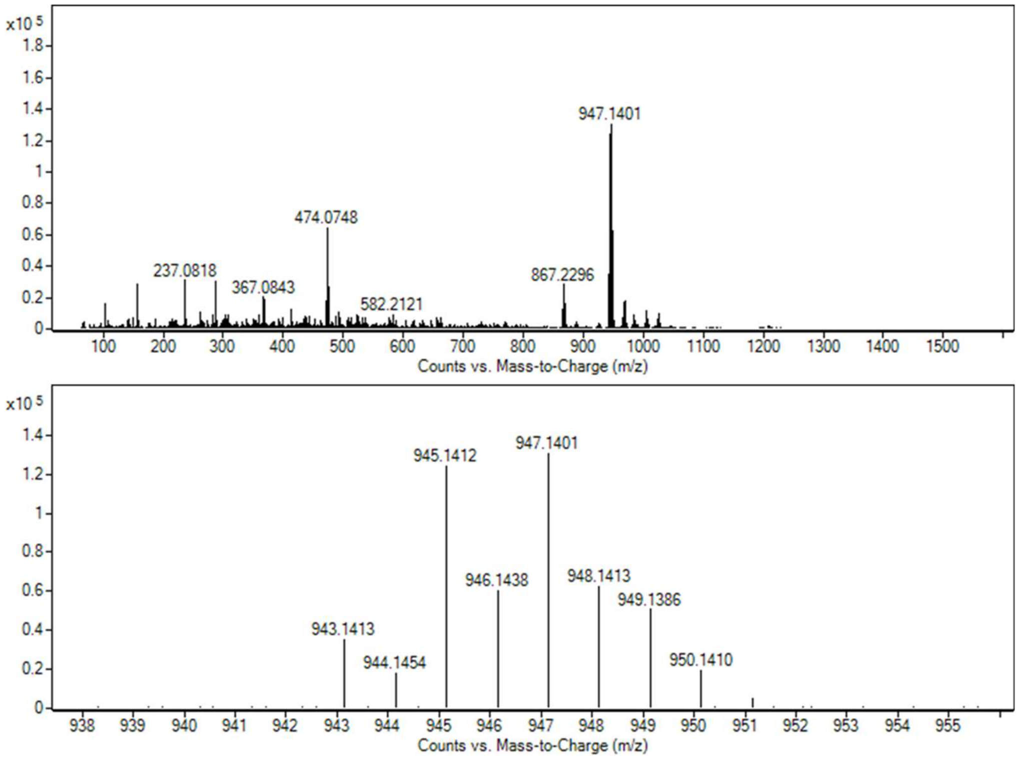

Supplementary Figure 81: HRMS spectra for cage **B2$_{[2+3]}$**

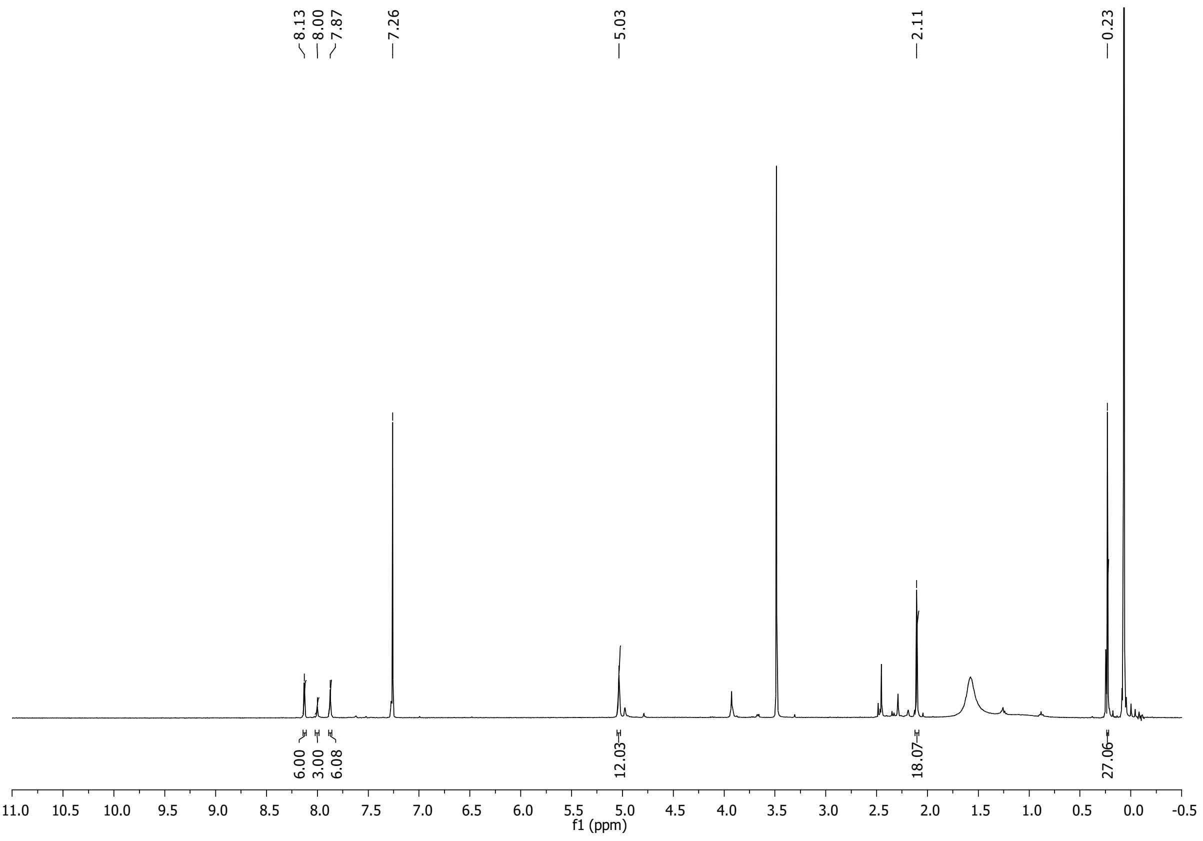

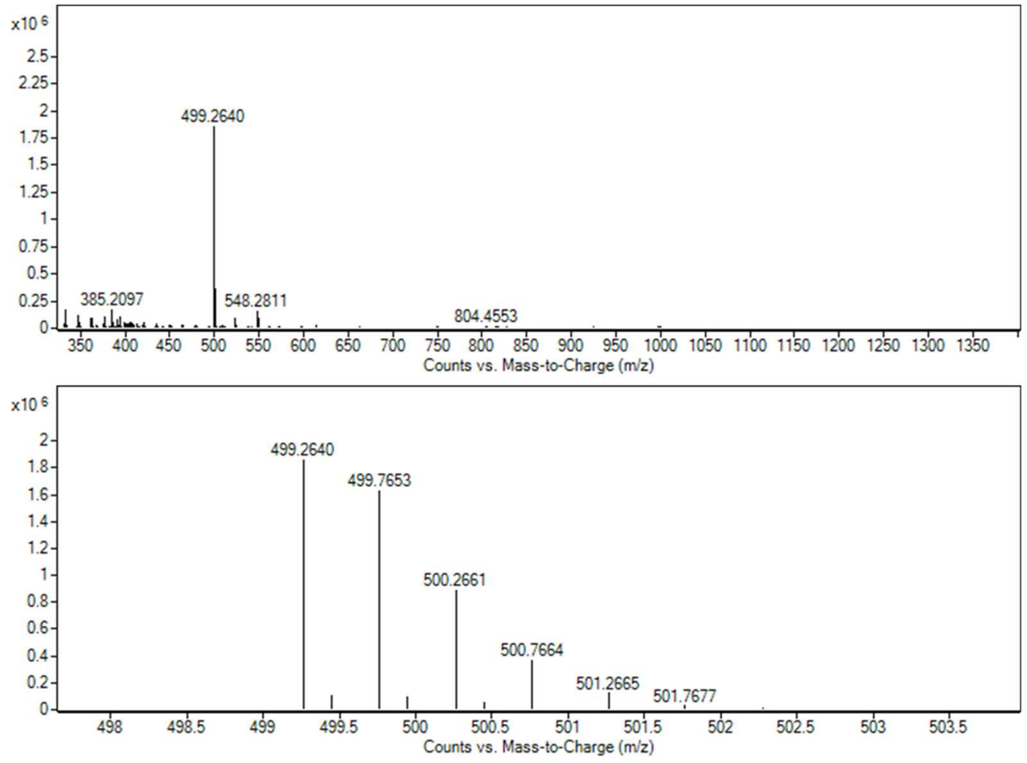

Supplementary Figure 82: ^{1}H NMR spectra ($CDCl_3$, upper) and HRMS spectra (lower) for cage **B4$_{[2+3]}$**

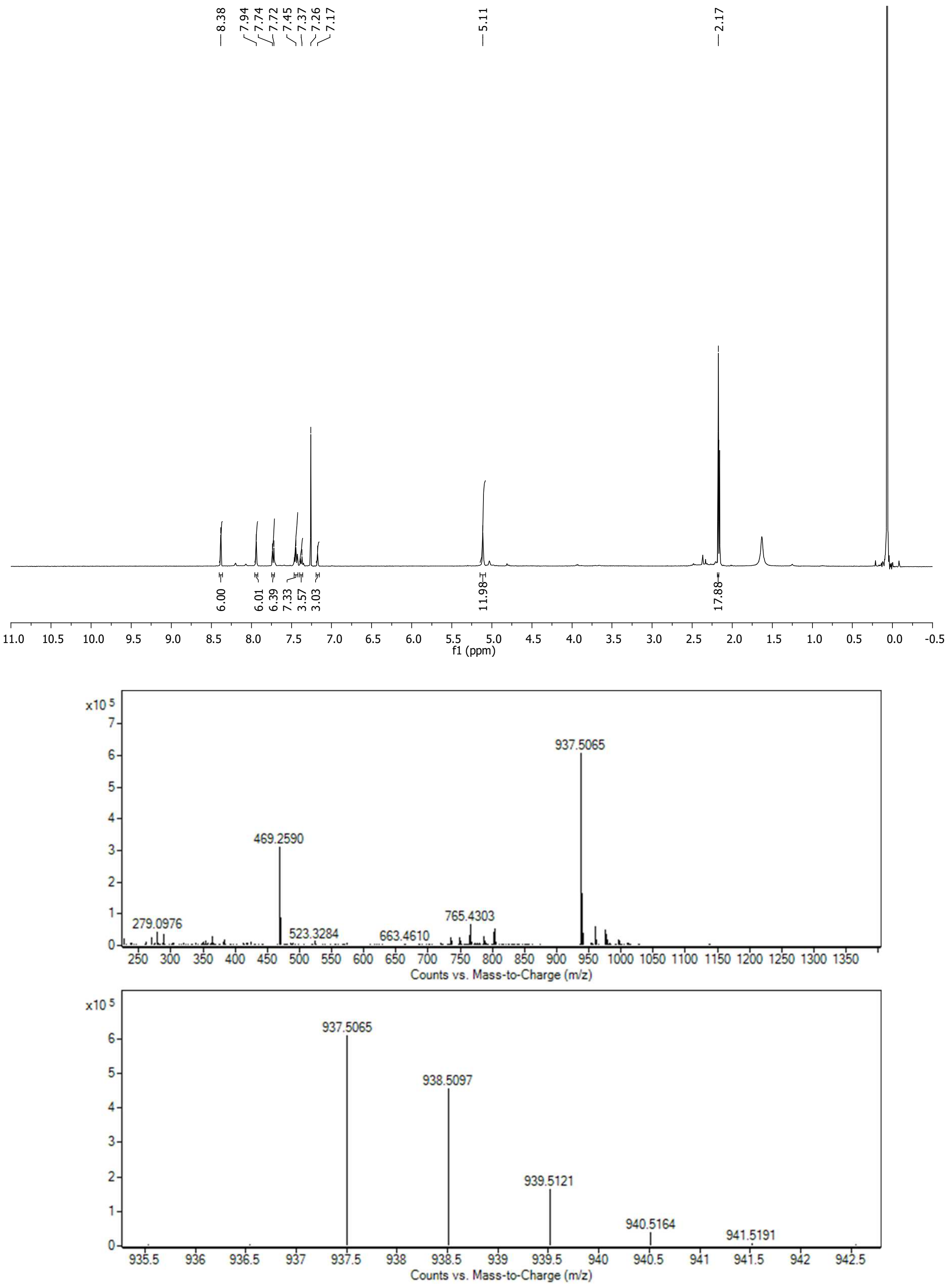

Supplementary Figure 83: ^{1}H NMR spectra ($CDCl_3$, upper) and HRMS spectra (lower) for cage **B5$_{[2+3]}$**

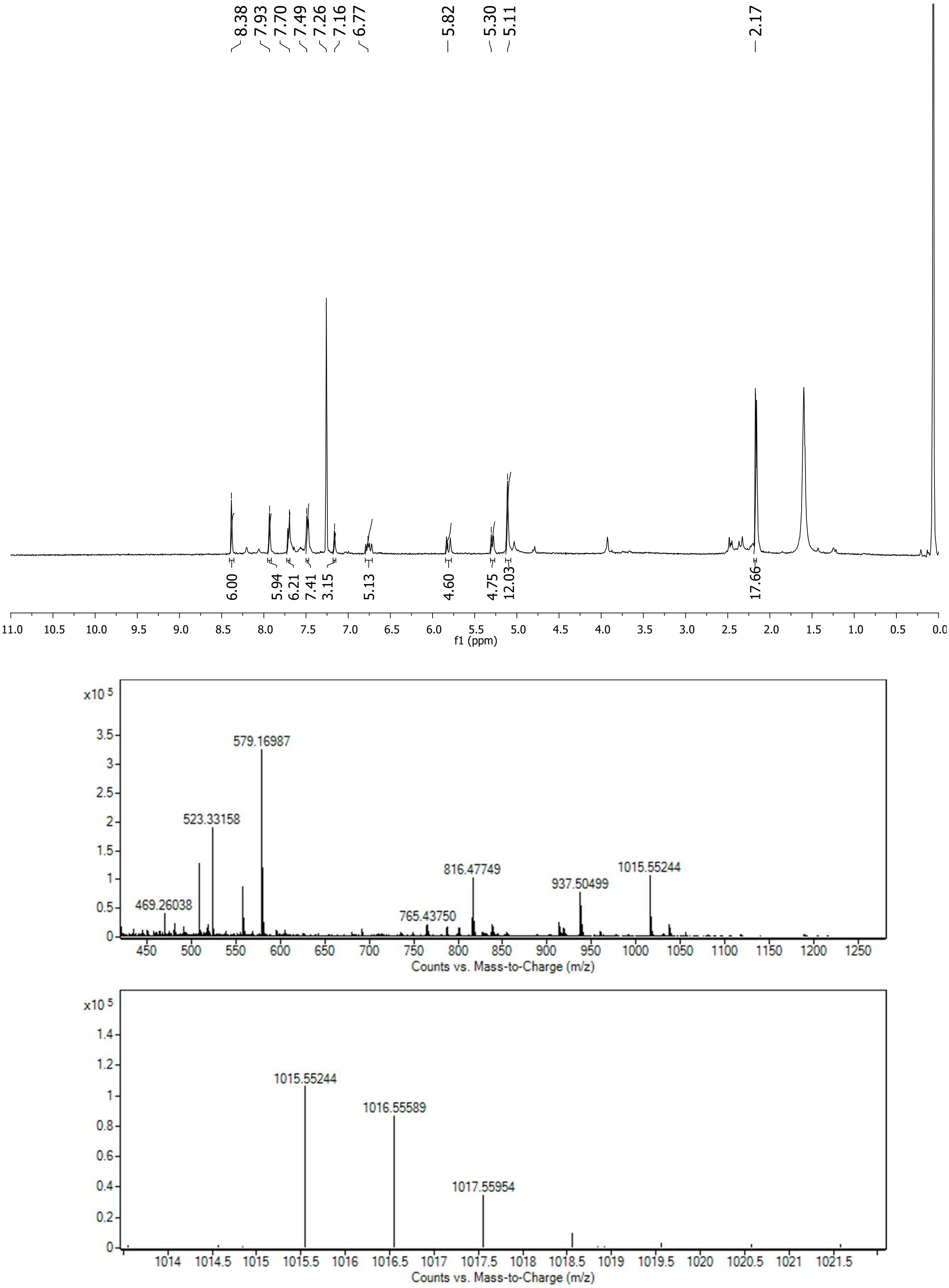

Supplementary Figure 84: ^{1}H NMR spectra ($CDCl_3$, upper) and HRMS spectra (lower) for cage **$B6_{[2+3]}$**

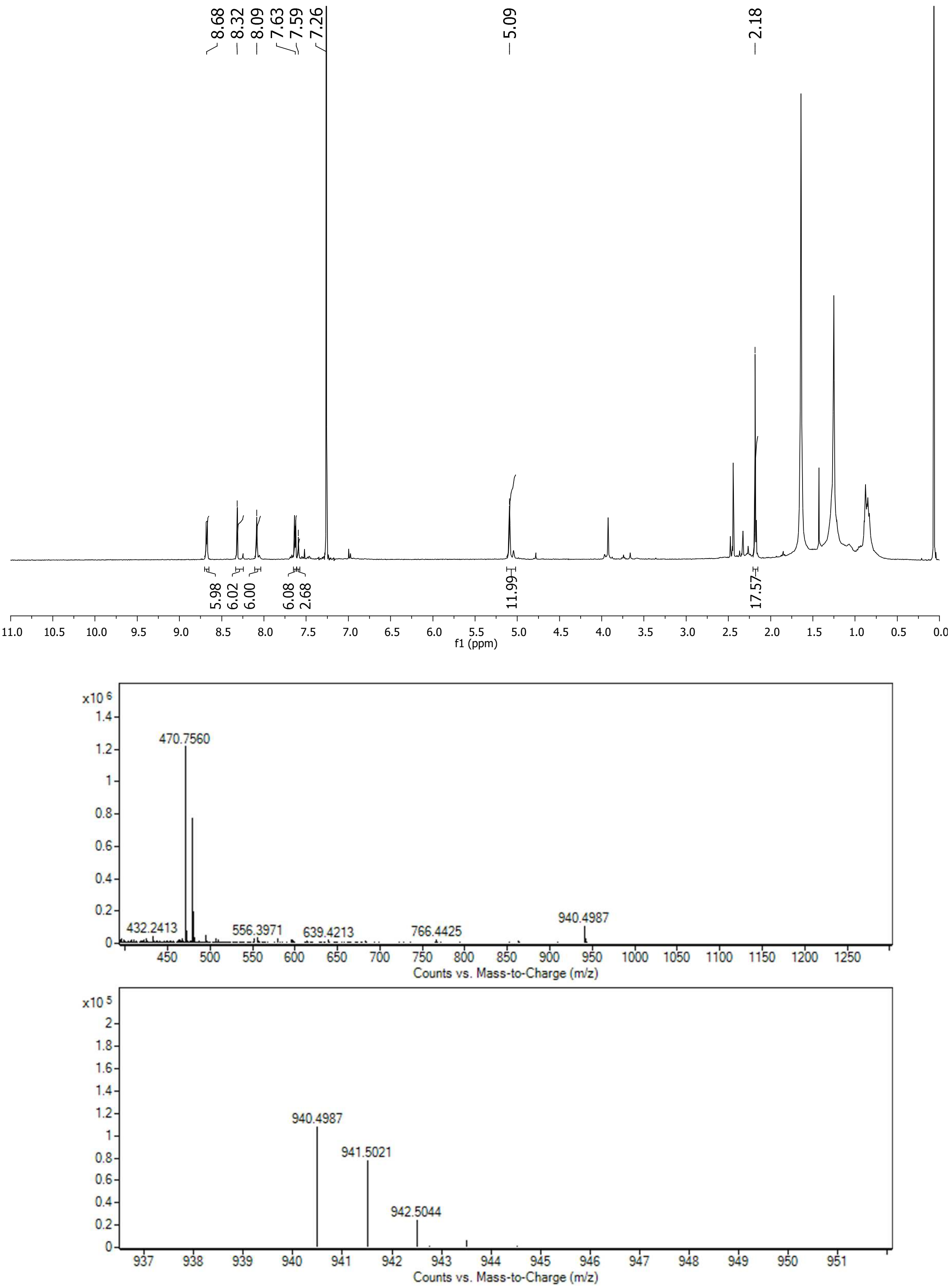

Supplementary Figure 85: ^{1}H NMR spectra ($CDCl_3$, upper) and HRMS spectra (lower) for cage **$B8_{[2+3]}$**

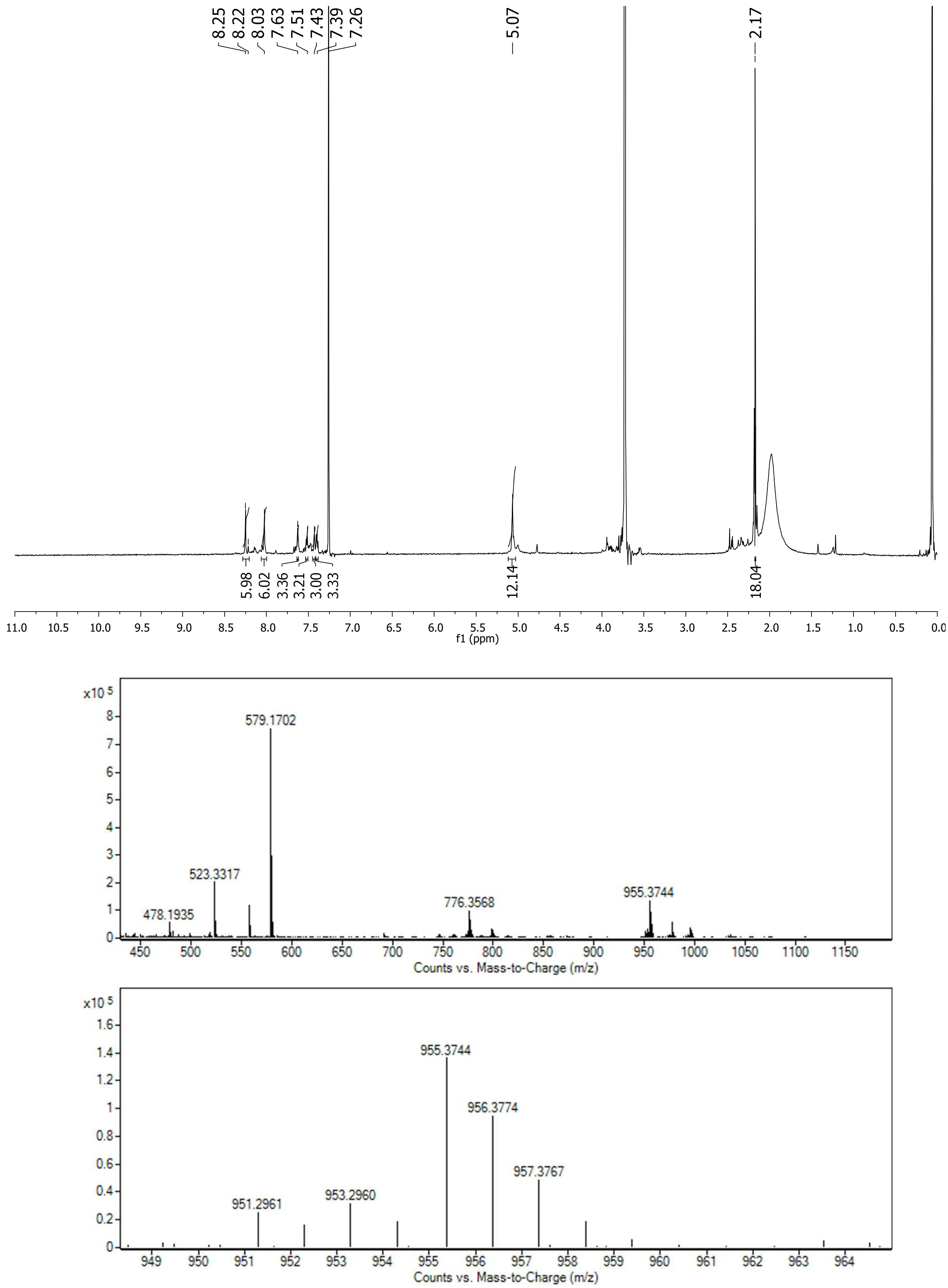

Supplementary Figure 86: ^{1}H NMR spectra ($CDCl_3$, upper) and HRMS spectra (lower) for cage **B9$_{[2+3]}$**

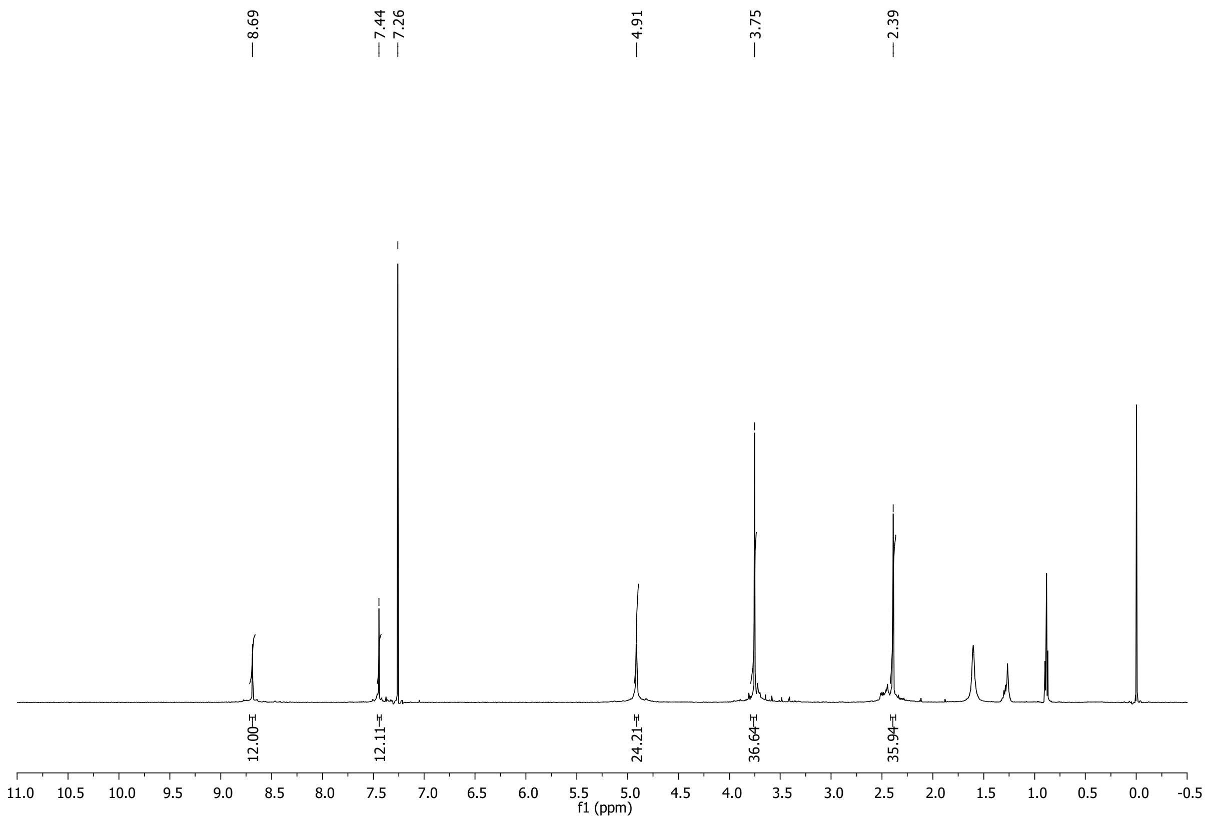

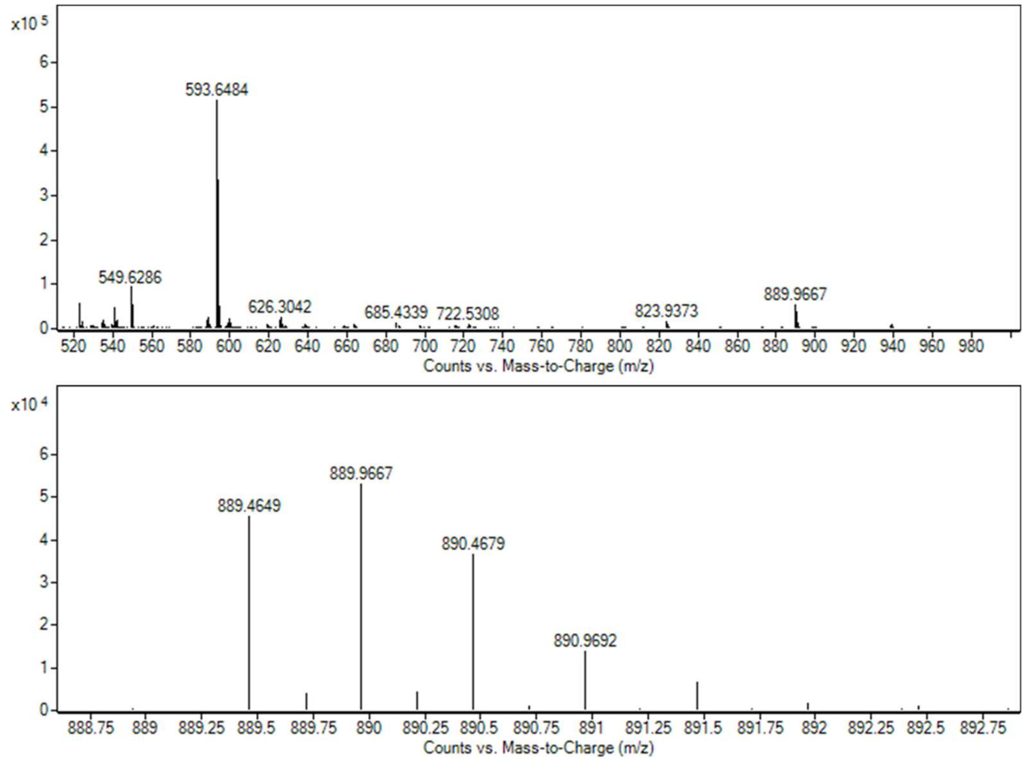

Supplementary Figure 87: ^{1}H NMR spectra ($CDCl_3$, upper) and HRMS spectra (lower) for cage **B13$_{[4+6]}$**

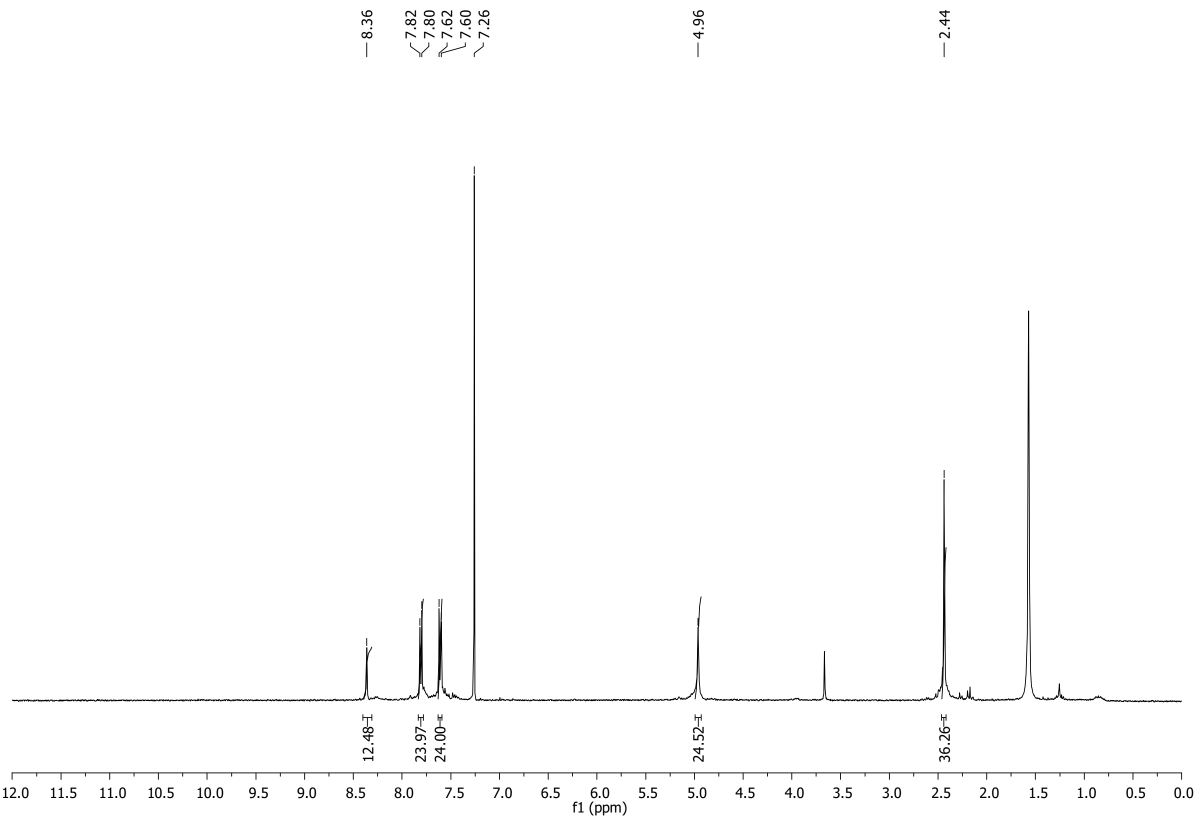

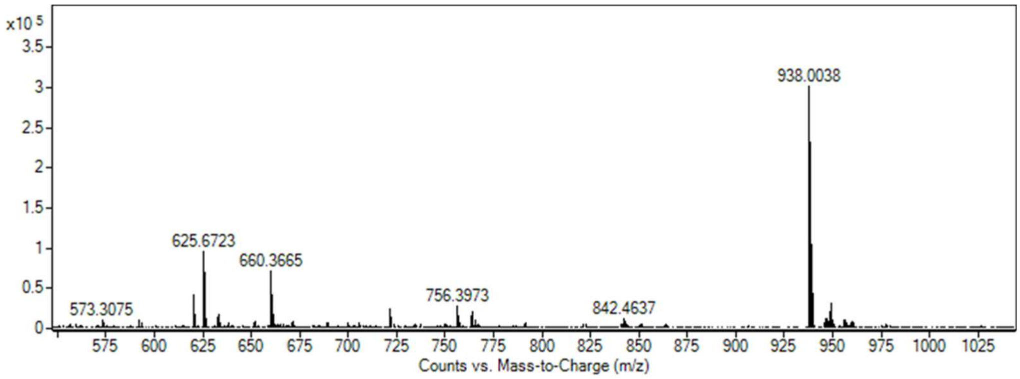

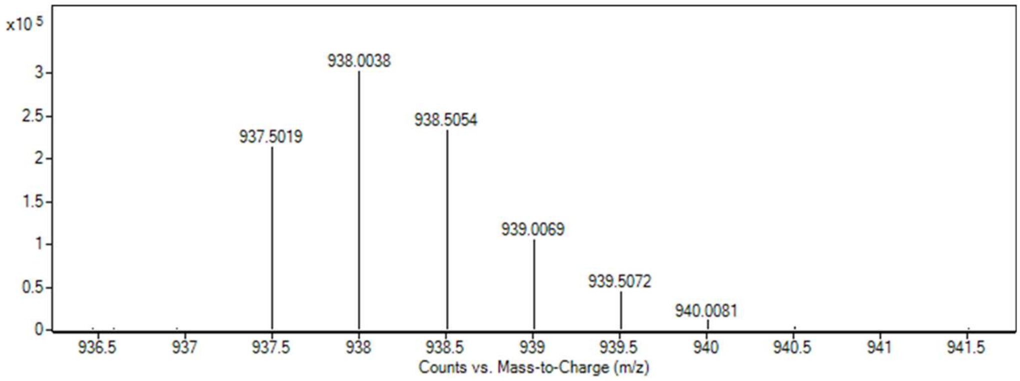

Supplementary Figure 88: ^{1}H NMR spectra ($CDCl_3$, upper) and HRMS spectra (lower) for cage **B15$_{[4+6]}$**

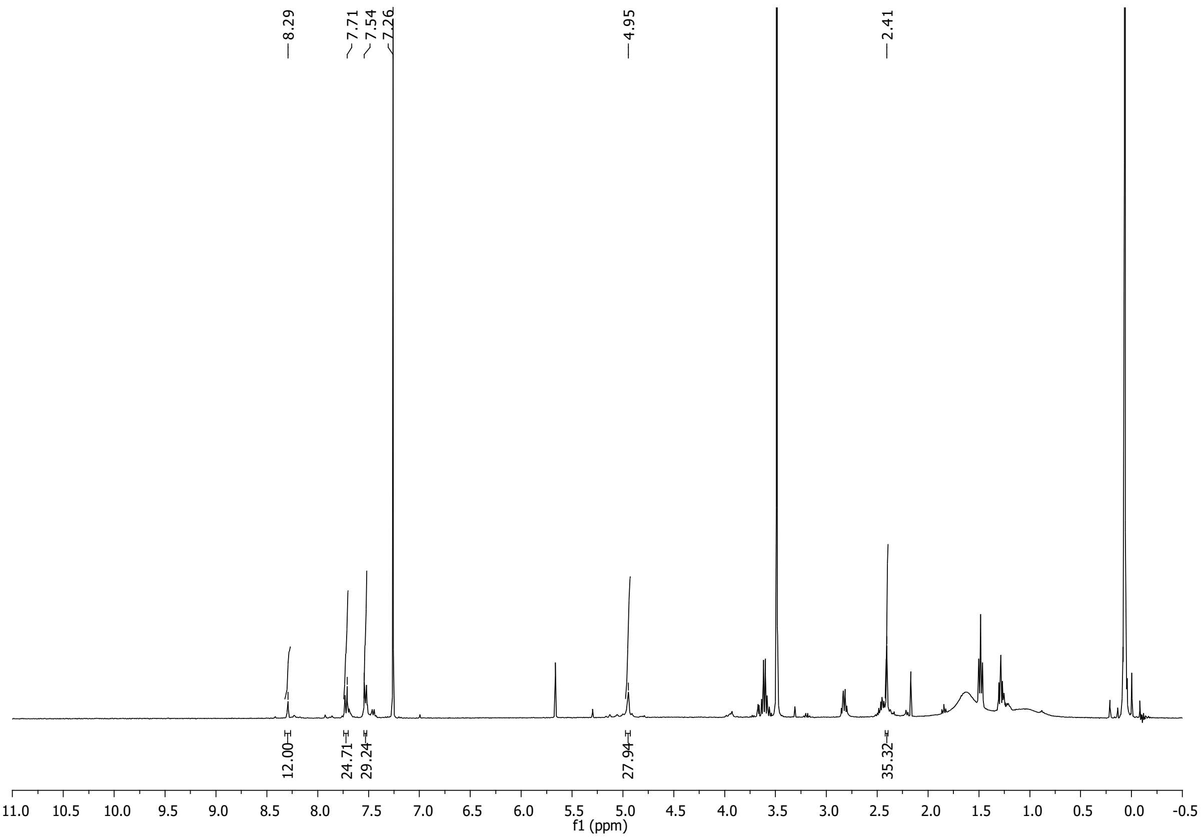

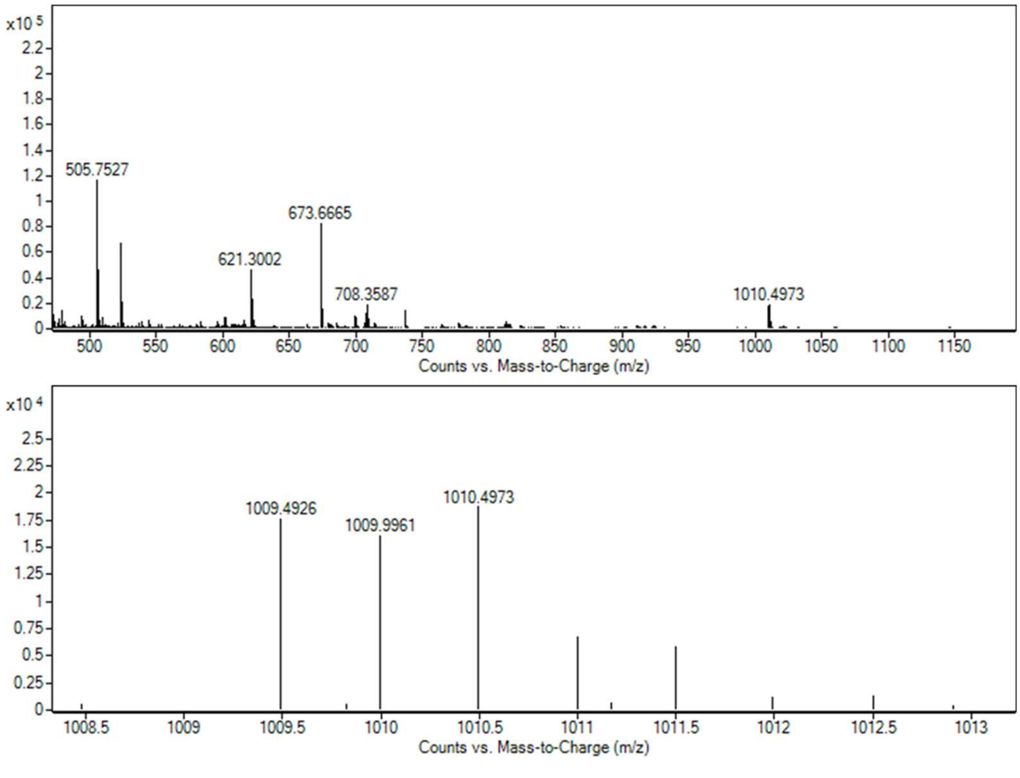

Supplementary Figure 89: ^{1}H NMR spectra ($CDCl_3$, upper) and HRMS spectra (lower) for cage **B18$_{[4+6]}$**

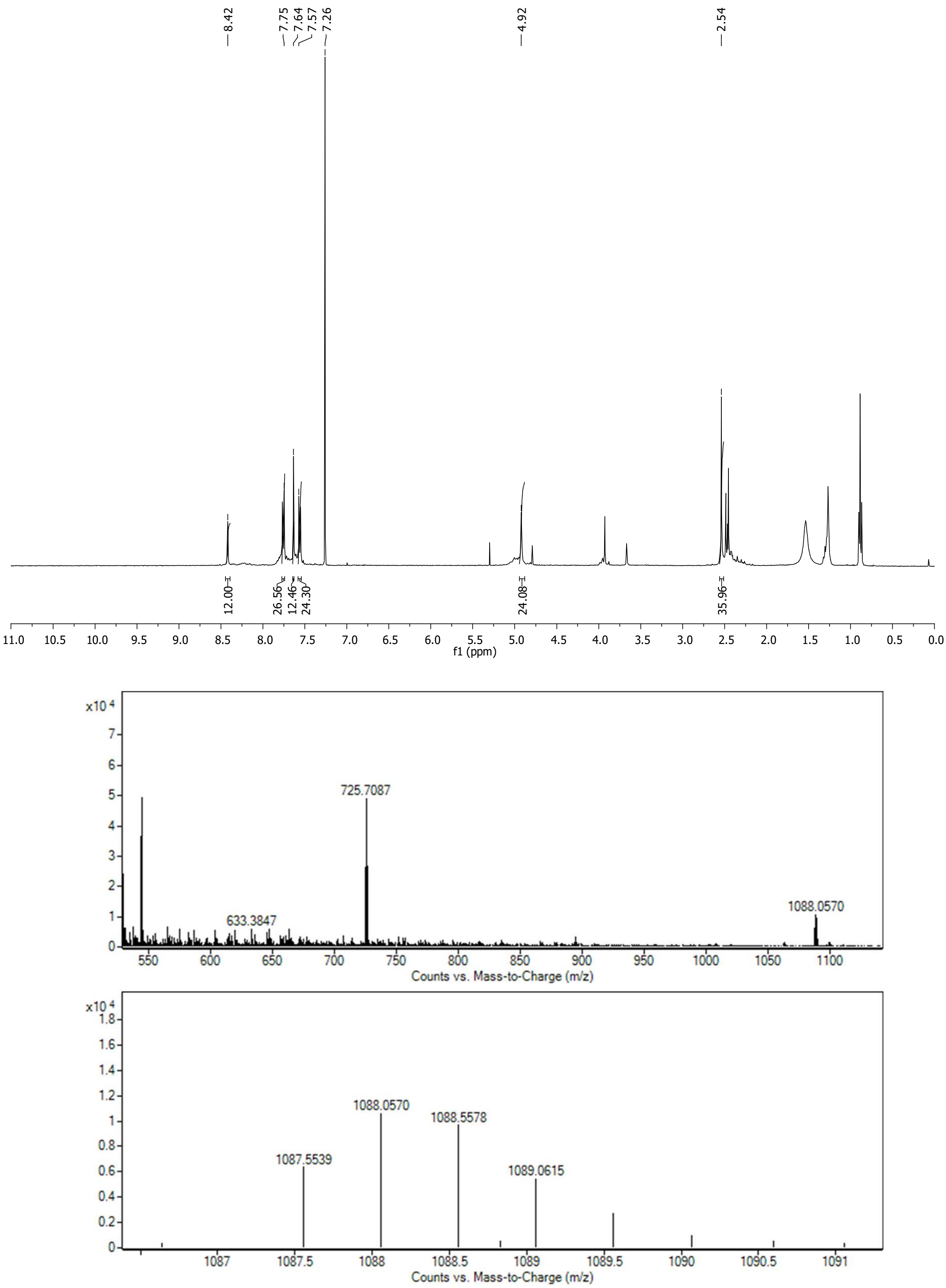

Supplementary Figure 90: ^{1}H NMR spectra ($CDCl_3$, upper) and HRMS spectra (lower) for cage **$B24_{[4+4]}$**

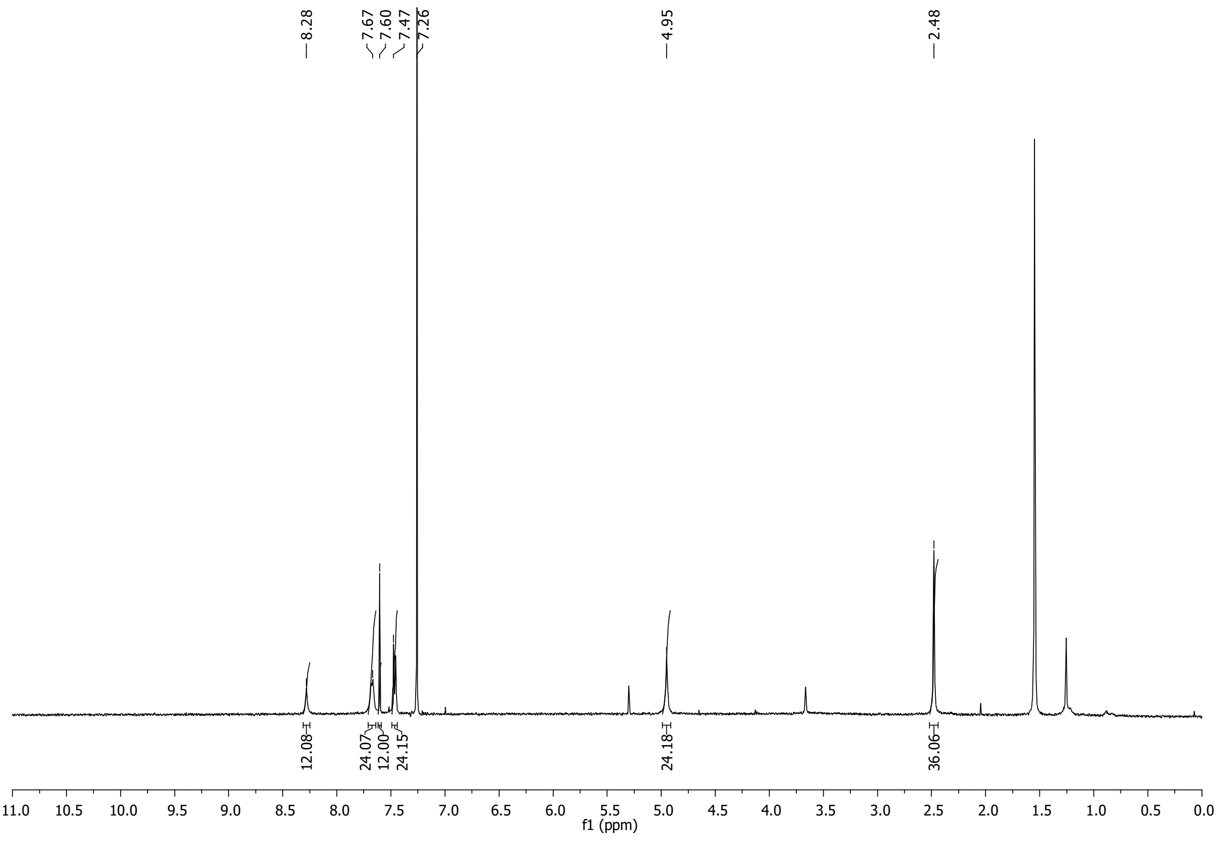

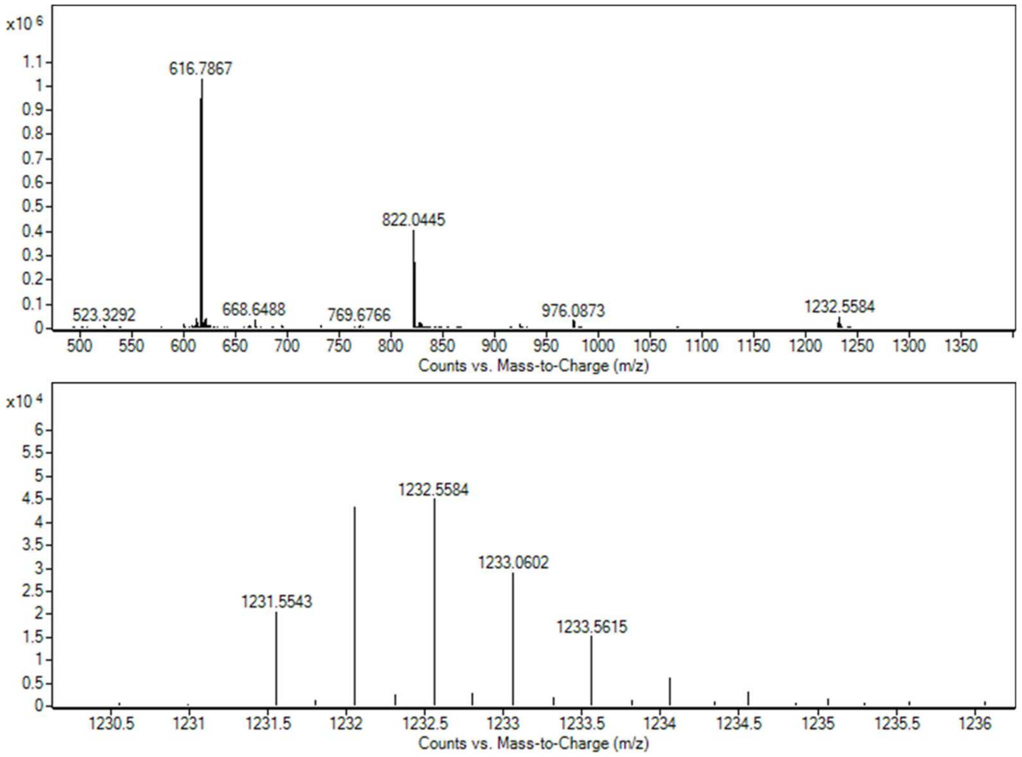

Supplementary Figure 91: ^{1}H NMR spectra ($CDCl_3$, upper) and HRMS spectra (lower) for cage **B25$_{[4+4]}$**

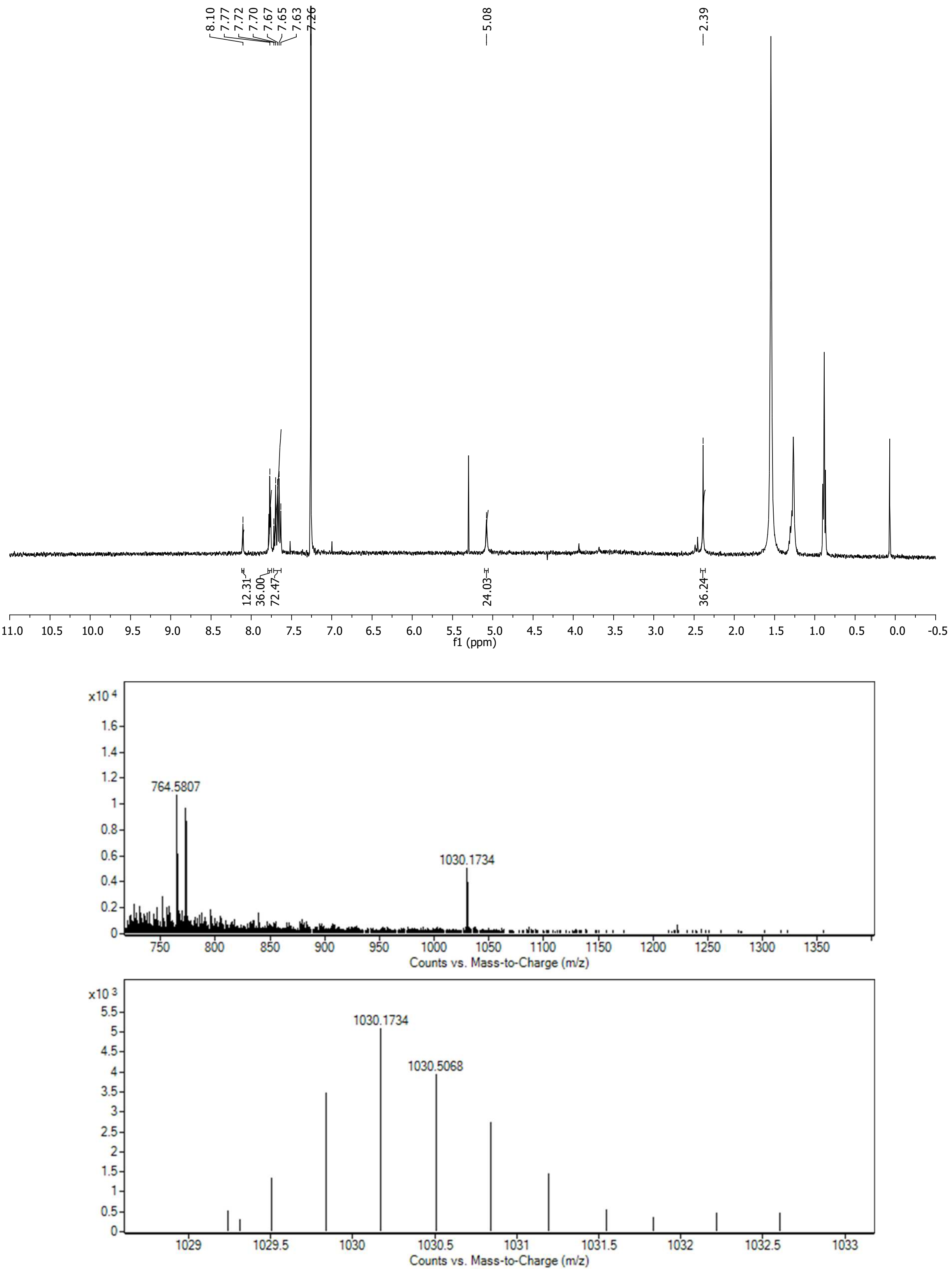

Supplementary Figure 92: ^{1}H NMR spectra ($CDCl_3$, upper) and HRMS spectra (lower) for cage **B26$_{[4+4]}$**

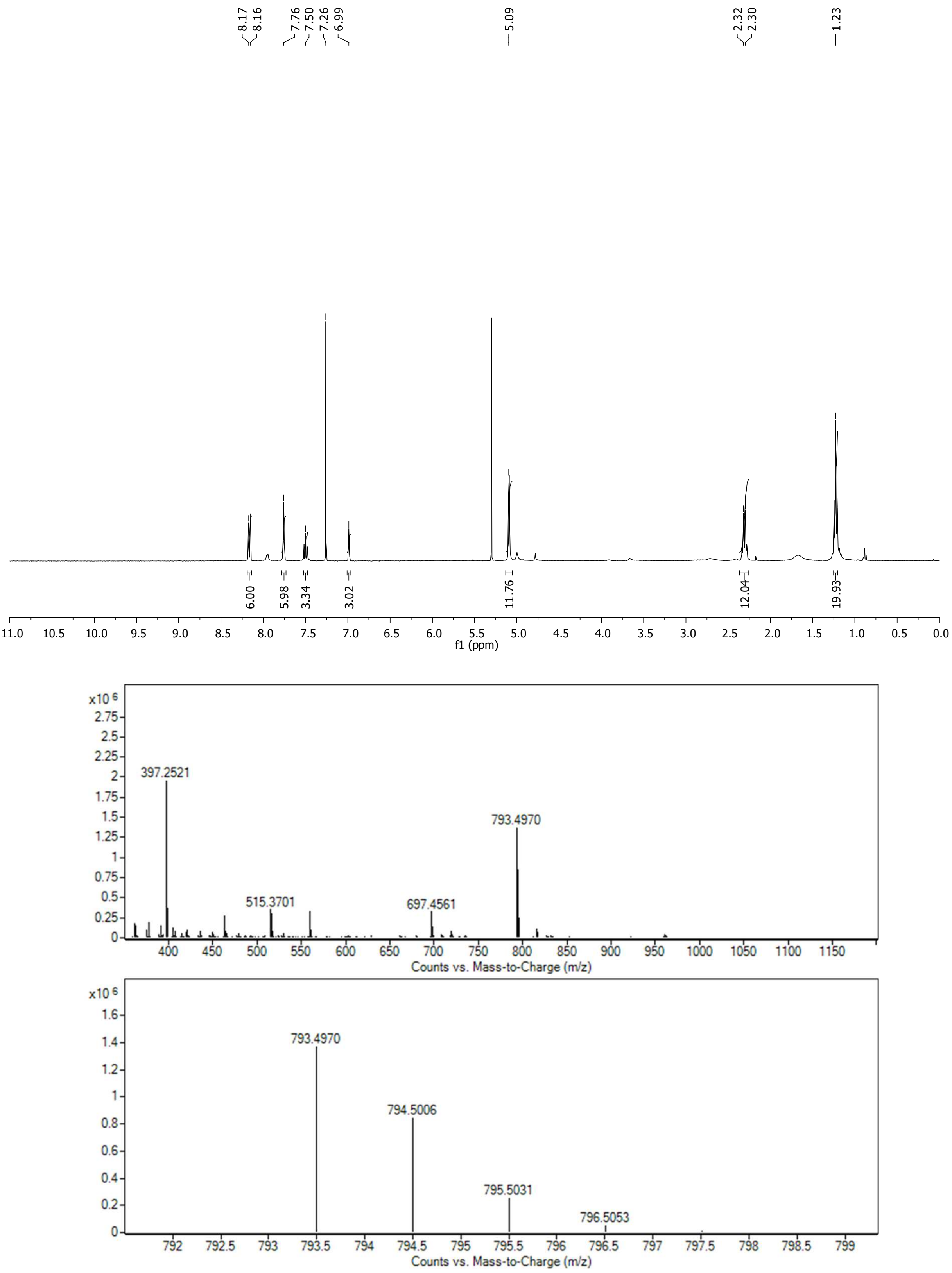

Supplementary Figure 93: ^{1}H NMR spectra ($CDCl_3$, upper) and HRMS spectra (lower) for cage **$C1_{[2+3]}$**

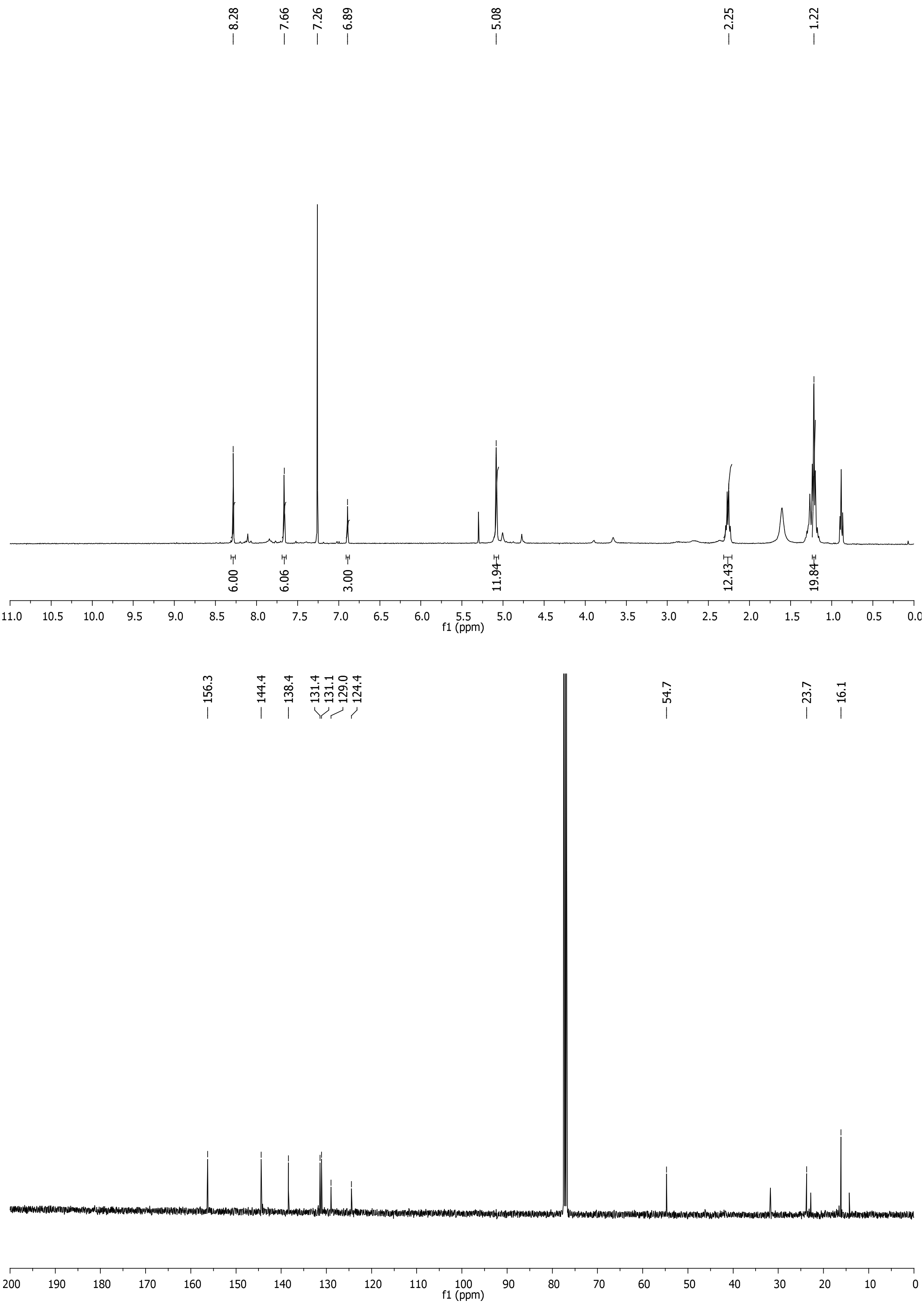

Supplementary Figure 94: ^{1}H NMR spectra ($CDCl_3$; upper) and ^{13}C NMR spectra ($CDCl_3$; lower) for cage **$C2_{[2+3]}$**

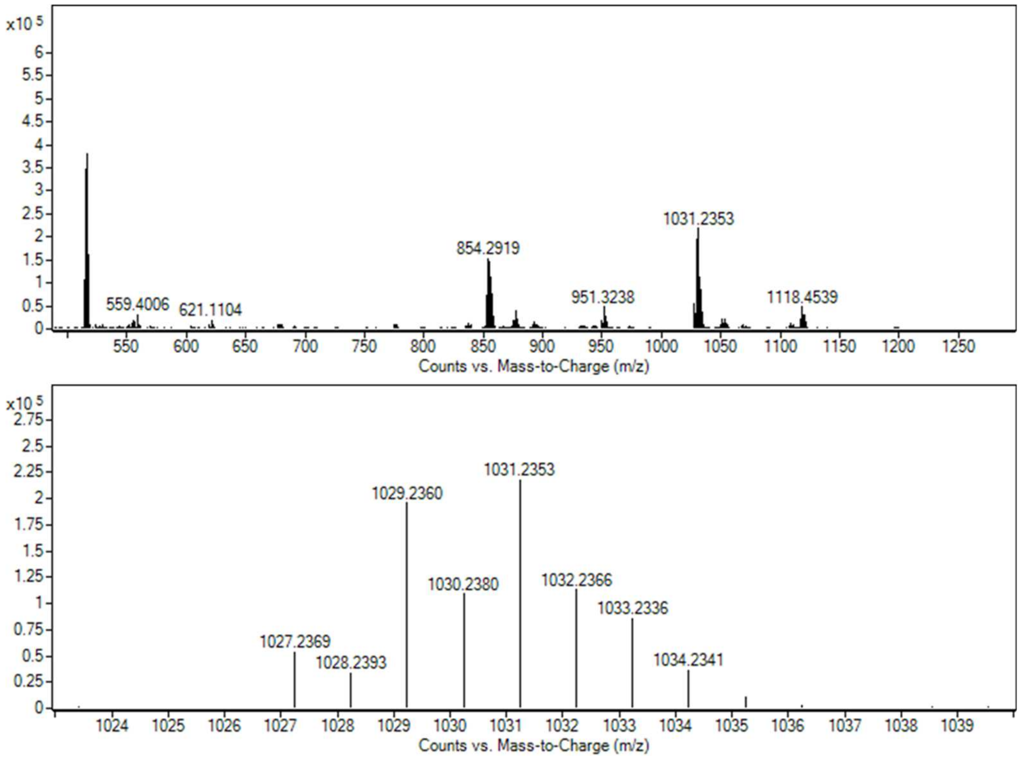

Supplementary Figure 95: HRMS spectra for cage **C2$_{[2+3]}$**

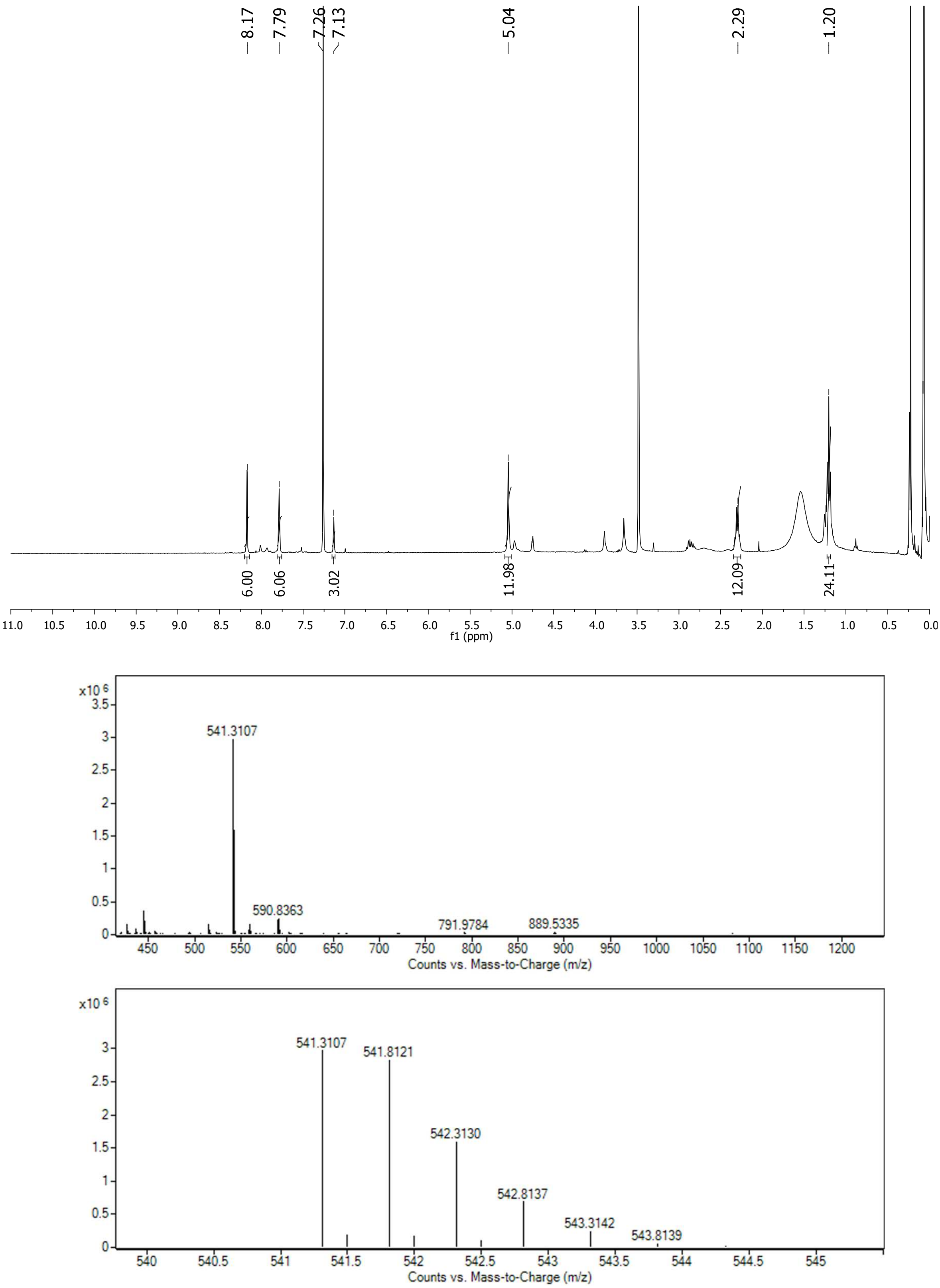

Supplementary Figure 96: ^{1}H NMR spectra ($CDCl_3$, upper) and HRMS spectra (lower) for cage **$C4_{[2+3]}$**

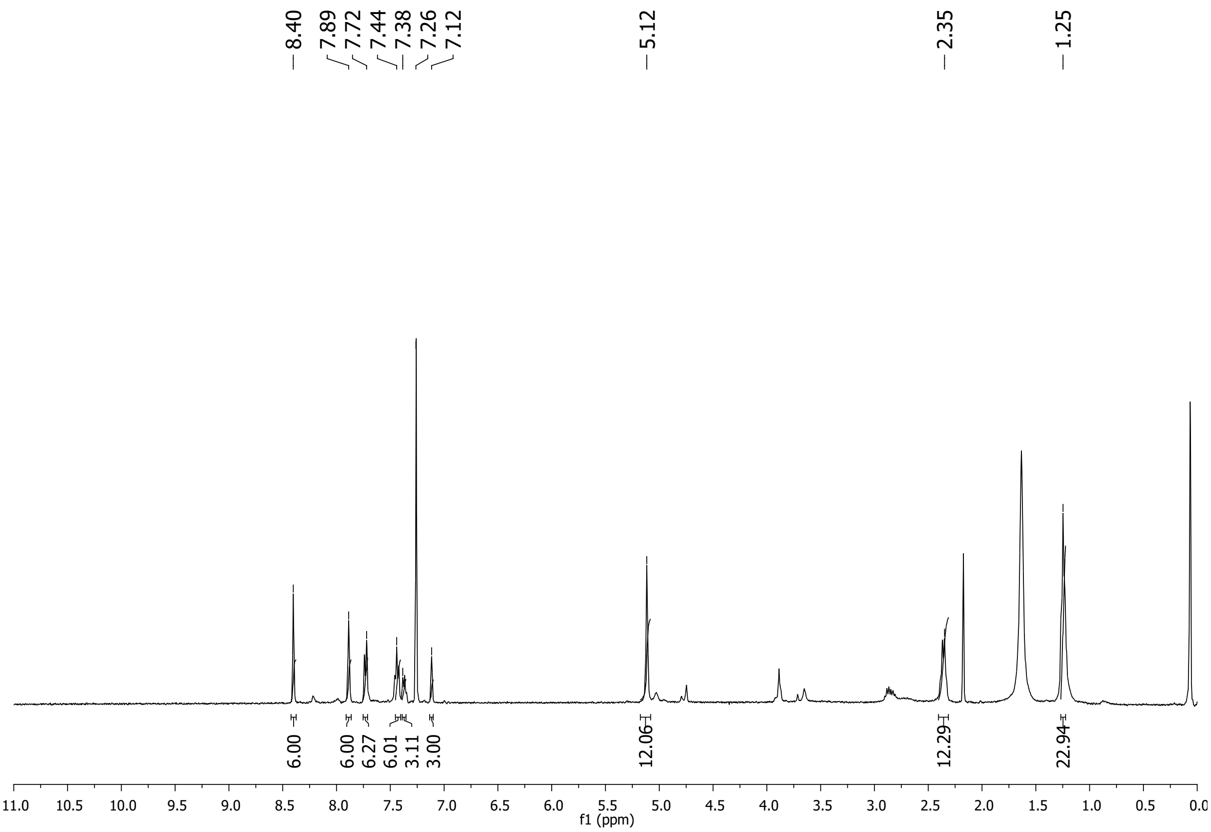

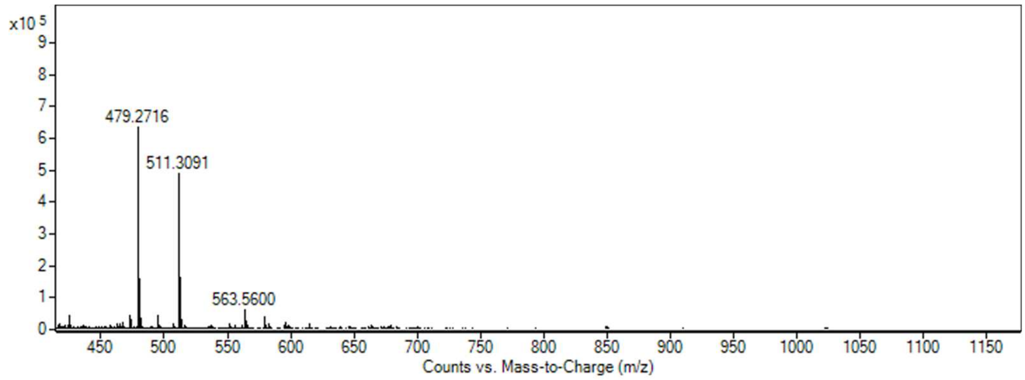

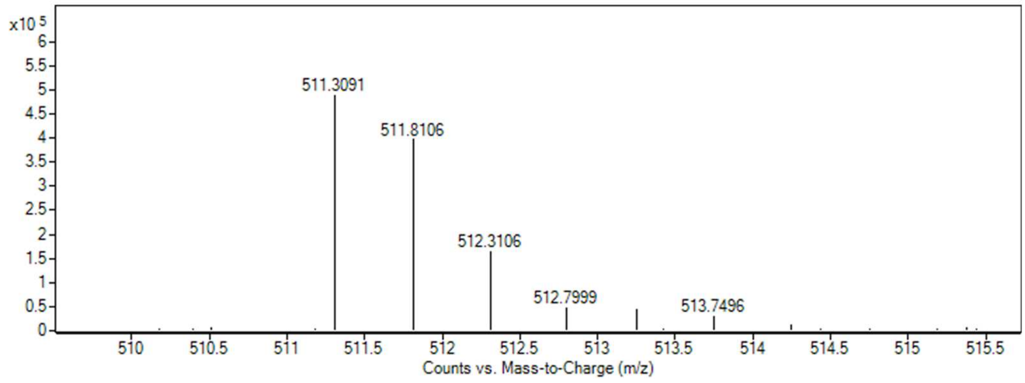

Supplementary Figure 97: ^{1}H NMR spectra ($CDCl_3$, upper) and HRMS spectra (lower) for cage **C5$_{[2+3]}$**

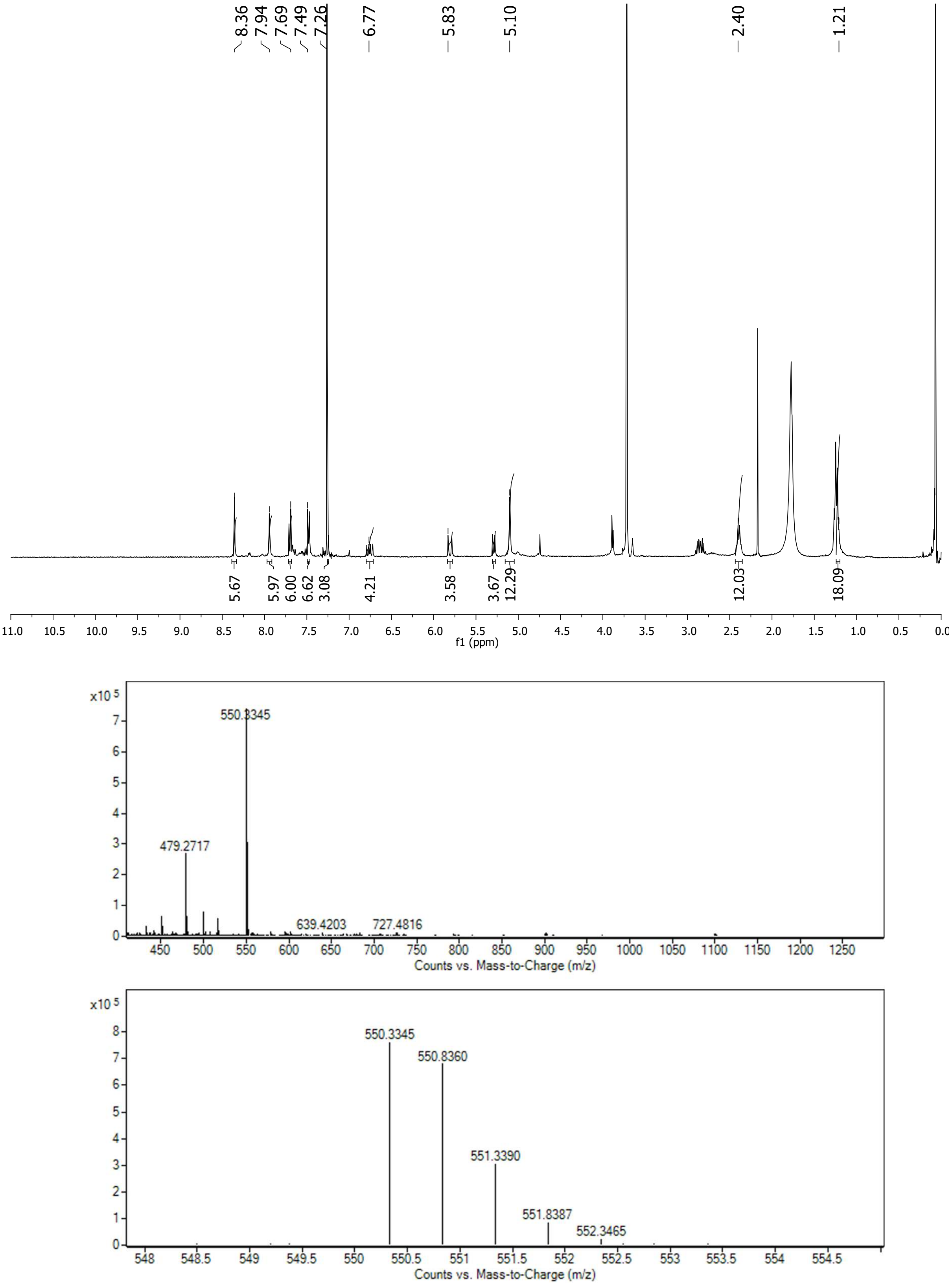

Supplementary Figure 98: ^{1}H NMR spectra ($CDCl_3$, upper) and HRMS spectra (lower) for cage **$C6_{[2+3]}$**

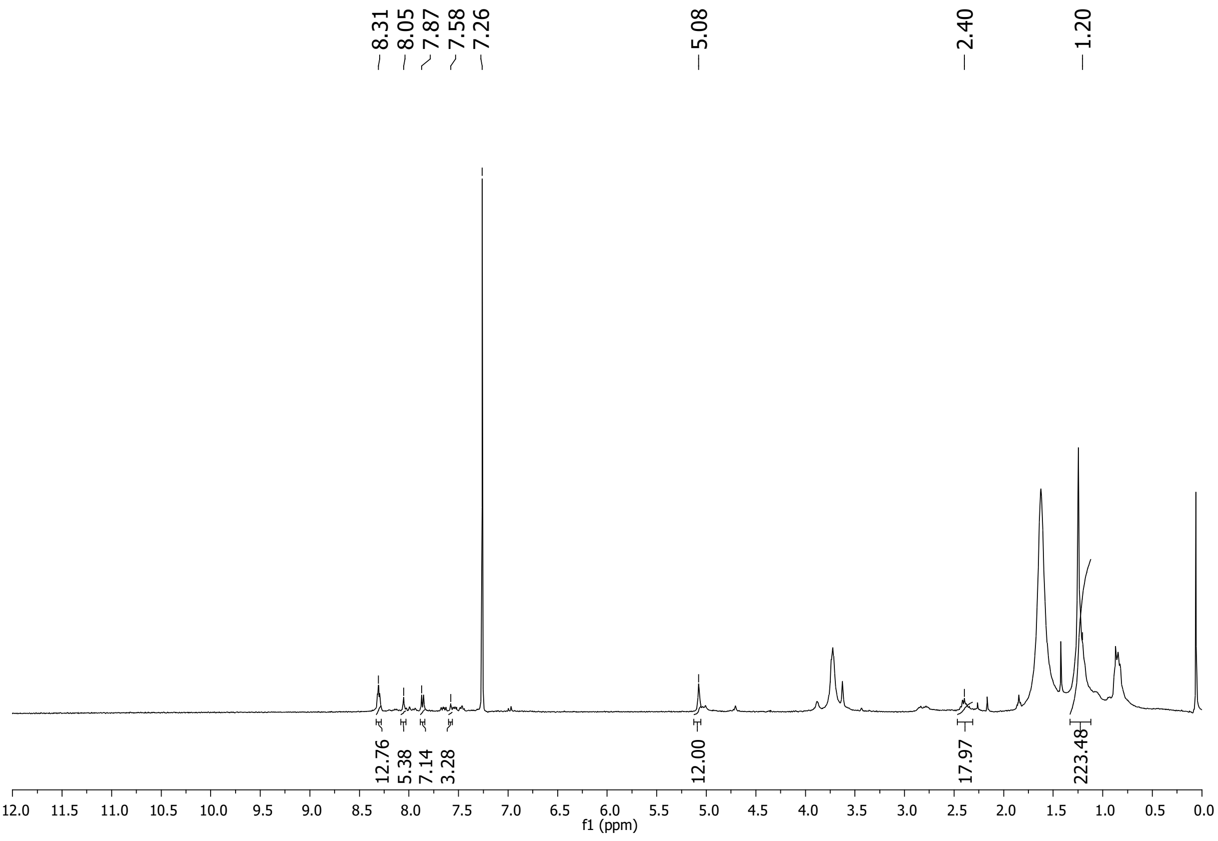

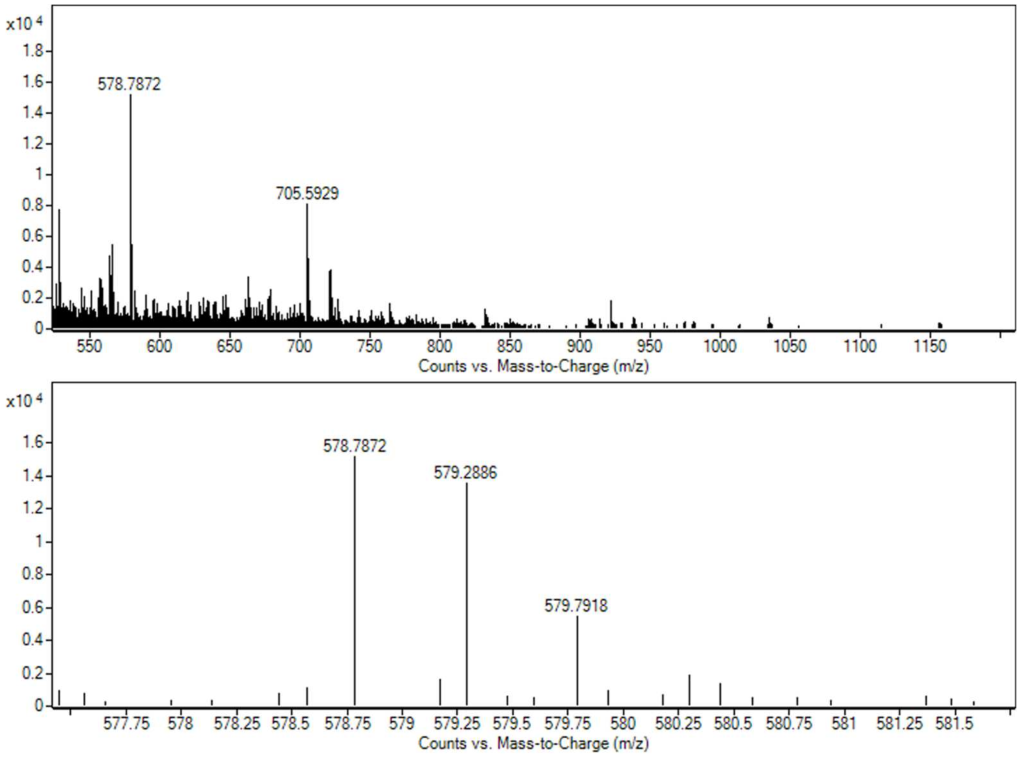

Supplementary Figure 99: ^{1}H NMR spectra ($CDCl_3$, upper) and HRMS spectra (lower) for cage **C7$_{[2+3]}$**

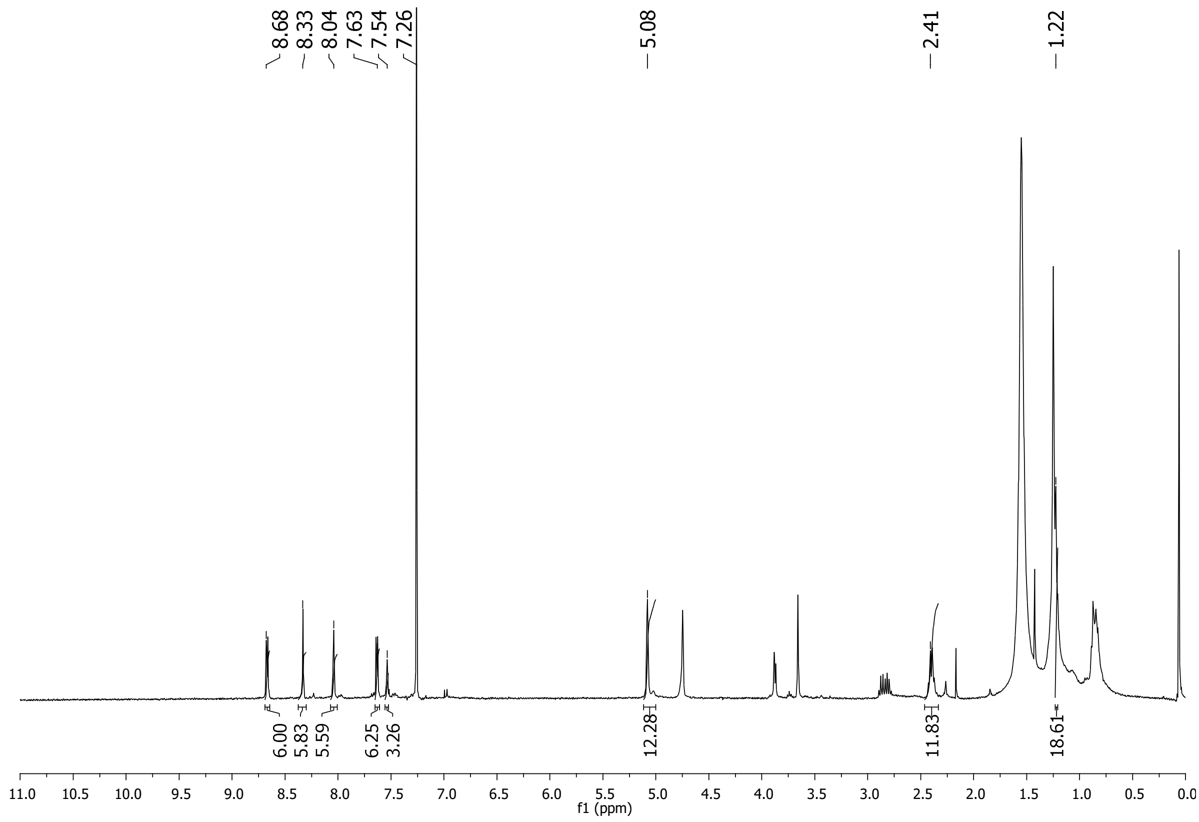

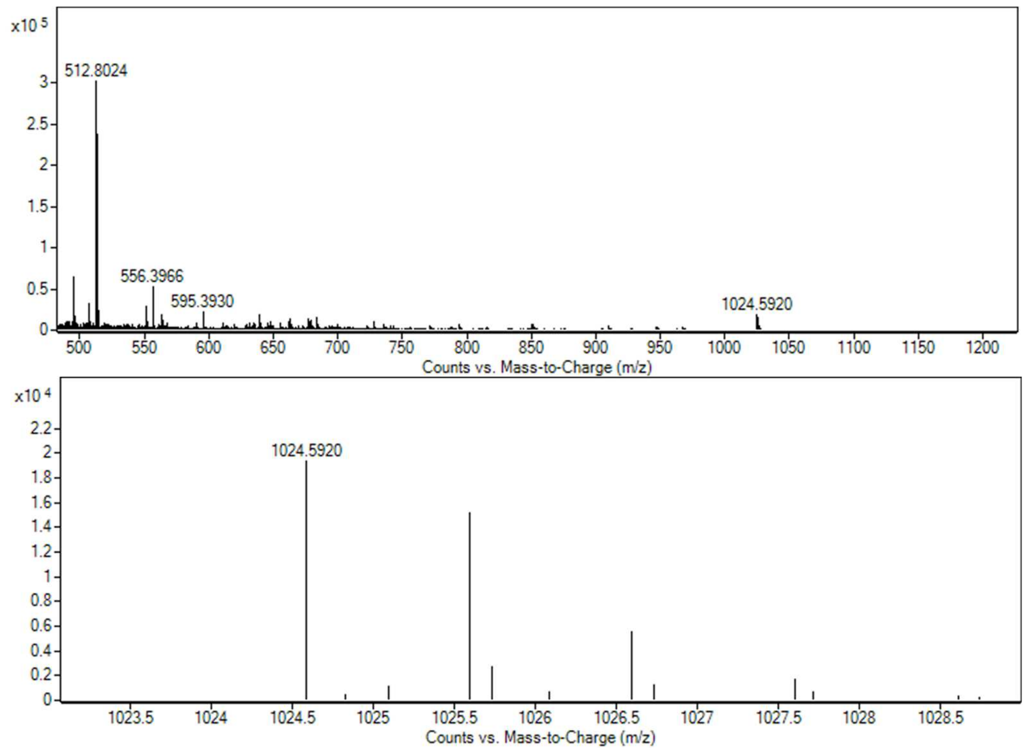

Supplementary Figure 100: ^{1}H NMR spectra ($CDCl_3$, upper) and HRMS spectra (lower) for cage **C8$_{[2+3]}$**

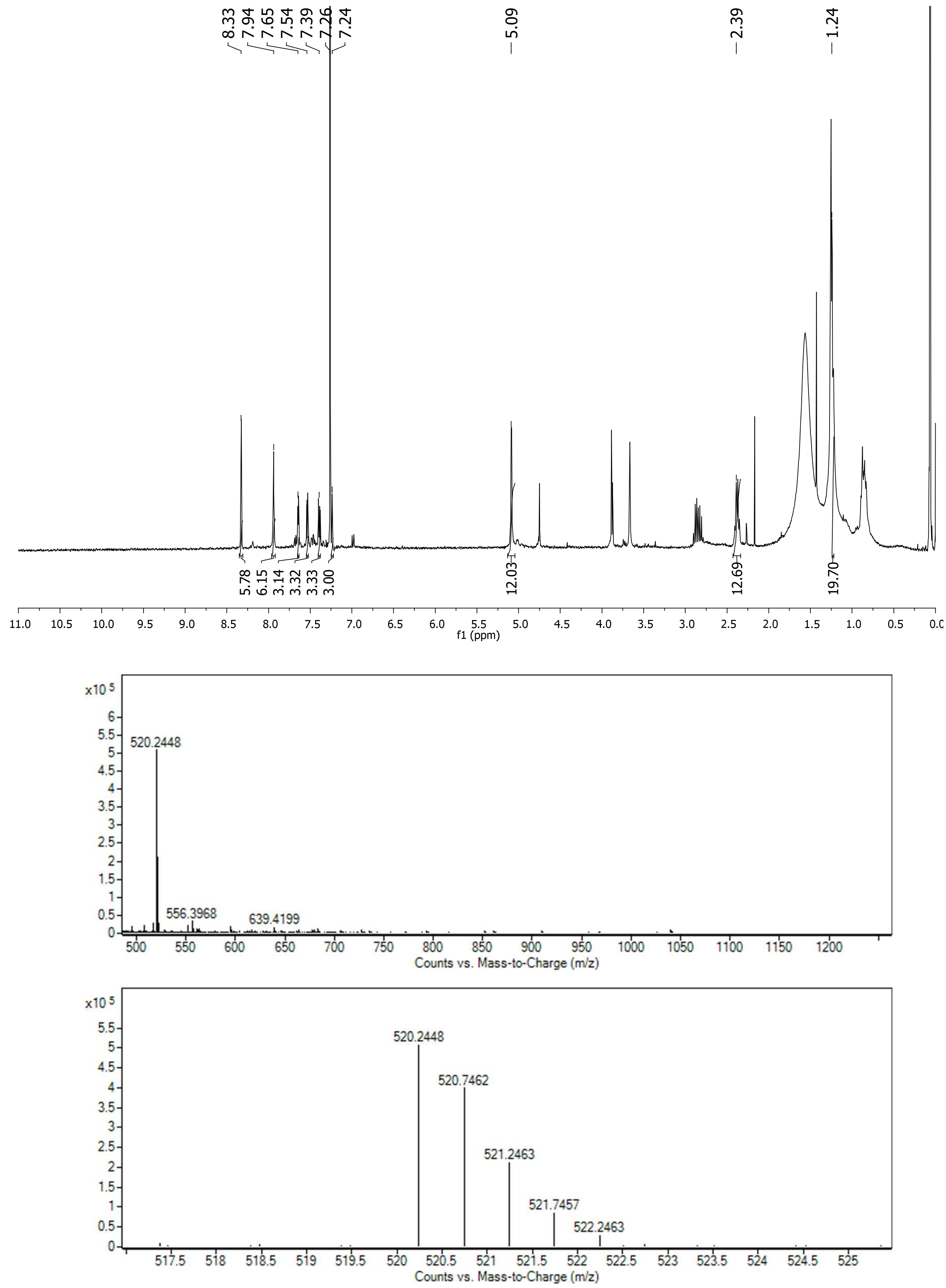

Supplementary Figure 101: ^{1}H NMR spectra ($CDCl_3$, upper) and HRMS spectra (lower) for cage **C9$_{[2+3]}$**

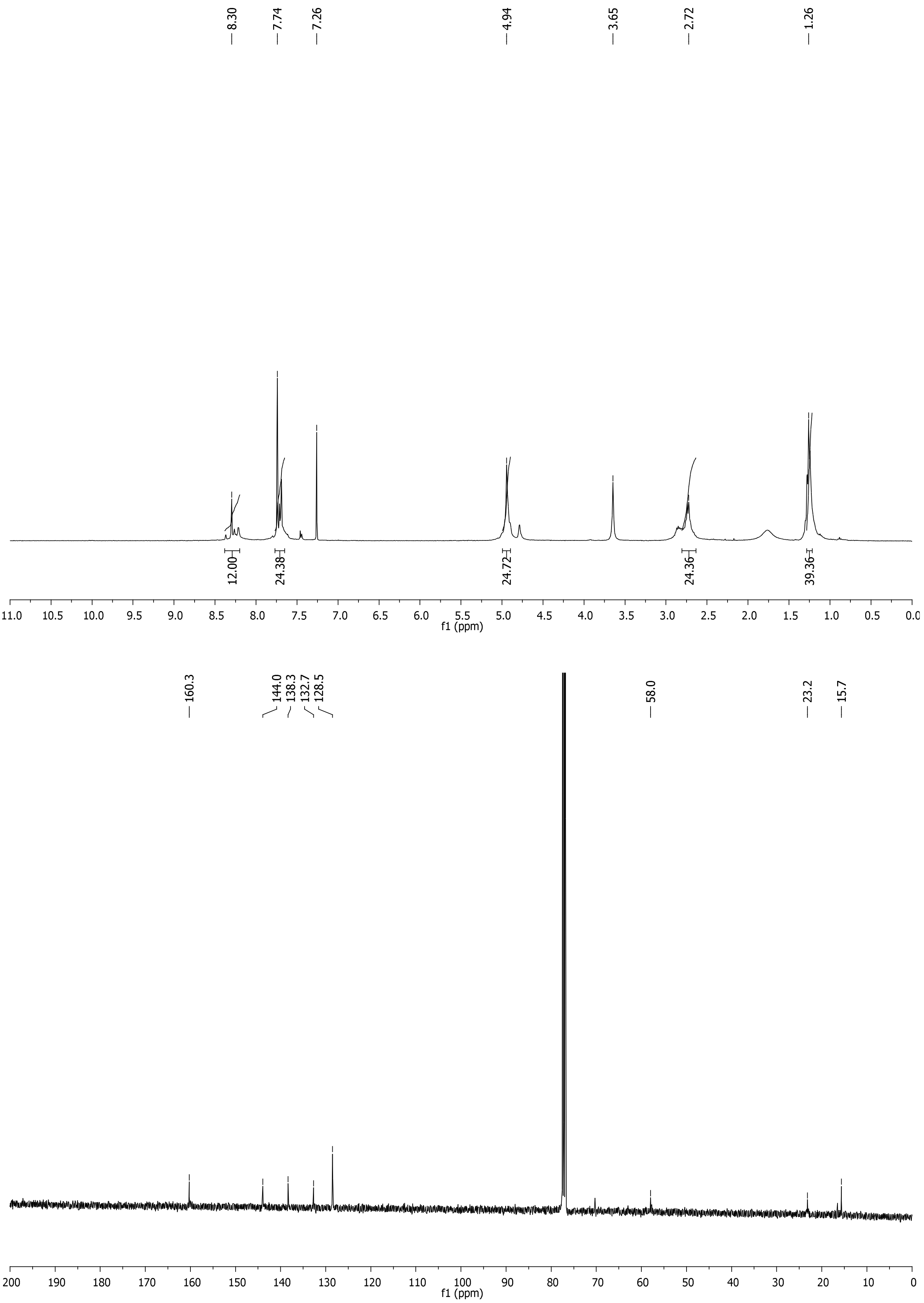

Supplementary Figure 102: ^{1}H NMR spectra ($CDCl_3$; upper) and ^{13}C NMR spectra ($CDCl_3$; lower) for cage **$C11_{[4+6]}$**

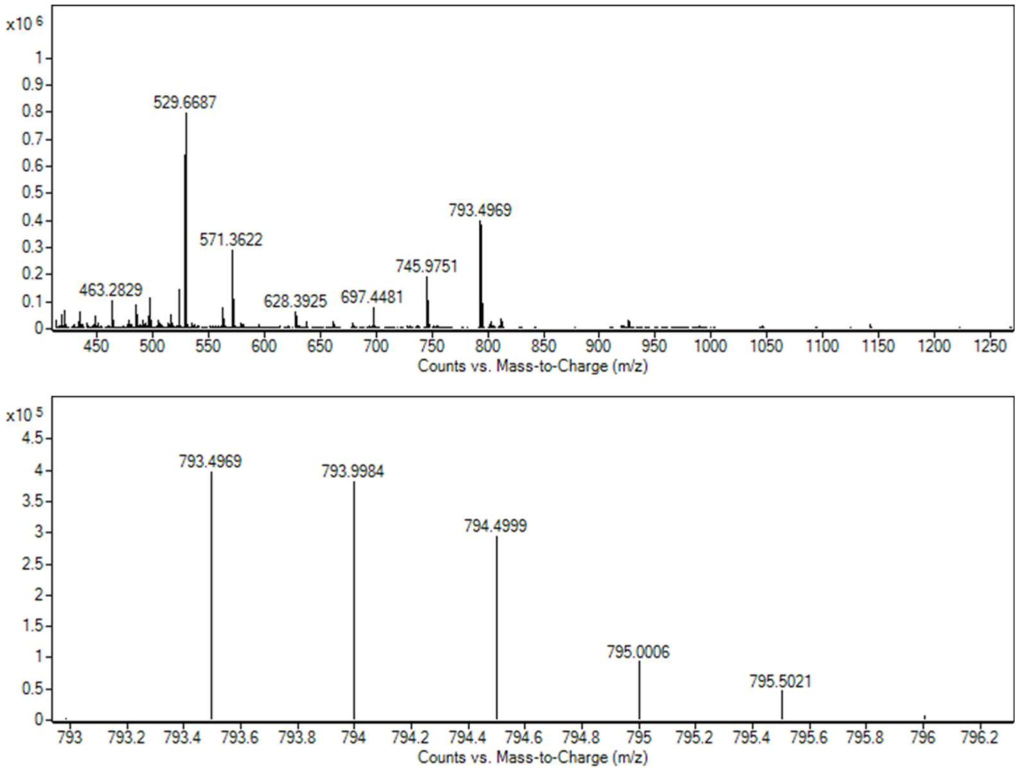

Supplementary Figure 103: HRMS spectra for cage **C11$_{[4+6]}$**

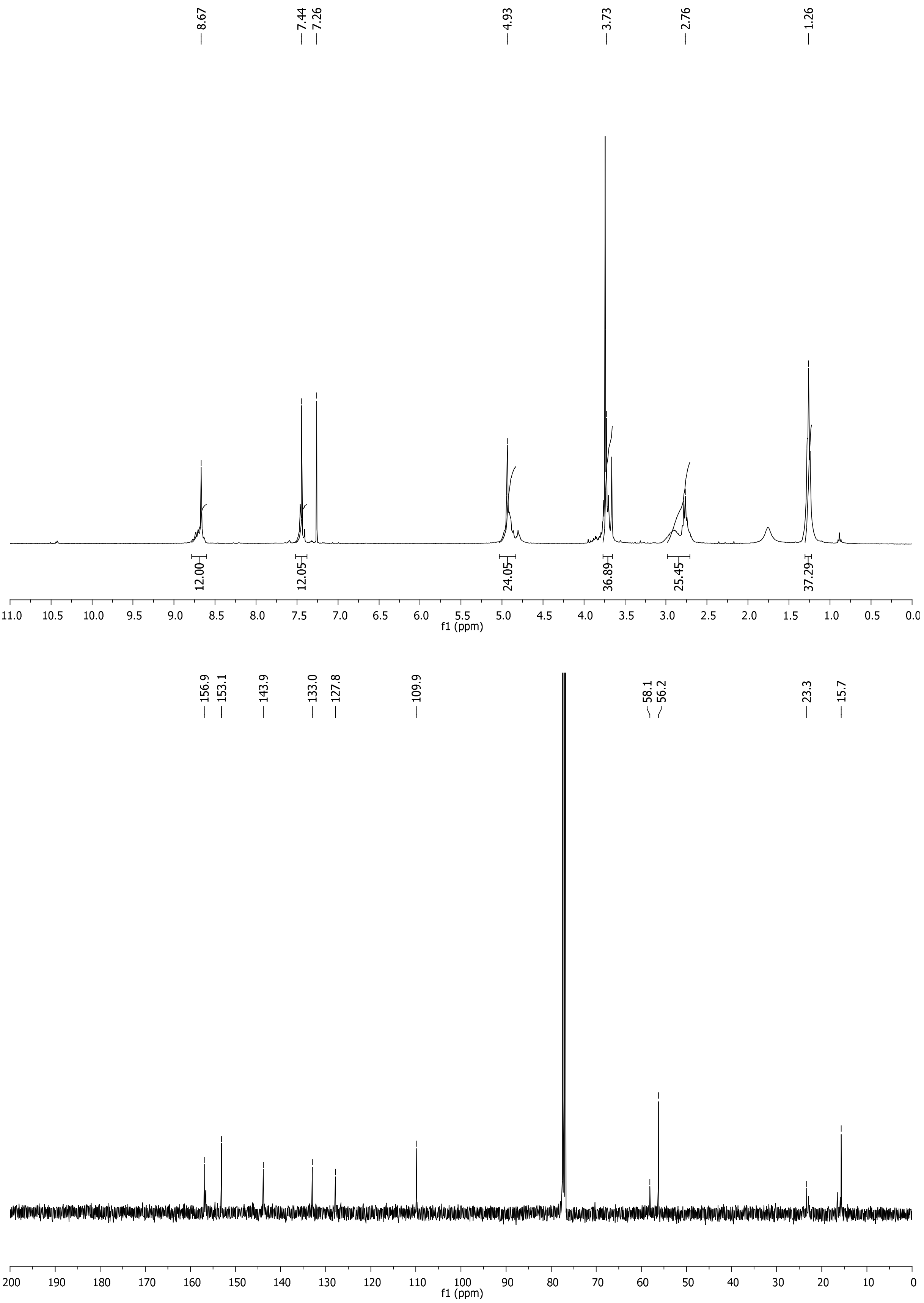

Supplementary Figure 104: ^{1}H NMR spectra ($CDCl_3$; upper) and ^{13}C NMR spectra ($CDCl_3$; lower) for cage **C13$_{[4+6]}$**

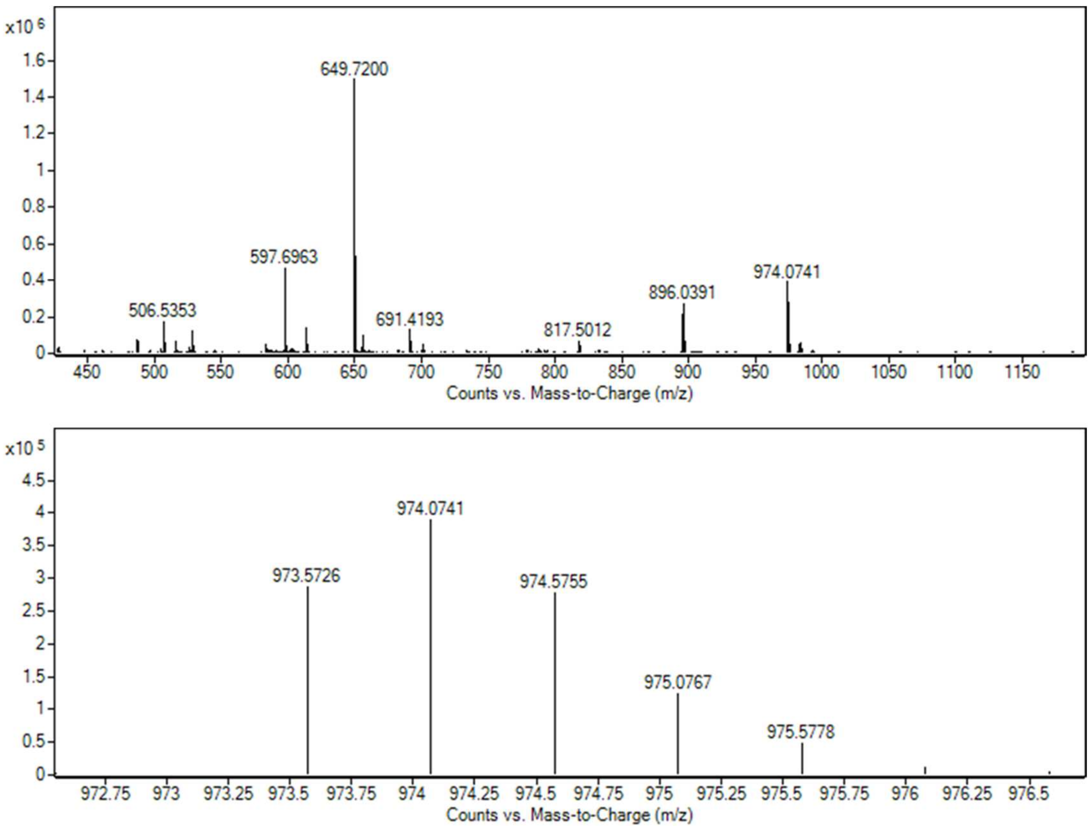

Supplementary Figure 105: HRMS spectra for cage **C13**$_{\mathbf{[4+6]}}$

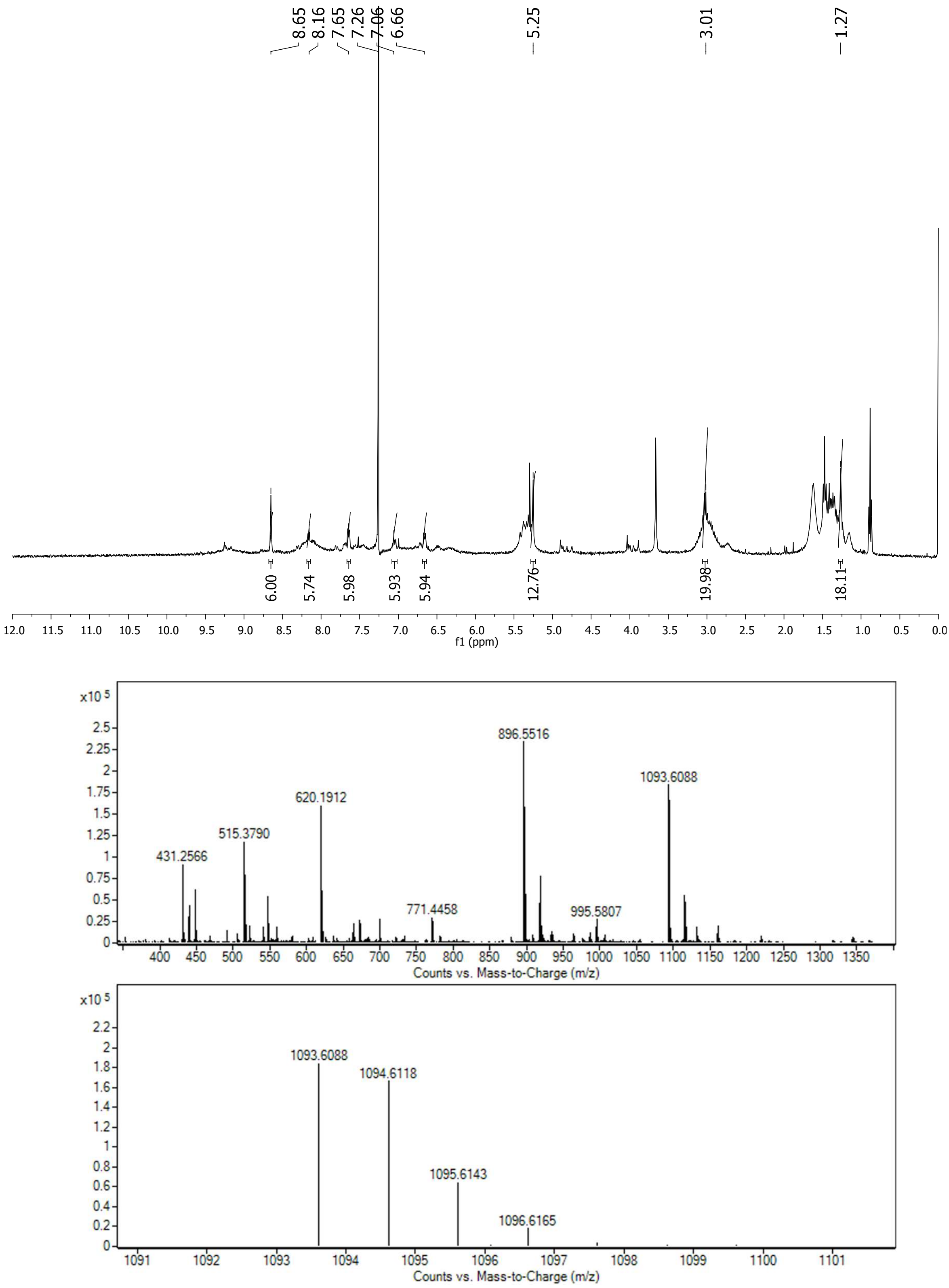

Supplementary Figure 106: ^{1}H NMR spectra ($CDCl_3$, upper) and HRMS spectra (lower) for cage **C14$_{[2+3]}$**

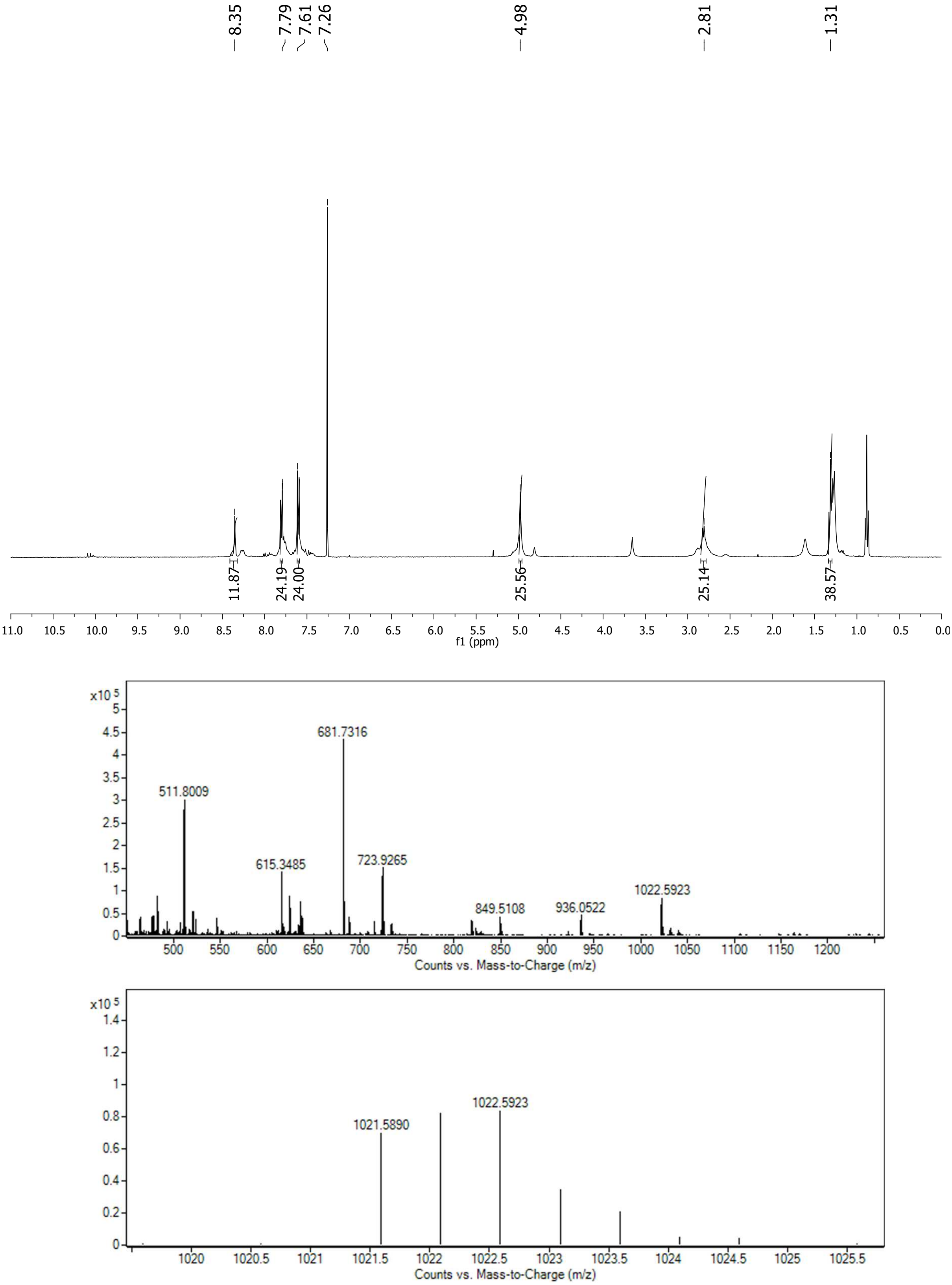

Supplementary Figure 107: ^{1}H NMR spectra ($CDCl_3$, upper) and HRMS spectra (lower) for cage **C15$_{[4+6]}$**

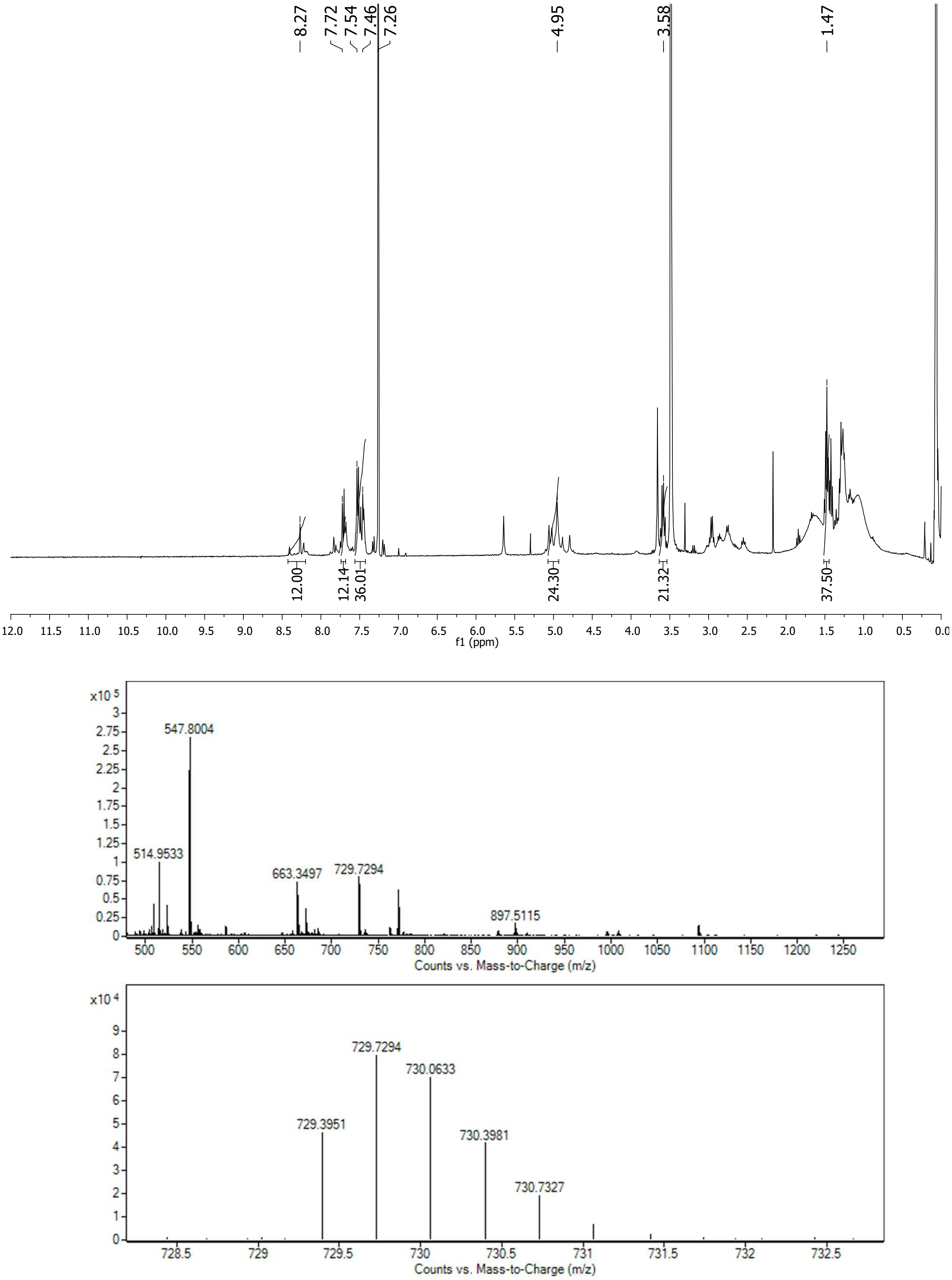

Supplementary Figure 108: ^{1}H NMR spectra ($CDCl_3$, upper) and HRMS spectra (lower) for cage **C18$_{[4+6]}$**

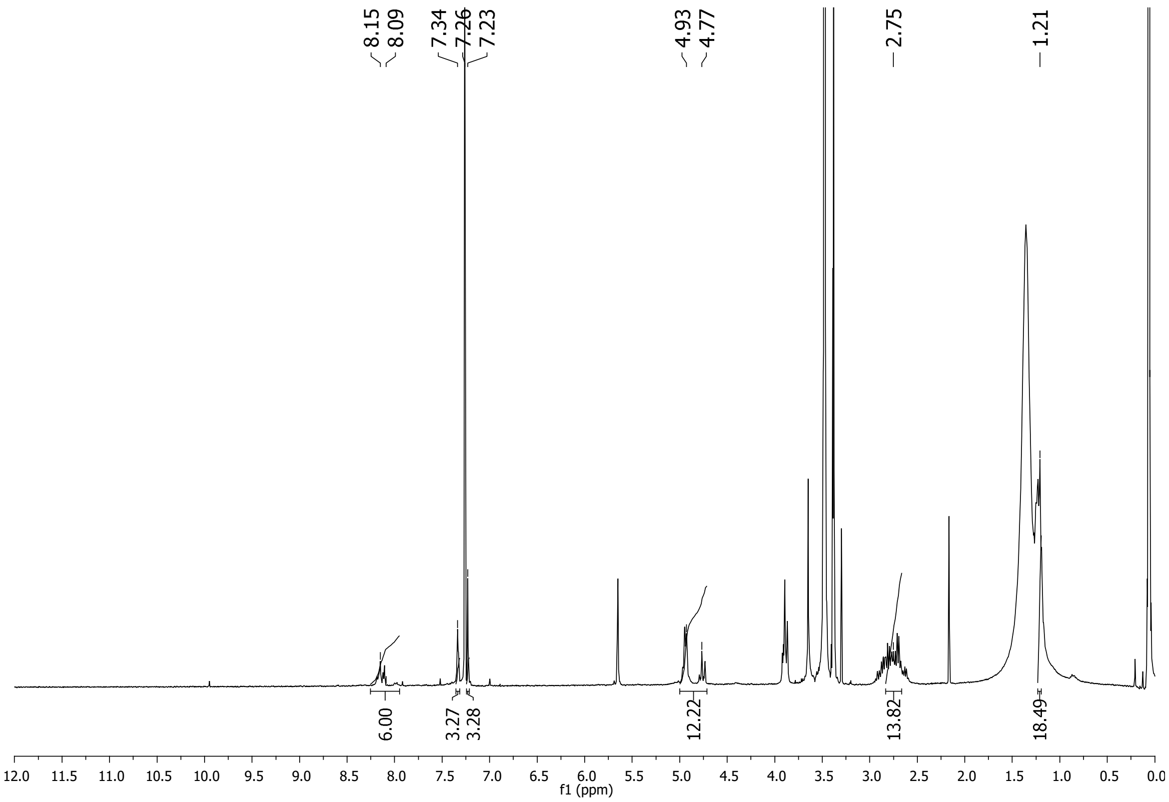

Supplementary Figure 109: ^{1}H NMR spectra ($CDCl_3$) for cage **$C20_{[2+3]}$**

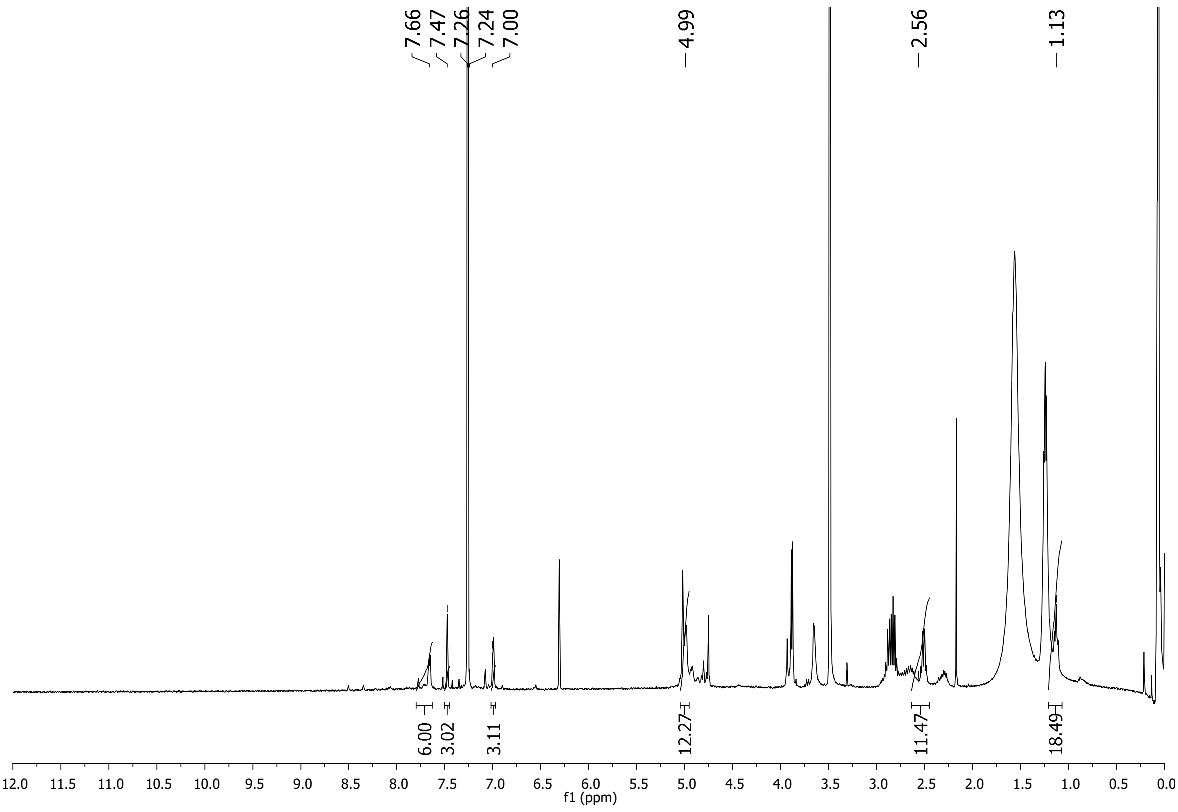

Supplementary Figure 110: ^{1}H NMR spectra ($CDCl_3$) for cage **$C21_{[2+3]}$**

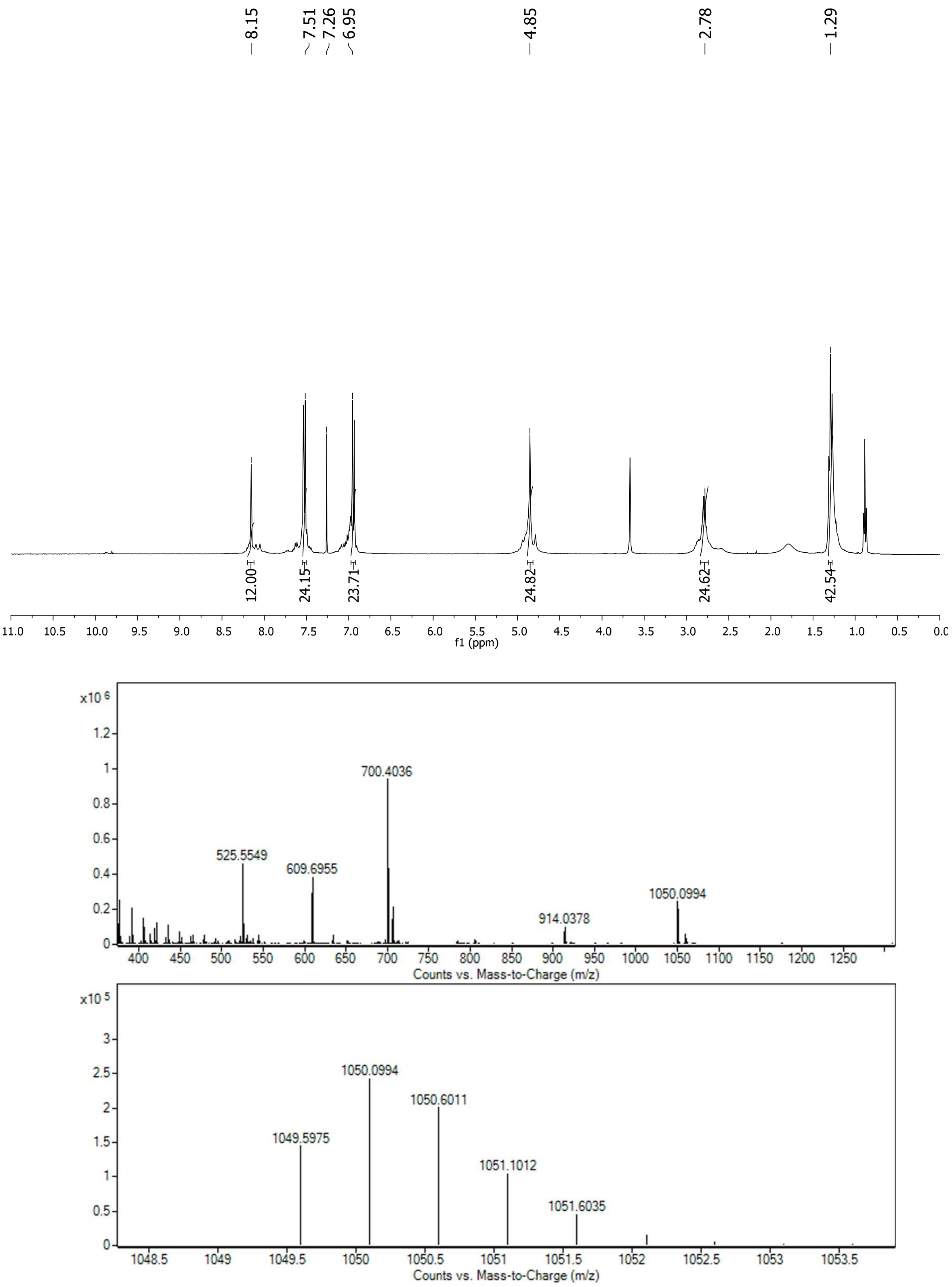

Supplementary Figure 111: ^{1}H NMR spectra ($CDCl_3$, upper) and HRMS spectra (lower) for cage **C23$_{[4+4]}$**

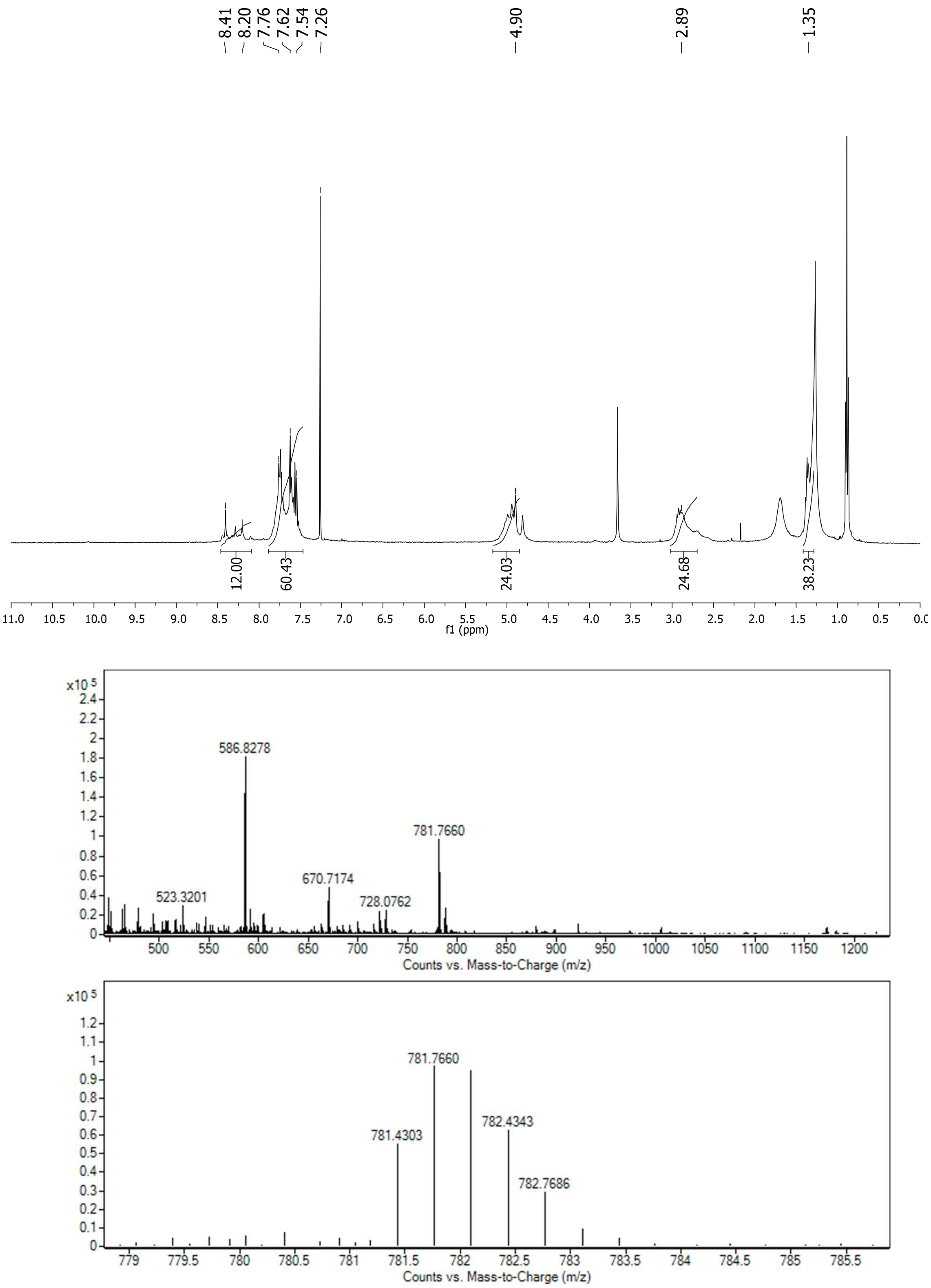

Supplementary Figure 112: ^{1}H NMR spectra ($CDCl_3$, upper) and HRMS spectra (lower) for cage **$C24_{[4+4]}$**

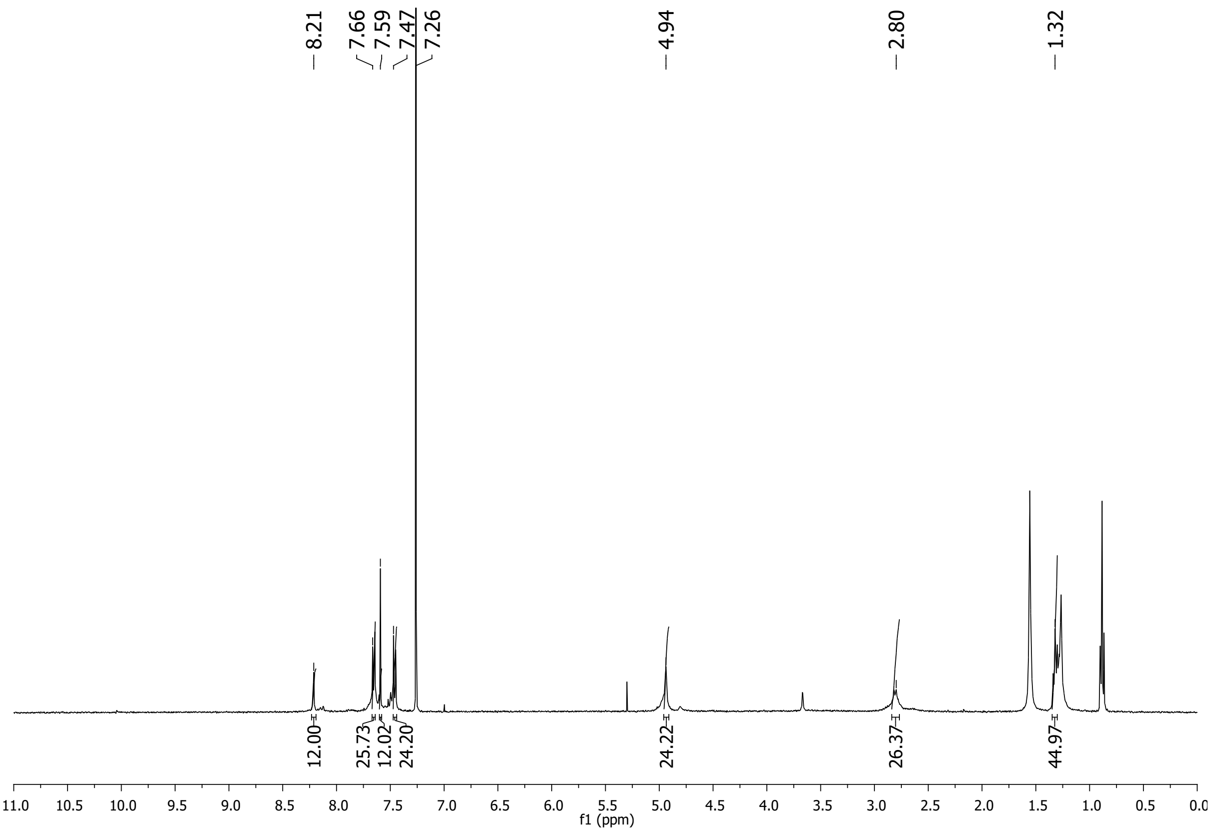

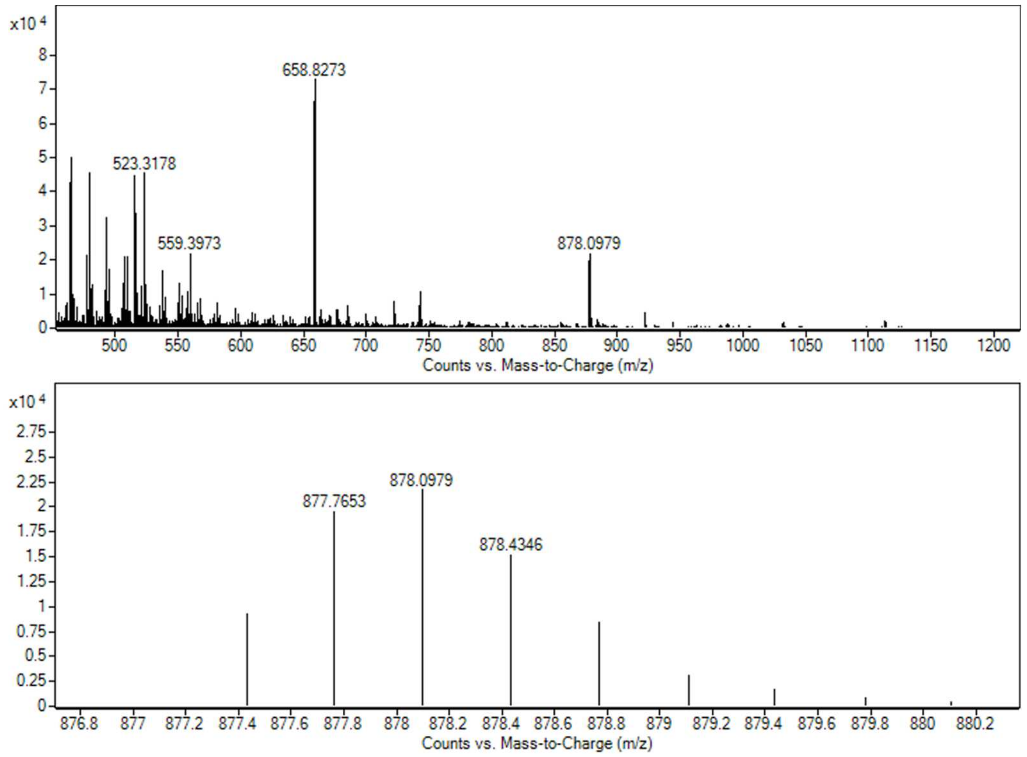

Supplementary Figure 113: ^{1}H NMR spectra ($CDCl_3$, upper) and HRMS spectra (lower) for cage **C25$_{[4+4]}$**

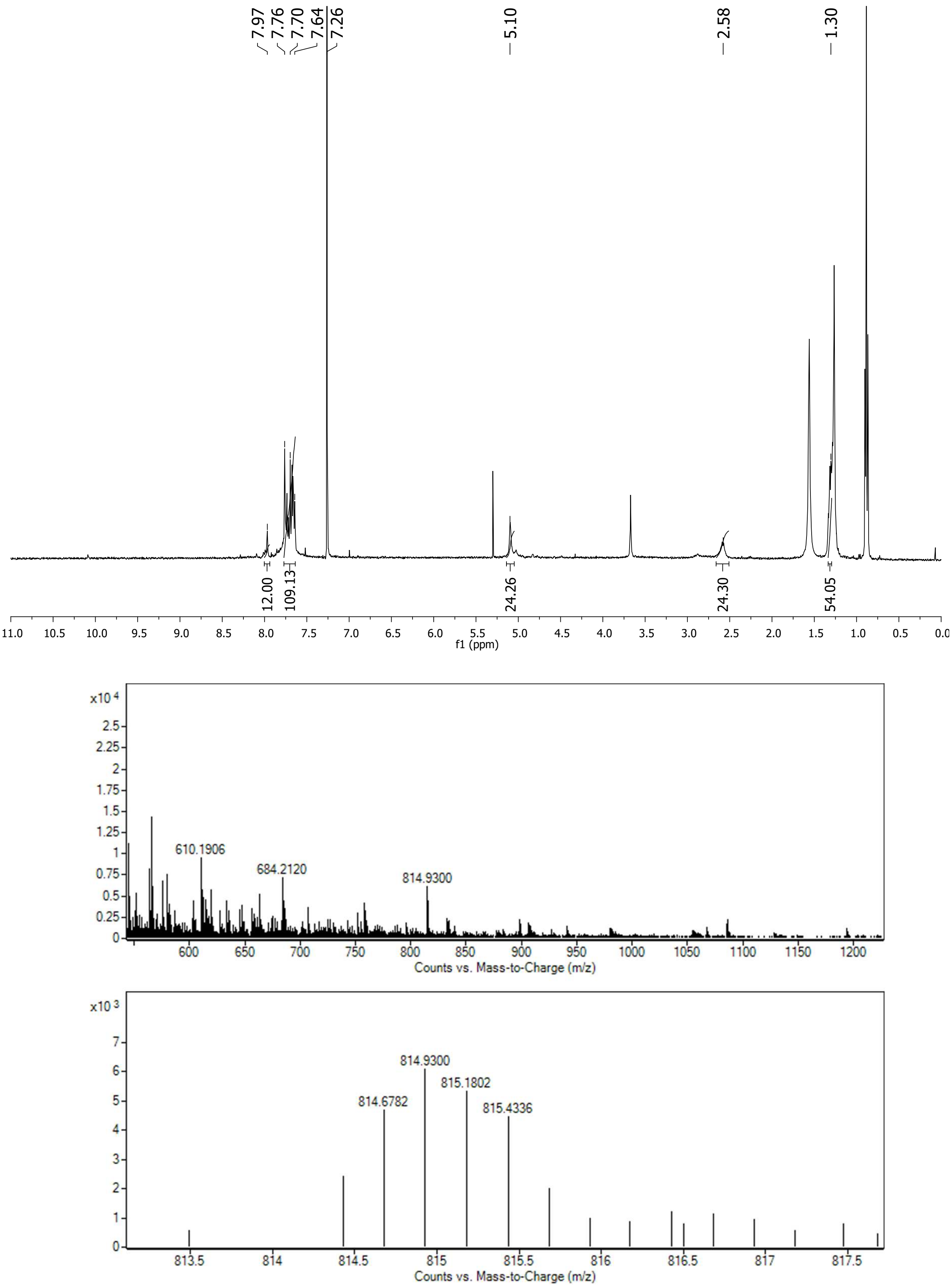

Supplementary Figure 114: ^{1}H NMR spectra ($CDCl_3$, upper) and HRMS spectra (lower) for cage **$C26_{[4+4]}$**

Supplementary Table 1: Formation energies and void diameters of **Tri^{2}Di^3** targeted cages modelled for precursors **A**/**B**/**C** with precursors **1**–**10**, excepting **7**.

Cage	Formation energy per bond formed (kJ mol^{-1})	Void Diameter (Å)
A1 – Tri^{2}Di^3	-10.8	1.7
B1 – Tri^{2}Di^3	-17.8	1.8
C1 – Tri^{2}Di^3	-17.5	1.7
A2 – Tri^{2}Di^3	-8.4	1.6
B2 – Tri^{2}Di^3	-20.3	1.7
C2 – Tri^{2}Di^3	-20.2	1.6
A3 – Tri^{2}Di^3	-13.2	1.6
B3 – Tri^{2}Di^3	-20.2	1.8
C3 – Tri^{2}Di^3	-19.9	1.7
A4 – Tri^{2}Di^3	-10.9	1.7
B4 – Tri^{2}Di^3	-18.1	1.8
C4 – Tri^{2}Di^3	-17.9	1.6
A5 – Tri^{2}Di^3	-15.4	1.6
B5 – Tri^{2}Di^3	-22.4	1.7
C5 – Tri^{2}Di^3	-22.1	1.6
A6 – Tri^{2}Di^3	-11.5	1.6
B6 – Tri^{2}Di^3	-18.5	1.7
C6 – Tri^{2}Di^3	-18.3	1.6
A8 – Tri^{2}Di^3	-16.4	1.5
B8 – Tri^{2}Di^3	-23.5	1.8
C8 – Tri^{2}Di^3	-23.3	1.6
A9 – Tri^{2}Di^3	-11.5	1.5
B9 – Tri^{2}Di^3	-18.5	1.7
C9 – Tri^{2}Di^3	-18.3	1.6
A10 – Tri^{2}Di^3	-4.0	2.3
B10 – Tri^{2}Di^3	-4.2	4.5
C10 – Tri^{2}Di^3	-1.0	3.7

Supplementary Table 2: Formation energies and void diameters of **Tri^{4}Di^6** cages modelled for precursors **A**/**B**/**C** with precursors **11**/**13**/**15**–**19** (excepting **A13** and **A17**), and **Tri^{4}Tri^4** cages modelled for precursors **A**/**B**/**C** with precursors **22**–**26**.

Cage	Formation energy per bond formed (kJ mol^{-1})	Void Diameter (Å)
A11 – Tri^{4}Di^6	-9.8	10.8
B11 – Tri^{4}Di^6	-13.7	6.0
C11 – Tri^{4}Di^6	-12.8	6.0
B13 – Tri^{4}Di^6	-12.9	3.4
C13 – Tri^{4}Di^6	-11.7	3.4
A15 – Tri^{4}Di^6	-6.5	11.5
B15 – Tri^{4}Di^6	-11.5	9.6
C15 – Tri^{4}Di^6	-10.2	9.5
A16 – Tri^{4}Di^6	-31.7	12.1
B16 – Tri^{4}Di^6	-37.4	9.7
C16 – Tri^{4}Di^6	-35.1	9.2
B17 – Tri^{4}Di^6	-18.2	7.7
C17 – Tri^{4}Di^6	-15.9	8.4
A18 – Tri^{4}Di^6	-7.4	14.8
B18 – Tri^{4}Di^6	-10.4	11.2
C18 – Tri^{4}Di^6	-10.9	10.8
A19 – Tri^{4}Di^6	-5.7	13.1
B19 – Tri^{4}Di^6	-11.3	12.6
C19 – Tri^{4}Di^6	-9.6	12.9
A22 – Tri^{4}Tri^4	-10.0	7.1
B22 – Tri^{4}Tri^4	-9.5	7.3
C22 – Tri^{4}Tri^4	-10.0	7.3
A23 – Tri^{4}Tri^4	-5.2	8.8
B23 – Tri^{4}Tri^4	-8.2	9.3
C23 – Tri^{4}Tri^4	-8.1	9.1
A24 – Tri^{4}Tri^4	-6.4	10.7
B24 – Tri^{4}Tri^4	-10.7	8.4
C24 – Tri^{4}Tri^4	-11.1	8.3
A25 – Tri^{4}Tri^4	-6.5	13.2
B25 – Tri^{4}Tri^4	-11.3	11.5
C25 – Tri^{4}Tri^4	-12.2	10.6
A26 – Tri^{4}Tri^4	-5.5	13.1
B26 – Tri^{4}Tri^4	-13.6	12.3
C26 – Tri^{4}Tri^4	-15.4	11.1

Supplementary Table 3: Summary of the results from the high-throughput screen: **Red** indicates the reaction failed and no cage was formed; **Yellow** indicates cage formed, but either contains impurities, is a complex mixture with oligomers or aldehyde starting material remains; **Green** indicates clear cage formation based on ^{1}H NMR analysis. The subscript **[X+Y]** indicates which cage stoichiometry is apparent for the successful reactions based on HRMS/diffusion NMR measurements, where **X** is the equivalents of triamine and **Y** is the equivalents of aldehyde that make up the cage.

	A	B	C		A	B	C
1	A1$_{[2+3]}$	B1$_{[2+3]}$	C1$_{[2+3]}$	14	A14	B14$_{[4+6]}$ & [2+3] mixture	C14$_{[2+3]}$
2	A2	B2$_{[2+3]}$	C2$_{[2+3]}$	15	A15	B15$_{[4+6]}$	C15$_{[4+6]}$
3	A3	B3	C3	16	A16	B16	C16
4	A4$_{[2+3]}$	B4$_{[2+3]}$	C4$_{[2+3]}$	17	A17	B17	C17
5	A5$_{[2+3]}$	B5$_{[2+3]}$	C5$_{[2+3]}$	18	A18	B18$_{[4+6]}$	C18$_{[4+6]}$
6	A6$_{[2+3]}$	B6$_{[2+3]}$	C6$_{[2+3]}$	19	A19	B19	C19
7	A7	B7$_{[2+3]}$	C7$_{[2+3]}$	20	A20	B20$_{[2+3]}$	C20$_{[2+3]}$
8	A8	B8$_{[2+3]}$	C8$_{[2+3]}$	21	A21	B21$_{[2+3]}$	C21$_{[2+3]}$
9	A9	B9$_{[2+3]}$	C9$_{[2+3]}$	22	A22$_{[4+4]}$	B22	C22
10	A10$_{[2+3]}$	B10$_{[2+3]}$	C10$_{[2+3]}$	23	A23	B23$_{[4+4]}$	C23$_{[4+4]}$
11	A11$_{[4+6]}$	B11$_{[4+6]}$	C11$_{[4+6]}$	24	A24	B24$_{[4+4]}$	C24$_{[4+4]}$
12	A12$_{[2+3]}$	B12	C12$_{[2+3]}$	25	A25	B25$_{[4+4]}$	C25$_{[4+4]}$
13	A13	B13$_{[4+6]}$	C13$_{[4+6]}$	26	A26	B26$_{[4+4]}$	C26$_{[4+4]}$

NB. **A/B/C16** and **B/C19** – possible cage formation was apparent in the ^{1}H NMR spectra, but stoichiometry could not be determined by HRMS or diffusion NMR, so classified as **red** in this study.

Supplementary Table 4: Summary of measured diffusion co-efficients for a range of the targeted **Tri^2Di^3** cages. Diffusion NMR was carried out blind on the crude cage reactions taken directly from the high-throughput screen where ^1H NMR analysis indicated clear cage formation. Measurements were carried out according to the general methods section (see **Supplementary Methods**).

.

**Diffusion Co-efficients (D, 10^{-10} $m^2.s^{-1}$)**							
**Peak**	**A10**	**B1**	**B2**	**B4**	**C1**	**C2**	**C4**
1	3.776	6.786	6.548	5.61	6.267	6.078	5.317
2	3.799	6.744	6.473	5.598	6.347	6.441	5.336
3	3.704	8.85	6.927	5.6	6.498	6.07	5.378
4	4.121	6.735	6.611	5.449	6.243	6.111	5.326
5	-	6.71	6.445	5.124	6.220	6.11	5.46
**Average**	**3.85**	**7.165**	**6.6008**	**5.4762**	**6.371**	**6.1620**	**5.3634**
SD	0.16	0.843	0.173	0.186	0.110	0.1405	0.0526

Supplementary Table 5: Calculated solvodynamic radii (R_S) for a range of the targeted **Tri²Di³** cages using the Stokes-Einstein equation with the measured diffusion co-efficients and the viscosity of chloroform (0.542 cP at 25 °C).

**Solvodynamic Radius (R_S, nm)**							
**Peak**	**A10**	**B1**	**B2**	**B4**	**C1**	**C2**	**C4**
1	1.0665	0.5935	0.6150	0.7179	0.6426	0.6626	0.7574
2	1.0601	0.5971	0.6221	0.7194	0.6345	0.6252	0.7547
3	1.0872	0.4550	0.5814	0.7191	0.6198	0.6635	0.7488
4	0.9772	0.5979	0.6092	0.7391	0.6451	0.6590	0.7561
5	-	0.6002	0.6249	0.7859	0.6475	0.6591	0.7376
**Average**	**1.0478**	**0.5688**	**0.6105**	**0.7363**	**0.6379**	**0.6539**	**0.7509**
SD	0.0419	0.0569	0.0156	0.0260	0.0116	0.0144	0.0073

Supplementary Table 6: Summary of measured diffusion co-efficients for a range of the targeted **Tri⁴Di⁶** cages. Diffusion NMR was carried out blind on the crude cage reactions taken directly from the high-throughput screen where ^{1}H NMR analysis indicated clear cage formation. Measurements were carried out according to the general methods section (see **Supplementary Methods**).

	**Diffusion Co-efficients (D, 10^{-10} $m^2.s^{-1}$)**									
**Peak**	**A11**	**B11**	**B13**	**B15**	**B18**	**B20**	**C11**	**C15**	**C20**	**C21**
1	5.677	4.789	4.466	4.118	3.976	6.315	4.53	3.918	5.425	5.764
2	6.051	4.796	4.436	4.082	3.934	7.259	4.56	3.901	5.582	6.085
3	5.309	4.832	4.365	4.087	3.918	6.361	4.66	3.975	5.475	6.025
4		4.823	4.386		3.800		4.69	3.937		5.713
5			4.406		3.893		5.02			
**Average**	**5.68**	**4.810**	**4.412**	**4.096**	**3.904**	**6.645**	**4.69**	**3.931**	**5.494**	**5.897**
SD	0.30	0.018	0.036	0.016	0.059	0.435	0.18	0.030	0.065	0.161

Supplementary Table 7: Calculated solvodynamic radii (R_s) for a range of the targeted **Tri^{4}Di^6** cages using the Stokes-Einstein equation with the measured diffusion co-efficients and the viscosity of chloroform (0.542 cP at 25 °C).

Solvodynamic Radius (R_s, nm)										
**Peak**	**A11**	**B11**	**B13**	**B15**	**B18**	**B20**	**C11**	**C15**	**C20**	**C21**
1	0.7094	0.8409	0.9017	0.9779	1.0129	0.6377	0.8898	1.0279	0.742	0.699
2	0.6655	0.8397	0.9078	0.9866	1.0237	0.5548	0.8826	1.0323	0.721	0.662
3	0.7586	0.8334	0.9226	0.9854	1.0279	0.6331	0.8640	1.0131	0.736	0.668
4		0.8350	0.9182		1.0598		0.8579	1.0229		0.705
5			0.9140		1.0345		0.8021			
**Average**	**0.7112**	**0.8373**	**0.9129**	**0.9833**	**1.0317**	**0.6085**	**0.8593**	**1.0241**	**0.7331**	**0.6835**
SD	0.0465	0.0031	0.0074	0.005	0.0157	0.0381	0.0133	0.0101	0.0087	0.0186

Supplementary Table 8: Summary of measured diffusion co-efficients for a range of the targeted **Tri⁴Tri⁴** cages. Diffusion NMR was carried out blind on the crude cage reactions taken directly from the high-throughput screen where ^{1}H NMR analysis indicated clear cage formation. Measurements were carried out according to the general methods section (see **Supplementary Methods**).

Diffusion Co-efficients (D, 10^{-10} $m^2.s^{-1}$)								
**Peak**	**B23**	**B24**	**B25**	**B26**	**C23**	**C24**	**C25**	**C26**
1	4.37	3.93	3.68	3.28	4.021	3.758	3.183	3.082
2	4.29	4.03	3.73	3.30	3.979	3.815	3.319	3.163
3	4.33	4.05	3.65	-	4.001	3.830	3.343	3.122
4	4.39	4.04	3.60	-	4.073	3.856	3.426	3.236
5	4.36	4.01	3.64	-	-	-	-	-
**Average**	**4.35**	**4.01**	**3.66**	**3.29**	**4.000**	**3.801**	**3.282**	**3.122**
SD	0.03	0.01	0.04	0.00	0.040	0.017	0.046	0.047

Supplementary Table 9: Calculated solvodynamic radii (R_S) for a range of the targeted **Tri⁴Tri⁴** cages using the Stokes-Einstein equation with the measured diffusion co-efficients and the viscosity of chloroform (0.542 cP at 25 °C).

Solvodynamic Radius (R_S, nm)								
**Peak**	**B23**	**B24**	**B25**	**B26**	**C23**	**C24**	**C25**	**C26**
1	0.9215	1.0255	1.0943	0.9215	1.0015	1.0716	1.2652	1.3067
2	0.9383	1.0003	1.0791	0.9215	1.0121	1.0556	1.2134	1.2732
3	0.9298	0.9936	1.1048	-	1.0065	1.0515	1.2047	1.2899
4	0.9176	0.9978	1.1187	-	0.9887	1.0444	1.1755	1.2445
5	0.9241	1.0033	1.1073	-	-	-	-	-
**Average**	**0.9263**	**1.0041**	**1.1008**	**1.2244**	**1.0022**	**1.0558**	**1.2147**	**1.2786**
SD	0.0084	0.0168	0.0130	0.0032	0.0053	0.0106	0.0327	0.0167

Supplementary Table 10: Summary of calculated pore sizes, OPT pore sizes (see description in **Supplementary Note 5**), average diameters, and maximum outer diameters, for a range of the targeted **Tri^2Di^3**, **Tri^4Di^6**, and **Tri^4Tri^4** cages, compared to the experimentally measured solvodynamic diameters (see **Supplementary Tables 5, 7, and 9**).

Calculated Sizes (nm)					Experimentally Measured Solvodynamic Diameter (nm)	Δ(CALC-EXP)
**Cage**	**Calculated Pore Size**	**Calculated OPT Pore Size**	**Calculated Average Diameter**	**Calculated Maximum Outer Diameter**		
**B1**	0.22	0.22	1.24	1.52	1.14	0.10
**B2**	0.22	0.22	1.27	1.75	1.22	0.05
**B4**	0.22	0.22	1.35	2.52	1.47	-0.12
**C1**	0.21	0.21	1.27	1.73	1.28	-0.01
**C2**	0.08	0.21	1.27	2.16	1.31	-0.04
**C4**	0.09	0.23	1.35	2.76	1.50	-0.15
**A11**	1.09	1.09	1.81	2.02	1.42	0.39
**B11**	0.63	0.63	1.85	2.09	1.67	0.18
**B13**	0.35	0.38	1.65	2.49	1.83	-0.18
**B15**	0.98	0.99	2.22	2.55	1.97	0.25
**B18**	1.20	1.21	2.46	2.81	2.06	0.40
**C11**	0.63	0.63	1.90	2.32	1.72	0.18
**C15**	0.99	1.00	2.25	2.76	2.05	0.20
**B23**	0.96	0.96	1.93	2.25	1.85	0.08
**B24**	0.88	1.00	2.07	2.50	2.01	0.06
**B25**	1.21	1.24	2.35	2.99	2.20	0.15
**B26**	1.20	1.22	2.43	3.30	2.45	-0.02
**C23**	0.76	0.86	1.92	2.46	2.00	-0.08
**C24**	0.83	0.90	2.06	2.68	2.11	-0.05
**C25**	1.14	1.20	2.37	3.13	2.43	-0.06
**C26**	1.16	1.18	2.41	3.43	2.56	-0.15

Supplementary Table 11: Summary of calculated pore sizes, OPT pore sizes (see description in **Supplementary Note 5**), average diameters, and maximum outer diameters, for different stoichiometries of **B20**, **C20** and **C21**.

Calculated Sizes (nm)					
**Cage**	**Topology**	**Calculated Pore Size**	**Calculated OPT Pore Size**	**Calculated Average Diameter**	**Calculated Maximum Outer Diameter**
**B20**	**Tri^2Di^3**	0.14	0.19	1.14	1.78
	**Tri^4Di^6**	0.91	0.91	2.00	2.30
**C20**	**Tri^2Di^3**	0.16	0.21	1.19	2.00
	**Tri^4Di^6**	0.88	0.90	2.04	2.51
**C21**	**Tri^2Di^3**	0.14	0.19	1.20	1.97
	**Tri^4Di^6**	1.05	1.06	2.07	2.53

Supplementary Table 12: Formation energies and void diameters of **Tri^2Di^3** and **Tri^4Di^6** cages modelled for precursors **A**/**B**/**C** with precursors **12**, **14**, **20** and **21** (excepting **A14**). An empty entry for void diameter corresponds to a cage with no cavity.

Cage	Formation energy per bond formed (kJ mol^{-1})	Void Diameter (Å)
A12 – Tri^2Di^3	-55.4	2.9
A12 – Tri^4Di^6	-56.3	10.6
B12 – Tri^2Di^3	-56.1	2.8
B12 – Tri^4Di^6	-57.9	7.0
C12 – Tri^2Di^3	-55.7	2.2
C12 – Tri^4Di^6	-57.9	6.9
B14 – Tri^2Di^3	-19.7	-
B14 – Tri^4Di^6	-23.7	3.1
C14 – Tri^2Di^3	-20.1	-
C14 – Tri^4Di^6	-23.5	3.3
B20 – Tri^2Di^3	-15.6	1.0
B20 – Tri^4Di^6	-17.4	8.7
C20 – Tri^2Di^3	-16.3	1.7
C20 – Tri^4Di^6	-16.4	8.6
A21 – Tri^2Di^3	-13.9	<1
A21 – Tri^4Di^6	-9.8	10.8
B21 – Tri^2Di^3	-15.5	1.4
B21 – Tri^4Di^6	-15.7	8.5
C21 – Tri^2Di^3	-20.8	1.6
C21 – Tri^4Di^6	-14.1	8.2

Supplementary Table 13: The RMSD between the SCXRD structures of each cage, where available, and the computed lowest energy conformation. To compare the geometry of the calculated models to the SCXRD structures, overlays of the models and single molecules extracted from the SCXRD were generated using MacroModel and their root-mean-square-deviation (RMSD) between all non-hydrogen atoms calculated. To ascertain the contribution to crystal packing effects on the geometry of the molecules, we also geometry optimised, in the gas phase, single molecules from the SCXRD structure using OPLS3, as described in the **Supplementary Methods**, and calculated the RMSD between these optimised structures and the computed structures. The latter should help remove the effects of crystal packing on the molecular conformations of the cages.

Cage	RMSD for comparison to SCXRD structure (Å)	RMSD for comparison to geometry optimised SCXRD structure (Å)
**B1 Tri^2Di^3**	0.25	0.00
**C1 Tri^2Di^3**	0.12	0.14
**B2 Tri^2Di^3**	0.32	0.07
**C2 Tri^2Di^3**	0.21	0.07
**B9 Tri^2Di^3**	0.34	0.12
**C9 Tri^2Di^3**	0.52	0.48
**B11 Tri^4Di^6**	1.07	1.00
**C14 Tri^2Di^3**	1.74	0.48
**B15 Tri^4Di^6**	1.23	0.97
**C21 Tri^2Di^3**	0.75	0.58
**C21 Tri^4Di^6**	0.91	0.90
**B23 Tri^4Tri^4**	1.12	0.94
**C23 Tri^4Tri^4**	1.10	1.07
**B24 Tri^4Tri^4**	1.11	1.03
**B26 Tri^4Tri^4**	1.24	1.09
**C26 Tri^4Tri^4**	1.76	1.62

Supplementary Table 14: Energetic preferences for unexpected cage topologies. For the set of molecules that unexpectedly formed **Tri^{2}Di^3** rather than the targeted **Tri^{4}Di^6** topology, we additionally modelled the **Tri^{2}Di^3** topology to allow comparison of the relative energies of the two possible outcomes. Preference for **Tri^{4}Di^6** over **Tri^{2}Di^3** topology, where a negative value indicates an energetic preference for **Tri^{4}Di^6** and a positive value an energetic preference for **Tri^{2}Di^3** topology.

Cage	Relative energy (kJ mol^{-1} per [2+3] unit)
A12	-5
C12	-13
B14	-24
C14	-22
B20	-11
C20	-3
B21	24
C21	35

Supplementary Table 15: Summary of optimisation screen for cage formation between (2,4,6-trimethylbenzene-1,3,5-triyl)trimethanamine (**B**) and terephthalaldehyde (**11**). General cage formation screen: Terephthalaldehyde **11** (9.7 mg, 0.072 mmol, 3.0 eq.) and (2,4,6-trimethylbenzene-1,3,5-triyl)trimethanamine **B** (2.0–3.0 eq.) were dissolved in $CDCl_3$ (1–13 mL) and stirred at room temperature (RT) or 60 °C for 1–6 days. Reaction progress was monitored by ^{1}H NMR spectroscopy (see **Supplementary Fig. 43**) to determine whether any cage formation had occurred.

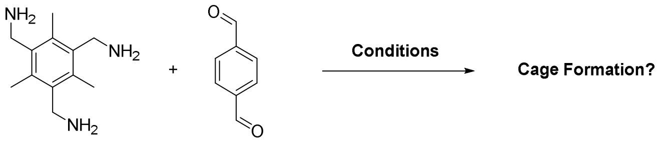

Reaction No.	Triamine (eq.)	$CDCl_3$ (mL)	Temp (°C)	Reaction Time (days)	Outcome
**1**	2.0	13	RT	6	Mainly SM – no clear sign of cage
**2**	2.0	13	60	3	SM + cage formation
**3**	2.5	13	60	3	Clean cage formation
**4**	3.0	13	60	3	Cage formation, but less pure than 3
**5**	2.0	10	60	1	↓ Increasing amounts of insoluble precipitate formed
**6**	2.0	5	60	1	
**7**	2.0	1	60	1	

Supplementary Table 16: Precursor stock solutions in $CDCl_3$ for high-throughput screening

Stock Solution Number	Reactant	MW (g mol^{-1})	Stock Solution Concentration (mg mL^{-1})	Stock Solution Concentration (mmol mL^{-1})
**1**	Triamine A	165.24	5	0.0303
**2**	Triamine B	207.32	5	0.0241
**3**	Triamine C	249.39	5	0.0200
**4**	Aldehyde 1	134.13	5	0.0373
**5**	Aldehyde 2	213.03	5	0.0235
**6**	Aldehyde 3	177.95	5	0.0281
**7**	Aldehyde 4	230.34	5	0.0217
**8**	Aldehyde 5	210.23	5	0.0238
**9**	Aldehyde 6	236.27	5	0.0212
**10**	Aldehyde 7	255.23	5	0.0196
**11**	Aldehyde 8	211.22	5	0.0237
**12**	Aldehyde 9	216.25	5	0.0231
**13**	Aldehyde 10	355.45	5	0.0141
**14**	Aldehyde 11	134.13	5	0.0373
**15**	Aldehyde 12	166.13	2.5	0.0150
**16**	Aldehyde 13	194.19	5	0.0257
**17**	Aldehyde 14	234.25	2.5	0.0107
**18**	Aldehyde 15	210.13	5	0.0238
**19**	Aldehyde 16	226.23	5	0.0221
**20**	Aldehyde 17	244.67	5	0.0204
**21**	Aldehyde 18	234.25	5	0.0213
**22**	Aldehyde 19	286.32	5	0.0175
**23**	Aldehyde 20	196.24	2.5	0.0127
**24**	Aldehyde 21	196.24	5	0.0255
**25**	Aldehyde 22	162.14	5	0.0308
**26**	Aldehyde 23	329.35	5	0.0152
**27**	Aldehyde 24	390.13	5	0.0128
**28**	Aldehyde 25	462.49	5	0.0108
**29**	Aldehyde 26	618.72	2.5	0.0040

Supplementary Table 17: Reaction volumes used in each reaction using triamine **A** on the Chemspeed Accelerator SLT-100 platform

Cage	Reaction Number	Triamine Reactant	Volume Triamine Stock Solution (mL)	Amount of Triamine (mmol)	Aldehyde Reactant	Ratio of equiv. used	Amount Aldehyde required (mmol)	Volume Aldehyde Stock Solution (mL)	Additional Amount of $CDCl_3$ (mL) added (Total Volume ≥13 mL)
**A1**	11	A	2	0.0605	1	3:3	0.0605	1.62	9.38
**A2**	43	A	2	0.0605	2	3:3	0.0605	2.58	8.42
**A3**	44	A	2	0.0605	3	3:3	0.0605	2.15	8.85
**A4**	45	A	2	0.0605	4	3:3	0.0605	2.79	8.21
**A5**	64	A	2	0.0605	5	3:3	0.0605	2.54	8.46
**A6**	65	A	2	0.0605	6	3:3	0.0605	2.86	8.14
**A7**	66	A	2	0.0605	7	3:3	0.0605	3.09	7.91
**A8**	67	A	2	0.0605	8	3:3	0.0605	2.56	8.44
**A9**	68	A	2	0.0605	9	3:3	0.0605	2.62	8.38
**A10**	42	A	2	0.0605	10	4:3	0.0807	5.74	5.26
**A11**	1	A	2	0.0605	11	6:5	0.0726	1.95	9.05
**A12**	38	A	2	0.0605	12	6:5	0.0726	4.83	6.17
**A13**	37	A	2	0.0605	13	6:5	0.0726	2.82	8.18
**A14**	39	A	2	0.0605	14	6:5	0.0726	6.80	4.20
**A15**	2	A	2	0.0605	15	6:5	0.0726	3.05	7.95
**A16**	5	A	2	0.0605	16	6:5	0.0726	3.29	7.71
**A17**	4	A	2	0.0605	17	6:5	0.0726	3.55	7.45
**A18**	33	A	2	0.0605	18	6:5	0.0726	3.40	7.60
**A19**	3	A	2	0.0605	19	6:5	0.0726	4.16	6.84
**A20**	40	A	2	0.0605	20	6:5	0.0726	5.70	5.30
**A21**	41	A	2	0.0605	21	3:2	0.0403	1.58	9.42
**A22**	6	A	2	0.0605	22	4:5	0.0484	1.57	9.43
**A23**	7	A	2	0.0605	23	4:5	0.0484	3.19	7.81
**A24**	9	A	2	0.0605	24	4:5	0.0484	3.78	7.22
**A25**	10	A	2	0.0605	25	4:5	0.0484	4.48	6.52
**A26**	8	A	2	0.0605	26	4:5	0.0484	11.98	0

Supplementary Table 18: Reaction volumes used in each reaction using triamine **B** on the Chemspeed Accelerator SLT-100 platform

Cage	Reaction Number	Triamine Reactant	Volume Triamine Stock Solution (mL)	Amount of Triamine (mmol)	Aldehyde Reactant	Ratio of equiv. used	Amount Aldehyde required (mmol)	Volume Aldehyde Stock Solution (mL)	Additional Amount of $CDCl_3$ (mL) added (Total Volume ≥13 mL)
**B1**	34	B	2.5	0.0603	1	3:3	0.0603	1.62	8.88
**B2**	52	B	2.5	0.0603	2	3:3	0.0603	2.57	7.93
**B3**	53	B	2.5	0.0603	3	3:3	0.0603	2.15	8.35
**B4**	54	B	2.5	0.0603	4	3:3	0.0603	2.78	7.72
**B5**	69	B	2.5	0.0603	5	3:3	0.0603	2.54	7.96
**B6**	70	B	2.5	0.0603	6	3:3	0.0603	2.85	7.65
**B7**	71	B	2.5	0.0603	7	3:3	0.0603	3.08	7.42
**B8**	72	B	2.5	0.0603	8	3:3	0.0603	2.55	7.95
**B9**	73	B	2.5	0.0603	9	3:3	0.0603	2.61	7.89
**B10**	51	B	2.5	0.0603	10	4:3	0.0804	5.71	4.79
**B11**	12	B	2.5	0.0603	11	6:5	0.0724	1.94	8.56
**B12**	47	B	2.5	0.0603	12	6:5	0.0724	4.81	5.69
**B13**	46	B	2.5	0.0603	13	6:5	0.0724	2.81	7.69
**B14**	48	B	2.5	0.0603	14	6:5	0.0724	6.78	3.72
**B15**	13	B	2.5	0.0603	15	6:5	0.0724	3.04	7.46
**B16**	16	B	2.5	0.0603	16	6:5	0.0724	3.27	7.23
**B17**	15	B	2.5	0.0603	17	6:5	0.0724	3.54	6.96
**B18**	35	B	2.5	0.0603	18	6:5	0.0724	3.39	7.11
**B19**	14	B	2.5	0.0603	19	6:5	0.0724	4.14	6.36
**B20**	49	B	2.5	0.0603	20	6:5	0.0724	5.68	4.82
**B21**	50	B	2.5	0.0603	21	3:2	0.0402	1.58	8.92
**B22**	17	B	2.5	0.0603	22	4:5	0.0482	1.56	8.94
**B23**	18	B	2.5	0.0603	23	4:5	0.0482	3.18	7.32
**B24**	20	B	2.5	0.0603	24	4:5	0.0482	3.76	6.74
**B25**	21	B	2.5	0.0603	25	4:5	0.0482	4.46	6.04
**B26**	19	B	2.5	0.0603	26	4:5	0.0482	11.94	0

Supplementary Table 19: Reaction volumes used in each reaction using triamine **C** on the Chemspeed Accelerator SLT-100 platform

Cage	Reaction Number	Triamine Reactant	Volume Triamine Stock Solution (mL)	Amount of Triamine (mmol)	Aldehyde Reactant	Ratio of equiv. used	Amount Aldehyde required (mmol)	Volume Aldehyde Stock Solution (mL)	Additional Amount of $CDCl_3$ (mL) added (Total Volume ≥13 mL)
**C1**	32	C	3	0.0601	1	3:3	0.0601	1.61	8.39
**C2**	61	C	3	0.0601	2	3:3	0.0601	2.56	7.44
**C3**	62	C	3	0.0601	3	3:3	0.0601	2.14	7.86
**C4**	63	C	3	0.0601	4	3:3	0.0601	2.77	7.23
**C5**	74	C	3	0.0601	5	3:3	0.0601	2.53	7.47
**C6**	75	C	3	0.0601	6	3:3	0.0601	2.84	7.16
**C7**	76	C	3	0.0601	7	3:3	0.0601	3.07	6.93
**C8**	77	C	3	0.0601	8	3:3	0.0601	2.54	7.46
**C9**	78	C	3	0.0601	9	3:3	0.0601	2.60	7.40
**C10**	60	C	3	0.0601	10	4:3	0.0802	5.70	4.30
**C11**	22	C	3	0.0601	11	6:5	0.0722	1.94	8.06
**C12**	56	C	3	0.0601	12	6:5	0.0722	4.80	5.20
**C13**	55	C	3	0.0601	13	6:5	0.0722	2.80	7.20
**C14**	57	C	3	0.0601	14	6:5	0.0722	6.76	3.24
**C15**	23	C	3	0.0601	15	6:5	0.0722	3.03	6.97
**C16**	26	C	3	0.0601	16	6:5	0.0722	3.27	6.73
**C17**	25	C	3	0.0601	17	6:5	0.0722	3.53	6.47
**C18**	36	C	3	0.0601	18	6:5	0.0722	3.38	6.62
**C19**	24	C	3	0.0601	19	6:5	0.0722	4.13	5.87
**C20**	58	C	3	0.0601	20	6:5	0.0722	5.67	4.33
**C21**	59	C	3	0.0601	21	3:2	0.0401	1.57	8.43
**C22**	27	C	3	0.0601	22	4:5	0.0481	1.56	8.44
**C23**	28	C	3	0.0601	23	4:5	0.0481	3.17	6.83
**C24**	30	C	3	0.0601	24	4:5	0.0481	3.75	6.25
**C25**	31	C	3	0.0601	25	4:5	0.0481	4.45	5.55
**C26**	29	C	3	0.0601	26	4:5	0.0481	11.91	0

Supplementary Table 20: Summary of precipitate formation in reaction solutions during high-throughput cage screening, where ✓✓ indicates a large amount of precipitate formed, ✓ a small amount, and × where no precipitate formed in the reaction solution. Generally, more insoluble precipitate was formed in the combinations involving triamine **A**, presumably due to the increased flexibility and reduced pre-organisation of the amines, and in combinations containing the larger aromatic aldehydes such as **18**, **19**, **24**, **25** and **26**.

	A	B	C		A	B	C
**1**	✓	×	×	**14**	✓	✓	×
**2**	✓	×	×	**15**	✓✓	✓	×
**3**	✓	×	×	**16**	✓	×	×
**4**	✓	×	×	**17**	✓	×	×
**5**	✓✓	✓	×	**18**	✓	✓	✓
**6**	✓✓	✓	×	**19**	✓	✓✓	×
**7**	✓✓	✓	×	**20**	✓	×	×
**8**	✓✓	×	×	**21**	✓	×	×
**9**	✓	×	×	**22**	✓	✓✓	×
**10**	×	×	×	**23**	✓✓	×	✓
**11**	✓	✓	×	**24**	✓✓	✓✓	✓✓
**12**	✓	✓	×	**25**	✓✓	✓✓	×
**13**	✓	✓	×	**26**	✓✓	✓✓	✓✓

Supplementary Table 21: Summary of characterisation data from high-throughput cage screening, using triamine **A**, on crude reaction solutions prior to any isolation.

	Characterisation prior to Isolation									Targeted Cage Size	Formed Cage Size (based on HRMS and/or diffusion NMR)?
	^{1}H NMR ($CDCl_3$)		HRMS		FTIR				Diffusion NMR		
	Clear Cage Formation?	Aldehyde SM?	Cage Present?	Mass ion?	C=O?	Stretch (cm^{-1})	C=N?	Stretch (cm^{-1})	Solvodynamic Diameter (nm)		
**A1**	✓[a]	×	✓	$[M+H]^+$ 625.3148	×	1689	✓	1645	n.d.	[2+3]	[2+3]
**A2**	×	✓	×	-	✓	1696	✓	1646	n.d.	[2+3]	-
**A3**	×	×	×	-	×	1703, 1687	✓	1646	n.d.	[2+3]	-
**A4**	✓[a]	×	✓	$[M+2H]^{2+}$ 457.2176	×	1706	✓	1645	n.d.	[2+3]	[2+3]
**A5**	✓[a]	×	✓	$[M+H]^+$ 853.4121	×	1716, 1695	✓	1644	n.d.	[2+3]	[2+3]
**A6**	✓[a]	×	✓	$[M+H]^+$ 931.4590[c]	×	1697	✓	1643	n.d.	[2+3]	[2+3]
**A7**	×	×	×	-	×	1700	✓	1644	n.d.	[2+3]	-
**A8**	×	×	×	-	×	1695	✓	1643	n.d.	[2+3]	-
**A9**	×	×	×	-	×	1710, 1697	✓	1642	n.d.	[2+3]	-
**A10**	✓[a]	✓	✓	$[M+2H]^{2+}$ 614.8863	✓	1687	✓	1643	n.d.	[2+3]	[2+3]
**A11**	✓	×	×	-	×	1690	✓	1643	1.42	[4+6]	[4+6]
**A12**	✓[a]	×	✓	$[M+H]^+$ 721.2757[c]	×	1679, 1688	✓	1630	n.d.	[4+6]	[2+3]
**A13**	×	✓	×	-	✓	1675	✓	1630	n.d.	[4+6]	-
**A14**	×	✓	×	-	✓	1672	✓	1636	n.d.	[4+6]	-
**A15**	×	×	×	-	×	1698	×	-	n.d.	[4+6]	-
**A16[e]**	✓[b]	×	×	-	×	1696, 1651	✓	1628	n.d.	[4+6]	-
**A17**	×	✓	×	-	✓	1703, 1685	✓	1641	n.d.	[4+6]	-
**A18**	×[d]	×	×	-	×	1697, 1687	✓	1643	n.d.	[4+6]	-
**A19**	×	✓	×	-	×	1679	✓	1647	n.d.	[4+6]	-
**A20**	×	✓	×	-	×	1658	✓	1627	n.d.	[4+6]	-
**A21**	×	✓	×	-	×	1669	✓	1628	n.d.	[4+6]	-
**A22**	×	×	✓	$[M+H]^+$ 1093.5246[c]	×	1694	✓	1645	n.d.	[4+4]	[4+4]
**A23**	×	✓	×	-	✓	1697	✓	1641	n.d.	[4+4]	-
**A24**	×	✓	×	-	✓	1699	✓	1642	n.d.	[4+4]	-
**A25**	×	✓	×	-	✓	1700	✓	1636-1653	n.d.	[4+4]	-
**A26**	×	✓	×	-	✓	1698, 1685	✓	1637-1652	n.d.	[4+4]	-

[a] Possibly cage present in ^{1}H NMR spectra, but reaction either a complex mixture with oligomers present, or aldehyde SM, where specified, or sample very weak; [b] Complex mixture by ^{1}H NMR analysis but the positional disorder of the hydroxyl-substituent or chloro-substituent may account for this; [c] Mass ion indicating cage formed, but present alongside a large number of other unknown masses; [d] Clean conversion to cage-like species observed by ^{1}H NMR spectroscopy, but two Ar-*H* species observed for the triamine component, and main mass ion instead consistent with clean formation of [2+2]-4H_2O species rather than the targeted [4+6] cage; [e] Possible cage formation apparent in the ^{1}H NMR spectra, but stoichiometry of cage could not be determined by HRMS or diffusion NMR; n.d. = not determined.

Supplementary Table 22: Summary of characterisation data from high-throughput cage screening, using triamine **B**, on crude reaction solutions prior to any isolation.

	**Characterisation prior to Isolation**									**Targeted Cage Size**	**Formed Cage Size (based on HRMS and/or diffusion NMR)?**
	**^{1}H NMR ($CDCl_3$)**		**HRMS**		**FTIR**				**Diffusion NMR**		
	**Clear Cage Formation?**	**Aldehyde SM?**	**Cage Present?**	**Mass ion?**	**C=O?**	**Stretch (cm^{-1})**	**C=N?**	**Stretch (cm^{-1})**	**Solvodynamic Diameter (nm)**		
**B1**	✓	✗	✓	$[M+H]^+$ 709.4009	✗	1702	✓	1643	1.14	[2+3]	[2+3]
**B2**	✓	✗	✓	$[M+H]^+$ 947.1284	✗	1696	✓	1642	1.22	[2+3]	[2+3]
**B3**	✗	✗	✗	-	✗	1703, 1687	✓	1638	n.d.	[2+3]	-
**B4**	✓	✗	✓	$[M+2H]^{2+}$ 499.2640	✗	1706	✓	1642	1.47	[2+3]	[2+3]
**B5**	✓	✗	✓	$[M+H]^+$ 937.5065	✗	1716, 1695	✓	1644	n.d.	[2+3]	[2+3]
**B6**	✓	✗	✓	$[M+H]^+$ 1015.5520	✗	1697	✓	1642	n.d.	[2+3]	[2+3]
**B7**	✓[a]	✗	✗	$[M+2H]^{2+}$ 536.7399[c]	✗	1700	✓	1641	n.d.	[2+3]	[2+3]
**B8**	✓[a]	✗	✓	$[M+H]^+$ 940.4987	✗	1695	✓	1641	n.d.	[2+3]	[2+3]
**B9**	✓	✗	✓	$[M+H]^+$ 955.3741	✗	1710, 1697	✓	1642	n.d.	[2+3]	[2+3]
**B10**	✓[a]	✓	✓	$[M+2H]^{2+}$ 656.9329	✓	1687	✓	1640, 1625	n.d.	[2+3]	[2+3]
**B11**	✓	✗	✓	$[M+2H]^{2+}$ 709.9136	✗	1690	✓	1637	1.67	[4+6]	[4+6]
**B12**	✗	✓	✗	-	✓	1679, 1688	✓	1629	n.d.	[4+6]	-
**B13**	✓	✗	✓	$[M+2H]^{2+}$ 889.9668	✓	1675	✓	1629	1.83	[4+6]	[4+6]
**B14**	✓[a]	✓	✓	$[M+3H]^{3+}$ 673.6685 and $[M+2H]^{2+}$ 505.2523	✓	1672	✓	1637	n.d.	[4+6]	[4+6] and [2+3] mix
**B15**	✓	✗	✓	$[M+2H]^{2+}$ 937.9986	✓	1698	✓	1639	1.97	[4+6]	[4+6]
**B16[e]**	✓[b]	✗	✗	-	✗	1696, 1651	✓	1627	n.d.	[4+6]	-
**B17**	✗	✓	✗	-	✓	1703, 1685	✓	1638	n.d.	[4+6]	-
**B18**	✓	✗	✓	$[M+2H]^{2+}$ 1009.4926	✗	1697, 1687	✓	1637	2.06	[4+6]	[4+6]
**B19[e]**	✓[a]	✗	✗	-	✗	1679	✓	1638	n.d.	[4+6]	-
**B20**	✓[a]	✓	✗	-	✗	1658	✓	1624	1.22	[4+6]	[2+3]
**B21**	✓[a]	✓	✓	$[M+H]^+$ 895.1855	✗	1669	✓	1625	n.d.	[4+6]	[2+3]
**B22**	✗	✗	✗	-	✗	1694	✓	1643	n.d.	[4+4]	-
**B23**	✓	✗	✓	$[M+2H]^{2+}$ 966.0047	✗	1697	✓	1638	1.85	[4+4]	[4+4]
**B24**	✓	✗	✗	-	✗	1699	✓	1640	2.01	[4+4]	[4+4]
**B25**	✓	✗	✗	-	✗	1700	✓	1640	2.20	[4+4]	[4+4]
**B26**	✓	✗	✗	-	✗	1698, 1685	✓	1639	2.45	[4+4]	[4+4]

[a] Possibly cage present in ^{1}H NMR spectra, but reaction either a complex mixture with oligomers present, or aldehyde SM, where specified, or sample very weak; [b] Complex mixture by ^{1}H NMR analysis but the positional disorder of the hydroxyl-substituent or chloro-substituent may account for this; [c] Mass ion indicating cage formed, but present alongside a large number of other unknown masses; [d] Clean conversion to cage-like species

observed by ^{1}H NMR spectroscopy, but two Ar-*H* species observed for the triamine component, and main mass ion instead consistent with clean formation of [2+2]-4H_2O species rather than the targeted [4+6] cage; [e] Possible cage formation apparent in the ^{1}H NMR spectra, but stoichiometry of cage could not be determined by HRMS or diffusion NMR; n.d. = not determined.

Supplementary Table 23: Summary of characterisation data from high-throughput cage screening, using triamine **C**, on crude reaction solutions prior to any isolation.

	**Characterisation prior to Isolation**									**Targeted Cage Size**	**Formed Cage Size (based on HRMS and/or diffusion NMR)?**
	**^{1}H NMR ($CDCl_3$)**		**HRMS**		**FTIR**				**Diffusion NMR**		
	**Clear Cage Formation?**	**Aldehyde SM?**	**Cage Present?**	**Mass ion?**	**C=O?**	**Stretch (cm^{-1})**	**C=N?**	**Stretch (cm^{-1})**	**Solvodynamic Diameter (nm)**		
**C1**	✓	✗	✓	$[M+H]^+$ 793.5057	✗	1689	✓	1643	1.28	[2+3]	[2+3]
**C2**	✓	✗	✓	$[M+H]^+$ 1031.2210	✓	1696	✓	1642	1.31	[2+3]	[2+3]
**C3**	✗	✗	✗	-	✗	1703, 1687	✓	1641	n.d.	[2+3]	-
**C4**	✓	✗	✓	$[M+2H]^{2+}$ 541.3111	✗	1706	✓	1643	1.50	[2+3]	[2+3]
**C5**	✓	✗	✓	$[M+H]^+$ 1021.5996	✗	1716, 1695	✓	1642	n.d.	[2+3]	[2+3]
**C6**	✓	✗	✓	$[M+H]^+$ 1099.6455	✗	1697	✓	1641	n.d.	[2+3]	[2+3]
**C7**	✓[a]	✗	✗	$[M+2H]^{2+}$ 578.7872	✗	1700	✓	1642	n.d.	[2+3]	[2+3]
**C8**	✓[a]	✗	✗	$[M+2H]^{2+}$ 512.8024	✗	1695	✓	1642	n.d.	[2+3]	[2+3]
**C9**	✓[a]	✓	✓	$[M+H]^+$ 1039.4674	✓	1710, 1697	✓	1641	n.d.	[2+3]	[2+3]
**C10**	✓[a]	✓	✓	$[M+2H]^{2+}$ 699.4800	✓	1687	✓	1641	n.d.	[2+3]	[2+3]
**C11**	✓	✗	✓	$[M+2H]^{2+}$ 794.0073	✓	1690	✓	1639	1.72	[4+6]	[4+6]
**C12**	✓[a]	✗	✓	$[M+H]^+$ 890.4577	✗	1679, 1688	✓	1625	n.d.	[4+6]	[2+3]
**C13**	✓	✓	✓	$[M+2H]^{2+}$ 974.0593	✓	1675	✓	1629	n.d.	[4+6]	[4+6]
**C14**	✓[a]	✓	✓	$[M+H]^+$ 1093.5862	✓	1672	✓	1639	n.d.	[4+6]	[2+3]
**C15**	✓[a]	✓	✓	$[M+2H]^{2+}$ 1022.0900	✓	1698	✓	1639	2.05	[4+6]	[4+6]
**C16[e]**	✓[b]	✗	✓	-	✗	1696, 1651	✓	1627	n.d.	[4+6]	-
**C17**	✗	✓	✗	-	✓	1703, 1685	✓	1637	n.d.	[4+6]	-
**C18**	✓[a]	✗	✓	$[M+3H]^{3+}$ 729.7295	✗	1697, 1687	✓	1637	n.d.	[4+6]	[4+6]
**C19[e]**	✓[a]	✗	✓	-	✗	1679	✓	1640	n.d.	[4+6]	-
**C20**	✓	✗	✗	-	✗	1658	✓	1623	1.47	[4+6]	[2+3]
**C21**	✓	✗	✓	$[M+H]^+$ 979.2783	✗	1669	✓	1627	1.37	[4+6]	[2+3]
**C22**	✗	✗	✗	-	✓	1694	✓	1642	n.d.	[4+4]	-
**C23**	✓	✗	✓	$[M+2H]^{2+}$ 1050.0983	✓	1697	✓	1639	2.00	[4+4]	[4+4]
**C24**	✓[a]	✗	✗	-	✓	1699	✓	1640	2.11	[4+4]	[4+4]
**C25**	✓	✗	✓	$[M+2H]^{2+}$ 1316.6399	✓	1700	✓	1639	2.43	[4+4]	[4+4]
**C26**	✓	✗	✗	-	✗	1698, 1685	✓	1639	2.56	[4+4]	[4+4]

[a] Possibly cage present in ^{1}H NMR spectra, but reaction either a complex mixture with oligomers present, or aldehyde SM, where specified, or sample very weak; [b] Complex mixture by ^{1}H NMR analysis but the positional disorder of the hydroxyl-substituent or chloro-substituent may account for this; [c] Mass ion indicating cage

formed, but present alongside a large number of other unknown masses; [d] Clean conversion to cage-like species observed by ^{1}H NMR spectroscopy, but two Ar-*H* species observed for the triamine component, and main mass ion instead consistent with clean formation of [2+2]-4H_2O species rather than the targeted [4+6] cage; [e] Possible cage formation apparent in the ^{1}H NMR spectra, but stoichiometry of cage could not be determined by HRMS or diffusion NMR; n.d. = not determined.

Supplementary Table 24: Summary of characterisation data for samples where cage had formed using triamine **A**, after isolation by removing the solvent under reduced pressure. If the solid sample did not fully re-dissolve, or showed signs of decomposition in the ^{1}H NMR spectra, this suggests the cage is not stable to isolation. For the isolated samples where cage was still present after analysis by ^{1}H NMR spectroscopy, the sample's crystallinity was also investigated by powder X-ray diffraction (PXRD) where there was enough sample.

	Characterisation After Isolation			
	^{1}H NMR ($CDCl_3$)			PXRD
	Fully re-dissolved?	Still cage?	Decomposition visible?	Crystallinity?
**A1**	✓	✓	✓	amorphous
**A4**	×	×	✓	amorphous
**A5**	×	×	✓	amorphous
**A6**	×	✓	×	amorphous
**A10**	×	✓	×	amorphous
**A11**	✓	✓	✓	n.d.
**A12**	✓	✓	✓	amorphous
**A22**	×	×	✓	amorphous

[a]On isolation, sample appears purer by ^{1}H NMR analysis

Supplementary Table 25: Summary of characterisation data for samples where cage had formed using triamine **B**, after isolation by removing the solvent under reduced pressure. If the solid sample did not fully re-dissolve, or showed signs of decomposition in the ^{1}H NMR spectra, this suggests the cage is not stable to isolation. For the isolated samples where cage was still present after analysis by ^{1}H NMR spectroscopy, the sample's crystallinity was also investigated by powder X-ray diffraction (PXRD) where there was enough sample.

	**Characterisation After Isolation**			
	**^{1}H NMR ($CDCl_3$)**			**PXRD**
	**Fully re-dissolved?**	**Still cage?**	**Decomposition visible?**	**Crystallinity?**
**B1**	✓	✓	×	amorphous
**B2**	×	✓[a]	×	n.d.
**B4**	✓	✓	×	amorphous
**B5**	×	✓	×	amorphous
**B6**	×	✓	×	amorphous
**B8**	×	✓	×	amorphous
**B9**	×	✓	×	amorphous
**B10**	✓	✓	×	amorphous
**B11**	×	✓	×	amorphous
**B13**	✓	✓	✓	amorphous
**B14**	✓	✓[a]	×	amorphous
**B15**	×	✓	✓	amorphous
**B18**	×	×	✓	amorphous
**B20**	✓	✓[a]	×	amorphous
**B21**	×	✓[a]	×	amorphous
**B23**	✓	✓	×	amorphous
**B24**	✓	✓	×	amorphous
**B25**	×	✓	✓	amorphous
**B26**	✓	✓	×	amorphous

[a]On isolation, sample appears purer by ^{1}H NMR analysis

Supplementary Table 26: Summary of characterisation data for samples where cage had formed using triamine **C**, after isolation by removing the solvent under reduced pressure. If the solid sample did not fully re-dissolve, or showed signs of decomposition in the ^{1}H NMR spectra, this suggests the cage is not stable to isolation. For the isolated samples where cage was still present after analysis by ^{1}H NMR spectroscopy, the sample's crystallinity was also investigated by powder X-ray diffraction (PXRD) where there was enough sample.

	Characterisation After Isolation			
	^{1}H NMR ($CDCl_3$)			PXRD
	Fully re-dissolved?	Still cage?	Decomposition visible?	Crystallinity?
**C1**	✓	✓	×	amorphous
**C2**	✓	✓	×	amorphous
**C4**	✓	✓	×	amorphous
**C5**	✓	✓	×	amorphous
**C6**	✓	✓	×	amorphous
**C8**	✓	✓	×	amorphous
**C9**	✓	✓	×	amorphous
**C10**	✓	✓	×	amorphous
**C11**	✓	✓	✓	amorphous
**C12**	×	✓	✓	amorphous
**C13**	×	✓	✓	amorphous
**C14**	✓	✓	×	amorphous
**C15**	✓	✓	×	amorphous
**C18**	×	✓	✓	amorphous
**C20**	✓	✓[a]	×	amorphous
**C21**	✓	✓[a]	×	amorphous
**C23**	✓	✓	✓	amorphous
**C24**	×	✓	×	amorphous
**C25**	✓	✓	×	amorphous
**C26**	✓	✓	×	amorphous

[a]On isolation, sample appears purer by ^{1}H NMR analysis

Supplementary Note 1: Nomenclature for cage topologies

We use the nomenclature for cage topologies that was introduced by Santolini *et al.* in 2017.[1] This nomenclature avoids confusion, frequent in the literature, resulting from naming topologies based on polyhedra such as "tetrahedron", since the latter is subjective and also depends on the shape that structure forms (which can be different in solution and the solid state). Several cages with the same underlying topology can have different shapes and therefore resemble different polyhedra.

Here, each structure is labelled as:

$$\mathbf{X_p^mY^n}$$

where *X* and *Y* are the two different component precursors that constitute the cage. *X* and *Y* are **Di** if they are di-topic (two reactive functionalities) and **Tri** if they are tri-topic (three reactive functionalities). By convention, the first precursor, *X*, has the highest number of reactive end groups (unless *X* = *Y*) and if the underlying topology relates to a polyhedron, the X will lie at the vertices. The second precursor, *Y*, can have a number of reactive end groups less to or equal to *X*. When *X* = *Y* is equal, then the choice of which functional unit is denoted as *X* and *Y* is arbitrary. The superscripts *m* and *n* denote the number of each precursor incorporated into the topology for *X* and *Y*, respectively. Most of the time, *X*-type precursors are connected to other *X*-type precursors through only one *Y*-type precursor; in this case, no subscript p is given. However, if two *X*-type precursors are directly connected through links with two distinct *Y*-type precursors, then *p* = 2. Hence, the subscript *p* gives the number of double connections between precursor pairs within a topology. These multiple links result in topologies with multiple ring sizes. For some of the smaller topologies with only two *X*-type building blocks (BBs), there can be triple or quadruple linking of the precursors; in this case, no subscript is given as there is no alternative connectivity for that topology. An example of a topology with these multiple links is the $\mathbf{Tri_2^4Di^6}$ topology, in which each of the tri-topic BBs is doubly connected to one of the neighbouring tri-topic BBs through two di-topic BBs. This contrasts to the more common $\mathbf{Tri^4Di^6}$ topology, which is related to a tetrahedron and does not have the multiple links (shown in Fig. 2a).

Supplementary Note 2: *A priori* prediction of cage topologies

Models of the most likely topologies were assembled to determine the intrinsic structural preference for a topology in the representative set of three systems (**B1**, **B11**, **B23**): for **B1** and **B11** - $\mathbf{Tri^2Di^3}$, $\mathbf{Tri^4Di^6}$, $\mathbf{Tri^6Di^9}$, and $\mathbf{Tri^8Di^{12}}$, and for **B23** - $\mathbf{Tri^1Tri^1}$, $\mathbf{Tri_2^2Tri^2}$ and $\mathbf{Tri^4Tri^4}$. The approach as described in the **Supplementary Methods** was applied. Their likelihood of

formation was then determined by comparing relative energies per [2+3] or [1+1] unit. This energetic comparison was possible for these systems as these topology series contain multiples of the same building unit. The equivalent comparison is not possible between molecules from different series (*e.g.* between a $\mathbf{Tri^{4}Di^{6}}$ and $\mathbf{Tri^{4}Tri^{4}}$ molecule, hence the formation energies were used in those instances, as described in the **Supplementary Methods**).

As an example, to compare the energies for **B1**, we compared $E_{\mathbf{Tri2Di3}}$ to ($E_{\mathbf{Tri4Di6}}$/2) to ($E_{\mathbf{Tri6Di9}}$/3) to ($E_{\mathbf{Tri8Di12}}$/4). This allows us to compare the relative internal energy of a single [2+3] unit in each of the molecules. We use the word "internal" in reference to the fact that we do not include a description of any other factors such as the solvent effect.

Excluded Topologies: An alternative topology to a $\mathbf{Tri^{4}Di^{6}}$ outcome for a [4+6] reaction is $\mathbf{Tri^{4}_{2}Di^{6}}$. We did not include this possibility here in the simulations because this topology contains two-doubly connected tri-topic building blocks (indicated in the nomenclature by the subscript *2*) as well as two singularly-connected building blocks. The double connections result in two small windows in the $\mathbf{Tri^{4}_{2}Di^{6}}$ topology, and any such molecule with highly symmetric tri-topic precursors is likely to be highly strained, as found previously in similar systems.[1] The same applies for the $\mathbf{Tri^{3}_{2}Tri^{3}}$ topology, which we also excluded from the simulations.

Supplementary Note 3: Model cage formation, reaction optimisation and extension to other topologies

Before translating the synthesis of organic cages onto an automated robotic platform, we first set out to determine robust reaction conditions for each of the three aldehyde types used in this study. Our aim was to develop reaction conditions that would afford reasonable conversion to the cage molecule while keeping it in solution without any crystallisation or precipitation. There were two reasons for this: first, some cages are unstable to isolation in the solid state, especially if they are not shape-persistent, and hence good solubility would allow high-throughput solution analysis to determine whether a cage had formed prior to isolation. Second, working under homogenous conditions avoids any potential problems associated with the handling of suspensions on the robotic platform.

Previously, the reaction of (2,4,6-trimethylbenzene-1,3,5-triyl)trimethanamine (**B**) with terephthalaldehyde (**11**) was reported to produce a complex precipitate.[2] However, by carrying out this reaction at a lower concentration than we have used previously for other imine cages,[3,4] and heating, clean albeit incomplete conversion to a cage species was observed by ^{1}H NMR spectroscopy (see **Supplementary Table 15**, entries 1 and 2, and **Supplementary Fig. 43**). Full conversion of the terephthalaldehyde (**11**) to a cage species was achieved by using a one-molar equivalent excess of triamine (**B**), although this was limited to

an additional equivalent per predicted cage stoichiometry (entry 3), *i.e.,* for a **Tri^{4}Di^6** topology, which contains 4 molecules of triamine, 5 equivalents were used in the reaction. Using a larger excess of triamine led to the formation of impurities (entry 4). Also, increasing the concentration of the reaction led to increasing amounts of a precipitate and this was avoided to facilitate transfer of the reactions to the robotic platforms (entries 5–7).

Scale-up of the optimised reaction conditions (**Supplementary Table 15**, entry 3), and analysis by high-resolution mass spectroscopy (HRMS), indicated that **B11** was a [4+6] cage. This validated computational predictions and the cage could be easily isolated in high purity by solvent-exchange (see **Supplementary Methods**, and **Supplementary Figs. 43-45**).

With the successful formation of the new **Tri^{4}Di^6** cage (**B11$_{[4+6]}$**), we needed to determine whether the reaction conditions were translatable to other systems before transferring them to an automated platform. Typically, the formation of different organic cages has required small alterations to tune the reaction conditions to successfully synthesise them in reasonable yields — for example, variations in the concentration, temperature, solvent, addition of a catalyst, removal of water during the reaction, rate and order of reagent addition, and layering have all been utilised previously, even for structurally analogous cages.[5] This is difficult to implement when transitioning the synthesis of cages onto an automated robotic platform where, ideally, a single set of robust conditions is required that can be applicable to a range of cage molecules. We therefore investigated the expansion to other aldehyde building blocks (isophthalaldehyde (**1**) and tris(4-formylphenyl)amine (**23**)) that might enable access to other cage topologies (**B1$_{[2+3]}$** and **B23$_{[4+4]}$**); the success of this confirmed that our optimised conditions could be applied to other systems (see **Supplementary Methods**, and **Supplementary Figs. 46-49**).

Supplementary Note 4: Modelling of cages in the high-throughput screen

All molecules were assembled into their targeted topologies, **Tri^{2}Di^3** for aldehydes **1**–**10**, **Tri^{4}Di^6** for aldehydes **11**–**21** and **Tri^{4}Tri^4** for aldehydes **22**–**26**. We did not include precursor **7** in the computational study because OPLS3 did not reproduce the nitro-group on the aromatic ring well. After initial assembly showed that the alkyl group on precursor **10** was external to the cage, we did not include the alkyl group in subsequent modelling as it was not deemed to be important for cage formation and yet would require significant additional computational cost.

Supplementary Note 5: Structural analysis of the computational models

For the samples that were investigated using diffusion NMR, the inner pore sizes, average diameters, and maximum outer diameters were calculated from the lowest energy modelled

structures to allow for comparison to the experimentally measured solvodynamic diameters, with Δ(CALC-EXP) showing the difference between the calculated average diameter (CALC) and the measured solvodynamic diameter (EXP):

i) Pore size: Calculated as the distance between the pore centre, assumed to be at the centre of mass of the molecule, and the closest atom. The obtained distance is then corrected for the appropriate van der Waals radius, and multiplied by 2.

ii) OPT pore size: The pore size in (i) assumes the centre of mass of the cage is also the central point of the cavity, which is true in the case of highly symmetric cage molecules, but not the case in all systems, for example in elongated, non-spherical cavities. To reflect the latter, we also calculated the pore size by finding the true centre of the cavity and then calculating the largest sphere that could fit in the cavity. The latter we name as the OPT pore size. Practically, to find the centre of the cavity, we used an optimisation step in our in-house software that finds the coordinates that maximise the pore size.

iii) The maximum outer diameter is defined as the distance between the two furthest atoms, corrected by the appropriate van der Waals radii.

iv) The steps required to calculate the average diameter are as follows:

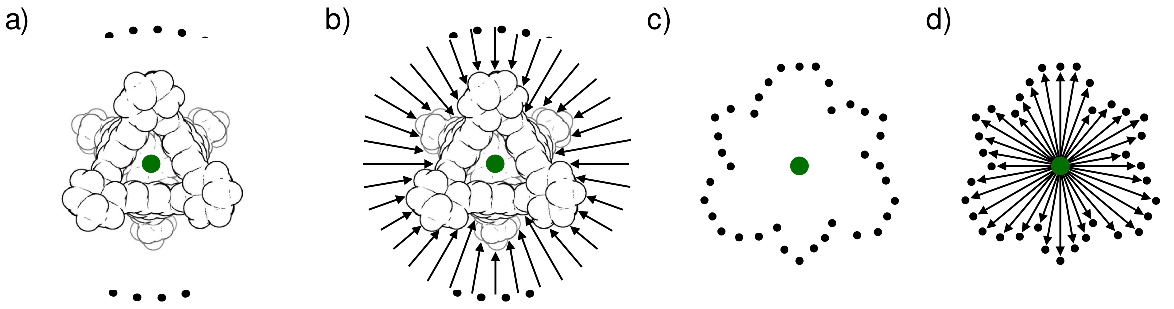

a) A molecule is taken and the maximum outer diameter is determined. Then, a set of sampling points is distributed evenly on a sphere with radius equal to the maximum outer diameter, using Vogel's golden vector approach for an even points distribution on a disc, modified for a sphere.[6]

b) Each sampling point is connected to the centre of mass of the molecule by a vector. The overlap of the vector paths and the van der Waals spheres of the molecule's atoms is then analysed.

c) The molecule's outline, as a set of points determined by the vectors crossing through the van der Waals spheres, is created.

d) The average of the distances of the molecular outline points yields the molecule's average diameter ($\bar{d}$) and is given by the equation:

$$\bar{d} = \frac{2}{n}\sum_{i=1}^{n} x_i$$

where x_i, is equal to the distance of the ith outline point from the centre of mass.

Supplementary Note 6: Modelling of covalently bridged cage catenane structures

To compare the relative energy of the covalently bridged cage catenane structure to the related **Tri⁴Di⁶** topology, we took the structure of a single [8+12] molecule from the SCXRD structure and geometry optimised at the DFT level, as described above in the **Supplementary Methods**.

Supplementary Note 7: Representative worked example of high-throughput characterisation

We include here a representative worked example of determining cage formation for **B15**, one of the 'hits' from the high-throughput screen:

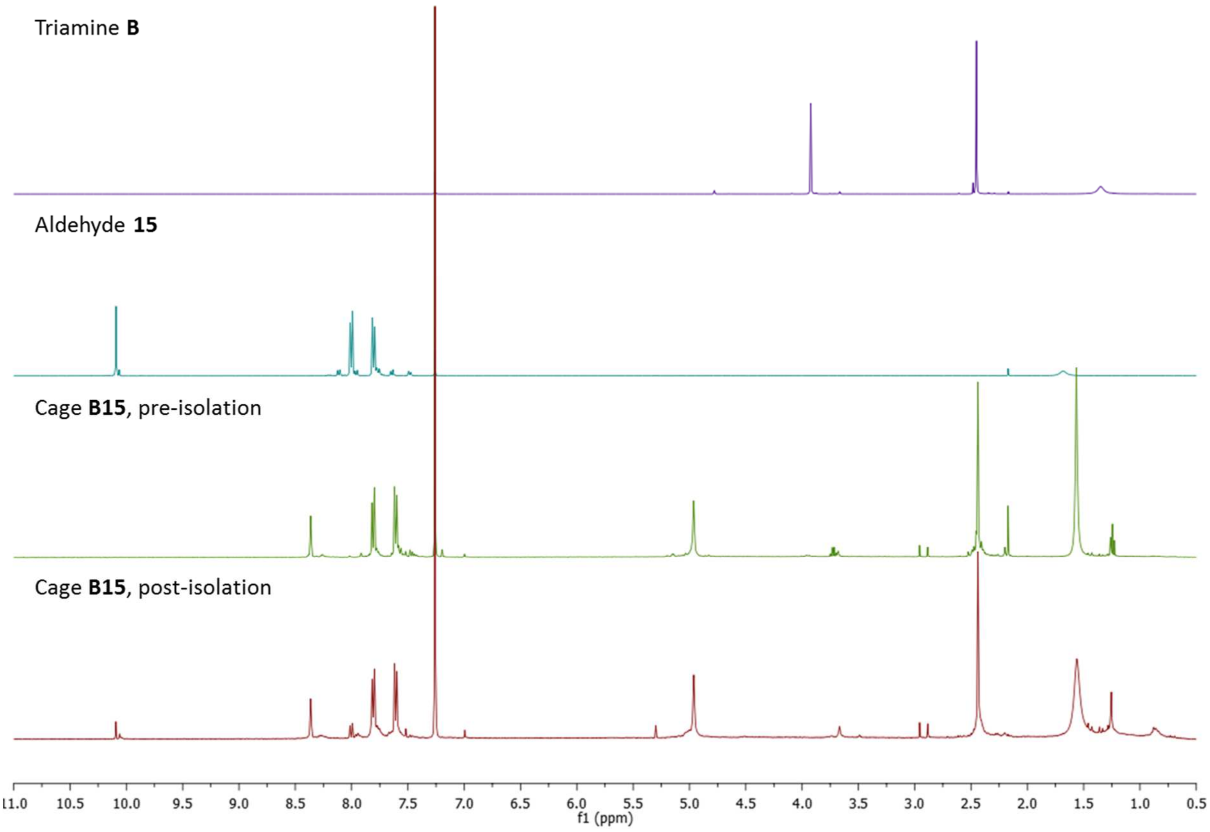

Supplementary Figure 115: Stacked ^{1}H NMR spectra ($CDCl_3$) of the precursors used, and the crude reaction, for **B15**, taken directly from the high-throughput screen (pre-isolation) indicating conversion of aldehyde to an imine, accompanied by a 'clean' aromatic region that indicates cage formation. Comparison of the ^{1}H NMR spectra (lower) after removal of solvent (post-isolation) shows cage still present, with a small amount of decomposition visible.

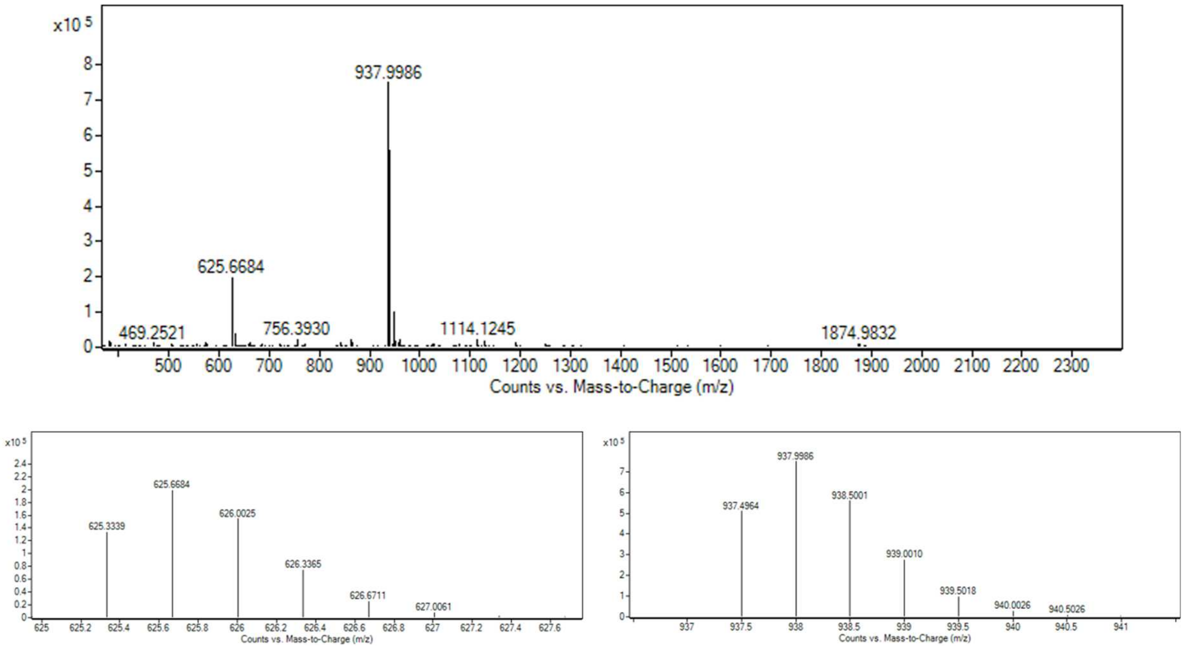

Supplementary Figure 116: HRMS spectra for crude reaction **B15**, taken directly from the high-throughput screen (pre-isolation), indicating the clean formation of a [4+6] cage – $[M+2H]^{2+}$ 937.9986 and $[M+3H]^{3+}$ 625.6684.

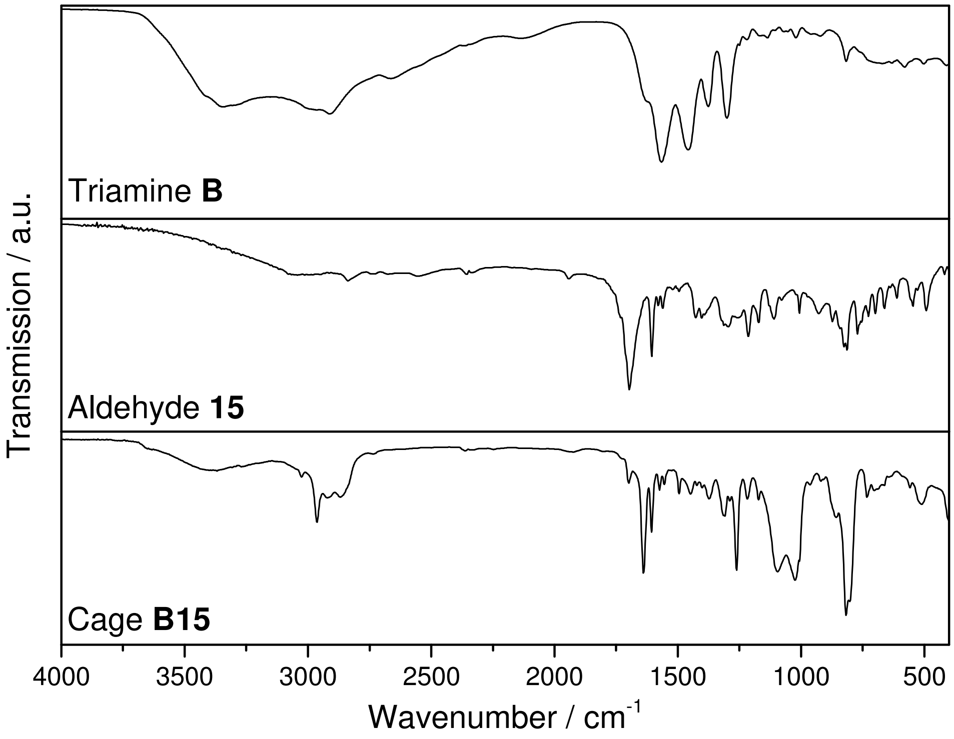

Supplementary Figure 117: Stacked FTIR spectra of the precursors used, and the crude reaction, for **B15**, taken directly from the high-throughput screen (pre-isolation) showing loss of the C=O stretch, and the formation of an imine C=N stretch.

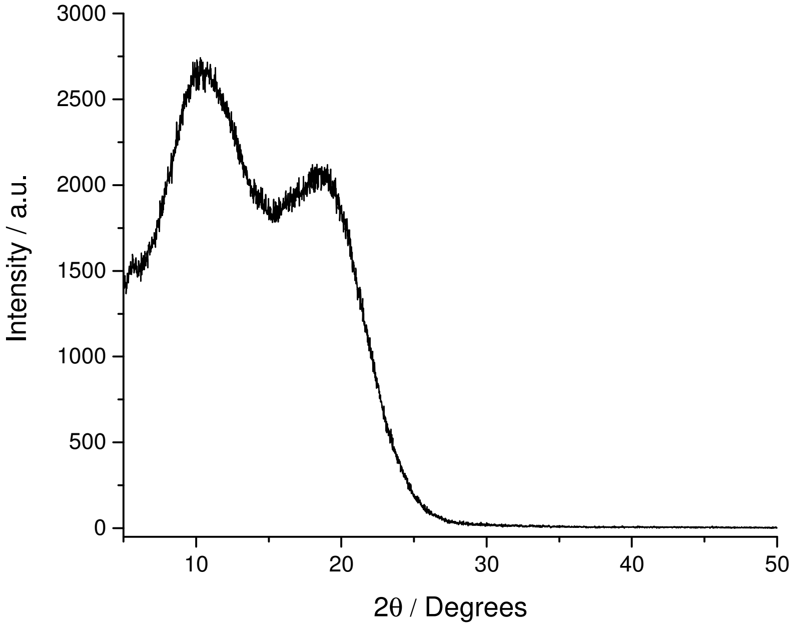

Supplementary Figure 118: PXRD of **B15** (post-isolation) indicating that the crude cage was amorphous in nature.

Supplementary Table 27: Summary of three separately measured diffusion co-efficients for **B15**, demonstrating reproducibility before remaining high-throughput samples only had a single measurement carried out.

Diffusion Co-efficients (D, 10^{-10} $m^2.s^{-1}$)					
**B15**	**Peak Range (ppm)**			**Average per run**	**SD per run**
**Run**	**7.867–7.530**	**5.050–4.902**	**2.450–2.424**		
1	4.137	4.139	4.085	4.120	0.025
2	4.129	4.070	4.083	4.094	0.025
3	4.118	4.082	4.087	4.096	0.016
**Average Diffusion Co-efficient**				**4.103 ± 0.022 x 10^{-10}**	

Supplementary Table 28: Calculated solvodynamic radii (R_S) for **B15** over three separate measurements, using the Stokes-Einstein equation with the measured diffusion co-efficients and the viscosity of chloroform (0.542 cP at 25 °C).

Solvodynamic Radius (R_S, nm)					
**B15**	**Peak Range (ppm)**			**Average per run**	**SD per run**
**Run**	**7.867–7.530**	**5.050–4.902**	**2.450–2.424**		
1	0.9734	0.9730	0.9858	0.9774	0.0060
2	0.9753	0.9895	0.9863	0.9837	0.0061
3	0.9779	0.9866	0.9854	0.9833	0.0038
**Average Solvodynamic Radius**				**0.981 ± 0.005**	

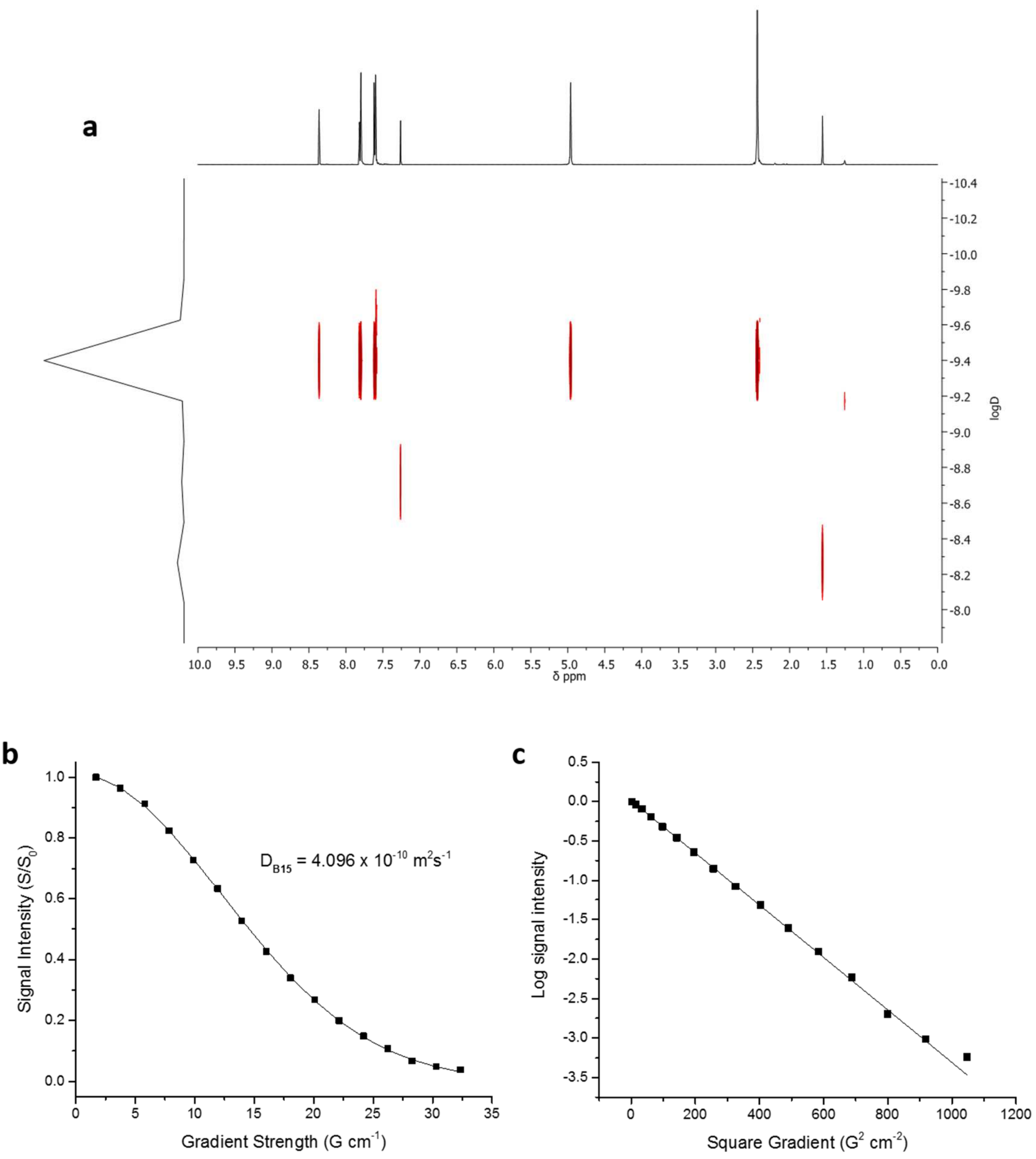

Supplementary Figure 119: Example ^{1}H DOSY spectra (with the least attenuated spectrum [top]) for cage **B15**; (b) Attenuation of aromatic ^{1}H NMR signal with increasing gradient strength for the example shown; (c) Straight-line Stejskal-Tanner PFG-NMR response curve for the example shown. All samples were calibrated individually but measured to the same standard as shown here.

Supplementary Methods

General methods for computational structure generation

When a cage model was required, the structures were assembled using in-house computational software that works as follows. Firstly, the pair of precursor molecules is generated based on their SMILES code and RDKit software[7] to generate precursor geometries, which are then optimised with the Universal Forcefield (UFF).[8] The precursors are then placed on the vertices and edges of the relevant polyhedra based on the underlying topology of the cage, the reactive end groups of the precursors are then linked and the chemistry corrected such that the aldehyde and amine pairs form imine bonds. The structures of the assembled molecules are then initially geometry optimised using UFF to produce feasible structures, and are then geometry optimised with the OPLS3 forcefield[9] using MacroModel (version 10.3.015, Schrödinger, LLC, New York, NY, 2015-3), which we have previously found to produce reliable structures for porous organic cages.[1] At the next stage, global optimisation searches are carried out in order to find the lowest energy conformations for the cage and to ensure there is no dependence upon the initial starting geometry.

Global optimization calculations

After trialling both low-mode conformer search calculations and sampling molecular dynamics (MD) simulations as an approach to finding low energy conformations, we found that the latter was more efficient for this work. For each cage molecule, we would carry out multiple high temperature MD simulations to locate the lowest energy conformers, and simulations were repeated until no new low energy conformers were found in at least 3 MD runs. Each MD run was performed with OPLS3 in MacroModel at 1000 K for 100 ns with a time step of 0.7 fs and sampling every 10 ps. Each sampled structure was geometry optimised with the Polak-Ribier Conjugate Gradient method and a convergence criteria of 0.05 kJ mol^{-1} Å^{-1}. Each individual MD simulation generated 10000 conformers. From the multiple MD simulations, the lowest energy conformation, plus a few selected conformations to represent the key conformations observed within a 20 kJ mol^{-1} energy gap were chosen for subsequent DFT refinement. A single conformation was chosen if the lowest energy conformation was symmetric; more were chosen if it the molecule was more flexible or asymmetric. Given the large amount of conformations generated, it was not possible to visualise or refine at Density Functional Theory (DFT) level every single conformer. In the future, software that analyses symmetry and cavity size could be applied to extract a selection of the best candidates within an energy range.

Refinement of structures with DFT calculations

For more reliable energetic rankings of the molecules, DFT calculations were conducted on the selected low energy conformations. DFT calculations were run in CP2K[10] with the PBE potential,[11] combined with the TZVP basis set,[12] Grimme D3 dispersion corrections,[13] and a plane-wave cut-off of 350 Ry. We have found previously that this approach gives a reasonable balance between computational cost and accuracy.[1] The convergence criteria was an energy difference of less than 1 x 10^{-3} Hartree.

Calculation of formation energies

The formation energies of the cages were calculated at the DFT level using the following equation:

$$\mathbf{E}_{formation} = \frac{(\mathbf{E}_{cage} + \mathbf{x}\,\mathbf{E}_{water}) - (\mathbf{m}\,\mathbf{E}_{aldehyde} + \mathbf{n}\,\mathbf{E}_{amine})}{\mathbf{xn}}$$

where E_{cage} is the energy of the cage formed, E_{water} is the energy of the water produced in the reaction, $E_{aldehyde}$ is the energy of the aldehyde and E_{amine} is the energy of the amine. The latter values were calculated at the same level as described in 3, on the lowest energy conformers of the molecules. The number of aldehyde reactants is *m*, *n* is the number of amine precursors and *xn* is the number of imine bonds formed, equivalent to the number of water molecules, *x*, produced in the reaction.

General synthetic and analytical methods

Materials: Chemicals were purchased from TCI UK, Fluorochem, or Sigma-Aldrich and used as received. Solvents were reagent or HPLC grade purchased from Fisher Scientific, with the exception of chloroform-D which was purchased from Apollo Scientific and used for the high-throughput screen. All chemicals and solvents were used as received, unless specified.

Synthesis: All reactions requiring anhydrous or inert conditions were performed in oven-dried apparatus under an inert atmosphere of dry nitrogen, using anhydrous solvents introduced into the flask using disposable needles and syringes. All reactions were stirred magnetically using Teflon-coated stirring bars. Where heating was required, the reactions were warmed using a stirrer hotplate with heating blocks with the stated temperature being measured externally to the reaction flask with an attached probe. Removal of solvents was done using a rotary evaporator.

High-throughput cage discovery: High-throughput automated synthesis was conducted using a Chemspeed Accelerator SLT-100 automated synthesis platform, and removal of solvents was carried out using a Combidancer evaporator.

TLC and column chromatography: Reactions were monitored by thin layer chromatography (TLC), conducted on pre-coated aluminium-backed plates (Merck Kieselgel 60 with fluorescent indicator UV_{254}). Spots were visualized either by quenching of UV fluorescence or by staining with potassium permanganate. Flash column chromatography was performed on a Biotage Isolera with KP-Sil Normal Phase disposable columns.

Melting points: Obtained using Griffin melting point apparatus and are uncorrected.

IR spectra: Infra-red (IR) spectra were recorded on a Bruker Tensor 27 FT-IR using ATR measurements for oils and solids as neat samples, or for the high-throughput screening, in transmission mode on a 96-well silica wafer deposited as a thin film.

NMR spectra: ^{1}H Nuclear magnetic resonance (NMR) spectra were recorded using an internal deuterium lock for the residual protons in $CDCl_3$ (δ = 7.26 ppm) at ambient probe temperature using either a Bruker Avance 400 (400 MHz) or Bruker DRX500 (500 MHz) instrument.

NMR data are presented as follows: chemical shift, integration, peak multiplicity (s = singlet, d = doublet, t = triplet, q = quartet, m = multiplet, br = broad, app = apparent) and coupling constants (J / Hz). Chemical shifts are expressed in ppm on a δ scale relative to δ_{CDCl_3} (7.26 ppm) and coupling constants, *J*, are given in Hz.

^{13}C NMR spectra were recorded using an internal deuterium lock using $CDCl_3$ (δ = 77.16 ppm) at ambient probe temperatures using either a Bruker Avance 400 (101 MHz) or Bruker DRX500 (126 MHz) instrument.

HRMS: Electrospray ionization mass spectrometry (ES-MS) was carried out using an Agilent Technologies 6530B accurate-mass QTOF Dual ESI mass spectrometer (MeOH + 0.1% formic acid, capillary voltage 4000 V, fragmentor 225 V) in positive-ion detection mode for cage samples, or using Agilent Technologies 6530B accurate-mass QTOF multimode mass spectrometer (MeOH + 0.1% formic acid, capillary voltage 1500 V, fragmentor 125 V) in positive ion mode for precursors.

HPLC: HPLC was conducted on a Dionex UltiMate 3000 equipped with a diode array UV detector using a Thermo-Scientific Syncronis C8 column, 150 x 4.6 mm, 3 µm (SN 10136940, Lot 12459). The mobile phase was isocratic MeOH at a flow rate of 1 mL/min for a 20 minute run time. The injection volume was 10 µL and the sample concentration was approximately 1 mg mL^{-1}. Detection for UV analysis was conducted at 254 nm.

PXRD: Laboratory powder X-ray diffraction data were collected in transmission mode on samples held on a black opaque 96-shallow well microplate (ProxiPlate-96 Black) on a Panalytical X'Pert PRO MPD equipped with a high-throughput screening (HTS) XYZ stage, X-ray focusing mirror and PIXcel detector, using Ni-filtered Cu Kα radiation. Data were measured over the range 4–50° in ~0.013° steps over 15 minutes.

Gas sorption analysis: Surface areas were measured by nitrogen sorption at 77.3 K. Powder samples were degassed offline at 90 °C for 15 hours under dynamic vacuum (10^{-5} bar) before analysis, followed by degassing on the analysis port under vacuum, also at 90 °C. Isotherms were measured using Micromeritics 2020, or 2420 volumetric adsorption analyzer. Gas uptake measurements (for N_2, H_2) were taken at a temperature of 77 K, stabilized using a circulating water chiller/heater.

Diffusion NMR: All measurements were carried out non-spinning on a 400 MHz Bruker Avance 400 spectrometer, using a 5 mm indirect detection probe, equipped with a z-gradient coil producing a nominal maximum gradient of 34 G/cm. Diffusion data was collected using the Bruker longitudinal eddy current delay (LED) pulse sequence (ledgp2s). A diffusion encoding pulse δ of length 1–7 ms and diffusion delay D of 0.1–0.25 s was used. Gradient amplitudes were equally spaced between 1.70 and 32.4 G/cm. Each FID was acquired using 16 k data points. All experiments were carried out at a nominal probe temperature of 298 K, with an air flow of 800 L/h to minimise convection. All diffusion coefficients were calculated using measurements from multiple peak areas in the ^{1}H NMR spectra and the numbers quoted represent the mean values, but measurement was only carried out once to generate a high-throughput methodology.

Diffusion coefficients were calculated from signal intensities using the Skejskal-Tanner equation:[14]

$$I = I_0 e^{\gamma^2 g^2 \delta^2 \left(\Delta - \delta/3\right) D}$$

Where I is the signal intensity, I_0 is the signal intensity at a gradient strength of zero, g is the gradient strength, and D is the diffusion coefficient (D = m^2/s). Solvodynamic radii, R_S (nm), of solution-phase species were calculated from the Stokes-Einstein equation assuming molecules have a spherical geometry:

$$D = \frac{kT}{6\pi\eta R_S}$$

Single crystal X-ray diffraction: Single crystal X-ray data sets were measured using a Rigaku MicroMax-007 HF rotating anode diffractometer (Mo-Kα radiation, λ = 0.71073 Å, Kappa 4-circle goniometer, Rigaku Saturn724+ detector); at beamline 11.3.1, Advanced Light Source, Berkeley, USA, using silicon monochromated synchrotron radiation (λ = 0.7749 Å or 1.0332 Å, PHOTON100 CMOS detector); at beamline I19, Diamond Light Source, Didcot, UK using silicon double crystal monochromated synchrotron radiation (λ = 0.6889 Å, Dectris Pilatus 2M, or Rigaku Saturn724+ detector); or using a Bruker D8 Venture Advance diffractometer equipped with IµS microfocus source (Cu-Kα radiation, λ = 1.54178 Å, Kappa 4-circle goniometer, PHOTON100 CMOS detector). Unless started in the refinement details section (**Supplementary Methods**), solvated single crystals, isolated from the crystallization solvent, were immersed in a protective oil, mounted on a MiTeGen loop, and flash cooled under a dry nitrogen gas flow. Rigaku frames were converted to Bruker compatible frames using the programme ECLIPSE.[15] Empirical absorption corrections, using the multi-scan method, were performed with the program SADABS.[16,17] Structures were solved with SHELXD,[18] SHELXT,[19] or by direct methods using SHELXS,[20] and refined by full-matrix least squares on $|F|^2$ by SHELXL,[21] interfaced through the programme OLEX2.[22] Unless stated, all non-H-atoms were refined anisotropically, and H-atoms were fixed in geometrically estimated positions and refined using the riding model. Supplementary CIF's, that include structure factors and responses to checkCIF alerts, are available free of charge from the Cambridge Crystallographic Data Centre (CCDC) via www.ccdc.cam.ac.uk/data_request/cif.

Synthesis of triamine precursors

2,2',2''-(Benzene-1,3,5-triyltris(methylene))tris(isoindoline-1,3-dione), S1

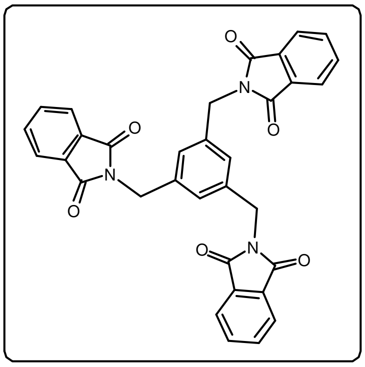

A modification of the procedure of Grawe *et al*. was used for this reaction.[23] To a solution of 1,3,5-tris(bromomethyl)-benzene (5.00 g, 14.01 mmol, 1.0 eq.) and 18-crown-6 (1.11 g, 4.20 mmol, 0.3 eq.) in toluene (180 mL) was added potassium phthalamide (9.34 g, 50.44 mmol, 3.6 eq.). The mixture was heated at 100 °C under N_2 for 23 h before being allowed to cool to room temperature. The mixture was concentrated *in vacuo* and the resulting solid suspended in water (200 mL) and collected by filtration. The resulting solid was further washed with water (2 × 200 mL) and MeOH (200 mL) before being dried *in vacuo* to afford the desired product **S2** as a colourless solid which was used without further purification (7.05 g, 12.69 mmol, 90%).

^{1}H NMR (500 MHz, $CDCl_3$): δ_H 7.82 (dd, *J* = 5.5, 3.0 Hz, 6H), 7.69 (dd, *J* = 5.4, 3.1 Hz, 6H), 7.35 (s, 3H), 4.78 (s, 6H); **^{13}C NMR** (126 MHz, $CDCl_3$): δ_C 168.0, 137.5, 134.1, 132.2, 128.0, 123.5, 41.4. Data in accordance with literature values.[24]

Benzene-1,3,5-triyltrimethanamine, A

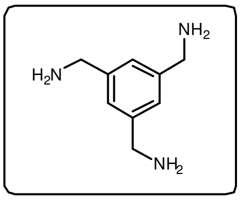

A modification of the procedure of Grawe *et al*. was used for this reaction.[23] To a suspension of 2,2',2''-(benzene-1,3,5-triyltris-(methylene))tris(isoindoline-1,3-dione) **S2** (7.05 g, 12.69 mmol, 1.0 eq.) in a mixture of toluene (100 mL) and EtOH (200 mL) was added hydrazine hydrate in a single portion (4.74 mL, 50 wt% solution in water, 76.14 mmol, 6.0 eq.). The resulting mixture was heated at 90 °C for 44 h, at which point a large amount of solid had precipitated, before being allowed to cool to room temperature. The reaction mixture was concentrated *in vacuo* (not to dryness) and partitioned between an aqueous KOH solution (40 mL, 40 wt%) and $CHCl_3$ (200 mL). The aqueous layer was further extracted with $CHCl_3$ (2 × 150 mL) before the combined organic layers were dried (Na_2SO_4) and concentrated *in vacuo* to afford the desired triamine **A** as a yellow oil which was used without further purification (785 mg, 4.75 mmol, 37%).

^{1}H NMR (500 MHz, $CDCl_3$): δ_H 7.14 (s, 3Hs, 3.85 (s, 6H), 1.49 (br s, 6H); **^{13}C NMR** (126 MHz, $CDCl_3$): δ_C 144.0, 124.4, 46.5. Data in accordance with literature values.[25]

2,2',2''-((2,4,6-Trimethylbenzene-1,3,5-triyl)tris(methylene))tris(isoindoline-1,3-dione), S2

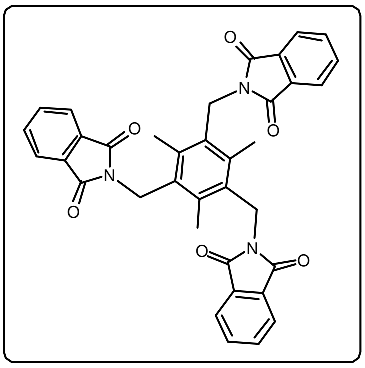

A modification of the procedure of Grawe *et al*. was used for this reaction.[23] To a solution of 1,3,5-tris(bromomethyl)-2,4,6-trimethylbenzene (20.00 g, 50.13 mmol, 1.0 eq.) and 18-crown-6 (3.97 g, 15.03 mmol, 0.3 eq.) in toluene (640 mL) was added potassium phthalamide (33.42 g, 180.47 mmol, 3.6 eq.). The mixture was heated at 100 °C under N_2 for 24 h before being allowed to cool to room temperature. The mixture was concentrated *in vacuo* and the resulting solid suspended in water (400 mL) and collected by filtration. The resulting solid was further washed with water (2 × 400 mL) and MeOH (400 mL) before being dried *in vacuo* to afford the desired product **S3** as a colourless solid which was used without further purification (30.45 g, quant).

^{1}H NMR (500 MHz, $CDCl_3$): δ_H 7.78 (dd, *J* = 5.5, 3.0 Hz, 6H), 7.67 (dd, *J* = 5.4, 3.1 Hz, 6H), 4.95 (s, 6H), 2.50 (s, 9H); **^{13}C NMR** (101 MHz, $CDCl_3$): δ_C 168.3, 138.7, 134.0, 132.1, 130.3, 123.4, 38.7, 17.5. Data in accordance with literature values.[26]

(2,4,6-Trimethylbenzene-1,3,5-triyl)trimethanamine, B

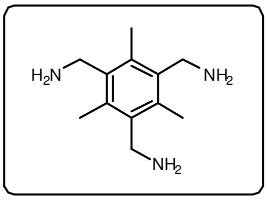

A modification of the procedure of Grawe *et al*. was used for this reaction.[23] To a suspension of 2,2',2''-((2,4,6-trimethylbenzene-1,3,5-triyl)tris(methylene))tris(isoindoline-1,3-dione) **S3** (37.43 g, 62.63 mmol, 1.0 eq.) in a mixture of toluene (550 mL) and EtOH (1100 mL) was added hydrazine hydrate in a single portion (23.4 mL, 50 wt% solution in water, 375.79 mmol, 6.0 eq.). The resulting mixture was heated at 90 °C for 5 days, at which point a large amount of solid had precipitated, before being allowed to cool to room temperature. The reaction mixture was concentrated *in vacuo* (not to dryness) and partitioned between an aqueous KOH solution (200 mL, 40 wt%) and $CHCl_3$ (500 mL). The aqueous layer was further extracted with $CHCl_3$ (2 × 300 mL) before the combined organic layers were dried (Na_2SO_4) and concentrated *in vacuo* to afford the desired triamine **B** as a pale yellow solid which was used without further purification (12.20 g, 58.87 mmol, 94%).

mp 126-128 °C; **IR** (ν_{max}/cm^{-1}) 3355 (br), 2907 (br), 2662 (br), 1565, 1455, 1376, 1299; **^{1}H NMR** (500 MHz, $CDCl_3$): δ_H 3.93 (s, 6H), 2.45 (s, 9H), 1.32 (br s, 6H); **^{13}C NMR** (101 MHz, $CDCl_3$): δ_C 138.3, 133.6, 41.0, 15.6.

1,3,5-Tris(bromomethyl)-2,4,6-triethylbenzene, S3

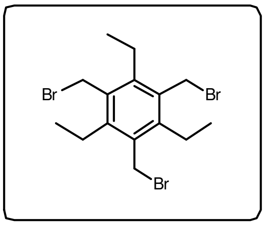

The procedure of Vacca *et al*. was used for this reaction.[27] To a mixture of paraformaldehyde (16.7 g, 556.11 mmol, 10.5 eq.) and triethylbenzene (10 mL, 53.12 mmol, 1.0 eq.) in a solution of HBr in AcOH (100 mL, 30 wt%) was added zinc bromide (19.7 g, 87.47 mmol, 1.65 eq.) portionwise at room temperature. After complete addition, the reaction was heated to 90 °C under N_2 for 22 h before being allowed to cool to room temperature. The precipitated solid was collected by filtration, washed with water (3 × 400 mL) and dried *in vacuo* to afford the desired product **S1** as an off-white solid (19.23 g, 43.60 mmol, 82%).

^{1}H NMR (500 MHz, $CDCl_3$): δ_H 4.58 (s, 6H), 2.94 (q, *J* = 7.6 Hz, 6H), 1.34 (t, *J* = 7.6 Hz, 9H); **^{13}C NMR** (126 MHz, $CDCl_3$): δ_C 145.2, 132.8, 28.7, 22.9, 15.8. Data in accordance with literature values.[27]

2,2',2''-((2,4,6-Triethylbenzene-1,3,5-triyl)tris(methylene))tris(isoindoline-1,3-dione), S4

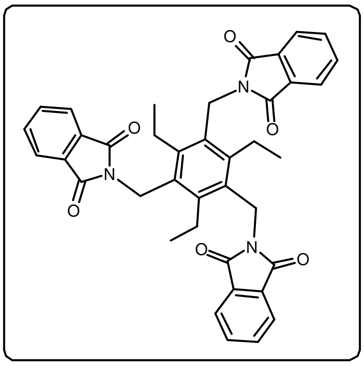

A modification of the procedure of Grawe *et al*. was used for this reaction.[23] To a solution of 1,3,5-tris(bromomethyl)-2,4,6-triethylbenzene **S1** (19.2 g, 43.53 mmol, 1.0 eq.) and 18-crown-6 (3.45 g, 13.06 mmol, 0.3 eq.) in toluene (555 mL) was added potassium phthalamide (29.02 g, 156.72 mmol, 3.6 eq.). The mixture was heated at 100 °C under N_2 for 20 h before being allowed to cool to room temperature. The mixture was concentrated *in vacuo* and the resulting solid suspended in water (400 mL) and collected by filtration. The resulting solid was further washed with water (2 × 400 mL) and MeOH (400 mL) before being dried *in vacuo* to afford the desired product **S4** as a colourless solid which was used without further purification (26.79 g, 41.88 mmol, 96%).

^{1}H NMR (500 MHz, $CDCl_3$): δ_H 7.80 (dd, *J* = 5.5, 3.0 Hz, 6H), 7.68 (dd, *J* = 5.4, 3.1 Hz, 6H), 4.94 (s, 6H), 3.10 (q, *J* = 7.5 Hz, 6H), 0.97 (t, *J* = 7.5 Hz, 9H); **^{13}C NMR** (101 MHz, $CDCl_3$): δ_C 168.4, 145.7, 134.0, 132.2, 129.6, 123.4, 37.6, 23.5, 15.9. Data in accordance to literature values.[27]

(2,4,6-Triethylbenzene-1,3,5-triyl)trimethanamine, C

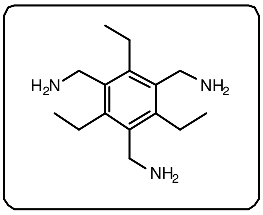

A modification of the procedure of Grawe *et al*. was used for this reaction.[23] To a suspension of 2,2',2''-((2,4,6-triethylbenzene-1,3,5-triyl)tris(methylene))tris(isoindoline-1,3-dione) **S4** (26.79 g, 41.88 mmol, 1.0 eq.) in a mixture of toluene (330 mL) and EtOH (670 mL) was added hydrazine hydrate in a single portion (15.6 mL, 50 wt% solution in water, 251.27 mmol, 6.0 eq.). The resulting mixture was heated at 90 °C for 44 h, at which point a large amount of solid had precipitated, before being allowed to cool to room temperature. The reaction mixture was concentrated *in vacuo* (not to dryness) and partitioned between an aqueous KOH solution (150 mL, 40 wt%) and $CHCl_3$ (400 mL). The aqueous layer was further extracted with $CHCl_3$ (2 × 200 mL) before the combined organic layers were dried (Na_2SO_4) and concentrated *in vacuo* to afford the desired triamine **C** as a pale yellow solid which was used without further purification (9.34 g, 37.45 mmol, 89%).

^{1}H NMR (500 MHz, $CDCl_3$): δ_H 3.87 (s, 6H), 2.82 (q, *J* = 7.6 Hz, 6H), 1.43 (br s, 6H), 1.23 (t, *J* = 7.5 Hz, 9H); **^{13}C NMR** (126 MHz, $CDCl_3$): δ_C 140.5, 137.6, 39.8, 22.7, 16.9. Data in accordance with literature values.[27]

Synthesis of aldehyde precursors

5-Bromoisophthalaldehyde, 2

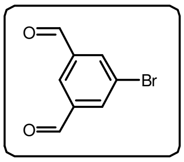

To a round bottomed flask, equipped with stirrer bar, was added isophthalaldehyde (50.00 g, 372.77 mmol, 1.0 eq.) followed by concentrated sulphuric acid (200 mL). The resulting mixture was heated to 65 °C, before direct heating was removed for the portionwise addition of *N*-bromosuccinimide (72.98 g, 410.05 mmol, 1.1 eq.) over 20 min. After complete addition, heating was resumed and the reaction was stirred at 65 °C for 19 h. The reaction was allowed to cool to room temperature, and was poured into ice (~1 L) and stirred. The mixture was left for 1 h before the resulting precipitate was collected by filtration. The collected solid was dissolved in DCM (1 L) and washed with water (2 x 200 mL). The organic layer was dried ($MgSO_4$) and hexane (500 mL) added, before the DCM was carefully removed *in vauo* to afford a beige precipitate which was collected by filtration. The resulting solid was washed with a 1:2 methanol:hexane mixture (300 mL) and dried in vacuo to afford 5-bromoisophthalaldehyde **2** which was used without further purification (43.14 g, 202.5 mmol, 54%).

^{1}H NMR (400 MHz, $CDCl_3$): δ_H 10.06 (s, 2H), 8.30 (t, *J* = 1.4 Hz, 1H), 8.26 (d, *J* = 1.4 Hz, 2H). Data in accordance with literature values.[28]

5-((Trimethylsilyl)ethynyl)isophthalaldehyde, 4

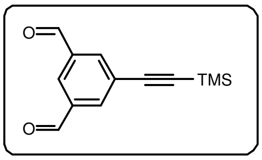

To an oven dried round bottomed flask equipped with stirrer bar, was added 5-bromoisophthalaldehyde **2** (2.00 g, 9.39 mmol, 1.0 eq.) and copper iodide (54 mg, 0.28 mmol, 0.03 eq.) before the flask was evacuated and refilled with N_2 (×3). Anhydrous THF (50 mL), triethylamine (10 mL) and trimethylsilylacetylene (2 mL, 14.08 mmol, 1.5 eq.) were added and the mixture degassed (N_2 bubbling, 15 mins) prior to the addition of $Pd(PPh_3)_4$ (542 mg, 0.47 mmol, 0.05 eq.). The resulting suspension was stirred at room temperature for 24 h before being diluted with water (100 mL) and the aqueous layer extracted with DCM (2 × 200 mL). The combined organic layers were dried ($MgSO_4$), passed through a small Celite® plug and concentrated *in vacuo*. Purification *via* column chromatography (0–10% EtOAc in hexane) afforded the desired product as a light brown oil (101 mg, 0.44 mmol, 5%). Note that this product seemed to suffer from stability issues during isolation and purification on silica gel.

R_f 0.32 (Hexane/EtOAc (9:1)); **^{1}H NMR** (500 MHz, $CDCl_3$): δ_H 10.07 (s, 2H), 8.30 (t, *J* = 1.6 Hz, 1H), 8.19 (d, *J* = 1.6 Hz, 2H), 0.28 (s, 9H); **^{13}C NMR** (101 MHz, $CDCl_3$): δ_C 190.5, 137.8, 137.1, 129.8, 125.9, 102.0, 98.6, -0.2. Data in accordance to literature values.[29]

[1,1'-Biphenyl]-3,5-dicarbaldehyde, 5

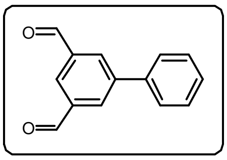

To an oven dried round bottomed flask equipped with a stirrer bar, was added 5-bromoisophthalaldehyde **2** (2.00 g, 9.39 mmol, 1.0 eq.) and phenylboronic acid (1.37 g, 11.27 mmol, 1.2 eq.) before the flask was evacuated and refilled with N_2 (x3). THF (100 mL) and 2M aqueous potassium carbonate (28.2 mL, 56.33 mmol, 6.0 eq.) were added and the mixture degassed (N_2 bubbling, 30 min) prior to the addition of $Pd(PPh_3)_4$ (542 mg 0.47 mmol, 0.05 eq.). The reaction was heated at reflux (80 °C) for 24 h before being cooled to room temperature. The THF was removed *in vacuo* and the remaining mixture diluted with $CHCl_3$ (100 mL) before being washed with deionised water (3 x 100 mL). The organic layer was isolated, dried over anhydrous $MgSO_4$ and filtered before the removal of solvent *in vacuo*. The crude product was purified by crystallisation from diethyl ether and hexane and dried *in vacuo* (1.24 g, 63%).

IR (ν_{max}/cm^{-1}) 3374, 3051, 3020, 2842, 2736, 1957, 1884, 1718, 1694, 1597, 1500, 1461, 1400, 1375, 1319, 1204, 1135, 1077; **^{1}H NMR** (400 MHz, $CDCl_3$): δ_H 10.18 (s, 2H), 8.37 (d, *J* = 1.5 Hz, 2H), 8.35 (t, *J* = 1.5 Hz, 1H), 7.71–7.65 (m, 2H), 7.55–7.49 (m, 2H), 7.49–7.43 (m, 1H); **^{13}C NMR** (101 MHz, $CDCl_3$): δ_C 191.2, 143.5, 138.4, 137.7, 133.1, 129.9, 129.4, 128.9, 127.3; **HRMS** (ES+) calc. for $C_{14}H_{10}O_2$ 210.0681; found $[M+15]^+$ 225.0923 — the $[M+15]^+$ mass ion has been observed previously with aromatic aldehydes in the presence of methanol in ES-MS.[30]

4'-Vinyl-[1,1'-biphenyl]-3,5-dicarbaldehyde, 6

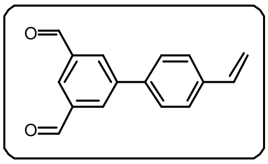

To an oven dried round bottomed flask equipped with a stirrer bar, was added 5-bromoisophthalaldehyde **2** (3.54 g, 16.61 mmol, 1.0 eq.) and 4-vinylphenylboronic acid (2.95 g, 19.94 mmol, 1.2 eq.) before the flask was evacuated and refilled with N_2 (x3). THF (300 mL) and 2 M aqueous potassium carbonate (49.9 mL, 99.68 mmol, 6.0 eq.) were added and the mixture degassed (N_2 bubbling, 30 min) prior to the addition of $Pd(PPh_3)_4$ (384 mg, 0.33 mmol, 0.02 eq.). The reaction was heated at reflux (80 °C) for 24 h before being cooled to room temperature. The THF was removed *in vacuo* and the remaining mixture diluted with chloroform (300 mL) before being washed with deionised water (3 x 250 mL). The organic layer was isolated, dried over anhydrous $MgSO_4$ and filtered before the removal of solvent *in vacuo* (2.78 g, 71%).

IR (ν_{max}/cm^{-1}) 3362, 3057, 2863, 2730, 1838, 1697, 1627, 1594, 1512, 1458, 1437, 1385, 1316, 1195, 1138; **^{1}H NMR** (400 MHz, $CDCl_3$): δ_H 10.17 (s, 2H), 8.36 (d, *J* = 1.5 Hz, 2H), 8.33 (t, *J* = 1.5 Hz, 1H), 7.65 (d, *J* = 8.4 Hz, 2H), 7.55 (d, *J* = 8.3 Hz, 2H), 6.77 (dd, *J* = 17.6, 10.9 Hz, 1H), 5.85 (d, *J* = 17.6 Hz, 1H), 5.34 (d, *J* = 11.1 Hz, 1H); **^{13}C NMR** (101 MHz, $CDCl_3$): δ_C 191.2, 143.0, 138.2, 137.7, 137.6, 136.1, 132.8, 129.9, 127.4, 127.2, 115.2; **HRMS** (ES+) calc. for $C_{16}H_{12}O_2$ 236.0837; found $[M+15]^+$ 251.1086 — the $[M+15]^+$ mass ion has been observed previously with aromatic aldehydes in the presence of methanol in ES-MS.[30]

4'-Nitro-[1,1'-biphenyl]-3,5-dicarbaldehyde, 7

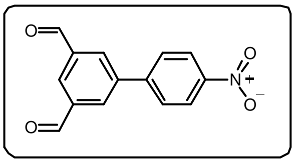

To an oven dried round bottomed flask equipped with a stirrer bar, was added 5-bromoisophthalaldehyde **2** (2.88 g, 13.53 mmol, 1.0 eq.) and 4-nitrophenylboronic acid (2.71 g, 16.23 mmol, 1.2 eq.) before the flask was evacuated and refilled with N_2 (x3). THF (300 mL) and 2M aqueous potassium carbonate (40.6 mL, 81.17 mmol, 6.0 eq.) were added and the mixture degassed (N_2 bubbling, 30 min) prior to the addition of $Pd(PPh_3)_4$ (312 mg, 0.27 mmol, 0.02 eq.). The reaction was heated at reflux (80 °C) for 24 h before being cooled to room temperature. The THF was removed *in vacuo* and the remaining mixture diluted with $CHCl_3$ (250 mL) before being washed with deionised water (3 x 250 mL). The organic layer was isolated, dried over anhydrous $MgSO_4$ and filtered before the removal of solvent *in vacuo*. Purification via column chromatography (3:1 hexane/EtOAc) afforded the desired product **7** as a static brown solid (1.86 g, 54%).

IR (v_{max}/cm^{-1}) 3500, 3371, 3059, 2869, 1805, 1702, 1597, 1572, 1515, 1439, 1388, 1343, 1292, 1261, 1237, 1143; **^{1}H NMR** (400 MHz, $CDCl_3$): δ_H 10.21 (s, 2H), 8.44 (t, *J* = 1.4 Hz, 1H), 8.41 (s, 1H), 8.40 (t, *J* = 2.2 Hz, 2H), 8.39–8.37 (m, 1H), 7.88–7.85 (m, 1H), 7.85–7.83 (m, 1H); **HRMS** (ES-, methanol & 0.1% formic acid) calc. for $C_{14}H_9NO_4$ 255.0532; found [M-] 255.0310.

5-(Pyridin-4-yl)isophthalaldehyde, 8

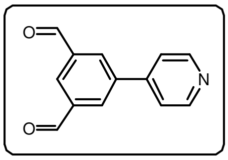

To an oven dried round bottomed flask equipped with a stirrer bar, was added 5-bromoisophthalaldehyde **2** (5.00 g, 23.47 mmol, 1.0 eq.) and pyridine-4-boronic acid (3.46 g, 28.17 mmol, 1.2 eq.) before the flask was evacuated and refilled with N_2 (x3). THF (300 mL) and 2M aqueous potassium carbonate (70.4 mL, 140.8 mmol, 6.0 eq.) were added and the mixture degassed (N_2 bubbling, 30 min) prior to the addition of $Pd(PPh_3)_4$ (542 mg, 0.47 mmol, 0.02 eq.). The reaction was heated at reflux (80 °C) for 24 h before being cooled to room temperature. The THF was removed *in vacuo* and the remaining mixture diluted with $CHCl_3$ (250 mL) before being washed with deionised water (3 x 250 mL). The organic layer was isolated, dried over anhydrous $MgSO_4$ and filtered before removal of the solvent *in vacuo*. Purification *via* column chromatography (5:1 EtOAc/hexane) afforded the desired product **8** as a static yellow solid (2.51g, 11.87 mmol, 50%).

R_f 0.43 (EtOAc/Hexane 5:1); **IR** (v_{max}/cm^{-1}) 3059, 3029, 1694, 1597, 1458, 1434, 1412, 1376, 1322, 1237, 1210, 1146, 1116; **^{1}H NMR** (400 MHz, $CDCl_3$): δ_H 10.20 (s, 2H), 8.78 (d, *J* = 4.5 Hz, 1H), 8.77 (d, *J* = 4.5 Hz, 1H), 8.44 (t, *J* = 1.4 Hz, 1H), 8.42 (d, *J* = 1.4 Hz, 2H), 7.61 (d, *J* = 4.5 Hz, 1H), 7.60 (d, *J* = 4.5 Hz, 1H); **^{13}C NMR** (101 MHz, $CDCl_3$): δ_C 190.6, 151.0, 145.7, 140.6, 138.0, 132.7, 131.5, 121.7; **HRMS** (ES+) calc. for $C_{13}H_9NO_2$ 211.0633; found $[M+H]^+$ 212.0717.

5-(Thiophen-3-yl)isophthalaldehyde,

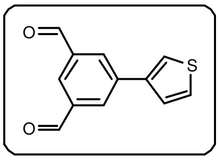

To an oven dried round bottomed flask equipped with a stirrer bar, was added 5-bromoisophthalaldehyde **2** (5.00 g, 23.47 mmol, 1.0 eq.) and 3-thienylboronic acid (3.61 g, 28.20 mmol, 1.2 eq.) before the flask was evacuated and refilled with N_2 (x3). THF (300 mL) and 2M aqueous potassium carbonate (70.4 mL, 140.8 mmol, 6.0 eq.) were added and the mixture degassed (N_2 bubbling, 30 min) prior to the addition of $Pd(PPh_3)_4$ (542 mg, 0.47 mmol, 0.02 eq.). The reaction was heated at reflux (80 °C) for 24 h before being cooled to room temperature. The THF was removed *in vacuo* and the remaining mixture diluted with $CHCl_3$ (200 mL) before being washed with deionised water (3 x 200 mL). The organic layer was isolated, dried over anhydrous $MgSO_4$ and filtered before removal of the solvent *in vacuo* affording the desired product **9** as a light brown solid and required no further purification (3.39 g, 67%).

IR (ν_{max}/cm^{-1}) 3090, 2815, 2758, 2733, 1705, 1594, 1439, 1409, 1358, 1298, 1231, 1198, 1128, 1074; **^{1}H NMR** (400 MHz, $CDCl_3$): δ_H 10.15 (s, 2H), 8.35 (d, *J* = 1.5 Hz, 2H), 8.27 (t, *J* = 1.5 Hz, 1H), 7.67–7.65 (m, 1H), 7.49–7.47 (m, 2H); **^{13}C NMR** (101 MHz, $CDCl_3$): δ_C 191.2, 139.6, 138.0, 137.7, 132.0, 129.8, 127.6, 126.0, 122.6; **HRMS** (ES+) calc. for $C_{12}H_8O_2S$ 216.0245; found $[M+15]^+$ 231.0486 – the $[M+15]^+$ mass ion has been observed previously with aromatic aldehydes in the presence of methanol in ES-MS.[30]

2,5-Dihydroxyterephthalaldehyde, 12

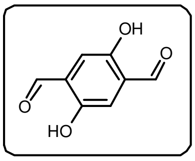

To an oven dried round bottomed flask equipped with stirrer bar was added 2,5-dimethoxybenzene-1,4-dicarbaldehyde (0.50 g, 2.57 mmol, 1.0 eq.) before the flask was evacuated and refilled with N_2 (×3). Anhydrous DCM (74 mL) was added and once fully dissolved, the solution was cooled to 0 °C in an ice-bath. To the cooled pale yellow solution was added a solution of boron tribromide (26 mL, 1 M in DCM, 25.75 mmol, 10.0 eq.) dropwise, at which point a colour change was observed to bright orange. After complete addition, the reaction was allowed to warm to room temperature and stirred overnight for 21 h before any residual BBr_3 was quenched by the dropwise addition of water whilst cooling in an ice-bath. Once quenched, water (100 mL) was added and the organic layer separated. The aqueous layer was extracted with DCM (2 × 200 mL) and the combined organic layers dried ($MgSO_4$) and concentrated *in vacuo* to afford the desired product **12** as an orange crystalline solid which was used without further purification (427 mg, quant.).

^{1}H NMR (400 MHz, $CDCl_3$): δ_H 10.22 (s, 2H), 9.96 (s, 2H), 7.24 (s, 2H); **^{13}C NMR** (101 MHz, $CDCl_3$): δ_C 196.6, 153.4, 125.3, 121.8. Data in accordance to literature values.[31]

3-Hydroxy-[1,1'-biphenyl]-4,4'-dicarbaldehyde, 16

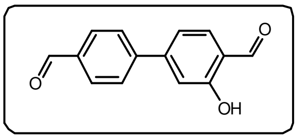

To an oven dried round bottomed flask equipped with stirrer bar was added 4-bromo-2-hydroxybenzaldehyde (488 mg, 2.43 mmol, 1.0 eq.) and 4-formylphenylboronic acid (436 mg, 2.91 mmol, 1.2 eq.) before being dissolved in THF (20 mL). This solution was degassed (N_2 bubbling, 30 min) before the addition of a 2M aqueous K_2CO_3 solution (7.3 mL, 14.56 mmol, 6.0 eq.) and $Pd(PPh_3)_4$ (56 mg, 0.048 mmol, 0.02 eq.). The reaction was heated at reflux (80 °C) for 18 h before being allowed to cool to room temperature and diluted with water (30 mL). The product was extracted with EtOAc (4 × 50 mL) and the combined organic layers dried ($MgSO_4$) and concentrated *in vacuo* to afford the desired product **16** as a vivid yellow solid which was used without further purification (410 mg, 1.81 mmol, 74%).

IR (ν_{max}/cm^{-1}) 2361, 2340, 1697, 1651, 1605, 1560, 1198, 840, 796; **^{1}H NMR** (400 MHz, $CDCl_3$): δ_H 11.12 (s, 1H), 10.08 (s, 1H), 9.96 (s, 1H), 7.98 (d, *J* = 8.4 Hz, 2H), 7.78 (d, *J* = 8.3 Hz, 2H), 7.67 (d, *J* = 8.0 Hz, 1H), 7.28 (dd, *J* = 8.0, 1.6 Hz, 1H), 7.25 (d, *J* = 1.6 Hz, 1H); **^{13}C NMR** (101 MHz, $CDCl_3$): δ_C 196.2, 191.8, 162.0, 148.3, 145.3, 136.4, 134.4, 130.5, 128.2, 120.3, 119.2, 116.5; **HRMS** (CI+) calc. for $C_{14}H_{10}O_3$ 226.0630; found $[M+H]^+$ 227.0708.

3-Chloro-[1,1'-biphenyl]-4,4'-dicarbaldehyde, 17

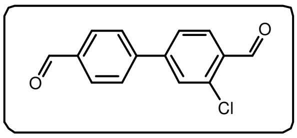

To an oven dried round bottomed flask equipped with stirrer bar was added 4-bromo-2-chlorobenzaldehyde (2.00 g, 9.11 mmol, 1.0 eq.) and 4-formylphenyl boronic acid (1.64 g, 10.94 mmol, 1.2 eq.) before being dissolved in THF (50 mL). This solution was degassed (N_2 bubbling, 30 min) before the addition of a 2M aqueous K_2CO_3 solution (27.3 mL, 54.68 mmol, 6.0 eq.) and $Pd(PPh_3)_4$ (210 mg, 0.18 mmol, 0.02 eq.). The reaction was heated at reflux (80 °C) for 18 h before being allowed to cool to room temperature and diluted with water (50 mL). The product was extracted with EtOAc (3 × 100 mL) and the combined organic layers dried ($MgSO_4$) and concentrated *in vacuo*. Purification by column chromatography (1:4 EtOAc/Hexanes) affords the desired product **17** as an off-white powder (310 mg, 1.27 mmol, 14%).

IR (ν_{max}/cm^{-1}) 2860, 2728, 2361, 2342, 1703, 1684, 1599, 1386, 1210, 1051, 833, 817 ; **^{1}H NMR** (400 MHz, $CDCl_3$): δ_H 10.52 (d, *J* = 0.7 Hz, 1H), 10.09 (s, 1H), 8.04 (d, *J* = 8.1 Hz, 1H), 8.00 (d, *J* = 8.4 Hz, 2H), 7.78 (d, *J* = 8.3 Hz, 2H), 7.73 (d, *J* = 1.7 Hz, 1H), 7.65 (ddd, *J* = 8.1, 1.7, 0.7 Hz, 1H); **^{13}C NMR** (101 MHz, $CDCl_3$): δ_C 191.7, 189.4, 146.7, 144.3, 138.6, 136.5, 132.0, 130.6, 130.1, 129.5, 128.1, 126.4; **HRMS** (CI+) calc. for $C_{14}H_9ClO_2$ 244.0291; found $[M+H]^+$ 245.0372.

4,4'-(Ethyne-1,2-diyl)dibenzaldehyde, 18

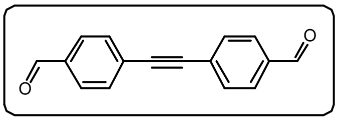

To an oven dried round bottomed flask equipped with stirrer bar was added 4-bromobenzaldehyde (1.01 g, 5.49 mmol, 1.0 eq.), 4-ethynylbenzaldehyde (1.00 g, 7.68 mmol, 1.4 eq.) and copper iodide (104 mg, 0.55 mmol, 0.1 eq.) before the flask was evacuated and refilled with N_2 (×3). Triethylamine (40 mL) was added and the mixture degassed (N_2 bubbling, 15 mins) prior to the addition of $Pd(PPh_3)_4$ (317 mg, 0.27 mmol, 0.05 eq.). The resulting suspension was heated at reflux for 24 h before being allowed to cool to room temperature. The reaction was diluted with water (200 mL) and the product extracted with DCM (2 × 200 mL) before the combined organic layers were dried ($MgSO_4$) and passed through a small Celite® plug. Hexane (200 mL) was added and the DCM carefully removed *in vacuo* to afford a dark yellow precipitate. The precipitate was collected by filtration and dried *in vacuo* to afford the desired product **18** which was used without further purification (1.22 g, 5.23 mmol, 95%).

^{1}H NMR (500 MHz, $CDCl_3$): δ_H 10.03 (s, 2H), 7.89 (d, *J* = 8.2 Hz, 4H), 7.70 (d, *J* = 8.1 Hz, 4H); **^{13}C NMR** (101 MHz, $CDCl_3$): δ_C 191.5, 136.0, 132.4, 129.7, 128.8, 92.2. Data in accordance with literature values.[32]

[1,1':4',1''-Terphenyl]-4,4''-dicarbaldehyde, 19

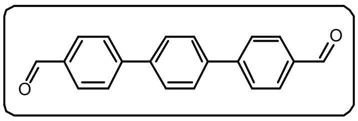

To an oven dried round bottomed flask equipped with stirrer bar was added 1,4-dibromobenzene (2.00 g, 8.48 mmol, 1.0 eq.) and 4-formylphenylboronic acid (3.05 g, 20.35 mmol, 2.4 eq.) before being dissolved in THF (100 mL). This solution was degassed (N_2 bubbling, 30 mins) prior to the addition of a 2M aqueous K_2CO_3 solution (50.9 mL, 101.74 mmol, 12.0 eq.) and $Pd(PPh_3)_4$ (392 mg, 0.34 mmol, 0.04 eq.). The reaction was heated at reflux (80 °C) for 18 h before being allowed to cool to room temperature, at which point a large quantity of small crystals formed. These crystals were collected by filtration under reduced pressure. The resulting filtrate was diluted with Et_2O (300 mL), yielding an additional, larger crop of crystals which were again recovered *via* filtration under reduced pressure and combined with the initial crop to afford the desired product **19** as beige crystals which were used without further purification (2.10 g, 7.33 mmol, 86%).

^{1}H NMR (500 MHz, $CDCl_3$): δ_H 10.08 (s, 2H), 7.99 (d, *J* = 8.3 Hz, 4H), 7.82 (d, *J* = 8.2 Hz, 4H), 7.77 (s, 4H); **^{13}C NMR** (126 MHz, $CDCl_3$): δ_C 192.0, 146.4, 139.9, 135.6, 130.5, 128.1, 127.8. Data in accordance with literature values.[33]

5'-(4-Formylphenyl)-[1,1':3',1''-terphenyl]-4,4''-dicarbaldehyde, 24

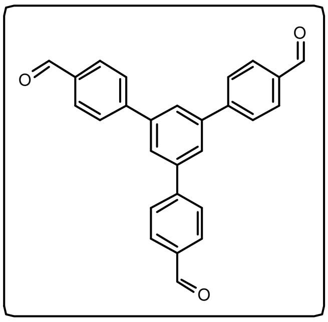

To an oven-dried round bottomed flask equipped with stirrer bar was added 1,3,5-tribromobenzene (7.29 g, 23.15 mmol, 1.0 eq.) and 4-formylphenylboronic acid (12.5 g, 83.36 mmol, 3.6 eq.) before the flask was evacuated and refilled with N_2 (×3). Isopropanol (140 mL) was added and the mixture degassed (N_2 bubbling, 15 mins) prior to the addition of $Pd(PPh_3)_4$ (1.34 g, 1.16 mmol, 0.05 eq.). The resulting suspension was heated at 50 °C until all the solids had dissolved, at which point a 2 M aqueous $NaHCO_3$ solution (43 mL) was added. The resulting mixture was heated at reflux for 17 h before being allowed to cool to room temperature. The precipitated solid was collected by filtration and washed with water (100 mL) and Et_2O (200 mL) before being dissolved in $CHCl_3$ (500 mL). The resulting solution was stirred over activated charcoal for 5 min, dried ($MgSO_4$), filtered and concentrated *in vacuo*. The resulting solid was slurried in Et_2O (400 mL), stirred at room temperature overnight, collected by filtration and dried *in vacuo* to afford the desired product **24** as a pale brown solid (6.77 g, 17.34 mmol, 75%).

^{1}H NMR (500 MHz, $CDCl_3$): δ_H 10.11 (s, 3H), 8.03 (d, *J* = 7.6 Hz, 6H), 7.91 (s, 3H), 7.88 (d, *J* = 7.4 Hz, 6H); **^{13}C NMR** (101 MHz, $CDCl_3$): δ_C 191.9, 146.4, 141.7, 135.9, 130.6, 128.1, 126.6. Data in accordance to literature values.[34]

4,4',4''-(Benzene-1,3,5-triyltris(ethyne-2,1-diyl))tribenzaldehyde, 25

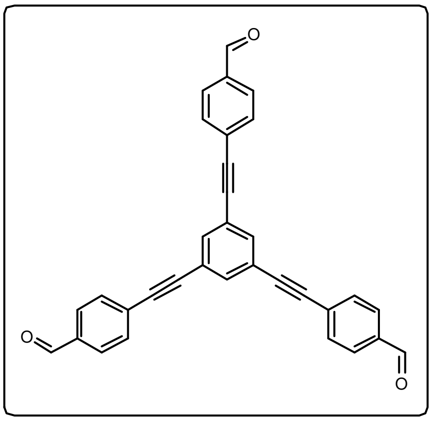

To an oven-dried round bottom flask equipped with stirrer bar was added 1,3,5-tribromobenzene (1.44 g, 4.57 mmol, 1.0 eq.), copper iodide (175 mg, 0.92 mmol, 0.2 eq.), triphenylphosphine (241 mg, 0.92 mmol, 0.2 eq.) and 4-ethynylbenzaldehyde (2.50 g, 19.2 mmol, 4.2 eq.). The flask was evacuated under vacuum for 10 min, before being refilled with N_2. Triethylamine (100 mL) was added and the mixture degassed (N_2 bubbling, 20 mins) prior to the addition of $PdCl_2(PPh_3)_2$ (323 mg, 0.092 mmol, 0.1 eq.). The resulting suspension was heated at reflux overnight before being allowed to cool to ambient temperature. The reaction was diluted with water (150 mL), and the solid collected by filtration, washed with water, and dried on the filter. The resulting solid was purified *via* column chromatography (0–10% EtOAc in DCM), and the product containing fractions concentrated to ~25 mL. The EtOAc suspension was filtered, and the solid washed with further EtOAc, before the solid was dried *in vacuo* to afford the desired product **25** as a colourless solid (1.50 g, 3.2 mmol, 71%).

IR (ν_{max}/cm^{-1}) 2361, 2340, 1703, 1602, 1560, 1207, 1165, 822; **^{1}H NMR** (400 MHz, $CDCl_3$): δ_H 10.04 (s, 3H), 7.90 (d, *J* = 8.3 Hz, 6H), 7.74 (s, 3H), 7.69 (d, *J* = 8.2 Hz, 6H); **^{13}C NMR** (101 MHz, $CDCl_3$): δ_C 191.5, 136.0, 135.0, 132.4, 129.8, 128.9, 123.8, 91.3, 90.1.

5''-(4'-Formyl-[1,1'-biphenyl]-4-yl)-[1,1':4',1'':3'',1''':4''',1''''-quinquephenyl]-4,4''''-dicarbaldehyde, 26

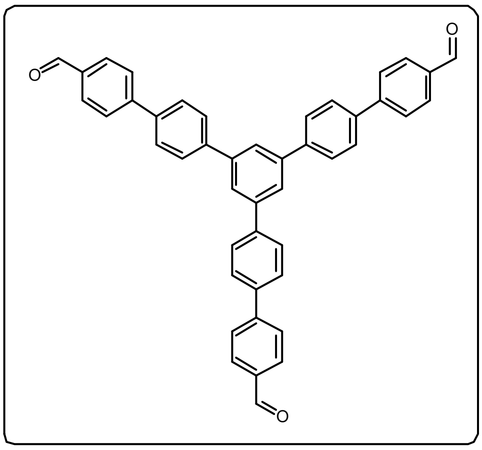

A solution of 1,3,5-tris(4-bromophenyl)benzene (4.02 g, 7.41 mmol, 1.0 eq.), 4-formylphenylboronic acid (5.00 g, 33.3 mmol, 4.5 eq.) and K_2CO_3 (6.14 g, 44.50 mmol, 6.0 eq.), in a mixture of toluene (142 mL), MeOH (47 mL) and water (40.6 mL), was degassed (N_2 bubbling, 10 min) prior to the addition of $Pd(PPh_3)_4$ (0.45 g, 0.39 mmol, 0.05 eq.). The resulting mixture was heated at reflux for 2 days. After cooling to ambient temperature, the solid was collected by filtration, washed with water, and dried on the filter. The solid was dissolved in DCM, and filtered through a pad of silica (1:9 EtOAc/DCM). The combined washes were concentrated to ~50 mL and the resulting solid was collected by filtration. The solid was dried in vacuo to afford the desired product **26** (1.68 g, 2.7 mmol, 37%).

^{1}H NMR (400 MHz, $CDCl_3$): δ_H 10.09 (s, 3H), 8.00 (d, *J* = 8.3 Hz, 6H), 7.91 (s, 3H), 7.89–7.82 (m, 12H), 7.80 (d, *J* = 8.4 Hz, 6H); **^{13}C NMR** (101 MHz, $CDCl_3$): δ_C 192.0, 146.6, 142.0, 141.2, 139.2, 135.5, 130.5, 128.12, 128.07, 127.7, 125.5. Data in accordance with literature values.[35]

Synthesis of model cage topologies

B11$_{[4+6]}$

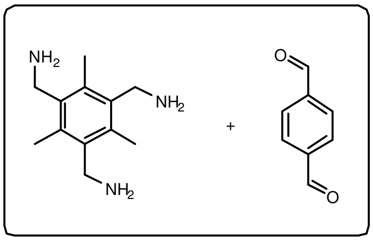

Method A: A solution of terephthalaldehyde **11** (194 mg, 1.45 mmol, 6.0 eq.) and (2,4,6-trimethylbenzene-1,3,5-triyl)trimethanamine **B** (250 mg, 1.21 mmol, 5.0 eq.) dissolved in $CHCl_3$ (260 mL) was heated at 60 °C for 3 days. Reaction allowed to cool to room temperature and solution filtered to remove any insoluble precipitate before concentration *in vacuo*. The resulting solid was re-dissolved in DCM (100 mL) and hexane (100 mL) was added. The DCM was carefully removed *in vacuo* to give a cream precipitate which was collected by filtration and dried *in vacuo* to afford the cage product **B11** as an off-white solid (254 mg, 0.18 mmol, 74%).

Method B: A solution of terephthalaldehyde **11** (194 mg, 1.45 mmol, 6.0 eq.) and (2,4,6-trimethylbenzene-1,3,5-triyl)trimethanamine **B** (250 mg, 1.21 mmol, 5.0 eq.) dissolved in DCM (260 mL) was heated at 40 °C for 2 days. Reaction allowed to cool to room temperature and solution filtered to remove any insoluble precipitate before the addition of hexane (200 mL). The DCM was carefully removed *in vacuo* to give a colourless precipitate which was collected by filtration and dried *in vacuo* to afford the cage product **B11** as a cream solid (224 mg, 0.16 mmol, 65%).

IR (ν_{max}/cm^{-1}) 3391 (br), 2868, 1636, 1566, 1449, 1374, 1315, 1218; **^{1}H NMR** (500 MHz, $CDCl_3$): δ_H 8.30 (t, *J* = 1.6 Hz, 12H), 7.75 (s, 24H), 4.93 (s, 24H), 2.37 (s, 36H); **^{13}C NMR** (101 MHz, $CDCl_3$): δ_C 160.4, 138.3, 137.7, 133.2, 128.5, 59.2, 16.4; **HRMS** (ES+) calc. for [4+6] cage $C_{96}H_{96}N_{12}$ 1417.7912; found $[M+H]^+$ 1418.8056, $[M+2H]^{2+}$ 709.9087 and $[M+H+Na]^{2+}$ 720.8998.

Subsequent crystallisation of **B11** further confirmed that the cage had formed the tetrahedral **Tri^{4}Di^6** topology; for SCXRD structures, see **Supplementary Fig. 10 and 11**.

B1[2+3]

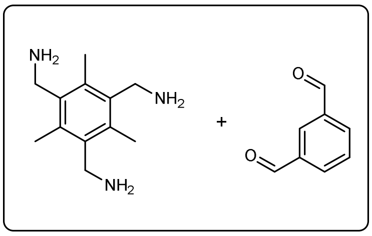

Method A: A solution of isophthalaldehyde **1** (80 mg, 0.60 mmol, 3.0 eq.) and (2,4,6-trimethylbenzene-1,3,5-triyl)trimethanamine **B** (125 mg, 0.602 mmol, 3.0 eq.) dissolved in $CHCl_3$ (130 mL) was heated at 60 °C for 4 days. Reaction allowed to cool to room temperature before concentration *in vacuo*. The resulting solid was re-dissolved in DCM (30 mL) and any insoluble precipitate removed by filtration before the addition of hexane (30 mL) to the filtrate. The DCM was carefully removed *in vacuo* to give a cream precipitate which was collected by filtration and dried in the vacuum oven at 50 °C overnight to afford the cage product **B1** as a cream solid (44 mg, 0.062 mmol, 31%).

Method B: A solution of isophthalaldehyde **1** (161 mg, 1.205 mmol, 3.0 eq.) and (2,4,6-trimethylbenzene-1,3,5-triyl)trimethanamine **B** (250 mg, 1.205 mmol, 3.0 eq.) dissolved in DCM (260 mL) was heated at 40 °C for 3 days. Reaction allowed to cool to room temperature and solution filtered to remove any insoluble precipitate before the addition of hexane (200 mL). The DCM was carefully removed *in vacuo* to give a precipitate which was collected by filtration and dried *in vacuo* to afford the cage product **B1** as a cream solid (139 mg, 0.196 mmol, 49%).

IR (ν_{max}/cm^{-1}) 2963, 2858, 1643, 1259, 1095, 1018, 800; **^{1}H NMR** (400 MHz, $CDCl_3$): δ_H 8.12 (dd, *J* = 7.7, 1.4 Hz, 6H), 7.85 (s, 6H), 7.50 (t, *J* = 7.8 Hz, 3H), 7.14 (s, 3H), 5.07 (d, *J* = 1.9 Hz, 12H), 2.12 (s, 18H); **^{13}C NMR** (101 MHz, $CDCl_3$): δ_C 158.2, 138.0, 136.7, 132.2, 130.9, 129.4, 128.6, 56.5, 16.4; **HRMS** (ES+) calc. for [2+3] cage $C_{48}H_{48}N_6$ 708.3940; found $[M+H]^+$ 709.4045. Data analogous to literature values in $CDCl_3$/MeOD, 9:1.[2]

B23$_{[4+4]}$

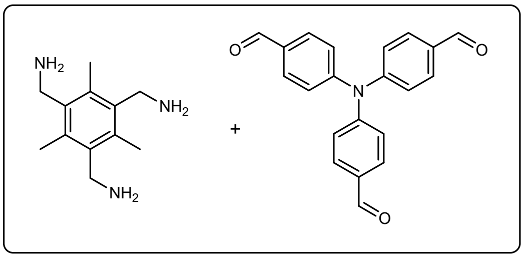

Method A: A solution of tris(4-formylphenyl)amine **23** (158 mg, 0.482 mmol, 4.0 eq.) and (2,4,6-trimethylbenzene-1,3,5-triyl)tri-methanamine **B** (125 mg, 0.602 mmol, 5.0 eq.) dissolved in $CHCl_3$ (130 mL) was heated at 60 °C for 4 days. Reaction allowed to cool to room temperature and solution filtered to remove any insoluble precipitate before concentration *in vacuo*. The resulting solid was re-dissolved in DCM (100 mL) and hexane (100 mL) added. The DCM was carefully removed *in vacuo* and the precipitated solid collected by filtration and dried *in vacuo* to afford the cage product **B23** as a yellow solid (219 mg, 0.113 mmol, 94%).

IR (ν_{max}/cm^{-1}) 3389 (br), 2965, 1637, 1597, 1505, 1320, 1262, 1102, 1025, 801; **^{1}H NMR** (400 MHz, $CDCl_3$): δ_H 8.18 (s, 12H), 7.54 (d, *J* = 8.6 Hz, 24H), 6.96 (d, *J* = 8.6 Hz, 24H), 4.88 (s, 24H), 2.43 (s, 36H); **^{13}C NMR** (101 MHz, $CDCl_3$): δ_C 159.4, 149.0, 136.6, 133.4, 131.9, 129.4, 124.2, 58.9, 16.0; **HRMS** (ES+) calc. for [4+4] cage $C_{132}H_{120}N_{16}$ 1929.9913; found $[M+H]^+$ 1931.0081, $[M+2H]^{2+}$ 966.0111 and $[M+3H]^{3+}$ 644.3445.

High-Throughput Screen

All triamine and aldehyde precursors dissolved in $CDCl_3$ to make stock solutions (2.5–5 mg/mL) for use in high-throughput screening (**Supplementary Table 16**). Over 3 runs on a Chemspeed Accelerator SLT-100 platform, the required volume of each triamine stock solution, followed by the required volume of each aldehyde stock solution, was added to jacketed reactors (27 mL maximum volume) *via* liquid dispensing, followed by additional $CDCl_3$ to make each total volume up to 13 mL (**Supplementary Table 17-19**). The resulting solutions were vortexed at 800 rpm and heated to 65 °C for 3 days before being allowed to cool to room temperature. All reactions removed from reactor vessels and filtered through a small cotton wool plug to remove any insoluble precipitate prior to analysis.

Scale-up of cage hits

General procedure: A solution of aldehyde (**1–21**) and triamine (**A–C**) was stirred in either DCM or $CHCl_3$ (4.6 mM with respect to mmol of triamine) at 40 °C or 65 °C respectively, for 1–4 days. The reaction was allowed to cool to room temperature and filtered to remove any insoluble precipitates, before 40 mL was removed for direct use in a crystallisation screens. For DCM: to the remainder of the filtrate was added an equivalent volume of hexane and the DCM carefully removed *in vacuo*. For $CHCl_3$: to the remainder of the filtrate was added an excess of hexane and the solution concentrated to ~100 mL, before an additional excess of hexane was added and re-concentrated to ~100 mL. The resulting precipitate was collected by filtration, dried *in vacuo* and the cage product characterised.

Targeted Cage	Aldehyde (eq.)	Triamine (eq.)
**Tri^2Di^3**	3.0	3.0
**Tri^4Di^6**	6.0	5.0
**Tri^4Tri^4**	4.0	5.0

For cages that were not scaled up, proved unstable to isolation, and/or gave a mostly or completely insoluble product, the cage was characterised in solution and the data presented is that from the robot screen where the reaction had proceeded cleanly. For all other data see **Supplementary Tables 21-23 and Supplementary Figs. 50-72**.

SCXRD Screen: The non-isolated cage solution (40 mL) was split for vial-in-vial slow diffusion crystallisation studies with a range of anti-solvents including methanol, hexane, THF, acetone, ethyl acetate, acetonitrile, isopropanol, o-/m-/p-xylene, ethanol, petroleum ether, toluene, pentane, and diethyl ether.

A1$_{[2+3]}$

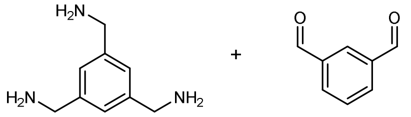

Synthesised according to the general scale-up procedure using benzene-1,3,5-triyltrimethanamine **A** (100 mg, 0.61 mmol, 3.0 eq.) and isophthalaldehyde **1** (81 mg, 0.61 mmol, 3.0 eq.) in DCM (130 mL) for 3 days to afford **A1** as a colourless solid (75 mg mass recovery). Isolated product proved to be poorly soluble suggesting some decomposition of cage product had occurred while the ^{1}H NMR spectrum indicates that residual triamine **A** is present.

IR (ν_{max}/cm^{-1}) 2965, 1644, 1260, 1096, 1022, 798; **^{1}H NMR** (400 MHz, $CDCl_3$) δ_H 8.32 (s, 6H), 7.76 (dd, *J* = 7.6, 1.6 Hz, 6H), 7.43 (t, *J* = 7.6 Hz, 3H), 7.19 (s, 6H), 7.07 (s, 3H), 4.79 (s, 12H); **HRMS** (ES+) calc. for [2+3] cage $C_{42}H_{36}N_6$ 624.3001; found $[M+H]^+$ 625.3089 and $[M+Na]^+$ 647.2894.

A11$_{[4+6]}$

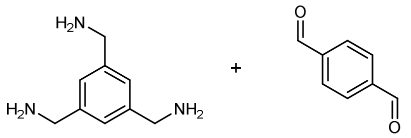

Synthesised according to the general scale-up procedure using benzene-1,3,5-triyltrimethanamine **A** (100 mg, 0.61 mmol, 5.0 eq.) and terephthalaldehyde **11** (97 mg, 0.72 mmol, 6.0 eq.) in DCM (130 mL) for 3 days to afford **A11** as a pale yellow solid (23 mg mass recovery).

IR (ν_{max}/cm^{-1}) 2962, 2907, 1644, 1413, 1262, 1096, 1025, 865, 806; **^{1}H NMR** (400 MHz, $CDCl_3$) δ_H 8.39 (s, 12H), 7.72 (s, 24H), 7.18–7.13 (m, 12H), 4.89 (s, 24H); **HRMS** (ES+) calc. for [4+6] cage $C_{84}H_{72}N_{12}$ 1248.6003, found $[M+2H]^{2+}$ 625.3084.

A12$_{[2+3]}$

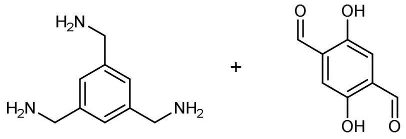

Data from the high-throughput screen: **IR** (ν_{max}/cm^{-1}) 3358 (br), 2963, 2906 (br), 1630, 1513, 1446, 1358, 1263, 1161, 1090, 1048, 865, 801; **^{1}H NMR** (400 MHz, $CDCl_3$) δ_H 8.48–8.26 (m, 6H), 7.21–7.10 (m, 6H), 6.83 (apparent dd, *J* = 11.7, 1.9 Hz, 6H), 4.80 (d, *J* = 15.8 Hz, 12H) *NB.* Multiple singlets apparent for all protons, likely due to the different environments from H-bonding within the cage molecule; **HRMS** (ES+) calc. for [2+3] cage $C_{42}H_{36}N_6O_6$ 720.2696, found $[M+H]^+$ 721.2759.

B2$_{[2+3]}$

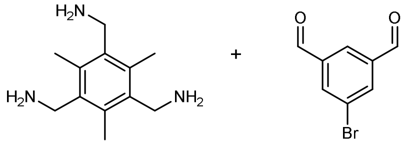

Synthesised according to the general procedure using (2,4,6-trimethylbenzene-1,3,5-triyl)trimethanamine **B** (500 mg, 2.41 mmol, 3.0 eq.) and 5-bromoisophthalaldehyde **2** (514 mg, 2.41 mmol, 3.0 eq.) in DCM (520 mL) for 4 days to afford **B2** as pale yellow solid (454 mg, 0.48 mmol, 60%).

IR (ν_{max}/cm^{-1}) 2963, 1644, 1569, 1259, 1096, 1023, 868, 800; **^{1}H NMR** (400 MHz, $CDCl_3$) δ_H 8.23 (d, *J* = 1.1 Hz, 6H), 7.76 (t, *J* = 2.0 Hz, 6H), 7.05 (s, 3H), 5.07 (d, *J* = 1.9 Hz, 12H), 2.09 (S, 18H); **^{13}C NMR** (101 MHz, $CDCl_3$) δ_C 157.0, 138.4, 138.0, 132.1, 131.5, 129.0, 124.3, 56.6, 16.4; **HRMS** (ES+) calc. for [2+3] cage $C_{48}H_{45}N_6Br_3$ 946.1226, found $[M+H]^+$ 947.1401 and $[M+2H]^{2+}$ 474.0748.

B4$_{[2+3]}$

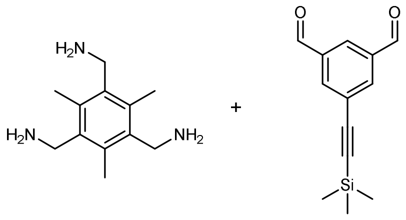

Data from the high-throughput screen: **IR** (ν_{max}/cm^{-1}) 2965, 2901, 2156, 1644, 1592, 1445, 1373, 1298, 1259, 1097, 1021, 957, 856, 843; **^{1}H NMR** (400 MHz, $CDCl_3$) δ_H 8.13 (d, J = 0.9 Hz, 6H), 8.00 (s, 3H), 7.87 (s, 6H), 5.03 (s, 12H), 2.11 (s, 18H), 0.23 (s, 27H); **HRMS** (ES+) calc. for [2+3] cage $C_{63}H_{72}N_6Si_3$ 996.5126, found $[M+2H]^{2+}$ 499.2640.

B5$_{[2+3]}$

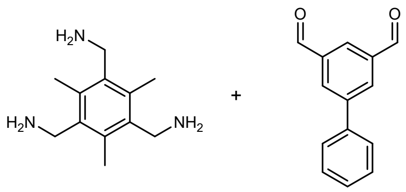

Data from the high-throughput screen: **IR** (ν_{max}/cm^{-1}) 2961, 2905, 1646, 1596, 1499, 14445, 1414, 1331, 1261, 1083, 1013, 864; **^{1}H NMR** (400 MHz, $CDCl_3$) δ_H 8.38 (d, J = 1.0 Hz, 6H), 7.94 (s, 6H), 7.73 (d, J = 7.2 Hz, 6H), 7.45 (dd, J = 10.1, 4.8 Hz, 6H), 7.37 (dd, J = 8.3, 6.3 Hz, 3H), 7.17 (s, 3H), 5.11 (s, 12H), 2.17 (s, 18H); **HRMS** (ES+) calc. for [2+3] cage $C_{66}H_{60}N_6$ 936.4879, found $[M+H]^{+}$ 937.5065 and $[M+2H]^{2+}$ 469.2590.

B6$_{[2+3]}$

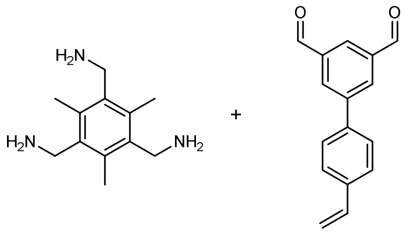

Data from the high-throughput screen: **IR** (ν_{max}/cm^{-1}) 2963, 1644, 1510, 1439, 1260, 1094, 1016, 844, 805; **^{1}H NMR** (400 MHz, $CDCl_3$) δ_H 8.38 (s, 6H), 7.93 (s, 6H), 7.70 (d, J = 7.6 Hz, 6H), 7.48 (d, J = 7.2 Hz, 6H), 7.16 (s, 3H), 6.76 (dd, J = 17.8, 10.8 Hz, 3H), 5.81 (d, J = 17.5 Hz, 3H), 5.29 (d, J = 10.2 Hz, 3H), 5.11 (s, 12H), 2.17 (s, 18H); **HRMS** (ES+) calc. for [2+3] cage $C_{72}H_{66}N_6$ 1014.5349, found $[M+H]^{+}$ 1015.5524.

B8$_{[2+3]}$

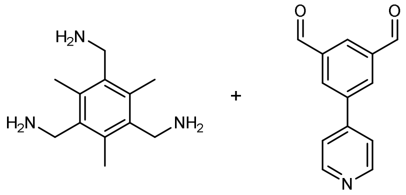

To a dried round-bottomed flask was added 5-(pyridin-4-yl)isophthalaldehyde **8** (2.00 g, 9.47 mmol, 3.0 eq.) and (2,4,6-trimethylbenzene-1,3,5-triyl)trimethanamine **B** (1.96 g, 9.47 mmol, 3.0 eq.) before being dissolved in $CHCl_3$ (1000 mL). The reaction was allowed to stir at room temperature for three days before the addition of hexane (1000 mL). The chloroform was selectively removed under reduced pressure until precipitation of the product occurred. The resulting precipitate was collected via filtration before being dried under reduced pressure overnight to yield an off-white solid (2.31 g, 2.46 mmol, 78%).

IR (ν_{max}/cm^{-1}) 3362 (br), 2963, 1644, 1595, 1562, 1433, 1263, 1094, 1023, 801; **^{1}H NMR** (400 MHz, $CDCl_3$) δ_H 8.68 (dd, *J* = 4.5, 1.6 Hz, 6H), 8.32 (d, *J* = 1.3 Hz, 6H), 8.09 (t, *J* = 1.8 Hz, 6H), 7.63 (dd, *J* = 4.6, 1.6 Hz, 6H), 7.59 (s, 3H), 5.09 (d, *J* = 1.5 Hz, 12H), 2.18 (s, 18H); **HRMS** (ES+) calc. for [2+3] cage $C_{63}H_{57}N_9$ 939.4737, found $[M+H]^+$ 940.4987 and $[M+2H]^{2+}$ 470.7560.

B9$_{[2+3]}$

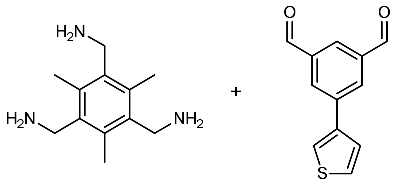

Data from the high-throughput screen: **IR** (ν_{max}/cm^{-1}) 3364 (br), 2963, 2903, 1640, 1595, 1439, 1355, 1260, 1094, 1023, 798; **^{1}H NMR** (400 MHz, $CDCl_3$) δ_H 8.25 (d, *J* = 1.0 Hz, 6H), 8.03 (s, 6H), 7.63 (dd, *J* = 2.9, 1.2 Hz, 3H), 7.52 (dd, *J* = 5.0, 1.3 Hz, 3H), 7.43 (s, 3H), 7.40 (dd, *J* = 5.1, 2.9 Hz, 3H), 5.07 (s, 12H), 2.17 (s, 18H); **HRMS** (ES+) calc. for [2+3] cage $C_{60}H_{54}N_6S_3$ 954.3572, found $[M+H]^+$ 955.3744.

B13$_{[4+6]}$

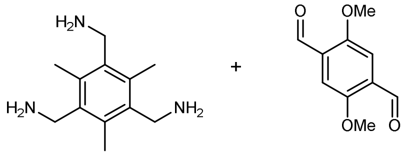

Synthesised according to the general procedure using (2,4,6-trimethylbenzene-1,3,5-triyl)trimethanamine **B** (500 mg, 2.41 mmol, 5.0 eq.) and 2,5-dimethoxybenzene-1,4-dicarboxaldehyde **13** (562 mg, 2.89 mmol, 6.0 eq.) in $CHCl_3$ (520 mL) for 3 days to afford **B13$_{[4+6]}$** as a pale yellow solid (356 mg, 0.20 mmol, 41%). *NB.* 300 mL was removed to investigate large scale crystallisation of the bridged cage catenane **B13$_{[12+8]}$** and remaining 200 mL used in the hexane solvent swap to isolate the [4+6] cage.

IR (ν_{max}/cm^{-1}) 3401 (br), 2963, 2905, 1679, 1627, 1489, 1463, 1410, 1377, 1313, 1286, 1261, 1209, 1156, 1096, 1040, 876, 798; **^{1}H NMR** (500 MHz, $CDCl_3$) δ_H 8.69 (s, 12H), 7.44 (s, 12H), 4.91 (s, 24H), 3.75 (s, 36H), 2.39 (s, 36H); **HRMS** (ES+) calc. for [4+6] cage $C_{108}H_{120}N_{12}O_{12}$ 1777.9180, found $[M+2H]^{2+}$ 889.9667 and $[M+3H]^{3+}$ 593.6484.

B15$_{[4+6]}$

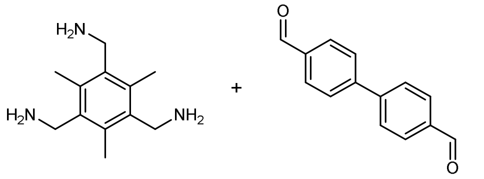

Synthesised according to the general procedure using (2,4,6-trimethylbenzene-1,3,5-triyl)trimethanamine **B** (125 mg, 0.60 mmol, 5.0 eq.) and 4,4'-biphenyldicarboxaldehyde **15** (152 mg, 0.72 mmol, 6.0 eq.) in $CHCl_3$ (130 mL) for 3 days to afford **B15** as a cream solid (101 mg, 0.053 mmol, 44%).

IR (ν_{max}/cm^{-1}) 3397 (br), 2966, 2925, 2872, 1699, 1637, 1606, 1575, 1495, 1447, 1372, 1315, 1259, 1216, 1097, 1024, 815; **^{1}H NMR** (400 MHz, $CDCl_3$) δ_H 8.36 (s, 12H), 7.81 (d, *J* = 8.3 Hz, 24H), 7.61 (d, *J* = 8.3 Hz, 24H), 4.96 (s, 24H), 2.44 (s, 36H); **HRMS** (ES+) calc. for [4+6] cage $C_{132}H_{120}N_{12}$ 1873.9791, found $[M+2H]^{2+}$ 938.0038, $[M+3H]^{3+}$ 625.6723.

B18[4+6]

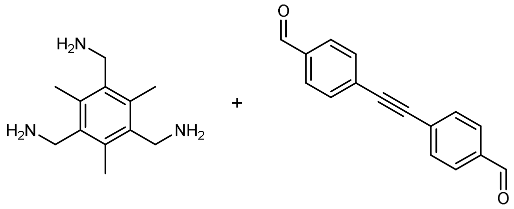

Data from the high-throughput screen: **IR** (ν_{max}/cm^{-1}) 3405 (br), 2965, 1639, 1602, 1261, 1094, 1023, 868, 802; **^{1}H NMR** (400 MHz, $CDCl_3$) δ_H 8.29 (s, 12H), 7.72 (d, *J* = 8.3 Hz, 24H), 7.53 (d, *J* = 8.2 Hz, 24H), 4.95 (s, 24H), 2.41 (s, 36H); **HRMS** (ES+) calc. for [4+6] cage $C_{144}H_{120}N_{12}$ 2017.9791, found $[M+2H]^{2+}$ 1009.4926, $[M+3H]^{3+}$ 673.6665 and $[M+4H]^{4+}$ 505.2523.

B24[4+4]

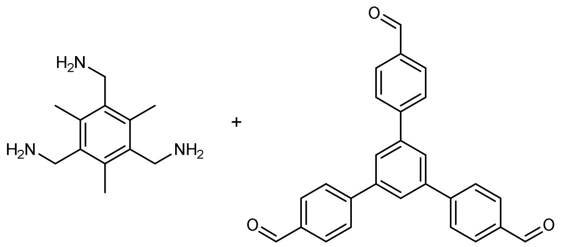

Synthesised according to the general scale-up procedure using (2,4,6-trimethylbenzene-1,3,5-triyl)trimethanamine **B** (250 mg, 1.21 mmol, 5.0 eq.) and 5'-(4-formylphenyl)-[1,1':3',1''-terphenyl]-4,4''-dicarbaldehyde **24** (375 mg, 0.96 mmol, 4.0 eq.) in DCM (260 mL) for 4 days to afford **B24** as a pale brown solid (113 mg mass recovery). Isolated product proved to be partially insoluble suggesting some decomposition of cage product had occurred, and ^{1}H NMR spectra indicates residual triamine **B** present.

IR (ν_{max}/cm^{-1}) 2961, 2904, 1637, 1607, 1410, 1262, 1097, 1025, 865, 797; **^{1}H NMR** (400 MHz, $CDCl_3$) δ_H 8.42 (s, 12H), 7.76 (d, *J* = 8.4 Hz, 24H), 7.64 (s, 12H), 7.56 (d, *J* = 8.3 Hz, 24H), 4.92 (s, 24H), 2.54 (s, 36H); **HRMS** (ES+) calc. for [4+4] cage $C_{156}H_{132}N_{12}$ 2174.0730, found $[M+2H]^{2+}$ 1088.0570, $[M+3H]^{3+}$ 725.7087 and $[M+4H]^{4+}$ 544.5330.

B25$_{[4+4]}$

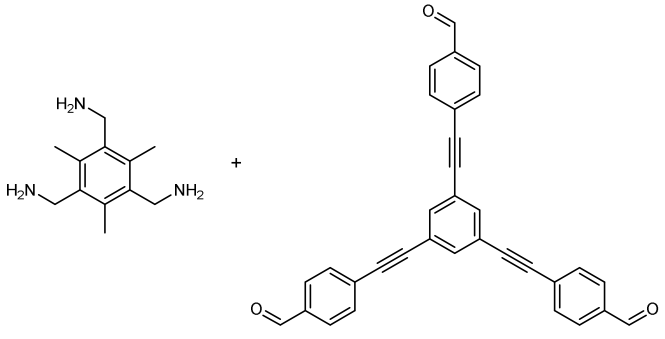

Synthesised according to the general scale-up procedure using (2,4,6-trimethylbenzene-1,3,5-triyl)trimethanamine **B** (33 mg, 0.16 mmol, 5.0 eq.) and 4,4',4''-(benzene-1,3,5-triyltris(ethyne-2,1-diyl))tribenzaldehyde **25** (60 mg, 0.13 mmol, 4.0 eq.) in DCM (35 mL) for 3 days. Complete sample used in recrystallisation screen without isolation - cage analysed directly from solution using a small aliquot.

IR (ν_{max}/cm^{-1}) 2961, 1701, 1639, 1606, 1582, 1415, 1303, 1259, 1097, 1019, 879, 805; **^{1}H NMR** (400 MHz, CDCl$_3$) δ_H 8.28 (s, 12H), 7.68 (d, *J* = 7.2 Hz, 24H), 7.60 (s, 12H), 7.46 (d, *J* = 8.1 Hz, 24H), 4.95 (s, 24H), 2.48 (s, 36H); **HRMS** (ES+) calc. for [4+4] cage $C_{180}H_{132}N_{12}$ 2462.0730, found $[M+2H]^{2+}$ 1232.0566, $[M+3H]^{3+}$ 821.7099 and $[M+4H]^{4+}$ 616.5368.

B26$_{[4+4]}$

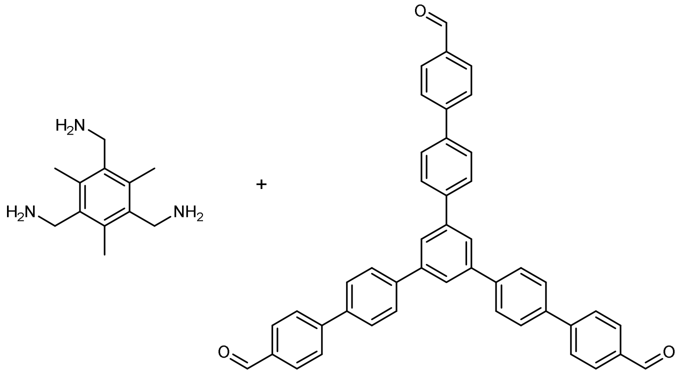

Synthesised according to the general scale-up procedure using (2,4,6-trimethylbenzene-1,3,5-triyl)trimethanamine **B** (42 mg, 0.20 mmol, 5.0 eq.) and 5''-(4'-formyl-[1,1'-biphenyl]-4-yl)-[1,1':4',1'':3'',1''':4''',1''''-quinquephenyl]-4,4''''-dicarbaldehyde **26** (100 mg, 0.16 mmol, 4.0 eq.) in CHCl$_3$ (100 mL) for 4 days to afford **B26** as a colourless solid (27 mg mass recovery). Isolated product proved to be partially insoluble suggesting some decomposition of cage product had occurred.

IR (ν_{max}/cm^{-1}) 2964, 2904, 1639, 1606, 1499, 1447, 1415, 1262, 1097, 1024, 863, 799; **^{1}H NMR** (400 MHz, CDCl$_3$) δ_H 8.10 (s, 12H), 7.77 (t, *J* = 4.2 Hz, 36H), 7.73–7.61 (m, 72H), 5.08 (s, 24H),

2.39 (s, 36H); **HRMS** (ES+) calc. for [4+4] cage $C_{228}H_{180}N_{12}$ 3087.4519, found $[M+3H]^{3+}$ 1030.1734 and $[M+4H]^{4+}$ 772.8817.

C1$_{[2+3]}$

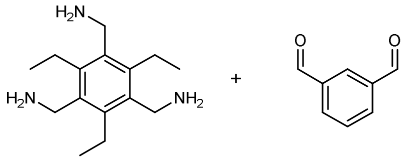

Synthesised according to the general scale-up procedure using (2,4,6-triethylbenzene-1,3,5-triyl)trimethanamine **C** (300 mg, 1.21 mmol, 3.0 eq.) and isophthalaldehyde **1** (161 mg, 1.21 mmol, 3.0 eq.) in DCM (260 mL) for 3 days to afford **C1** as a cream solid (239 mg, 0.30 mmol, 75%).

IR (ν_{max}/cm^{-1}) 2961, 2904, 1644, 1454, 1376, 1351, 1259, 1097, 1014, 865, 796; **^{1}H NMR** (400 MHz, $CDCl_3$) δ_H 8.16 (dd, *J* = 7.8, 1.5 Hz, 6H), 7.76 (s, 6H), 7.50 (t, *J* = 7.8 Hz, 3H), 6.99 (s, 3H), 5.09 (d, *J* = 1.8 Hz, 12H), 2.31 (q, *J* = 7.4 Hz, 12H), 1.23 (t, *J* = 7.5 Hz, 18H); **HRMS** (ES+) calc. for [2+3] cage $C_{54}H_{60}N_6$ 792.4879, found $[M+H]^2$ 793.4970 and $[M+2H]^{2+}$ 397.2521.

C2$_{[2+3]}$

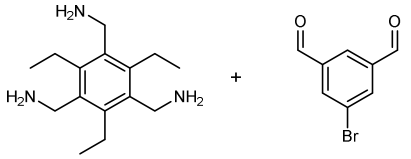

Synthesised according to the general procedure using (2,4,6-triethylbenzene-1,3,5-triyl)trimethanamine **C** (600 mg, 2.41 mmol, 3.0 eq.) and 5-bromoisophthalaldehyde **2** (514 mg, 2.41 mmol, 3.0 eq.) in DCM (520 mL) for 4 days to afford **C2** as pale yellow solid (608 mg, 0.59 mmol, 73%).

IR (ν_{max}/cm^{-1}) 3377 (br), 2965, 2926, 2870, 1683, 1644, 1594, 1567, 1491, 1455, 1381, 1354, 1259, 1097, 1017, 974, 864, 804; **^{1}H NMR** (400 MHz, $CDCl_3$) δ_H 8.28 (d, *J* = 1.0 Hz, 6H), 7.66 (s, 6H), 6.89 (s, 3H), 5.08 (d, *J* = 1.5 Hz, 12H), 2.26 (q, *J* = 7.3 Hz, 12H), 1.22 (t, *J* = 7.5 Hz, 18H); **^{13}C NMR** (101 MHz, $CDCl_3$) δ_C 156.3, 144.4, 138.4, 131.4, 131.1, 129.0, 124.4, 54.7, 23.7, 16.1; **HRMS** (ES+) calc. for [2+3] cage $C_{54}H_{57}N_6Br_3$ 1030.2167, found $[M+H]^+$ 1031.2353 and $[M+2H]^{2+}$ 516.1235.

C4$_{[2+3]}$

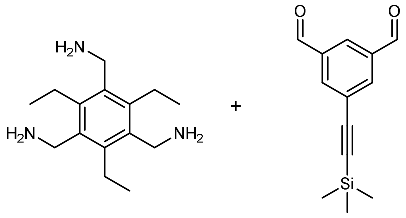

Data from the high-throughput screen: **IR** (ν_{max}/cm^{-1}) 3354 (br), 2966, 2155, 1640, 1588, 1454, 1376, 1260, 1097, 1020, 854, 801; **^{1}H NMR** (400 MHz, $CDCl_3$) δ_H 8.17 (d, *J* = 1.2 Hz, 6H), 7.79 (s, 6H), 7.13 (s, 3H), 5.04 (s, 12H), 2.30 (q, *J* = 7.3 Hz, 12H), 1.20 (t, *J* = 7.4 Hz, 18H); **HRMS** (ES+) calc. for [2+3] cage $C_{69}H_{84}N_6Si_3$ 1080.6065, found $[M+2H]^{2+}$ 541.3107.

C5$_{[2+3]}$

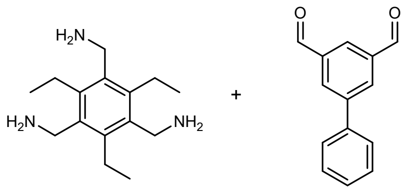

Data from the high-throughput screen: **IR** (ν_{max}/cm^{-1}) 3358 (br), 2966, 1640, 1591, 1447, 1376, 1313, 1263, 1154, 1094, 1041, 974, 801; **^{1}H NMR** (400 MHz, $CDCl_3$) δ_H 8.40 (s, 6H), 7.89 (s, 6H), 7.73 (d, *J* = 7.6 Hz, 6H), 7.43 (d, *J* = 7.2 Hz, 6H), 7.39–7.34 (m, 3H), 7.12 (s, 3H), 5.12 (s, 12H), 2.36 (q, *J* = 7.5 Hz, 12H), 1.25 (t, *J* = 6.4 Hz, 18H); **HRMS** (ES+) calc. for [2+3] cage $C_{72}H_{72}N_6$ 1020.5818, found $[M+H]^+$ 1021.5996 and $[M+2H]^{2+}$ 511.3091.

C6$_{[2+3]}$

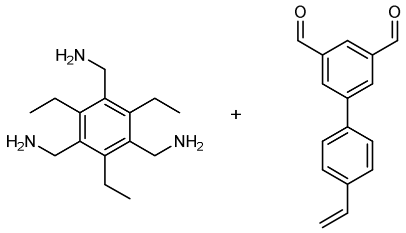

Data from the high-throughput screen: **IR** (ν_{max}/cm^{-1}) 3358 (br), 2966, 1640, 1598, 1450, 1380, 1316, 1267, 1094, 1020, 844, 801; **^{1}H NMR** (400 MHz, $CDCl_3$) δ_H 8.36 (d, *J* = 1.1 Hz, 6H), 7.94 (s, 6H), 7.70 (d, *J* = 8.3 Hz, 6H), 7.48 (d, *J* = 8.2 Hz, 6H), 7.26 (s, 3H), 6.76 (dd, *J* = 17.6, 10.9 Hz, 3H), 5.81 (d, *J* = 17.6 Hz, 3H), 5.29 (d, *J* = 11.1 Hz, 3H), 5.10 (s, 12H), 2.39 (q, *J* = 7.3 Hz, 12H), 1.25 (t, *J* = 7.4 Hz, 18H); **HRMS** (ES+) calc. for [2+3] cage $C_{78}H_{78}N_6$ 1098.6288, found $[M+2H]^{2+}$ 550.3345.

C7$_{[2+3]}$

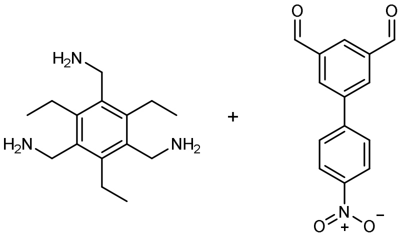

To a round bottomed flask was added (2,4,6-triethylbenzene-1,3,5-triyl)trimethanamine **C** (600 mg, 2.41 mmol, 2.0 eq.) and 4'-nitro-[1,1'-biphenyl]-3,5-dicarbaldehyde **7** (922 mg, 3.62 mmol, 3.0 eq.) before being dissolved in $CHCl_3$ (520 mL). The reaction was heated to reflux (65 °C) for 24 h, after which the reaction was cooled to room temperature, diluted with hexane (250 mL) and the $CHCl_3$ selectively removed *in vacuo*. The resulting precipitated product was filtered before being dried *in vacuo* to yield an off-white powder (448 mg mass recovery) — isolated product contained triphenylphosphine and hexane.

IR (ν_{max}/cm^{-1}) 3284 (br), 2966, 1644, 1598, 1563, 1517, 1457, 1337, 1260, 1108, 1045, 1013, 851, 798; **^{1}H NMR** (400 MHz, $CDCl_3$) δ_H 8.34–8.28 (m, 12H), 8.05 (s, 6H), 7.86 (d, J = 8.7 Hz, 6H), 7.58 (s, 3H), 5.08 (s, 12H), 2.41 (m, 12H), 1.25 (s, 18H); **HRMS** (ES+) calc. for [2+3] cage $C_{72}H_{69}N_9O_6$ 1155.5371, found $[M+2H]^{2+}$ 578.7872.

C8$_{[2+3]}$

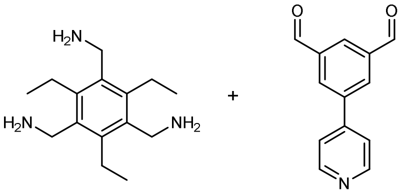

To a round bottomed flask was added (2,4,6-triethylbenzene-1,3,5-triyl)trimethanamine **C** (600 mg, 2.41 mmol, 2.0 eq.) and 5-(pyridin-4-yl)isophthalaldehyde **8** (764 mg, 3.62 mmol, 3.0 eq.) before being dissolved in $CHCl_3$ (520 mL). The reaction was heated to reflux (65 °C) for 24 h, after which the reaction was cooled to room temperature, diluted with hexane (250 mL) and the $CHCl_3$ selectively removed *in vacuo*. The resulting precipitated product was filtered before being dried *in vacuo* to yield an off-white powder (390 mg, 15%).

IR (ν_{max}/cm^{-1}) 3357 (br), 2970, 1640, 1598, 1563, 1450, 1379, 1263, 1094, 1041, 826; **^{1}H NMR** (400 MHz, $CDCl_3$) δ_H 8.67 (dd, *J* = 4.7, 1.4 Hz, 6H), 8.33 (s, 6H), 8.04 (s, 6H), 7.63 (dd, *J* = 4.7, 1.5 Hz, 6H), 7.54 (s, 3H), 5.08 (s, 12H), 2.40 (q, *J* = 7.6 Hz, 12H), 1.24–1.21 (m, 18H); **HRMS** (ES+) calc. for [2+3] cage $C_{69}H_{69}N_9$ 1023.5676, found $[M+H]^+$ 1024.5920 and $[M+2H]^{2+}$ 512.8024.

C9$_{[2+3]}$

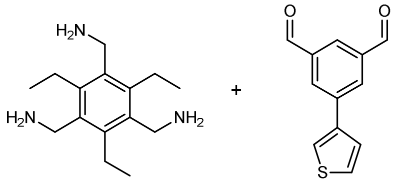

To a round bottomed flask was added (2,4,6-triethylbenzene-1,3,5-triyl)trimethanamine **C** (600 mg, 2.41 mmol, 2.0 eq.) and 5-(thiophen-3-yl)isophthalaldehyde **9** (781 mg, 3.62 mmol, 3.0 eq.) before being dissolved in $CHCl_3$ (520 mL). The reaction was heated to reflux (65 °C) for 24 h, after which the reaction was cooled to room temperature, diluted with hexane (250 mL) and the $CHCl_3$ selectively removed *in vacuo*. The resulting precipitated product was filtered before being dried *in vacuo* to yield a dark yellow powder (788 mg, 31%).

IR (ν_{max}/cm^{-1}) 3379 (br), 2963, 1701, 1640, 1595, 1454, 1355, 1260, 1101, 1013, 868, 798; **^{1}H NMR** (400 MHz, $CDCl_3$) δ_H 8.33 (d, *J* = 1.4 Hz, 6H), 7.94 (s, 6H), 7.64 (dd, *J* = 2.9, 1.3 Hz, 3H), 7.53 (dd, *J* = 5.1, 1.3 Hz, 3H), 7.39 (dd, *J* = 5.0, 2.9 Hz, 3H), 7.24 (s, 3H), 5.08 (s, 12H), 2.38 (q, *J* = 7.5 Hz, 12H), 1.25–1.22 (m, 18H); **HRMS** (ES+) calc. for [2+3] cage $C_{66}H_{66}N_6S_3$ 1038.4511, found $[M+2H]^{2+}$ 520.2448.

C11$_{[4+6]}$

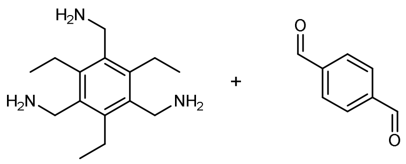

Synthesised according to the general procedure using (2,4,6-triethylbenzene-1,3,5-triyl)trimethanamine **C** (300 mg, 1.20 mmol, 5.0 eq.) and terephthalaldehyde **11** (194 mg, 1.44 mmol, 6.0 eq.) in DCM (260 mL) for 4 days to afford **C11** as yellow solid (146 mg, 0.092 mmol, 38%).

IR (ν_{max}/cm^{-1}) 2961, 2904, 1637, 1452, 1410, 1376, 1264, 1097, 1024, 861, 799; **^{1}H NMR** (400 MHz, $CDCl_3$) δ_H 8.40–8.18 (m, 12H), 7.78–7.65 (m, 24H), 4.94 (br s, 24H), 2.73 (q, *J* = 7.3 Hz, 24H), 1.26 (t, *J* = 7.3 Hz, 36H), *NB.* Multiple singlets apparent for the imine and aromatic protons at 8.40-8.18 and 7.78-7.65 ppm respectively, possibly due to slightly different environments within the cage molecule; **^{13}C NMR** (101 MHz, $CDCl_3$) δ_C 160.3, 144.0, 138.3, 132.7, 128.5, 58.0, 23.2, 15.7; **HRMS** (ES+) calc. for [4+6] cage $C_{108}H_{120}N_{12}$ 1585.9791, found $[M+2H]^{2+}$ 793.9984 and $[M+3H]^{3+}$ 529.6687.

C13[4+6]

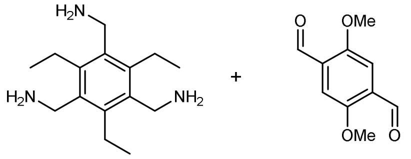

Synthesised according to the general procedure using (2,4,6-triethylbenzene-1,3,5-triyl)trimethanamine **C** (150 mg, 0.60 mmol, 5.0 eq.) and 2,5-dimethoxybenzene-1,4-dicarboxaldehyde **13** (140 mg, 0.72 mmol, 6.0 eq.) in DCM (130 mL) for 3 days to afford **C13** as a pale yellow solid (140 mg, 0.072 mmol, 60%).

IR (ν_{max}/cm^{-1}) 3391 (br), 2963, 1683, 1630, 1489, 1464, 1411, 1375, 1287, 1262, 1213, 1102, 1041, 799; **^{1}H NMR** (400 MHz, $CDCl_3$) δ_H 8.67 (s, 12H), 7.44 (s, 12H), 4.93 (s, 24H), 3.74 (s, 36H), 2.77 (q, *J* = 7.6 Hz, 24H), 1.26 (t, *J* = 7.3 Hz, 36H); **^{13}C NMR** (101 MHz, $CDCl_3$) δ_C 156.9, 153.1, 143.9, 133.0, 127.8, 109.9, 58.1, 56.2, 23.3, 15.7; **HRMS** (ES+) calc. for [4+6] cage $C_{120}H_{144}N_{12}O_{12}$.1946.1059, found $[M+2H]^{2+}$ 974.0741 and $[M+3H]^{3+}$ 649.7200.

C14[2+3]

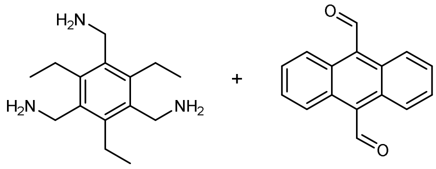

Synthesised according to the general procedure using (2,4,6-triethylbenzene-1,3,5-triyl)trimethanamine **C** (150 mg, 0.60 mmol, 3.0 eq.) and anthracene-9,10-dicarboxaldehyde **14** (141 mg, 0.60 mmol, 3.0 eq.) in DCM (130 mL) for 3 days to afford **C14** as a bright yellow solid (136 mg mass recovery). *NB*. Cage present but contains impurities and broad baseline peaks.

IR (ν_{max}/cm^{-1}) 3368 (br), 2966, 1675, 1640, 1454, 1256, 1309, 1101, 1016, 794; **^{1}H NMR** (400 MHz, $CDCl_3$) δ_H 8.65 (s, 6H), 8.16 (dd, *J* = 6.9, 3.1 Hz, 6H), 7.64 (dd, *J* = 6.5, 3.6 Hz, 6H), 7.05 (dd, *J* = 6.9, 3.3 Hz, 6H), 6.66 (ddd, *J* = 6.7, 3.2, 1.5 Hz, 6H), 5.25 (s, 12H), 3.02 (q, *J* = 7.5 Hz, 12H), 1.26 (t, *J* = 9.0 Hz, 18H); **HRMS** (ES+) calc. for [2+3] cage $C_{78}H_{72}N_6$ 1092.5818, found $[M+H]^+$ 1093.6088.

C15$_{[4+6]}$

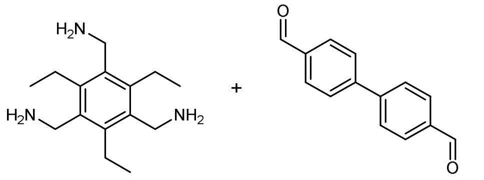

Synthesised according to the general procedure using (2,4,6-triethylbenzene-1,3,5-triyl)trimethanamine **C** (300 mg, 1.21 mmol, 5.0 eq.) and 4,4'-biphenyldicarboxaldehyde **15** (304 mg, 1.45 mmol, 6.0 eq.) in DCM (260 mL) for 4 days to afford **C15** as a cream solid (363 mg, 0.178 mmol, 74%).

IR (ν_{max}/cm^{-1}) 3394 (br), 2966, 1696, 1642, 1604, 1497, 1450, 1377, 1320, 1261, 1098, 1026, 811; **^{1}H NMR** (400 MHz, $CDCl_3$) δ_H 8.35 (s, 12H), 7.80 (d, *J* = 8.3 Hz, 24H), 7.60 (d, *J* = 8.3 Hz, 24H), 4.98 (s, 24H), 2.82 (q, *J* = 7.4 Hz, 24H), 1.31 (t, *J* = 7.5 Hz, 36H); **HRMS** (ES+) calc. for [4+6] cage $C_{144}H_{144}N_{12}$ 2042.1669, found $[M+2H]^{2+}$ 1022.5923.

C18$_{[4+6]}$

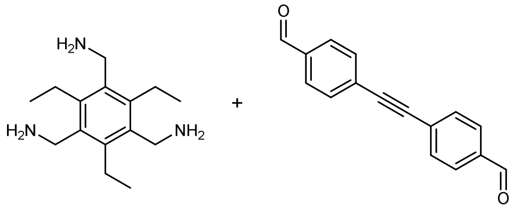

Data from the high-throughput screen: **IR** (ν_{max}/cm^{-1}) 3389 (br), 2966, 1637, 1602, 1263, 1097, 1019, 797; **^{1}H NMR** (400 MHz, $CDCl_3$) δ_H 8.41–8.16 (m, 12H), 7.71 (d, *J* = 8.4 Hz, 12H), 7.57–7.41 (m, 36H), 5.10–4.91 (m, 24H), 3.59 (q, *J* = 7.2 H, 24Hz), 1.47 (t, *J* = 6.5 Hz, 36H); **HRMS** (ES+) calc. for [4+6] cage $C_{156}H_{144}N_{12}$ 2186.1669, found $[M+3H]^{3+}$ 729.7294 and $[M+4H]^{4+}$ 547.5494.

C20$_{[2+3]}$

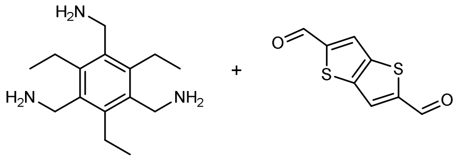

Data from the high-throughput screen: **IR** (ν_{max}/cm^{-1}) 3361 (br), 2963, 1620, 1496, 1450, 1348, 1260, 1154, 1084, 1045, 974, 805; **^{1}H NMR** (400 MHz, $CDCl_3$) δ_H 8.23–7.94 (m, 6H), 7.33 (d, *J* = 3.3 Hz, 3H), 7.23 (s, 3H), 5.03–4.68 (m, 12H), 2.84–2.66 (m, 12H), 1.24–1.17 (m, 18H). *NB.* Multiple singlets apparent for the imine at 8.23–7.94, possibly due to slightly different environments within the cage molecule.

C21

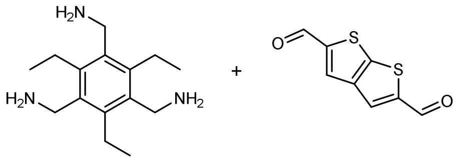

C21$_{[2+3]}$

Data from high-throughput screen: **IR** (ν_{max}/cm^{-1}) 3358 (br), 2966, 1627, 1517, 1450, 1376, 1309, 1263, 1097, 1020, 798; **^{1}H NMR** (400 MHz, $CDCl_3$) δ_H 7.81–7.62 (m, 6H), 7.47 (s, 3H), 7.02–6.96 (m, 3H), 5.05–4.94 (m, 12H), 2.51 (q, *J* = 7.7 Hz, 12H), 1.13 (t, *J* = 7.3 Hz, 18H) *NB.* Multiple singlets apparent for the imine and aromatic peaks, possibly due to slightly different environments within the cage molecule; **HRMS** (ES+) calc. for [2+3] cage $C_{54}H_{54}N_6S_6$ 978.2734, found $[M+H]^+$ 979.2783 and $[M+2H]^{2+}$ 490.1445.

C21$_{[2+3]/[4+6]}$

Synthesised according to the general procedure using (2,4,6-triethylbenzene-1,3,5-triyl)trimethanamine **C** (150 mg, 0.60 mmol, 3.0 eq.) and thieno[2,3-*b*]thiophene-2,5-dicarboxaldehyde **21** (118 mg, 0.60 mmol, 3.0 eq.) in DCM (130 mL) for 3 days to afford a mixture of **C21$_{[2+3]}$** and **C21$_{[4+6]}$** as a cream solid (127 mg mass recovery).

Both the ^{1}H NMR and HRMS indicate formation of both a [2+3] and [4+6] cage — see **Supplementary Figures 29 and 30**.

C23$_{[4+4]}$

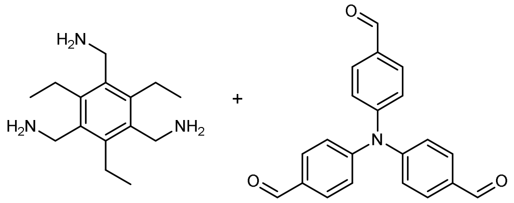

Synthesised according to the general procedure using (2,4,6-triethylbenzene-1,3,5-triyl)trimethanamine **C** (300 mg, 1.21 mmol, 5.0 eq.) and tris(4-formylphenyl)amine **23** (316 mg, 0.96 mmol, 4.0 eq.) in DCM (260 mL) for 3 days to afford **C23** as a yellow solid (266 mg, 0.127 mmol, 53%).

IR (ν_{max}/cm^{-1}) 3397 (br), 2966, 1642, 1595, 1504, 1320, 1261, 1174, 1102, 1014, 826; **^{1}H NMR** (400 MHz, $CDCl_3$) δ_H 8.15 (s, 12H), 7.52 (d, *J* = 8.6 Hz, 24H), 6.94 (d, *J* = 8.6 Hz, 24H), 4.85 (s, 24H), 2.79 (q, *J* = 7.5 Hz, 24H), 1.29 (t, *J* = 7.5 Hz, 36H); **HRMS** (ES+) calc. for [4+4] cage $C_{144}H_{144}N_{16}$ 2098.1791, found $[M+2H]^{2+}$ 1050.0994, $[M+3H]^{3+}$ 700.4036 and $[M+4H]^{4+}$ 525.5549.

C24$_{[4+4]}$

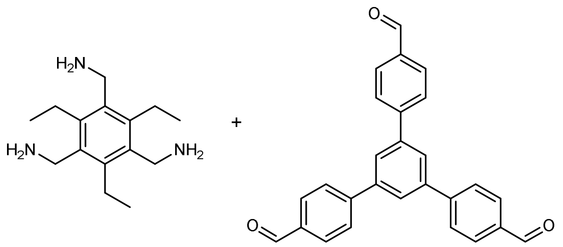

Synthesised according to the general procedure using (2,4,6-triethylbenzene-1,3,5-triyl)trimethanamine **C** (300 mg, 1.21 mmol, 5.0 eq.) and 5'-(4-formylphenyl)-[1,1':3',1''-terphenyl]-4,4''-dicarbaldehyde **24** (375 mg, 0.96 mmol, 4.0 eq.) in DCM (260 mL) for 1 day to afford **C24** as a pale brown solid (439 mg mass recovery). Isolated product proved to be partially insoluble suggesting some decomposition of cage product had occurred, and ^{1}H NMR spectra indicates residual hexane present.

IR (ν_{max}/cm^{-1}) 3394 (br), 2969, 1700, 1639, 1605, 1377, 1320, 1261, 1109, 1018, 797; **^{1}H NMR** (400 MHz, $CDCl_3$) δ_H 8.50–8.08 (m, 12H), 7.90–7.46 (m, 60H), 5.19–4.84 (m, 24H), 3.08–2.70 (m, 24H), 1.42–1.28 (m, 36H) *NB.* Multiple singlets apparent for the imine protons at 8.40-8.18, alongside broad peaks and multiplets, possibly due to slightly different environments within the cage molecule; **HRMS** (ES+) calc. for [4+4] cage $C_{168}H_{156}N_{12}$ 2342.2608, found $[M+3H]^{3+}$ 781.7660 and $[M+4H]^{4+}$ 586.8278.

C25$_{[4+4]}$

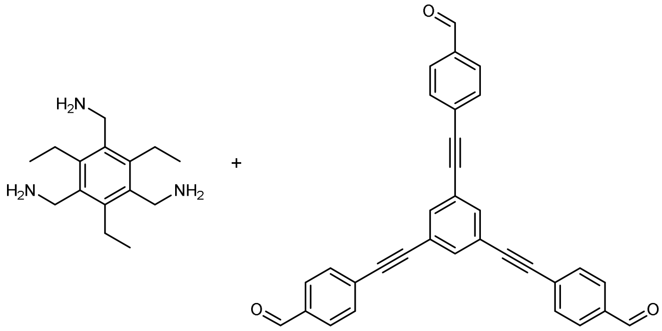

Synthesised according to the general procedure using (2,4,6-triethylbenzene-1,3,5-triyl)trimethanamine **C** (40 mg, 0.16 mmol, 5.0 eq.) and 4,4',4''-(benzene-1,3,5-triyltris(ethyne-2,1-diyl))tribenzaldehyde **25** (60 mg, 0.13 mmol, 4.0 eq.) in DCM (35 mL) for 1 day to afford **C25** as a pale yellow solid (23 mg mass recovery). Isolated product proved to be partially insoluble suggesting some decomposition of cage product had occurred, and ^{1}H NMR spectra indicates residual hexane present.

IR (ν_{max}/cm^{-1}) 3390, 2962, 1704, 1639, 1602, 1580, 1453, 1413, 1374, 1305, 1261, 1102, 1018, 877, 801; **^{1}H NMR** (400 MHz, $CDCl_3$) δ_H 8.21 (s, 12H), 7.65 (d, *J* = 8.4 Hz, 24H), 7.59 (s, 12H), 7.46 (d, *J* = 8.3 Hz, 24H), 4.94 (s, 24H), 2.81 (q, *J* = 7.5 Hz, 24H), 1.32 (t, *J* = 7.3 Hz, 36H); **HRMS** (ES+) calc. for [4+4] cage $C_{192}H_{156}N_{12}$ 2631.2641, found $[M+3H]^{3+}$ 878.0979 and $[M+4H]^{4+}$ 658.8273.

C26$_{[4+4]}$

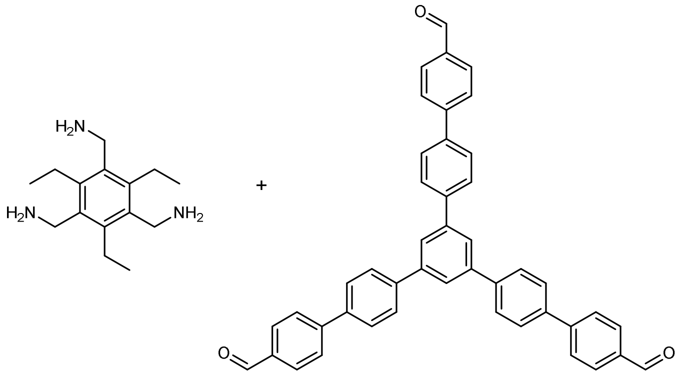

Synthesised according to the general procedure using (2,4,6-triethylbenzene-1,3,5-triyl)trimethanamine **C** (50 mg, 0.20 mmol, 5.0 eq.) and 5''-(4'-formyl-[1,1'-biphenyl]-4-yl)-[1,1':4',1'':3'',1''':4''',1''''-quinquephenyl]-4,4''''-dicarbaldehyde **26** (100 mg, 0.16 mmol, 4.0 eq.) in $CHCl_3$ (100 mL) for 4 days to afford **C26** as a colourless solid (25 mg mass recovery). Isolated product proved to be partially insoluble suggesting some decomposition of cage product had occurred, and ^{1}H NMR spectra indicates residual hexane present.

IR (ν_{max}/cm^{-1}) 3394 (br), 2962, 1642, 1606, 1493, 1450, 1413, 1261, 1105, 1018, 811; **^{1}H NMR** (400 MHz, $CDCl_3$) δ_H 7.97 (s, 12H), 7.78–7.62 (m, 108H), 5.10 (s, 24H), 2.63–2.53 (m, 24H), 1.35–1.30 (m, 36H); **HRMS** (ES+) calc. for [4+4] cage $C_{240}H_{204}N_{12}$ 3255.6397, found $[M+4H]^{4+}$ 814.9300.

Crystal structure refinement details for cages and bridged catenanes

	**B1**·0.82($C_4H_8O_2$)·0.18(CH_2Cl_2)·0.2(H_2O)	3(**B2**)·6(CH_2Cl_2)[a]	**B9**·0.75($CDCl_3$)·1.99(H_2O)
Crystallisation Solvent	CH_2Cl_2 EtOAc	CH_2Cl_2 Et_2O	$CDCl_3$ MeOH
Wavelength [Å]	Mo *Kα*	Mo *Kα*	Mo *Kα*
Collection Temperature	100 K	150 K	100 K
Formula	$C_{51.48}H_{55.33}Cl_{0.35}N_6O_{1.84}$	$C_{150}H_{147}Br_9Cl_{12}N_{18}$	$C_{60.76}H_{57.98}Cl_{2.26}D_{0.75}N_6O_{1.99}S_3$
Mr	799.98	3346.44	1081.83
Crystal Size (mm)	0.153 x 0.148 x 0.058	0.234 x 0.181 x 0.09	0.143 x 0.054 x 0.037
Crystal System	Triclinic	Hexagonal	Triclinic
Space Group	$P\bar{1}$	$P\bar{6}$	$P\bar{1}$
a [Å]	11.9249(4)	16.1857(6)	12.3943(9)
b [Å]	14.1652(5)		12.4852(9)
c [Å]	14.5526(5)	16.6104(7)	18.2566(12)
α [°]	73.9550(10)		94.0179(19)
β [°]	70.5990(10)		92.5322(19)
γ [°]	88.0330(13)		91.790(2)
V [Å^3]	2223.81(13)	3768.5(3)	2813.8(3)
Z	2	1	2
D_{calcd} [g cm^{-3}]	1.195	1.475	1.277
μ [mm^{-1}]	0.094	2.662	0.288
F(000)	854	1692	1135
2θ range [°]	2.998–58.268	2.906–52.730	2.238–46.512
Reflections collected	29925	43063	38510
Independent reflections, R_{int}	11975, 0.0463	5325, 0.0812	8075, 0.0558
Obs. Data [$I > 2\sigma$]	8720	4463	5769
Data / restraints / parameters	11975 / 4 / 587	5325 / 220 / 314	8075 / 97 / 749
Final R1 values ($I > 2\sigma(I)$)	0.0572	0.0820	0.0824
Final R1 values (all data)	0.0819	0.0988	0.1112
Final wR(F^2) values (all data)	0.1576	0.2369	0.2474
Goodness-of-fit on F^2	1.027	1.024	1.024
Largest difference peak and hole [e.A^{-3}]	0.348 / -0.389	1.655 / -1.004	1.146 / -1.072
Flack parameter	-	0.423(13)	-
CCDC	1827867	1827873	1827877

[a] The crystal structure was refined with the TWINLAW [010 100 00$\bar{1}$], BASF [0.340(5)].

	**B11**·4.5(C_6H_{14})·4.5(CH_2Cl_2)	**B11**·1.16(H_2O)[a]	**B13**$_{[8+12]}$·2($CHCl_3$)·2(C_4H_8O)·18.5(H_2O)
Crystallisation Solvent	CH_2Cl_2 Hexane	$CHCl_3$ MeCN	$CHCl_3$ THF
Wavelength [Å]	1.0332	Mo *Kα*	Mo *Kα*
Collection Temperature	100 K	100 K	100 K
Formula	$C_{127.50}H_{168}Cl_9N_{12}$	$C_{96}H_{98.32}N_{12}O_{1.16}$	$C_{226}H_{295}Cl_6N_{24}O_{44.5}$
Mr	2187.78	1438.74	4272.54
Crystal Size (mm)	0.17 x 0.08 x 0.07	0.19 x 0.10 x 1	0.25 x 0.25 x 0.10
Crystal System	Tetragonal	Trigonal	Triclinic
Space Group	$P4_1$	$P\bar{3}$	$P\bar{1}$
a [Å]	18.4998(8)	19.5104(10)	17.524(2)
b [Å]			19.681(3)
c [Å]	37.5158(18)	14.1156(8)	21.948(4)
α [°]			112.027(4)
β [°]			112.920(3)
γ [°]			95.170(4)
V [Å^3]	12839.5(13)	4653.3(5)	6210.9(16)
Z	4	2	1
D_{calcd} [g cm^{-3}]	1.132	1.027	1.142
μ [mm^{-1}]	1.299	0.062	0.141
F(000)	4680	1535	2277
2θ range [°]	3.568–70.272	2.410–41.622	2.252–43.452
Reflections collected	34493	28202	69169
Independent reflections, R_{int}	12128, 0.0693	3264, 0.0697	14703, 0.0506
Obs. Data [$I > 2\sigma$]	9363	2437	8881
Data / restraints / parameters	12128 / 871 / 973	3264 / 261 / 339	14703 / 1023 / 1398
Final R1 values ($I > 2\sigma(I)$)	0.0508	0.0889	0.1596
Final R1 values (all data)	0.0620	0.1167	0.2159
Final wR(F^2) values (all data)	0.1405	0.2718	0.4292
Goodness-of-fit on F^2	1.020	1.118	2.755
Largest difference peak and hole [e.A^{-3}]	0.251 / -0.205	0.428 / -0.264	1.116 / -0.530
CCDC	1827868	1827869	1827870

[a] The single crystal was mounted on a MiTeGen gripper and desolvated *in-situ* at 400 K under a dry N_2 gas flow. The single crystal was allowed to slowly cool to room temperature in air before being flash cooled to 100 K. At 100 K a full data set was recorded.

	**B15**·1.5(C_6H_{14})·5.5($CHCl_3$)·3(H_2O)	**B15**·2.25(C_6H_{14})·2.25(CH_2Cl_2)·2.25(H_2O)
Crystallisation Solvent	$CHCl_3$ Hexane	CH_2Cl_2 Hexane
Wavelength [Å]	0.7749	0.7749
Collection Temperature	100 K	100 K
Formula	$C_{146.50}H_{152.50}Cl_{16.50}N_{12}O_3$	$C_{147.75}H_{160.50}Cl_{4.50}N_{12}O_{2.25}$
Mr	2714.22	2299.90
Crystal Size (mm)	0.11 x 0.10 x 0.04	0.13 x 0.10 x 0.05
Crystal System	Trigonal	Triclinic
Space Group	$P\bar{3}$	$P\bar{1}$
a [Å]	22.9539(10)	18.8234(11)
b [Å]		20.8419(12)
c [Å]	16.4507(9)	22.4054(13)
α [°]		103.931(4)
β [°]		93.490(4)
γ [°]		114.824(3)
V [Å^3]	7506.3(8)	7611.2(8)
Z	2	2
D_{calcd} [g cm^{-3}]	1.201	1.004
μ [mm^{-1}]	0.444	0.167
F(000)	2840	9910
2θ range [°]	4.468–57.926	4.260–50.998
Reflections collected	75416	54729
Independent reflections, R_{int}	10239, 0.0541	21798, 0.0444
Obs. Data [$I > 2\sigma$]	7647	14359
Data / restraints / parameters	10239 / 32 / 577	21798 / 1463 / 1777
Final R1 values ($I > 2\sigma(I)$)	0.0882	0.1544
Final R1 values (all data)	0.1123	0.1986
Final wR(F^2) values (all data)	0.2760	0.4278
Goodness-of-fit on F^2	1.062	2.683
Largest difference peak and hole [e.A^{-3}]	1.610 / -1.343	0.844 / -0.583
CCDC	1827872	1827871

	**B23**·7.75(CH_2Cl_2)·4.5(C_6H_{14})·0.5(H_2O)	**B24**·13.35($C_4H_8O_2$)·11.75(CH_2Cl_2)	**B26**·23.25(C_4H_8O)·23.25($CHCl_3$)
Crystallisation Solvent	CH_2Cl_2 Hexane	CH_2Cl_2 EtOAc	$CHCl_3$ THF
Wavelength [Å]	0.6889	Mo *Kα*	Mo *Kα*
Collection Temperature	100 K	100 K	100 K
Formula	$C_{166.75}H_{198.50}Cl_{15.50}N_{16}O_{0.50}$	$C_{377.15}H_{394.3}Cl_{23.5}N_{24}O_{26.7}$	$C_{344.25}H_{389.25}Cl_{69.75}N_{12}O_{23.25}$
Mr	2984.38	6523.52	7539.54
Crystal Size (mm)	0.17 x 0.13 x 0.1	0.379 x 0.154 x 0.15	0.21 x 0.177 x 0.108
Crystal System	Triclinic	Monoclinic	Monoclinic
Space Group	$P\bar{1}$	*C*2/*c*	*C*2
a [Å]	15.989(4)	33.688(8)	42.199(13)
b [Å]	17.472(5)	33.545(8)	24.268(7)
c [Å]	31.697(8)	33.801(8)	40.276(13)
α [°]	94.735(3)		
β [°]	102.180(3)	102.385(5)	115.819(6)
γ [°]	109.008(3)		
V [Å^3]	8073(4)	37308(16)	37128(20)
Z	2	4	4
D_{calcd} [g cm^{-3}]	1.228	1.161	1.349
μ [mm^{-1}]	0.289	0.234	0.565
F(000)	3157	13753	15642
2θ range [°]	1.252–52.412	1.968–38.006	1.940–41.788
Reflections collected	126063	84793	64627
Independent reflections, R_{int}	35114, 0.0334	14782, 0.1399	30659, 0.0662
Obs. Data [$I > 2\sigma$]	26026	7347	18537
Data / restraints / parameters	35114 / 127 / 1638	14782 / 1387 / 1311	30659 / 1781 / 1470
Final R1 values ($I > 2\sigma(I)$)	0.0772	0.1667	0.0956
Final R1 values (all data)	0.0934	0.2327	0.1210
Final wR(F^2) values (all data)	0.2421	0.4077	0.2428
Goodness-of-fit on F^2	1.071	1.887	1.074
Largest difference peak and hole [e.A^{-3}]	1.205 / -1.051	0.416 / -0.337	0.266 / -0.257
CCDC	1827874	1827875	1827876

	**C1**·(C_8H_{10})·0.25(CH_2Cl_2)·0.25 (H_2O)	**C2**·3(CH_2Cl_2)	**C7**·2.66($CDCl_3$)·1.5(C_4H_8O)·1.6(H_2O)
Crystallisation Solvent	CH_2Cl_2 *m*-xylene	CH_2Cl_2 MeOH	$CDCl_3$ EtOAc
Wavelength [Å]	Mo *Kα*	Mo *Kα*	Mo *Kα*
Collection Temperature	100 K	150 K	150 K
Formula	$C_{51.48}H_{55.33}Cl_{0.35}N_6O_{1.84}$	$C_{57}H_{63}Br_3Cl_6N_6$	$C_{80.66}H_{86.86}Cl_{7.97}N_9O_{9.10}$
Mr	925.48	1284.56	1637.32
Crystal Size (mm)	0.22 x 0.20 x 0.17	0.40 x 0.26 x 0.24	0.30 x 0.29 x 0.19
Crystal System	Triclinic	Triclinic	Monoclinic
Space Group	$P\bar{1}$	$P\bar{1}$	*C*2/*c*
a [Å]	15.0024(6)	13.9893(7)	21.536(3)
b [Å]	15.2730(7)	15.0462(7)	15.147(2)
c [Å]	15.2779(6)	16.1794(8)	27.037(5)
α [°]	60.8167(16)	117.2705(11)	
β [°]	81.7670(16)	104.1249(13)	109.508(2)
γ [°]	76.8256(17)	93.9722(14)	
V [$Å^3$]	2973.7(17)	2870.0(2)	8313(2)
Z	2	2	4
D_{calcd} [g cm^{-3}]	1.034	1.486	1.308
μ [mm^{-1}]	0.082	2.431	0.332
F(000)	995	1308	3417
2θ range [°]	2.790–52.798	3.07–56.564	3.196–41.624
Reflections collected	33904	35446	17574
Independent reflections, R_{int}	12184, 0.0559	14245, 0.0451	4339, 0.0338
Obs. Data [$I > 2\sigma$]	7014	11466	3505
Data / restraints / parameters	12184 / 29 / 644	14245 / 43 / 692	4339 / 159 / 466
Final R1 values ($I > 2\sigma(I)$)	0.0977	0.0592	0.0958
Final R1 values (all data)	0.1586	0.0736	0.1078
Final wR(F^2) values (all data)	0.3292	0.1601	0.2882
Goodness-of-fit on F^2	1.047	1.064	1.054
Largest difference peak and hole [e.A^{-3}]	1.179 / -0.341	2.219 / -2.540	0.496 / -0.352
CCDC	1827878	1827881	1827886

	**C9**·4.67($CDCl_3$)·0.48(C_2H_3N)	**C13**·3.67(CH_2Cl_2)·6(CH_4O)·1.5(H_2O)	**C14**·2(C_4H_8O)
Crystallisation Solvent	$CDCl_3$ MeCN	CH_2Cl_2 MeOH	CH_2Cl_2 THF
Wavelength [Å]	Cu *Kα*	0.7749	Mo *Kα*
Collection Temperature	100 K	100 K	100 K
Formula	$C_{71.62}H_{67.43}Cl_{14.02}D_{4.68}N_{6.47}S_3$	$C_{249.67}H_{320.33}Cl_{7.33}N_{24}O_{31.5}$	$C_{86}H_{88}N_6O_2$
Mr	1621.50	4421.50	1237.62
Crystal Size (mm)	0.39 x 0.27 x 0.26	0.16 x 0.05 x 0.03	not measured[a]
Crystal System	Triclinic	Triclinic	Triclinic
Space Group	$P\bar{1}$	$P\bar{1}$	$P\bar{1}$
a [Å]	13.6116(5)	18.078(2)	13.2233(10)
b [Å]	15.8683(6)	18.632(2)	13.3938(10)
c [Å]	20.3799(8)	20.282(2)	19.8291(16)
α [°]	100.2965(16)	109.669(3)	75.682(3)
β [°]	98.7148(16)	95.102(3)	81.032(3)
γ [°]	113.3470(14)	104.266(3)	72.388(3)
V [$Å^3$]	3854.1(3)	6123.2(13)	3230.8(4)
Z	2	1	2
D_{calcd} [g cm^{-3}]	1.397	1.199	1.272
μ [mm^{-1}]	5.708	0.192	0.076
F(000)	1667	2363	1324
2θ range [°]	6.288–140.136	2.648–45.592	4.782–49.424
Reflections collected	84648	22476	48180
Independent reflections, R_{int}	14556, 0.0441	12457, 0.0446	11000, 0.597
Obs. Data [$I > 2\sigma$]	12964	7289	7997
Data / restraints / parameters	14556 / 47 / 956	12457 / 1261 / 1543	11000 / 51 / 907
Final R1 values ($I > 2\sigma(I)$)	0.0716	0.1465	0.0790
Final R1 values (all data)	0.0785	0.2106	0.1088
Final wR(F^2) values (all data)	0.1886	0.3798	0.2176
Goodness-of-fit on F^2	1.028	1.974	1.069
Largest difference peak and hole [e.A^{-3}]	1.530 / -1.282	0.953 / -0.716	0.739 / -0.393
CCDC	1827887	1827879	1827880

[a] the crystal fell of the mount during the data collection and the crystal size could not be measured.

	**C21Tri⁴Di⁶**·7.12(CH_2Cl_2)·5.12(C_2H_3N)·0.25(H_2O)	**C21Tri²Di³**·0.5(CH_2Cl_2)·0.25($C_4H_{10}O$)
Crystallisation Solvent	CH_2Cl_2 MeCN	CH_2Cl_2 Et_2O
Wavelength [Å]	Mo *Kα*	0.6889
Collection Temperature	100 K	100 K
Formula	$C_{125.38}H_{138.12}Cl_{14.25}N_{17.12}O_{0.25}S_{12}$	$C_{55.5}H_{57.5}ClN_6O_{0.25}S_6$
Mr	2778.78	1040.38
Crystal Size (mm)	not measured[a]	0.091 x 0.087 x 0.049
Crystal System	Monoclinic	Triclinic
Space Group	$P2_1/n$	$P\bar{1}$
a [Å]	18.776(9)	12.90010(10)
b [Å]	31.091(14)	12.99880(10)
c [Å]	26.235(11)	17.19660(10)
α [°]		87.0750(10)
β [°]	95.934(12)	68.5880(10)
γ [°]		82.3650(10)
V [Å³]	15233(12)	2660.79(4)
Z	4	2
D_{calcd} [g cm^{-3}]	1.212	1.299
μ [mm^{-1}]	0.470	0.342
F(000)	5786	1095
2θ range [°]	2.038–41.632	2.466–51.004
Reflections collected	100268	35616
Independent reflections, R_{int}	15945, 0.0599	10768, 0.0534
Obs. Data [$I > 2\sigma$]	10496	9080
Data / restraints / parameters	15945 / 1544 / 1421	10768 / 14 / 683
Final R1 values ($I > 2\sigma(I)$)	0.0827	0.0626
Final R1 values (all data)	0.1104	0.0693
Final wR(F^2) values (all data)	0.2385	0.1960
Goodness-of-fit on F^2	1.483	1.080
Largest difference peak and hole [e.A^{-3}]	0.566 / -0.556	1.259 / -0.389
CCDC	1827883	1827882

[a] the crystal fell of the mount during the data collection and the crystal size could not be measured.

	**C23**·6.5(CH_2Cl_2)·7.5(C_2H_3N)	**C26**·12.25($CHCl_3$)·12.25($C_4H_{10}O$)
Crystallisation Solvent	CH_2Cl_2 MeCN	$CHCl_3$ Et_2O
Wavelength [Å]	Mo *Kα*	Mo *Kα*
Collection Temperature	100 K	100
Formula	$C_{165.50}H_{179.50}Cl_{13}N_{23.50}$	$C_{301.25}H_{338.75}Cl_{36.75}N_{12}O_{12.25}$
Mr	2958.67	5626.36
Crystal Size (mm)	0.257 x 0.170 x 0.123	0.32 x 0.281 x 0.246
Crystal System	Monoclinic	Tetragonal
Space Group	*P*2/*c*	$I4_1/a$
a [Å]	26.591(2)	41.493(9)
b [Å]	16.3674(14)	
c [Å]	39.525(4)	20.413(4)
β [°]	98.7932(18)	
V [$Å^3$]	17000(3)	35.145(16)
Z	4	4
D_{calcd} [g cm^{-3}]	1.156	1.063
μ [mm^{-1}]	0.266	0.333
F(000)	6232	11812
2θ range [°]	1.550–39.564	1.962–37.694
Reflections collected	67739	49964
Independent reflections, R_{int}	15390, 0.698	6915, 0.0685
Obs. Data [$I > 2\sigma$]	8603	2985
Data / restraints / parameters	15390 / 1393 / 1524	6915 / 538 / 515
Final R1 values ($I > 2\sigma(I)$)	0.1126	0.1257
Final R1 values (all data)	0.1572	0.1894
Final wR(F^2) values (all data)	0.3121	0.3497
Goodness-of-fit on F^2	1.666	1.671
Largest difference peak and hole [e.A^{-3}]	0.619 / -0.586	0.273 / -0.282
CCDC	1827884	1827885

Crystal structure refinement notes:

B1·0.82($C_4H_8O_2$)·(CH_2Cl_2)·(H_2O)

The single crystal structure, **B1**·0.82($C_4H_8O_2$)·0.18(CH_2Cl_2)·0.2(H_2O), crystallised from a CH_2Cl_2/EtOAc solution in the triclinic space group $P\bar{1}$. For this phase, the asymmetric unit comprises one complete **B1** cage and disordered solvent molecules. The solvent was modelled as a mixture of EtOAc, CH_2Cl_2, and H_2O. In the structure, EtOAc and CH_2Cl_2 were disordered over one position and site occupancies for each solvent was determined using a free variable. The disordered CH_2Cl_2 in the structure was refined with C-Cl bond distance restraints (DFIX in SHELX). The occupancy of the disordered H_2O molecule was determined using a free variable. For this H_2O molecule, it was not possible to accurately determine H atom positions, H atoms were therefore placed in estimated positions (PLAT415_ALERT_2_B checkCIF alert). For a displacement ellipsoid plot of the asymmetric unit, see **Supplementary Fig. 7**.

3(**B2**)·6(CH_2Cl_2)

The single crystal structure, 3(**B2**)·6(CH_2Cl_2), crystallised from a CH_2Cl_2/Et_2O solution in the hexagonal space group $P\bar{6}$ and the structure was refined with the TWINLAW [010 100 00$\bar{1}$], BASF [0.340(5)]. For this structure, the asymmetric unit comprises three 1/6 cage fragments of three crystallographically distinct **B2** cage molecules. The X-ray data quality was poor and the **B2** cage molecules are disordered in the crystal structure (PLAT341_ALERT_3_B checkCIF alert). As a result, the structure was refined with a rigid bond restraint (RIGU in SHELX). In addition, due to solvent disorder, the CH_2Cl_2 molecules were refined with C-Cl bond distance restraints (DFIX in SHELX). For a displacement ellipsoid plot, see **Supplementary Fig. 8**.

B9·0.75($CDCl_3$)·1.99(H_2O)

The single crystal structure, **B9**·0.75($CDCl_3$)·1.99(H_2O), crystallised from a $CDCl_3$/MeOH solution in the triclinic space group $P\bar{1}$. For this phase, the asymmetric unit comprises one complete **B9** cage and disordered solvent molecules. Due to disorder, a suitable resolution limit of 0.9 Å was applied during refinement (THETM01_ALERT_3_B checkCIF alert). In the structure, S atoms of the three thiophene groups were disordered over two positions. C-S and C-C bond distance restraints were used to model the disordered parts (DFIX in SHELX). For the disordered parts, free variables were used to determine site occupancies and atoms that shared almost equivalent coordinates were refined with constrained displacement parameters (EADP in SHELX). The disordered, partially occupied, solvent was modelled as a mixture of $CDCl_3$ and H_2O. In was necessary to refine some of the disordered solvent with

bond distance restraints (DFIX, SADI and DANG in SHELX) and rigid bond restraints (RIGU in SHELX). For a displacement ellipsoid plot, see **Supplementary Fig. 9**.

B11·4.5(C_6H_{14})·4.5(CH_2Cl_2)

The single crystal structure, **B11**·4.5(C_6H_{14})·4.5(CH_2Cl_2), crystallised from a CH_2Cl_2/hexane solution in the tetragonal space group $P4_1$. For this phase, the asymmetric unit comprises one complete **B11** cage. Chirality in the structure arises from the cage molecules packing helically around screw axes, however, absolute configuration could not be meaningfully determined. Due to disorder, X-ray data quality was poor and solvent molecules could not be resolved in the large pores (PLAT602_ALERT_2_A and PLAT049_ALERT_1_B checkCIF alerts). Hence, the SQUEEZE routine in Platon was used during the final refinement cycles.[36,37] SQUEEZE found a 6133 Å^3 void with a disordered electron count of 1681 (e-). As a result, 18 CH_2Cl_2 and 18 hexane solvent molecules were tentatively added to the unit cell atom count (CHEMW03_ALERT_2_A ALERT, PLAT043_ALERT_1_A, and PLAT051_ALERT_1_A checkCIF alerts). During refinement a suitable resolution limit of 0.9 Å was applied (PLAT027_ALERT_3_A and THETM01_ALERT_3_B checkCIF alerts) and the **B11** cage molecule was refined with a rigid bond restraint (RIGU in SHELX). In addition, 1,2 and 1,3 bond distance restraints were used during refinement (DFIX, SADI, and DANG in SHELX). Due to disorder, and the limited resolution of the diffraction data, it was not possible to accurately determine H atom positions. H atoms were therefore placed in estimated positions and refined using the riding model. The estimated positions of the disordered H atoms are unlikely to be correct resulting in close intramolecular H-H contacts (PLAT412_ALERT_2_B checkCIF alert). For a displacement ellipsoid plot, see **Supplementary Fig. 10**.

B11·1.16(H_2O)

The single crystal that refined to, **B11**·1.16(H_2O), was initially crystallised from a $CHCl_3$/MeCN solvent mixture and then thermally desolvated at 400 K, under a dry N_2 gas flow. Prior to the data collection being recorded, the single crystal was sat in air, and during refinement, residual electron density, located in the pores, was modelled as disordered, partially occupied H_2O. There was no clear evidence of any notable solvent molecules in the structure, despite collecting the single crystal data at 100 K, and is indicative of the majority, if not all, of the solvent being removed from the pores during thermal desolvation of the pores (PLAT601_ALERT_2_A). Due to the limited resolution of the single crystal X-ray data, a resolution limit of 1.0 Å was applied during refinement (PLAT601_ALERT_2_A alert). For a displacement ellipsoid plot, see **Supplementary Fig. 11a**, and for a diagram of the crystal packing, see **Supplementary Fig. 11b**.

B13$_{[8+12]}$·$2(CHCl_3)\cdot2(C_4H_8O)\cdot18.5(H_2O)$

The single crystal structure, **B13$_{[8+12]}$**·$2(CHCl_3)\cdot2(C_4H_8O)\cdot18.5(H_2O)$, crystallised from a $CHCl_3$/THF solution in the triclinic space group $P\overline{1}$. The asymmetric unit for this phase comprises ½ of the interlocked covalently attached cage; the complete cage is related by inversion symmetry. Due to disorder, a suitable resolution limit of 0.95 Å was applied during refinement (THETM01_ALERT_3_B and PLAT340_ALERT_3_B checkCIF alerts) and the final refinement statistics were poor (PLAT082_ALERT_2_B, PLAT084_ALERT_3_B checkCIF alerts), in part, this was due to severely disordered solvent. Diffuse electron density, found in voids in the crystal structure, was modelled as a mixture of $CHCl_3$, THF, and tentatively assigned H_2O. In the crystal structure, the **B13$_{[8+12]}$** cage was disordered and refined with 1,2 and 1,3 bond distance restraints (DFIX, SADI and DANG in SHELX), and a group rigid bond restraint (RIGU in SHELX). In addition, six aromatic rings were refined with constrained geometries (AFIX 66 in SHELX). Two of these were used to model a dimethoxybenzene unit that was disordered over two positions. These dimethoxy benzene units were refined isotropically and, due to severe disorder, one of these dimethoxy benzene units was refined with constrained displacement parameters (EADP in SHELX). Due to the limited resolution of the diffraction data, it was not possible to determine H atom positions. H atoms were therefore placed in estimated positions and refined using the riding model. The estimated positions of the disordered H atoms are unlikely to be correct resulting in close intramolecular H-H contact between a disordered $-CH_3$ group and disordered aryl$-CH_2$ group (PLAT412_ALERT_2_B checkCIF alert). Solvent was extremely disordered in the crystal structure. Solvent molecules that could be located were refined with 1,2 and 1,3 bond distance restraints (DFIX and DANG in SHELX) and only the $CHCl_3$ molecules were refined anisotropically. As a result of disorder, occupancies of the solvent molecules are tentative and diffuse electron density was modelled as partially occupied H_2O molecules, which were refined without H atoms. For a displacement ellipsoid plot, see **Supplementary Fig. 12**.

B15·$1.5(C_6H_{14})\cdot5.5(CHCl_3)\cdot3(H_2O)$

The single crystal structure, **B15**·$1.5(C_6H_{14})\cdot5.5(CHCl_3)\cdot3(H_2O)$, crystallised from a $CHCl_3$/hexane solution in the trigonal space group $P\overline{3}$. The asymmetric unit for this phase comprises 1/3 of a **B15** cage, one complete $CHCl_3$ molecule, three 1/3 parts of ordered $CHCl_3$ molecules, one H_2O molecule, and one hexane molecule. The hexane molecule was refined with C-C bond distance restraints (DFIX in SHELX) and a rigid bond restraint (RIGU in SHELX). In the crystal structure, the **B15** cage is well resolved and no disorder was evident. For a displacement ellipsoid plot, see **Supplementary Fig. 13**.

B15·2.25(C_6H_{14})2.25(CH_2Cl_2)·2.25(H_2O)

The single crystal structure, **B15**·2.25(C_6H_{14})2.25(CH_2Cl_2), crystallised from a CH_2Cl_2/hexane solution in the triclinic space group $P\bar{1}$. The asymmetric unit for this phase comprises one complete **B15** cage and disordered solvent. Due to disorder, a suitable resolution limit of 0.9 Å was applied during refinement (PLAT027_ALERT_3_A and THETM01_ALERT_3_B checkCIF alert) and the final refinement statistics were poor (RFACG01_ALERT_3_B, RFACR01_ALERT_3_B, and PLAT084_ALERT_3_B checkCIF alerts). In this crystal structure the **B15** cage molecule is disordered (PLAT220_ALERT_2_B and PLAT340_ALERT_3_B checkCIF alerts). During refinement five of the six diphenyl vertex groups were modelled over two positions and two of these vertex groups were refined isotropically (PLAT201_ALERT_2_A checkCIF alert). In addition, a number of restraints and constraints were used during refinement, for the exact details see the supporting information CIF file. In summary, seventeen aromatic rings were refined with constrained geometries (AFIX 66), chemically unrealistic 1,2 and 1,3 bond distances were refined with bond distance restraints (DFIX, DANG, and SADI in SHELX), and two aromatic groups were refined with planarity restraints (FLAT in SHELX). Electron density in the large pores was disordered (PLAT223_ALERT_4_B checkCIF alert) and modelled as a mixture of hexane and CH_2Cl_2. Solvent that could not be resolved was tentatively modelled as partially occupied H_2O. It is likely that all solvent molecules could not be resolved in the structure (PLAT601_ALERT_2_B checkCIF alert). The disordered solvent was refined with 1,2 and 1,3 bond distance restraints (DFIX, DANG, and SADI in SHELX) and the partially occupied hexane molecules were refined isotropically. For a displacement ellipsoid plot, see **Supplementary Fig. 14**.

B23·7.75(CH_2Cl_2)·4.5(C_6H_{14})·0.5(H_2O)

The single crystal structure, **B23**·7.75(CH_2Cl_2)·4.5(C_6H_{14})·0.5(H_2O), crystallised from a CH_2Cl_2/hexane solution in the triclinic space group $P\bar{1}$. The asymmetric unit for this phase comprises one complete **B23** cage, disordered CH_2Cl_2 solvent molecules, and tentatively assigned, disordered, H_2O. Due to disorder, it was not possible model solvent molecules in the large interconnected voids in the crystal structure (PLAT602_ALERT_2_A and PLAT049_ALERT_1_B checkCIF alerts). Hence, the SQUEEZE routine in Platon was used during the final refinement cycles.[36,37] SQUEEZE found a 2618 Å^3 void with a disordered electron count of 796 (e-). As a result, 9 CH_2Cl_2 and 9 hexane solvent molecules were tentatively added to the unit cell atom count (CHEMW03_ALERT_2_A, PLAT043_ALERT_1_A, and PLAT051_ALERT_1_A checkCIF alerts). In the crystal structure, two aromatic rings were disordered over two positions. For the disordered parts, site occupancies were determined using free variables, and two of these disordered aromatic rings were refined with constrained geometries (AFIX 66 in SHELX) and rigid bond restraints (RIGU in SHELX). For a displacement ellipsoid plot, see **Supplementary Fig. 15**.

B24·13.35($C_4H_8O_2$)·11.75(CH_2Cl_2)

The single crystal structure, **B24**·13.35($C_4H_8O_2$)·11.75(CH_2Cl_2), crystallised from a CH_2Cl_2/EtOAc solution in the monoclinic space group *C*2/*c*. The asymmetric unit for this phase comprises two ½ cage fragments of two crystallographically distinct **B24** cages. The X-ray data quality was poor and severely disordered. Therefore, a resolution limit of 1.09 Å was applied during refinement (THETM01_ALERT_3_A, PLAT027_ALERT_3_A, and PLAT340_ALERT_3_B checkCIF alerts) and the cage was refined with a rigid bond restraint (RIGU in SHELX). During refinement, all the aromatic groups were refined with constrained geometries (AFIX 66 in SHELX), and the C-C bonds between these aromatics were restrained (DFIX in SHELX). Due to disorder, the final refinement statistics were poor (RFACG01_ALERT_3_B, RFACR01_ALERT_3_B, PLAT082_ALERT_2_B, and PLAT084_ALERT_3_B checkCIF alerts) and it was not possible to resolve all the solvent positions (PLAT602_ALERT_2_A and PLAT049_ALERT_1_B checkCIF alerts). Hence, it was necessary to use the SQUEEZE routine in Platon during the refinement cycles.[36,37] SQUEEZE found a 14792 Å^3 void with a disordered electron count of 4217 (e-). As a result, 47 $C_4H_8O_2$ and 47 CH_2Cl_2 solvent molecules were tentatively added to the unit cell atom count (CHEMW03_ALERT_2_A, PLAT043_ALERT_1_A, and PLAT051_ALERT_1_A checkCIF alerts). The one solvent molecule that could be modelled was refined isotropically with C-C and C-O bond distance restraints. For a displacement ellipsoid plot, see **Supplementary Fig. 16**.

B26·23.25(C_4H_8O)·23.25($CHCl_3$)

The single crystal structure, **B26**·23.25(C_4H_8O)·23.25($CHCl_3$), crystallised from a $CHCl_3$/THF solution in the monoclinic space group *C*2. The asymmetric unit for this phase comprises one complete **B26** cage. The X-ray data quality was poor and in the crystal structure the **B26** cage was severely disordered. Absolute configuration could not be meaningfully determined. During refinement a suitable resolution limit of 1.0 Å was applied (THETM01_ALERT_3_A checkCIF alerts) and the **B26** cage was refined with a rigid bond restraint (RIGU in SHELX). In addition, all the aromatic groups were refined with constrained geometries (AFIX 66 in SHELX) and the C-C bonds between these aromatic groups were restrained during refinement (DFIX in SHELX). It was also necessary to restrain 1,3 bond distances for the disordered groups (DANG and SADI in SHELX). In the crystal structure **B26** is shaped like a tetrapod. Three biphenyl groups; C49 > C59; C82 > C91; and C127 > C136; that make up ¼ of the tetrapod were severely disordered. These biphenyl groups were refined isotropically (PLAT201_ALERT_2_A checkCIF alert) with constrained displacement parameter (EADP in SHELX). Five additional aromatic groups were severely disordered, this resulted in differences between the displacement parameters of neighbouring C atoms (PLAT241_ALERT_2_B and PLAT242_ALERT_2_B checkCIF alert). Due to disorder, solvent molecules could not be resolved in the large pores (PLAT602_ALERT_2_A and PLAT049_ALERT_1_B checkCIF alerts). Hence, the SQUEEZE routine in Platon was used during the final refinement cycles.[36,37]

SQUEEZE found a 23138 Å^3 void with a disordered electron count of 8162 (e-). As a result, 93 $CHCl_3$ and 93 THF solvent molecules were tentatively added to the unit cell atom count (CHEMW03_ALERT_2_A, PLAT043_ALERT_1_A, and PLAT051_ALERT_1_A checkCIF alerts). In the structure, it was not possible to accurately determine H atom positions. H atoms were therefore placed in estimated positions and refined using the riding model. The estimated positions of the disordered H atoms are unlikely to be correct resulting in close H-H contacts (PLAT412_ALERT_2_A, PLAT412_ALERT_2_B, and PLAT410_ALERT_2_B checkCIF alerts). For a displacement ellipsoid plot, see **Supplementary Fig. 17**.

C1·(C_8H_{10})·0.25(CH_2Cl_2)·0.25 (H_2O)

The single crystal structure, **C1**·(C_8H_{10})·0.25(CH_2Cl_2)·0.25(H_2O), crystallised from a CH_2Cl_2/*m*-xylene solution in the triclinic space group $P\overline{1}$. The asymmetric unit for this phase comprises one complete **C1** cage and disordered solvent molecules. In the crystal structure 1-D channels are full of disordered solvent that was modelled as partially occupied *m*-xylene. These *m*-xylene molecules were refined isotropically with constrained geometries (AFIX 66 in SHELX), bond distance restraints (DFIX in SHELX), and planarity restraints (FLAT in SHELX). It was not possible to accurately resolve all disorder around the *m*-xylene molecules resulting in large unassigned q-peaks. Due to disorder, the CH_2Cl_2 molecule was refined with C-Cl bond distance restraints (DFIX in SHELX) and a rigid bond restraint (RIGU in SHELX). For a displacement ellipsoid plot, see **Supplementary Fig. 18**.

C2·3(CH_2Cl_2)

The single crystal structure, **C2**·3(CH_2Cl_2), was crystallised from a CH_2Cl_2/MeOH solution in the triclinic space group $P\overline{1}$. The asymmetric unit for this phase comprises one complete **C2** molecule, and four CH_2Cl_2 molecules. In the structure, one CH_2Cl_2 is disordered over two positions. This molecule was refined with bond distance restraints (SADI in SHELX) and a rigid bond restraint (RIGU in SHELX). In addition, two CH_2Cl_2 molecule were refined with C-Cl bond distance restraints (DFIX in SHELX) and rigid bond restraints (RIGU in SHELX). For a displacement ellipsoid plot, see **Supplementary Fig. 19**.

C7·2.49($CDCl_3$)·1.33($C_4H_8O_2$)·1.6(H_2O)

The single crystal structure, **C7**·2.49($CHCl_3$)·1.33($C_4H_8O_2$)·1.6(H_2O), crystallised from a $CDCl_3$/EtOAc solution in the monoclinic space group *C*2/*c*. The asymmetric unit for this phase comprises ½ a **C7** cage molecule and disordered solvent molecules. Due to disorder, a suitable resolution limit of resolution limit of 1.0 Å was applied during refinement (CHEMW03_ALERT_2_A, THETM01_ALERT_3_A, and PLAT027_ALERT_3_A checkCIF alerts).

In the structure one of the nitrophenyl groups was disordered over two positions, the disordered groups were refined with constrained geometries (AFIX 66 in SHELX), bond distance restraints (DFIX in SHELX), and a rigid bond restraint (RIGU in SHELX). In addition, disordered C atoms that shared almost equivalent coordinates were refined with constrained displacement parameters (EADP in SHELX). The second nitrophenyl group, that is centred around a twofold rotation axis, was disordered (PLAT230_ALERT_2_B) and refined with a rigid bond restraint (RIGU in SHELX). Solvent was poorly resolved in the crystal structure and it was necessary to use the SQUEEZE routine in Platon during the final refinement cycles (PLAT602_ALERT_2_A checkCIF alert).[36,37] SQUEEZE found four 511 Å^3 voids with disordered electron counts of 141 (e-). As a result, 6 $CDCl_3$ and 6 $C_4H_8O_2$ solvent molecules were tentatively added to the unit cell atom count (PLAT043_ALERT_1_A, CHEMW03_ALERT_2_A, and PLAT051_ALERT_1_A checkCIF alerts). For a displacement ellipsoid plot see **Supplementary Fig. 20**.

C9·4.66($CDCl_3$)·0.48(C_2H_3N)

The single crystal structure, **C9**·4.66($CDCl_3$)·0.48(C_2H_3N), crystallised from a $CDCl_3$/MeCN solution in the triclinic space group $P\bar{1}$. The asymmetric unit for this phase comprises one complete **C9** cage. In the structure, S atoms for two thiophene groups were disordered over two positions, C-S and C-C bond distance restraints were used to model the disordered parts (DFIX in SHELX) and free variables were used to determine site occupancies. Atoms that shared almost equivalent coordinates were refined with constrained displacement parameters (EADP in SHELX). The disordered solvent was modelled as a mixture of $CDCl_3$ and MeCN. It was necessary to refine some of the disordered solvent isotropically with bond distance restraints (DFIX and DANG in SHELX). For a displacement ellipsoid plot, see **Supplementary Fig. 21**.

C13$_{[8+12]}$·3.67(CH_2Cl_2)·6(CH_4O)·1.5(H_2O)

The single crystal structure, **C13**·3.67(CH_2Cl_2)·6(CH_4O)·1.5(H_2O), crystallised from a CH_2Cl_2/MeOH solution in the triclinic space group $P\bar{1}$. The asymmetric unit for this phase comprises ½ of a **C13** cage; the complete cage is related by inversion symmetry. The single crystal X-ray data was collected using synchrotron radiation, despite this X-ray resolution was poor and a 1.0 Å resolution limit was applied during refinement (THETM01_ALERT_3_A and PLAT340_ALERT_3_B checkCIF alerts) and the final wR_2 value was high (PLAT084_ALERT_3_B checkCIF alert). In the structure, it was not possible to accurately determine H atom positions. H atoms were therefore placed in estimated positions and refined using the riding model. The estimated positions of the disordered H atoms are unlikely to be correct resulting in unlikely close H-H contacts (PLAT413_ALERT_2_A, PLAT413_ALERT_2_B, and PLAT415_ALERT_2_B

checkCIF alerts), and D-H groups without acceptors (PLAT420_ALERT_2_B checkCIF alert). In the structure, one of dimethoxy aromatic groups of **C13** was disordered and modelled over two positions resulting in a high U(eq) for one of the methyl group C atoms (C71) (PLAT340_ALERT_3_B checkCIF alert). Due to additional disorder, three phenyl rings of **C13** were refined with constrained geometries (AFIX 66 in SHELX), 1,2 and 1,3 bond distance restraints were used to model **C13** and the disordered solvent molecules (DFIX and DANG in SHELX), and the structure was refined with a rigid bond restraint (RIGU in SHELX). Despite the disorder in the crystal structure, a solvent mask was not used during refinement. For a displacement ellipsoid plot, see **Supplementary Fig. 22**.

C14·2(C_4H_8O)

The single crystal structure, **C14**·2(C_4H_8O), crystallised from a CH_2Cl_2/THF solution in the triclinic space group $P\bar{1}$. The asymmetric unit for this phase comprises one complete **C14** cage and two THF molecules. Due to disorder, diffuse scatter beyond 0.85 Å was omitted during refinement (PLAT027_ALERT_3_A checkCIF alert), and two -CH_2=N-CH- groups were modelled over two positions. In the crystal structure two reasonably well ordered THF molecules were located between **C14** cages. Due to slight disorder, one of these THF molecules was refined with a rigid bond restraint (RIGU in SHELX) and for this molecule a -O-CH_2- group was modelled over two positions. For a displacement ellipsoid plot see, **Supplementary Fig. 23**.

C21-Tri^{2}Di^3·0.5(CH_2Cl_2)·0.25($C_4H_{10}O$)

The single crystal structure, **C21-Tri^{2}Di^3**·0.5(CH_2Cl_2)·0.25($C_4H_{10}O$), crystallised from a CH_2Cl_2/Et_2O solution in the triclinic space group $P\bar{1}$. Due to slight disorder of the cage structure, the C-S bond distances were restrained during refinement (DFIX in SHELX), and one methyl group was modelled over two positions. In the structure, the solvent was disordered and modelled as a mixture of partially occupied CH_2Cl_2 and Et_2O. The CH_2Cl_2 molecule was refined with C-Cl bond distance restraints (DFIX in SHELX). During refinement, all H-atoms were placed in estimated positions and refined using the riding model. For the disordered Et_2O molecules the $-CH_3$ H-atoms are unlikely to be in the correct position, resulting in there being close intermolecular contacts in the final structure (PLAT413_ALERT_2_A alert). For a displacement ellipsoid plot, see **Supplementary Fig. 24**.

C21-Tri^{4}Di^6·7.12(CH_2Cl_2)·5.12(C_2H_3N)·0.25(H_2O)

The single crystal structure, **C21-Tri^{4}Di^6**·7.12(CH_2Cl_2)·5.12(C_2H_3N)·0.25(H_2O), crystallised from a CH_2Cl_2/MeCN solution in the monoclinic space group $P2_1/n$. The asymmetric unit for this phase comprises one complete **C21-Tri^{4}Di^6** cage. The crystal data quality was poor and a

resolution limit of 1 Å was applied during refinement (THETM01_ALERT_3_A and PLAT027_ALERT_3_A checkCIF alert). In the crystal structure, **C21-Tri⁴Di⁶** is disordered; one thiophene group was modelled over two positions, and one thiophene group was modelled over three positions. For the disordered parts site occupancies were determined using free variables and one severely disordered part was refined with constrained displacement parameters (EADP in SHELX). Due to disorder, **C21-Tri⁴Di⁶** was refined with a rigid bond restraint (RIGU in SHELX), and C-C, C-N, S-C 1,2 and 1,3 of bond distance restraints were used during refinement (DFIX and DANG in SHELX), in addition to planarity restraints (FLAT in SHELX). Atoms that shared similar coordinates were refined with constrained displacement parameters (EADP in SHELX). It is likely that additional disorder of **C21-Tri⁴Di⁶** could not be resolved. Two CH_2Cl_2 molecules were located in the crystal structure, these were refined with C-Cl bond distance restraints (DFIX in SHELX). Solvent was poorly resolved in the large lattice voids, hence, it was necessary to use the SQUEEZE routine in Platon during the final refinement cycles (PLAT602_ALERT_2_A and PLAT049_ALERT_1_B checkCIF alert).[36,37] SQUEEZE found a 5825 $Å^3$ void with disordered electron count of 1575 (e-). As a result, 20.5 CH_2Cl_2 and 20.5 MeCN solvent molecules were tentatively added to the unit cell atom count (CHEMW03_ALERT_2_A, PLAT043_ALERT_1_A, and PLAT051_ALERT_1_A checkCIF alert). Due to disorder, and the limited resolution of the diffraction data, it was not possible to accurately determine H atom positions. H atoms were therefore placed in estimated positions and refined using the riding model. The estimated positions of the disordered H atoms are unlikely to be correct resulting in close inter- and intramolecular H-H contacts (PLAT410_ALERT_2_A, PLAT413_ALERT_2_A, and PLAT411_ALERT_2_B checkCIF alerts). For a displacement ellipsoid plot, see **Supplementary Fig. 25**.

C23·6.5(CH_2Cl_2)·7.5(C_2H_3N)

The single crystal structure, **C23**·6.5(CH_2Cl_2)·7.5(C_2H_3N), crystallised from a CH_2Cl_2/MeCN solution in the monoclinic space group *P*2/*c*. The asymmetric unit for this phase comprises with one complete **C23** cage molecule. Due to disorder, **C23** was refined with a rigid bond restraint (RIGU in SHELX) and diffuse scatter beyond 1.05 Å was omitted during refinement (THETM01_ALERT_3_A, PLAT027_ALERT_3_A, and PLAT340_ALERT_3_B checkCIF alerts). In the structure, a 1,4 substituted aromatic ring was disordered over two positions, site occupancies for the disordered parts were determined using a free variable. Disordered CH_2Cl_2 (1 ½ per **C23**) and MeCN (2 ½ per **C23**) were located in the structure, these were refined isotropically with bond distance restraints (DFIX in SHELX). Additional solvent molecules were too disordered to be accurately modelled in the large pores (PLAT602_ALERT_2_A and PLAT049_ALERT_1_B checkCIF alerts). Hence, the SQUEEZE in PLATON was used during the final refinement cycles.[36,37] SQUEEZE found a 4915 $Å^3$ void with disordered electron counts of 1298 (e-). As a result, 20 CH_2Cl_2 and 20 MeCN solvent molecules were tentatively added to the unit cell atom count (CHEMW03_ALERT_2_A, PLAT043_ALERT_1_A, and

PLAT051_ALERT_1_A checkCIF alerts). For a displacement ellipsoid plot, see **Supplementary Fig. 26**.

C26·12.25($CHCl_3$)·12.25($C_4H_{10}O$)

The single crystal structure, **C26**·12.25($CHCl_3$)·12.25($C_4H_{10}O$), crystallised from a $CHCl_3$/Et_2O solution in the tetragonal space group $I4_1/a$. The asymmetric unit for this phase comprises ¼ of a **C26** cage molecule. Due to disorder, **C26** was refined with a rigid bond restraint (RIGU in SHELX), and a 1.1 Å resolution limit was applied during refinement (THETM01_ALERT_3_A and PLAT027_ALERT_3_A checkCIF alerts). In addition, the seven aromatic rings were refined with constrained geometries (AFIX 66 in SHELX). Two of these were modelled over two positions and the severely disordered parts being refined isotropically. Additional disorder was modelled using 1,2 and 1,3 bond distances restraints (DFIX, DELU, and SADI in SHELX), and planarity restraints (FLAT in SHELX). Solvent molecules were too disordered to accurately model in the large pores (PLAT602_ALERT_2_A and PLAT049_ALERT_1_B checkCIF alerts). Hence, the SQUEEZE in PLATON was used during the final refinement cycles.[36,37] SQUEEZE found a 19258 $Å^3$ void with a disordered electron count of 4908 (e-). As a result, 49 $CHCl_3$ and 49 Et_2O solvent molecules were tentatively added to the unit cell atom count (CHEMW03_ALERT_2_A, PLAT043_ALERT_1_A, and PLAT051_ALERT_1_A checkCIF alerts). Due to disorder, and the limited resolution of the diffraction data, it was not possible to accurately determine H atom positions. H atoms were therefore placed in estimated positions and refined using the riding model. The estimated positions of the disordered H atoms are unlikely to be correct resulting in close inter- and intramolecular H-H contacts (PLAT410_ALERT_2_A and PLAT411_ALERT_2_B checkCIF alerts). For a displacement ellipsoid plot, see **Supplementary Fig. 27**.

Supplementary References

1. Santolini, V., Miklitz, M., Berardo, E. & Jelfs, K. E. Topological landscapes of porous organic cages. *Nanoscale* **9,** 5280–5298 (2017).

2. De Rycke, N., Marrot, J., Couty, F. & David, O. R. P. Synthesis and characterisation of hexasubstituted azacryptands. *Tetrahedron Lett.* **51,** 6521–6525 (2010).

3. Lydon, D. P., Campbell, N. L., Adams, D. J. & Cooper, A. I. Scalable synthesis for porous organic cages. *Synth. Commun.* **41,** 2146–2151 (2011).

4. Jones, J. T. A. *et al.* Modular and predictable assembly of porous organic molecular crystals. *Nature* **474,** 367–371 (2011).

5. Briggs, M. E. & Cooper, A. I. A perspective on the synthesis, purification, and characterisation of porous organic cages. *Chem. Mater.* **29,** 149–157 (2017).

6. Vogel, H. A better way to construct the sunflower head. *Math. Biosci.* **44,** 179–189 (1979).

7. RDKit: Open-Source Cheminformatics Software. **2013,**

8. Rappe, A. K., Casewit, C. J., Colwell, K. S., Goddard, W. A. & Skiff, W. M. UFF, a full periodic table force field for molecular mechanics and molecular dynamics simulations. *J. Am. Chem. Soc.* **114,** 10024–10035 (1992).

9. Harder, E. *et al.* OPLS3: A force field providing broad coverage of drug-like small molecules and proteins. *J. Chem. Theory Comput.* **12,** 281–296 (2016).

10. VandeVondele, J. *et al.* Quickstep: Fast and accurate density functional calculations using a mixed Gaussian and plane waves approach. *Comput. Phys. Commun.* **167,** 103–128 (2005).

11. Perdew, J., Burke, K. & Ernzerhof, M. Generalised gradient approximation made simple. *Phys. Rev. Lett.* **77,** 3865–3868 (1996).

12. Khaliullin, R. Z., VandeVondele, J. & Hutter, J. Efficient linear-scaling density functional theory for molecular systems. *J. Chem. Theory Comput.* **9,** 4421–4427 (2013).

13. Grimme, S., Antony, J., Ehrlich, S. & Krieg, H. A consistent and accurate ab initio parametrization of density functional dispersion correction (DFT-D) for the 94 elements H-Pu. *J. Chem. Phys.* **132,** (2010).

14. Stejskal, E. O. & Tanner, J. E. Spin diffusion measurements: Spin echoes in the presence of a time dependent field gradient. *J. Chem. Phys.* **42,** 288–292 (1965).

15. Parsons, S. ECLIPSE. (2004).

16. Sheldrick, G. SADABS. (2008).

17. Krause, L., Herbst-Irmer, R., Sheldrick, G. M. & Stalke, D. Comparison of silver and molybdenum microfocus X-ray sources for single-crystal structure determination. *J. Appl. Crystallogr.* **48,** 3–10 (2015).

18. Sheldrick, G. M. Experimental phasing with SHELXC/D/E: Combining chain tracing with density modification. *Acta Crystallogr. Sect. D Biol. Crystallogr.* **66,** 479–485 (2010).

19. Sheldrick, G. M. SHELXT - Integrated space-group and crystal-structure determination. *Acta*

Crystallogr. Sect. A Found. Crystallogr. **71,** 3–8 (2015).

20. Sheldrick, G. M. A short history of SHELX. *Acta Crystallogr. Sect. A Found. Crystallogr.* **64,** 112–122 (2008).

21. Sheldrick, G. M. Crystal structure refinement with SHELXL. *Acta Crystallogr. Sect. C Struct. Chem.* **71,** 3–8 (2015).

22. Dolomanov, O. V., Bourhis, L. J., Gildea, R. J., Howard, J. A. K. & Puschmann, H. OLEX2: A complete structure solution, refinement and analysis program. *J. Appl. Crystallogr.* **42,** 339–341 (2009).

23. Grawe, T., Schrader, T., Gurrath, M., Kraft, A. & Osterod, F. Self-organisation of spheroidal molecular assemblies in polar solvents. *Org. Lett.* **2,** 29–32 (2000).

24. Vacogne, C. D. & Schlaad, H. Primary ammonium/tertiary amine-mediated controlled ring opening polymerisation of amino acid N-carboxyanhydrides. *Chem. Commun.* **51,** 15645–15648 (2015).

25. Granzhan, A., Schouwey, C., Riis-Johannessen, T., Scopelliti, R. & Severin, K. Connection of metallamacrocycles via dynamic covalent chemistry: a versatile method for the synthesis of molecular cages. *J. Am. Chem. Soc.* **133,** 7106–7115 (2011).

26. Roelens, S., Vacca, A., Francesconi, O. & Venturi, C. Ion-pair binding: is binding both binding better? *Chem. Eur. J.* **15,** 8296–8302 (2009).

27. Vacca, A., Nativi, C., Cacciarini, M., Pergoli, R. & Roelens, S. A new tripodal receptor for molecular recognition of monosaccharides. A paradigm for assessing glycoside binding affinities and selectivities by 1H NMR spectroscopy. *J. Am. Chem. Soc.* **126,** 16456–16465 (2004).

28. Blackburn, O. A., Coe, B. J., Helliwell, M. & Raftery, J. Syntheses, structures, and electronic and optical properties of platinum(II) complexes of 1,3-bis(imino)benzene-derived pincer ligands. *Organometallics* **31,** 5307–5320 (2012).

29. Catala, L. *et al.* Towards a better understanding of the magnetic interactions within m-phenylene α-nitronyl imino nitroxide based biradicals. *Chem. Eur. J.* **7,** 2466–2480 (2001).

30. Wang, L., Chai, Y., Tu, P., Sun, C. & Pan, Y. Formation of [M+15]+ ions from aromatic aldehydes by use of methanol: in-source aldolisation reaction in electrospray ionization mass spectrometry. *J. Mass Spectrom.* **46,** 1203–1210 (2011).

31. Carella, A. *et al.* Rigid chain ribbon-like metallopolymers. *J. Polym. Sci. Part A* **52,** 2412–2421 (2014).

32. Zhang, W., Kraft, S. & Moore, J. S. Highly active trialkoxymolybdenum(VI) alkylidyne catalysts synthesized by a reductive recycle strategy. *J. Am. Chem. Soc.* **126,** 329–335 (2004).

33. Bounos, G. *et al.* Controlling chain conformation in conjugated polymers using defect inclusion strategies. *J. Am. Chem. Soc.* **133,** 10155–10160 (2011).

34. Jiang, S. *et al.* Selective gas sorption in a [3+2] 'propeller' cage crystal. *Chem. Commun.* **47,** 8919–8921 (2011).

35. Kotha, S., Kashinath, D., Lahiri, K. & Sunoj, R. B. Synthesis of C3-symmetric nano-sized polyaromatic compounds by trimerisation and Suzuki–Miyaura cross-coupling reactions. *Eur.*

J. Org. Chem. **2004,** 4003–4013 (2004).

36. Spek, A. PLATON, An Integrated Tool for the Analysis of the Results of a Single Crystal Structure Determination. *Acta Crystallogr. Sect. A* **46,** c34 (1990).

37. Van Der Sluis, P. & Spek, A. BYPASS: an effective method for the refinement of crystal structures containing disordered solvent regions. *Acta Crystallogr. Sect. A* **46,** 194–201 (1990).
