# Supplementary material for: High-throughput discovery of organic cages and catenanes using computational screening fused with robotic synthesis
Source: Nat Commun. 2018 Jul 20;9:2849. doi: 10.1038/s41467-018-05271-9 (PMC6054661; doi:10.1038/s41467-018-05271-9)
Supplement: Supplementary file 2 — Description of Additional Supplementary Files [file 41467_2018_5271_MOESM2_ESM.pdf]

Description of Additional Supplementary Files

File Name: Supplementary Data 1
Description: The full set of computational structures generated in our study.
